# Discrimination of Green Coffee (*Coffea arabica* and *Coffea canephora*) of Different Geographical Origin Based on Antioxidant Activity, High-Throughput Metabolomics, and DNA RFLP Fingerprinting

**DOI:** 10.3390/antiox12051135

**Published:** 2023-05-21

**Authors:** Giuseppe Mannino, Ronja Kunz, Massimo E. Maffei

**Affiliations:** 1 Department of Life Sciences and Systems Biology, University of Turin, Via Quarello 15/A, 10135 Turin, Italy; massimo.maffei@unito.it; 2 Department of Chemistry, University of Cologne, Zülpicher Straße 47, D-50939 Köln, Germany; rkunz3@smail.uni-koeln.de

**Keywords:** melatonin, caffeine, chemical fingerprinting, DNA PCR-RFLP, lipidomics, reducing activity, *5S-rRNA-NTS*, *trnL*, molecular fingerprinting

## Abstract

The genus *Coffea* is known for the two species *C. arabica* (CA) and *C. canephora* (CC), which are used to prepare the beverage coffee. Proper identification of green beans of coffee varieties is based on phenotypic and phytochemical/molecular characteristics. In this work, a combination of chemical (UV/Vis, HPLC-DAD–MS/MS, GC–MS, and GC-FID) and molecular (PCR-RFLP) fingerprinting was used to discriminate commercial green coffee accessions from different geographical origin. The highest content of polyphenols and flavonoids was always found in CC accessions, whereas CA showed lower values. ABTS and FRAP assays showed a significant correlation between phenolic content and antioxidant activity in most CC accessions. We identified 32 different compounds, including 28 flavonoids and four N-containing compounds. The highest contents of caffeine and melatonin were detected in CC accessions, whereas the highest levels of quercetin and kaempferol derivatives were found in CA accessions. Fatty acids of CC accessions were characterized by low levels of linoleic and *cis* octadecenoic acid and high amounts of elaidic acid and myristic acid. Discrimination of species according to their geographical origin was achieved using high-throughput data analysis, combining all measured parameters. Lastly, PCR-RFLP analysis was instrumental for the identification of recognition markers for the majority of accessions. Using the restriction enzyme *AluI* on the *trnL-trnF* region, we clearly discriminated *C. canephora* from *C. arabica*, whereas the cleavage performed by the restriction enzymes *MseI* and *XholI* on the *5S-rRNA-NTS* region produced specific discrimination patterns useful for the correct identification of the different coffee accessions. This work extends our previous studies and provides new information on the complete flavonoid profile, combining high-throughput data with DNA fingerprinting to assess the geographical discrimination of green coffee.

## 1. Introduction

Coffee is one of the world’s most popular beverages, and plants are currently cultivated in about 80 countries on four continents [1,2]. Three main plant species are used for commercial purposes and for coffee production, namely, *Coffea arabica* L. (also known as Arabica coffee), *C. canephora* Pierre ex A. Froehner (also known as Robusta coffee), and *C. liberica* Bull. ex Hiern (also knowns as Liberian/Liberica coffee or Excelsa coffee) [3]. Among these three species, the most commercially important is *C. arabica*, which accounts for more than 95% of the world’s coffee production [4].

Since ancient times, coffee has always attracted consumer interest for its potential effects on cognitive function and pain [5]. The main chemical constituent exerting pharmacological functions is the alkaloid caffeine (which is present from 1.5% to 2.4% *w*/*w* depending on the species [1]). However, because coffee records the highest amount of caffeine in comparison to other plant species, it was considered an exclusively recreational beverage, whose intake should be limited to avoid potential adverse effects on human health [6]. On the other hand, recent scientific evidence demonstrated the inconsistency of this claim, ascribing it to specific population groups (i.e., children and adolescents) and even suggesting an increase in its consumption during specific physiological conditions, such as pregnancy and lactation [7]. In addition, it has recently been pointed out that other bioactive compounds are present in coffee in addition to caffeine [8]. Indeed, a variety of chlorogenic acids, which are the most abundant polyphenolic molecules of green coffee [9], hydroxycinnamates (e.g., caffeoylquinic acids, feruloylquinic acids, and p-coumaroylquinic acids) [9,10,11,12,13], carbohydrates, and lipid constituents, such as diterpenes and fatty acids (free and esterified), may also be found in coffee beans [14]. These compounds can be extracted using either conventional procedures or microwave, ultrasound, or supercritical CO_2_ extractions [15].

Coffee processing requires unequivocal identification of both *C. arabica* and *C. canephora* accessions, which is currently performed on the basis of coffee bean morphology or by chemical characterization using gas chromatography or liquid chromatography coupled with mass spectrometry [16], spectroscopic techniques [17,18], and nuclear magnetic resonance [19,20], with both untargeted fingerprinting (i.e., metabolomics-based discrimination without identifying the metabolites) and targeted chemical profiling (i.e., performed with the identification and quantification of metabolites) [21]. Using chemometric-driven approaches [22], the main metabolites monitored during chemical fingerprinting are mainly bioactive components characteristic of the genus *Coffea*, with particular reference to organic acids, chlorogenic acids, and caffeine [23]. Furthermore, hyperspectral imaging (1000–2500 nm) has been successfully used for the prediction of total lipid content in intact green coffee beans [24]. Storage conditions of green coffee may change the chemical composition, with modification of the chemical profile involving lipids, amino acids, and phenolic compounds [25,26], and the composition of the coffee green beans can be indicative of the sensory quality of the coffee brew [27]. 

Previous studies have shown that it is possible to use the chemometrics of chlorogenic acids and caffeine to assess the geographical origin of green coffee beans [28]. Discriminant analysis using partial least squares (PLS-DA) was also applied to discriminate genealogical groups of *C. arabica* [29], and the same methodology was successful when the fatty acid composition of green coffee beans was analyzed for the discrimination of *C. arabica* from different geographical origin [30]. Despite the undiscussed power of chemometrics, the chemical composition of plants is affected by environmental and/or developmental factors or by the method of sample storage [31], meaning that the same genotype may express different chemical patterns or, conversely, that different genotypes may respond to the same environmental pressure with the same phenotypic expression [32]. By contrast, DNA analysis relies on the presence of molecules with higher stability, and DNA-based methods have become widely employed techniques for a rapid and unequivocal identification of plants [33]. Consequently, an increasing number of gene sequences are now available for DNA barcoding of flowering plants [34]. Recently, we showed that the combination of genomic analysis (using ITS DNA PCR-RFLP) and gene product (phenolic compounds and fatty acids) chemometrics allowed the geographical differentiation of pistachio varieties according to their geographical origin [35]. 

The aim of this work was to combine DNA fingerprinting, metabolomic chemometrics, and antioxidant assays to obtain the genomic and functional discrimination of green coffee from different geographical origin. In particular, the chemical composition of 20 green coffee commercial accessions of *C. arabica* and *C. conephora* was analyzed using both UV/Vis and chromatographic methodologies, while the genomic profile was assessed using DNA PCR-RFLP on *5S-rRNA-NTS* and *tnrL* genomic regions. In our previous work, we analyzed, in the same coffee accessions, the discriminant power of the chlorogenic acids, which represent the most important polyphenols of green coffee [28]. In this work, we extended our search for chemical and molecular markers in green coffee and focused our attention on the flavonoid moiety, because of the scant information in the scientific literature on the flavonoid profile of green bean coffee, and we assessed how it may be affected by variations such as geographic origin, variety, and species. Moreover, the combination of chemical and molecular data, together with the evaluation of antioxidant properties, provides an interesting and high-throughput approach for the unambiguous identification of commercial coffee beans of different geographical origins.

## 2. Materials and Methods

### 2.1. Plant Material and Extraction

*Coffea arabica* L. and *C. canephora* Pierre ex A. Froehner (Rubiaceae) green coffee beans were kindly provided by Green Elite Coffee (Genova, Italy). Table 1 summarizes the species, cultivars, and origin of the different green coffee accessions.

For the preparation of green coffee extracts for metabolomic studies, 5 g of each sample was ground in order to obtain a fine powder. The obtained powder was extracted using 25 mL of a hydroalcoholic mixture composed of 70% (*v*/*v*) ethanol. Samples were then sonicated for 30 min at room temperature and macerated in the dark for 48 h. After a cleanup step via centrifugation (10 min, 10,000× *g*, 4 °C), the supernatant was transferred into a clean tube, and the extraction process was repeated twice. After centrifugation, the resulting supernatants were combined. For each sample, three extractions were performed. Lastly, samples were stored at −20 °C until the analytical determinations were performed.

### 2.2. Total Polyphenol Content (TPC)

The total polyphenol content (TPC) was quantified via the reduction of phosphotungstic–phosphomolybdic acid (Folin–Ciocâlteu reagent) to blue pigments in an alkaline solution [36]. Briefly, 5 μL of appropriately diluted sample was incubated with 5 μL of Folin–Ciocâlteu reagent, together with 10 μL of 20% (*w*/*v*) Na_2_CO_3_ and 176 μL of deionized water (ddH_2_O) in a 96-well plate. Samples were then incubated at 80 °C for 1 min, and the absorbance was measured at 725 nm in a microplate reader (NB-12-0035, Neo Biotech, Nanterre, France). The amount of total polyphenols was expressed as mg gallic acid equivalent (GAE)·100 g^−1^ of fresh weight (FW) using a calibration curve of pure gallic acid (VWR International, Radnor, PA, USA). All measurements were performed in three different biological replicates.

### 2.3. Total Flavonoid Content

The total flavonoid content (TFdC) was quantified using the aluminum complex reaction [37]. Briefly, after 5 min incubation of 40 μL of properly diluted extract with 6 μL of 5% (*w*/*v*) NaNO_2_, 6 μL of 10% (*w*/*v*) AlCl_3_ was added. After 6 min, 80 μL of 4% (*w*/*v*) NAOH and 68 μL of ddH_2_O were dispensed into the wells. Samples were mixed again, and, after 15 min, the absorbance was read at 510 nm using a microplate reader (NB-12-0035, Neo Biotech, Nanterre, France). The TFdC was expressed as mg of rutin equivalent (RE)·100 g^−1^ FW, using an external calibration curve of pure rutin (VWR International, Radnor, PA, USA). All measurements were performed in three different biological replicates.

### 2.4. Total Flavonol Content

The total flavonol content (TFlC) was quantified by adapting the protocol of Miliauskas et al. [38] to a 96-well plate reader. Briefly, 10 μL of properly diluted extract was combined with 20 μL of 2% (*w*/*v*) NaNO_2_ and 0.5% (*w*/*v*) CH_3_COONa. After a 2.5 h incubation at 20 °C, spectrophotometric determination was performed at 440 nm using the NB-12-0035 microplate reader. The TFlC was expressed as mg of quercetin equivalent (QE)·100 g^−1^ FW, using an external calibration curve of pure quercetin (VWR International, Radnor, PA, USA). All measurements were performed in three different biological replicates.

### 2.5. Total Flavan-3-ol Content

The total flavan-3-ol content (TPAC) was evaluated using the 4-(dimethylamino)cinnamaldehyde (DMAC) assay, according to Prior and coworkers [39], with minor modifications. Briefly, 56 μL of properly diluted green coffee bean extract was incubated with 168 μL of DMAC solution (0.1% (*w*/*v*) dissolved in 75% (*v*/*v*) ethanol acidified with 12.5% HCl). The TPAC was quantified using an external calibration curve of pure A2-type proanthocyanidin (Extrasynthese^®^, Genay, France). The results were expressed as mg A2-type PAC equivalent (PACE)·100 g^−1^ FW. All measurements were performed in three different biological replicates.

### 2.6. Radical-Scavenging Activity

In order to evaluate the radical-scavenging activity, 7 mM 2,2′-azino-bis(3-ethylbenzothiazoline-6-sulfonic acid) (ABTS) was dissolved in water and left to react with 2.45 mM K_2_S_2_O_8_. After a 16 h incubation in the dark and at RT, the radical ABTS^+^ solution was diluted with methanol until an absorbance of 0.70 was measured at 734 nm. Consequently, 20 μL of properly diluted samples were incubated with 180 μL of radical ABTS^+^ in a 96-multiwell plate. After 5 min incubation, the decolorization of the resulting mixture was evaluated by reading the absorbance at 734 nm [40]. An external calibration curve of 6-hydroxy-2,5,7,8-tetramethylchroman-2-carboxylic acid (Trolox) was used to estimate the radical-scavenging activity, and the results were expressed as mmol of Trolox equivalent (TE)·100 g^−1^ FW. All measurements were repeated three times.

### 2.7. Reducing Activity

In order to evaluate the reducing potential, the ferric reducing antioxidant power (FRAP) assay was performed according to Svečnjak and coworkers [41]. Briefly, 20 μL of properly diluted sample were incubated with the reaction buffer consisting of 0.3 M acetate buffer (pH 3.6), 10 mM 2,4,6-Tri(2-pyridyl)-s-triazine (TPTZ) and 20 mM FeCl_3_ mixed in an 8:1:1 (*v*/*v*/*v*) ratio. After 20 min incubation, absorbance was read at 595 nm. An external Trolox calibration curve was used for quantification of reducing power, and the results were then expressed as mmol TE·100 g^−1^ FW. All measurements were repeated three times.

### 2.8. Identification and Quantification of Plant Bioactive Compounds

The same green coffee bean extracts used for spectrophotometric quantifications were utilized to evaluate their phytochemical profiling by HPLC analysis (Agilent Technologies 1100 instrument, Santa Clara, CA, USA) coupled to a diode array detector (DAD) and a 6330 Series Ion Trap LC–MS System ((Agilent Technologies 1100 instrument, Santa Clara, CA, USA) equipped with an electrospray ionization (ESI) source. The chromatographic separation was carried out using a constant flow rate (0.2 mL·min^−1^) through a C18 Luna reverse phase column (3.00 μm, 150 × 3.0 mm i.d., Phenomenex, Aschaffenburg, Germany), thermo-maintained at 25 °C by an Agilent 1100 HPLC G1316A Column Compartment. The UV/Vis spectra were recorded between 220 and 650 nm, and the chromatographic profiles were registered at 280, 360, and 520 nm. Tandem mass spectrometry analyses were performed operating in negative mode for polyphenol compounds and in positive mode for alkaloids or melatonin, as previously described [42,43,44]. Compound identification was carried out by comparing the obtained retention times and UV/Vis–MS spectra with those of reference compounds. For both chromatographic analyses, separation was obtained by a binary solvent system consisting of MilliQ H_2_O acidified with 0.1% (*v*/*v*) formic acid (Solvent A) and acetonitrile acidified with 0.1% (*v*/*v*) formic acid (Solvent B). The initial concentration of the solvents was set at 90% (*v*/*v*) A and 10% (*v*/*v*) B for 5 min; then, the concentration of Solvent B was raised to 55% (*v*/*v*) A in 25 min, and finally 70% (*v*/*v*) B in 25 min. The starting solvent composition was restored at the end of each chromatographic run and maintained for an additional 10 min before the subsequent injection. For each analysis, the sample injection volume was set at 10 μL.

### 2.9. Identification and Quantification of Fatty Acids

In order to obtain fatty acid methyl esters (FAMEs), 10 mg of previously ground green coffee bean powder was directly trans-esterified using 500 μL of 10% (*w*/*v*) boron trifluoride dissolved in methanol and incubated at 80 °C for 1 h. Fifty micrograms of heptadecanoic acid (C17:0) was added as an internal standard before incubation, with the purpose of monitoring the exhaustiveness of the trans-esterification reaction. FAMEs were then purified with two serial additions of 500 μL of *n*-hexane and water. After separation of the organic phase, water was removed from the samples by addition of MgSO_4_. FAME identification and quantification was performed by injecting 5 μL of the organic phase using both GC–MS (5975T, Agilent Technologies, Santa Clara, CA, USA) and GC-FID (GC-2010 Plus, SHIMADZU, Kyoto, Japan). In both systems, the GC carrier gas was helium which was set at constant flux equal to 1 mL·min^−1^, and the separation was obtained with a nonpolar capillary column ZB5-MS (30 m length, 250 μm diameter, stationary phase thickness of 0.25 μm, 5% phenyl-arylene, and 95% poly-dimethyl siloxane) (Phenomenex, Torrance, CA, USA). The injector temperature was set at 250 °C, while the oven was initially set at 60 °C, held for 1 min, and raised to 180 °C (10.0 °C·min^−1^ and held for 1 min). Then, the temperature was raised to 230 °C (1.0 °C·min^−1^ and held for 2 min) and to 320 °C (15 °C·min^−1^ and held for 5 min). For MS analysis, the ionization energy of the ion source was set to 70 eV, and the acquisition mode was set to 50–350 *m*/*z*. Compounds were identified through a comparison of mass fragmentation spectra with the reference NIST 98 spectra and by comparison of Kovats indices. FAME quantification was obtained using a calibration curve of pure FAME standard. At least three replicates were run for each coffee accession.

### 2.10. DNA Extraction, PCR Amplification, Subcloning, and Sequencing

Whole green coffee beans were pulverized in liquid nitrogen using a mortar and pestle. Genomic DNA was extracted and quantified according to a previously published protocol [44]. Twenty nanograms of genomic DNA was used as a template for PCR amplification with specific primers for *5S-rRNA-NTS* (F: GTGCTTGGGCGAGAGTAGTA; R: TTAGTGCTGGTATGATCGCA) or *trnL-trnF* (F: CGAAATCGGTAGACGCTACG; R: ATTTGAACTGGTGACACGAG) regions. PCR products were separated by 1.5% (*w*/*v*) agarose gel electrophoresis and visualized by GelRed staining (Biotium, Fremont, CA, USA) under UV light. PCR products were purified with the NucleoSpin PCR Cleanup kit (Macherey-Nagel) following the manufacturer’s instructions. The purified products were then used for subcloning via the TOPO-TA Cloning Kit (Thermo Fisher Scientific, Waltham, MA, USA) and cloned in *Escherichia coli* Subcloning DH5α Efficiency Competent Cells (Invitrogen, Waltham, MA, USA). The colonies containing DNA inserts were picked and grown overnight in 5 mL of Luria–Bertani (LB) liquid medium fortified with 100 μg·mL^−1^ ampicillin. After purification using a NucleoSpin Plasmid Miniprep Kit (Macherey-Nagel), the plasmid DNA was used as a template for sequencing (Macrogen, Wageningen, Holland). Both forward and reverse DNA strands were sequenced using an outsourcing company.

### 2.11. PCR-RFLP Analysis

After analyzing the results obtained from outsourced sequencing, the PCR products obtained from amplification of the *5S-rRNA-NTS* and *trnL-trnF* regions were used for controlled enzymatic digestion. Specifically, the *5S-rRNA-NTS* regions were cut by *MseI* and *XhoI* (NEB, New England Biolabs, Ipswich, MA, USA) at 37 °C for 60 min, followed by inactivation via incubation at 85 °C for 20 min. On the other hand, PCR products from *trnL-trnF* region were digested at 37 °C for 60 min with *AluI* (NEB, New England Biolabs, Ipswich, MA, USA), followed by enzymatic inactivation at 65 °C for 20 min. Before utilization, all enzymes were resuspended in the appropriate buffer as described by the manufacturer’s protocol. Following enzymatic reactions, 1 µL of each digestion reaction was analyzed by capillary gel electrophoresis (CGE) using the Agilent 2100 Bioanalyzer (Agilent Technologies) and the DNA 1000 LabChip Kit (Agilent Technologies), as previously described [45].

### 2.12. Statistical Analysis

For all analyses, data were expressed as the mean ± standard deviation of at least three biological replicates. ANOVA followed by the Tukey–Kramer HSD test (*p* < 0.05) was used to test for significant differences between analytical measurements. Principal component analysis (PCA) was performed on the chemical data using the extraction covariate matrix and varimax rotation. Molecules used for the PCA were chosen on the basis of their lowest internal variability (i.e., with the lowest CV among the biological replicates) All statistical analyses were performed using SPSS v. 28 software. The cladogram of gene sequences was performed with CLC software using the neighbor joining (NJ) method. Bootstrap values were calculated from 100 resamples of the alignment data. Heatmaps were generated using Heatmapper (http://www.heatmapper.ca/, accessed on 1 March 2023).

## 3. Results and Discussion

This work extends our previous studies on the chemical and molecular discrimination of green coffee by adding new information on the polyphenolic moieties beyond chlorogenic acids (see [28]) and through DNA fingerprinting. Therefore, the first part of this study analyzes the phytochemical profile of *C. arabica* and *C. canephora* green beans from different geographical origins via high-throughput analysis of spectrophotometric assays, GC–MS/FID, and HPLC-DAD–ESI-MS/MS data, whereas in the second part the genomic discrimination of all coffee accessions is obtained by DNA RFLP fingerprinting.

### 3.1. The Total Polyphenol Content Correlates with the Antioxidant Power of Green Coffee Extracts

**Figure 1:**
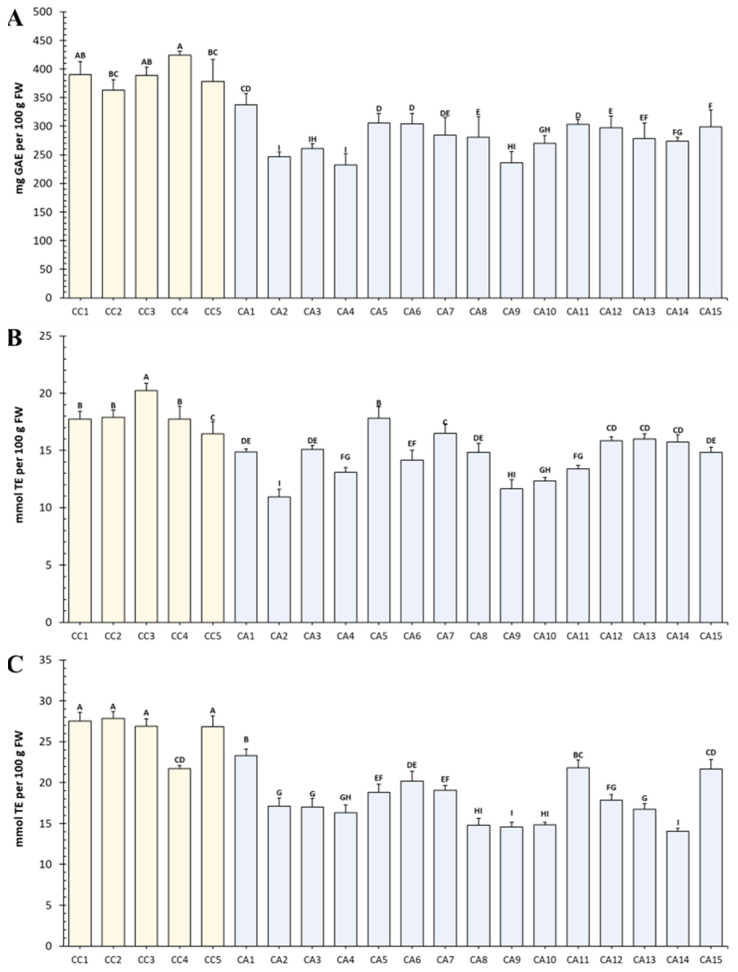
Antioxidant activity and total phenolic compounds content of green coffee of different geographical origin. (**A**) Total polyphenol content (TPC), expressed as mg of gallic acid equivalents (GAE). (**B**) Radical-scavenging activity (ABTS), expressed as mmol of Trolox^®^ equivalents (TE). (**C**) Reducing power activity (FRAP), expressed as mmol TE. CA, *Coffea arabica*, blue column; CC, *C. canepehora*, yellow column. Numbers correspond to the different green coffee accessions listed in Table 1. Values are represented as the mean ± SD of three different biological replicates. For each column, different luppercase letters indicate significant differences (*p* ≤ 0.05, as measured by Tukey’s multiple range test).

In general, a significant (*p* < 0.05) difference was found in the total polyphenol content (TPC) among the accessions of the two species (Figure 1A). TPC ranged between 232.31 ± 19.42 and 423.93 ± 6.82 mg GAE 100 g^−1^ FW. Similar TPC values were previously reported for green coffee beans from different regions of Ethiopia [46] and for coffee beans before the roasting process [47].

Interestingly, the highest values were always measured in *C. canephora* (CC) accessions (388.90 ± 22.43 mg GAE·100 g^−1^), whereas *C. arabica* (CA) had TPC about 30% lower than CC (280.55 ± 28.72 mg GAE·100 g^−1^). Moreover, a consistent variability in TPC was observed in the CA group. Among the analyzed green coffee beans, the variety Vietnam Unwashed (CC4) had the highest values among all CC accessions, whereas the variety Low Grade from Kenya (CA1) had the highest values among CA accessions. On the other hand, the cultivar Jolly Quartz from Uganda (CC2) and Natural Terraforte from Brasil (CA4) were the varieties with the lowest TPC among CC and CA accessions, respectively.

Figure 1 also shows the variation in the radical-scavenging activity and reducing power of green coffee beans. In general, ABTS activity (Figure 1B) was always higher in CC accessions and was associated with the TPC content (Figure 1A), with the sole exception being CC4 (from Uganda) that showed similar values to CA11 (from Guatemala) and CA15 (from Honduras). With regard to FRAP (Figure 1C), the highest activity was found for CC3 (from Kenya); even in this case, it was associated with a high TPC content (Figure 1A). 

The reduction reaction carried out by the molybdenum and tungsten salts contained in the Folin–Ciocâlteu reagent used for the TPC analysis is unable to discriminate individual classes of polyphenols or prevent potential interferences from molecules having a phenol linked to the chemical scaffold [42]. Consequently, although this assay has become a common methodology to gain information on the polyphenol content of plant extracts, it turns out to be an assay that leads to overestimated results [48]. Other chemical assays based on selective reactions have been developed to gain more reliable analytical data on the content of specific bioactive compounds, while excluding potential interferences. These include the aluminum complexation assay for the total flavonoid content (TFdC), quantification of total flavonol content (TFlC) [49], and the DMAC assay for the total proanthocyanidin content (TPAC) [50]. As expected, a preliminary search of anthocyanins or anthocyanidins resulted in the complete absence of these compounds. Therefore, the term flavonoids used herein does not include anthocyanins. Therefore, we evaluated the polyphenol content using TFdC, TFlC, and TPAC, as reported in Table 2.

In general, the TFdC is in line with the literature data for both *C. arabica* and *C. canpehora* accessions [51,52]. Significant (*p* < 0.05) differences were found among accessions belonging to the two coffee species. In general, CC samples (102.20 ± 22.62 mg RE·100 g^−1^) showed a twofold higher value in comparison to CA accessions (57.02 ± 27.33 mg RE·100 g^−1^). In particular, TFdC ranged between 31.23 ± 1.05 (CA9) and 136.11 ± 18.43 (CA1) mg RE·100 g^−1^ FW. However, the accession with the highest TFdC value was Arabica Low Grade from Kenya (CA1), followed by CC1, CC3, CC2, and CC4. On the other hand, the Brazilian CC5 recorded a TFdC comparable to the accession from Colombia (CA11) and HB ep from Guatemala (CA14). The CC5 accession of *C. canephora* is also known as caracol (also called peaberry) and has a natural mutation inside its cherry that affects about 5% of the world’s coffee. Its phenotype is characterized by a single, rather than a double bean that appears smaller, denser, and with a more rounded shape with respect to the wildtype [53,54].

Flavonols are a particular class of flavonoids characterized by a 3-hydroxyflavone backbone. Unlike flavanols, such as catechins and proanthocyanidins that are detected by the DMAC assay, a double bond is present between C2 and C3 of the C ring [42]. This particular chemical structure allows the aluminum ion to chelate with the ketone group of the C ring and the hydroxyl of the A ring, resulting in the formation of a green complex [55]. Regarding TFlC, the highest value was found in CA1 from Kenya, while most of the CC accessions showed the highest values, with the exception of CC5 that was discriminated by its low TFlC with respect to all other CA accessions. The flavonol content was in line with previous studies [52].

Concerning TPAC, a statistical difference in flavan-3-ol content between CC (2.20 ± 0.32 mg PACE·100 g^−1^) and CA (2.01 ± 0.27 mg PACE·100 g^−1^) accessions was not observed (*p* > 0.05). However, the cultivar Jolly Quartz from Uganda (CC1) and HB ep from Guatemala (CC10) had the highest values, followed by CC2, CA7, CA9, and CA6. In contrast, the lowest contents were observed for the cultivars Arabika K3 (CA2) and Arabika K2 (CA3), both from Kenya. The data were in line with previous investigations [56,57].

Because TPC, TFdC, TFlC, and TPAC data can be related to the antioxidant properties of a plant extract [58], we evaluated the potential correlation existing between these data and ABTS and FRAP assays. Low values were obtained for TFlC (ρ_TFlC/ABTS_ = 0.47; ρ_TFlC/FRAP_ = 0.44) and TPAC (ρ_TPAC/ABTS_ = 0.22; ρ_TPAC/FRAP_ = 0.22), indicating that these molecules probably play a minor role as antioxidants in the analyzed green coffee bean accessions. In contrast, we found a medium correlation between TFdC and ABTS (ρ_TFdC/ABTS_ = 0.58) and a higher correlation with FRAP (ρ_TFdC/FRAP_ = 0.75). However, the best correlation coefficient was obtained when TPC was compared to ABTS (ρ_TPC/ABTS_ = 0.81) and FRAP (ρ_TPC/FRAP_ = 0.84). Overall, our correlation analysis strongly suggests that the antioxidant activity of green bean coffee may depend in part on TFdC, but that compounds other than flavonoids most probably contribute to the total antioxidant activity of the extracts, such as chlorogenic acids and alkaloids, including caffeine [28,59].

**Figure 2:**
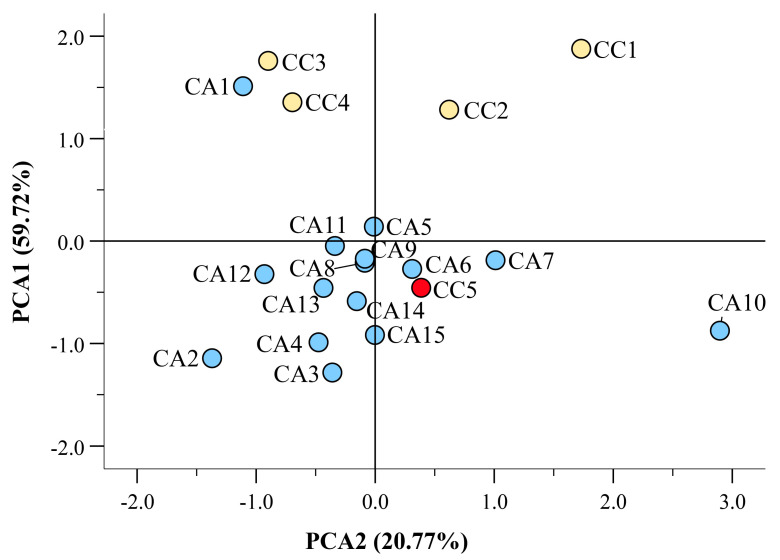
Scatter plot of the principal component (PC1 and PC2) factor scores calculated using spectrophotometric data (TPC, TFdC, TFlC, TPAC, ABTS, and FRAP) on the *C. arabica* (CC) and *C. canephora* (CA) accessions from different geographical origin. The caracol variety (CC5) is evidenced in red. Supplementary Figure S1 shows the partitioning of the different spectrophotometric assays based on PC1 and PC2 factor scores.

Using data obtained from spectrophotometric analysis, a principal component analysis (PCA) was calculated using varimax rotation. The PCA explained 50.42% and 25.32% variation by PC1 and PC2, respectively (Figure 2). The sole use of spectrophotometric data allowed the discrimination of four CC species (CC1, CC2, CC3, and CC4) from the CAs, although CA1 failed to separate from the CC group due to its high TFdC value (see also Table 2). In addition, although the caracol variety (CC5, marked in red) diverged from the CC group, it showed a closer fitting with varieties CA4, CA5, CA6, and CA7, which all originate from South America.

### 3.2. Phenolic Compounds, Xanthine Derivatives, and Melatonin Contents Discriminate C. arabica from C. canephora Accessions

Green coffee is well known for its content of chlorogenic acids [60]. These compounds, derived from the shikimate pathway, are the product of esterification of caffeic acid with quinic acid, and they are primarily responsible for the antioxidant property of green coffee [61]. In our previous work, we demonstrated that chlorogenic acids greatly differ among green coffee species and cultivars, and how the ratio of specific chlorogenic acids can be used to gain important information on the origin of samples [28]. 

**Figure 3:**
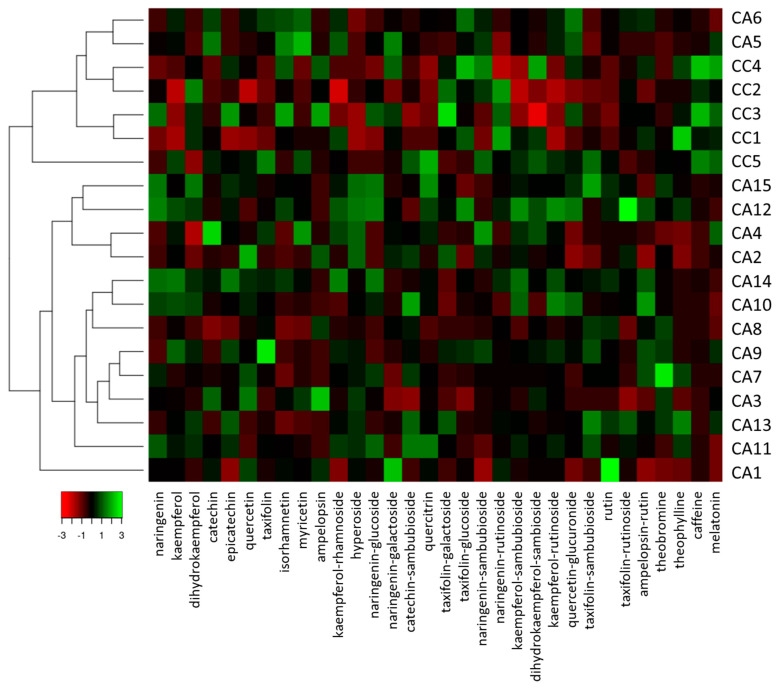
Heatmap coupled to cluster analysis showing the compound variation among the *C. arabica* (CC) and *C. canephora* (CA) accessions of different geographical origin based on data reported in Supplementary Table S1. The dendrogram was generated using the average linkage and Pearson distance methods; the different colors refer to higher (green) or lower (red) relative amounts of molecules among the analyzed accessions.

Although chlorogenic acids are the main contributors to the polyphenols of green bean coffee and play an important role in the chemical discrimination at the geographical level [28], other bioactive molecules, including flavonoids, are potent phytochemical markers of plant species [62]. In this context, the presence of polyphenols in green coffee bean other than chlorogenic acids has been underestimated, as evidenced by the poor literature related to their phytochemical characterization [63]. Therefore, in order to extend the phytochemical profile of the analyzed green coffee beans, we analyzed the different accessions by evaluating coffee compounds other than chlorogenic acids. Thirty-two different compounds were identified. Four contained N in the chemical scaffold, while the remaining 28 were flavonoids. Quantitative data are reported in Supplementary Table S1, and the graphical representation of the quantitative variations of the several identified compounds is shown in Figure 3, which was obtained using a heatmap visualization and cluster analysis. In general, two main clusters were obtained: the first cluster gathering all CC accessions along with CA5 and CA6 from Peru, and the second cluster gathering all the remaining CA accessions (Figure 3).

Among the N-containing compounds, we identified the xanthine derivatives caffeine, theobromine, and theophylline. Melatonin was also detected in the analyzed samples. This compound does not derive from xanthine and is produced through serial modifications starting from the amino acid tryptophan [64]. Although the theobromine content was unable to discriminate CC and CA accessions, statistical differences (*p* < 0.05) were observed for the other N-containing compounds, with CC accessions discriminated from CA accessions on the basis of the highest contents of caffeine (17.23 ± 8.60 mg/g), theophylline (0.60 ± 0.04 mg/g), and melatonin (7.71 ± 5.62 μg/g). In particular, CC2 and CC3 were identified as the variety with the highest amount of caffeine, with a content of almost 22 mg/g, while CC3 and CC4 were the richest in melatonin, accounting for about 9 μg/g on a FW basis. These data are in agreement with the literature data, confirming the higher content of caffeine in the *C. canephora* accessions [65,66]. Moreover, although weaker than chlorogenic acid [67], caffeine was found to possess a significant antioxidant capacity [68], which agrees with our antioxidant data. Interestingly, CA4, CA5, CA9, and CA13 (from South and Central America) also had melatonin contents almost comparable to the CC accessions. These results are in agreement with the literature data [69]. Concerning theophylline, CA13 possessed the highest amounts among all CA accessions, and comparable or higher contents with respect to CC samples (see Supplementary Table S1). Even for theophylline, our results are in line with the literature data [70].

Within the 28 compounds belonging to the flavonoid family, 11 were flavonols, along with nine flavanonols, five flavanones, and three flavan-3-ols (Figure 3). Among flavonols, four different aglycones (kaempferol, quercetin, myricetin, and isorhamnetin) were detected, along with their glycosides. With the exception of isorhamnetin, all detected flavonols did not have *O*-methylated substituents at the hydroxyl group of the B ring. Among the flavanols, dihydrokaempferol, taxifolin, and ampelopsin were detected. These aglycones were in most cases conjugated with rutin and to sugar units such as sambubioside, while only one was found to be conjugated to glucose (taxifolin-glucoside) and galactose (taxifolin-galactoside). Regarding flavanones, only naringenin and its conjugates were detected among the analyzed samples. Lasty, catechin, epicatechin, and catechin-sambubioside were the flavan-3-ols identified in the coffee accession.

We found that CA accessions had kaempferol, quercetin, kaempferol-rhamnoside, hyperoside, kaempferol-sambubioside, kaempferol-rutinoside, and rutin contents 1.5-fold to 2.3-fold higher than CC samples (see Supplementary Table S1). On the other hand, CC samples were characterized by higher levels of taxifolin-galactoside (35.86 ± 3.35 μg/100 g), taxifolin-glucoside (21.39 ± 0.81 μg/100 g), and naringenin-rutinoside (13.47 ± 0.19 μg/100 g). All other compounds, although differing within the CC and CA group, were found not to be statistically different between the two species (see Supplementary Table S1).

Because chlorogenic acids significantly contribute to the total polyphenol content of green coffee, other polyphenolic compounds have been poorly investigated [71,72]. Here, we provide the flavonoid profile of green bean coffee, and we describe its use in order to discriminate gene coffee geographic origin, variety, and species. Our data are in agreement with Mustafa and coworkers [63], who developed a method for the simultaneous determination of quercetin and glycosidic derivatives in green coffee beans. In addition, the contents of kaempferol and myricetin, along with their respective analogues resulting from the complexation with sugar moieties, evidenced in our analyses are in agreement with recent literature data [73,74].

**Figure 4:**
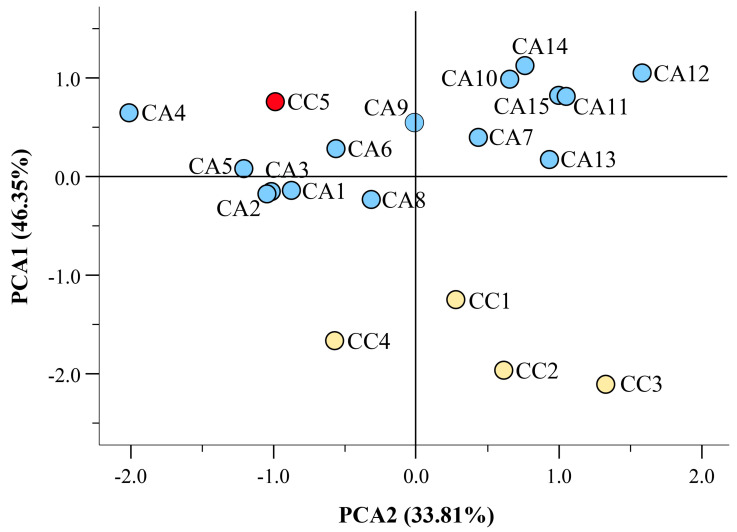
Scatter plot of the principal component (PC1 and PC2) factor scores calculated using quantitative data from the *C. arabica* (CC) and *C. canephora* (CA) accessions of different geographical origin (see Supplementary Table S1). The caracol variety (CC5) is evidenced in red. Supplementary Figure S2 shows the portioning of the various identified compounds based on PC1 and PC2 factor scores.

Using the data reported in Supplementary Table S1, a PCA was calculated with varimax rotation. The PCA explained 36.32% and 33.81% variation by PC1 and PC2, respectively (Figure 4). The chemical quantification of the identified compounds allowed the discrimination of four CC (CC1, CC2, CC3, and CC4) from all other CA accessions. In this case, the caracol variety (CC5, marked in red) also diverged from the CC group, with a closer fitting with varieties CA4, CA5, and CA6, which all originate from South America.

### 3.3. The Fatty Acid Composition and Content Discriminate C. arabica and C. canephora Accessions of Different Geographical Origin

To further discriminate the different accessions of the two species, we analyzed the fatty acid profile, because it has been successfully used to discriminate plants species and their origin [35,75,76,77]. With regard to coffee, fatty acids have been successfully used to discriminate the geographic origin of different varieties of *C. arabica* [78,79,80] and *C. canephora* [81]. Here, GC-FID and GC–MS analyses were performed on trans-esterified fatty acid samples in order to find additional indicators for green coffee discrimination. The main identified fatty acids were linoleic acid (C18:2) and palmitic acid (C16:0), each accounting for about 39% (*w*/*w*) of the total fatty acids, followed by elaidic acid (*trans* C18:1) and stearic acid (C18:0), which amounted to 8.75% (*w*/*w*) and 7.70% (*w*/*w*), respectively. Arachidic acid (C20:0) was also present in good percentages, reaching about 4% (*w*/*w*) of the total fatty acids, while oleic acid (*cis* C18:1), behemic acid (C22:0), and myristic acid (C14:0) contributed less than 2% (*w*/*w*) together. Our results agree with previous published data on the fatty acid composition of coffee accessions [78,79,81]. In general, CC green coffee beans were characterized by significantly (*p* < 0.05) lower levels of C18:2 and *cis* C18:1 than CA samples. At the same time, CC beans had the highest amounts of *trans* C18:1 and C14:0.

**Figure 5:**
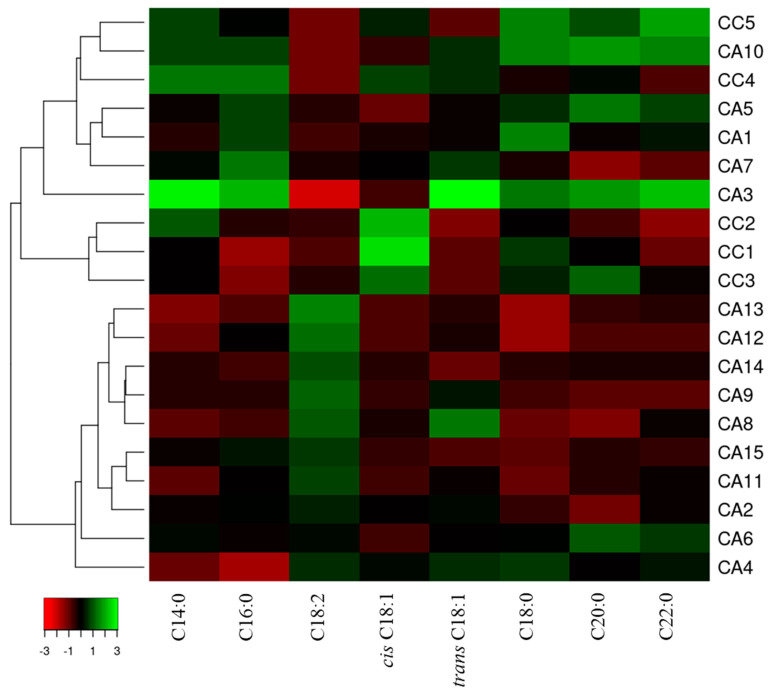
Heatmap coupled to cluster analysis showing the variation in fatty acid composition among the *C. arabica* (CC) and *C. canpehora* (CA) accessions of different geographical origin based on data reported in Supplementary Table S2. The dendrogram was generated using the average linkage and Pearson distance methods; the different colors refer to higher (green) or lower (red) relative amounts of the respective fatty acid among the analyzed samples.

Quantitative data obtained from GC analyses are reported in Supplementary Table S2, while Figure 5 shows the visualization of the percentage content using a heatmap combined with cluster analysis. Two main clusters are evidenced; the first gathers most of the CA accessions, whereas the second is subdivided in subclusters, one of which collects some CC accessions (CC1, CC2, and CC3) (Figure 5).

**Figure 6:**
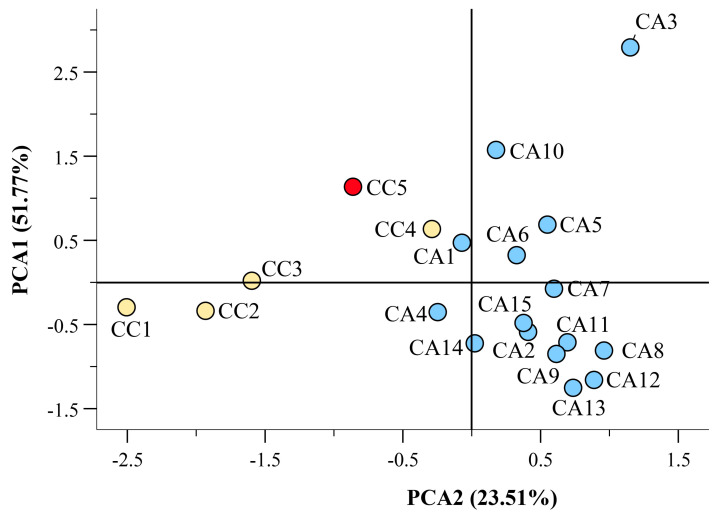
Scatter plot of the principal component (PC1 and PC2) factor scores calculated using quantitative and qualitative fatty acid composition of *C. arabica* (CC) and *C. canephora* (CA) accessions of different geographical origin (Supplementary Table S2). The caracol variety (CC5) is evidenced in red. The figure shows the partitioning of the different accessions based on PC1 and PC2 factor score. The partitioning of the different fatty acids based on PC1 and PC2 factor score is reported in Supplementary Figure S3.

Using the quantitative data reported in Supplementary Table S2, a PCA was calculated with varimax rotation. The PCA explained 51.77% and 23.51% variation on PC1 and PC2, respectively (Figure 6). Quantitative fatty acid data allowed the discrimination of three CC (CC1, CC2, CC3, and CC5, marked in red) from CA accessions, whereas CC4 diverged from the CC group, being closer to CA accessions (Figure 6).

### 3.4. Hight Throughput Analysis Allows the Metabolic and Geographical Discrimination of C. arabica and C. canephora

**Figure 7:**
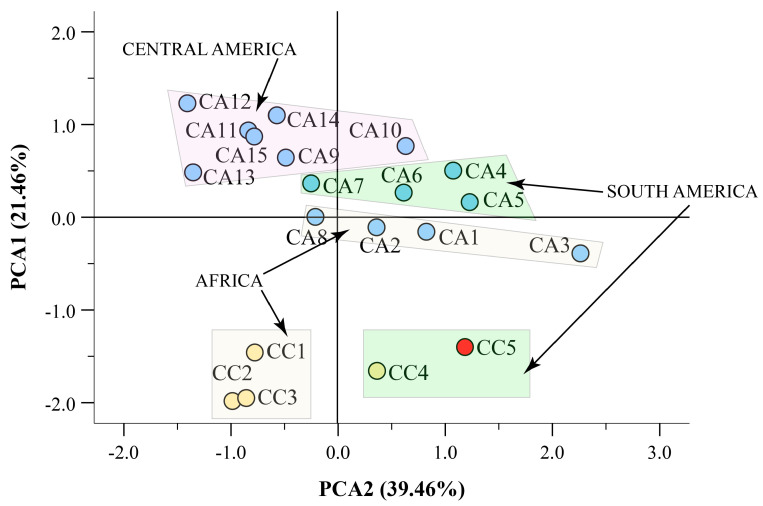
Scatter plot of the principal component (PC1 and PC2) factor scores calculated using high-throughput data including TPC, TFdC, TFlC, TPAC, ABTS, FRAP, phenolics, xanthine derivatives, melatonin, and fatty acids of *C. arabica* (CC) and *C. canephora* (CA) accessions from different geographical origin. The high-throughput analysis from all metabolomic and antioxidant data clearly separated CC accessions from CA accessions. The caracol variety (CC5) is evidenced in red. Moreover, CA and CC accessions were further separated according to their geographical origin. The partitioning of the different parameters based on PC1 and PC2 factor score are shown in Supplementary Figure S4. Data used for this PCA analysis are from Table 2, Figure 1, and Supplementary Tables S1 and S2.

The combined use of all data from spectrophotometric quantitative data (TPC, TFdC, TFlC, and TPAC), antioxidant activity (ABTS and FRAP), phenolics, xanthine derivatives, and melatonin, along with fatty acid composition, allowed us to perform a high-throughput PCA (Figure 7). The PCA explained 39.46% and 21.41% variation by PC1 and PC2, respectively. The use of the high-throughput data allowed the discrimination of the five CC accessions from all CA accessions, with a clear distinction between the African (CC1, CC2, and CC3) and the South American (CC4 and CC5, marked in red) *C. canephora*. Moreover, a further discrimination was observed for all remaining *C. arabica*, with a clear distinction of the South American, Central American, and African accessions (Figure 7).

### 3.5. DNA Fingerprinting Using trnL-trnF and 5S-rRNA-NTS Regions Allows the Molecular Discrimination of Species and Accessions

Plant chemometrics used in our study allowed us to discriminate the two coffee species and to evidence a geographical partitioning. This requires time, and the technology transfer to industrial applications needs fast and cheap technologies. Moreover, the phenotypic plasticity of plants responding to environmental, biotic, and abiotic stresses affects gene expression and product (molecules) formation that may render the metabolomic approach less stable. In this context, DNA analysis is relatively fast, and the presence of molecules with higher stability makes discrimination easier. The genetic method requires genotype rather than phenotype; therefore, DNA-based discrimination has become a widely employed technique for a reliable partitioning of plants [33].

In order to provide a molecular fingerprinting of the coffee accessions, two different primers were used to amplify *trnL-trnF* and *5S-rRNA-NTS* regions of the coffee genome. 

**Figure 8:**
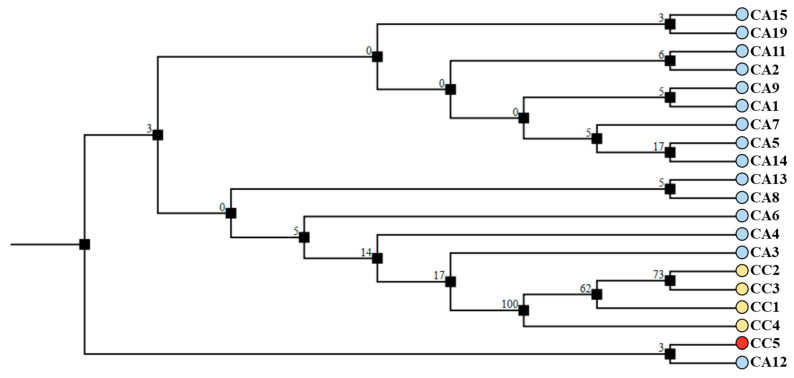
Cladogram of *trnL-trnF* sequences performed with CLC software using the neighbor joining (NJ) method on the *C. canephora* (CC) and *C. arabica* (CA) accessions of different geographical origin. The caracol variety (CC5) is evidenced in red. The percentage of replicate trees in which the associated taxa clustered together in the bootstrap test (500 replicates) are shown next to the branches. The tree is drawn to scale, with branch lengths in the same units as those of the evolutionary distances used to infer the phylogenetic tree.

Regarding *trnL-trnF*, the size of the amplified fragment was 984 bp for all analyzed accessions. Data from the sequence analyses (deposited in NCBI GenBank with Accession Numbers from OQ779490 to OQ779509) were aligned using CLC software (Qiagen, Hilden, Germany) (see Supplementary Figure S5). In general, the consensus sequence showed highly conserved regions (99.19%) in the amplified site, with 0.71% of them being singletons. The *trnL-trnF* fragments were compared by BLAST alignment to other sequences deposited in GeneBank, and the analysis provided a match almost identical to *C. canephora* for CC accessions or to *C. arabica* for CA accessions. Sequences were further analyzed using the neighbor joining (NJ) method to infer the phylogenetic relationship among the green coffee accessions. The phylogenetic tree obtained shows the presence of two main clades: one comprising the caracol variety (CC5, marked in red) and one comprising the *C. arabica* from Honduras (CA12), which were joined by a low bootstrap score, while another clade gathered all other accessions. In particular, this second clade isolated the remaining *C. canephora* accessions (CC1, CC2, CC3, and CC4) that formed an independent clade, whose robustness was supported by high bootstrap scores (Figure 8).

*trnL-trnF* encodes for a region composed of the *trnL* gene, a group of introns, and the *trnL-trnF* intergenic spacer. Moreover, this region is also characterized by the presence of both conserved elements (which are essential for a correct splicing) and less constrained regions of variable size [82]. Although the number of available markers is gradually increasing, the *trnL-trnF* region still represents one of the most frequently used molecular markers in plants. Indeed, since its introduction into molecular systematics [83], this region has been considered appropriate for investigations at various taxonomic levels. However, to the best of the knowledge of the authors, the intron has never been used to elucidate relationships between species of the genus *Coffea*, although the *trnL-trnF* spacer was successfully used to analyze the evolution of the region in three gymnosperm families [30].

**Figure 9:**
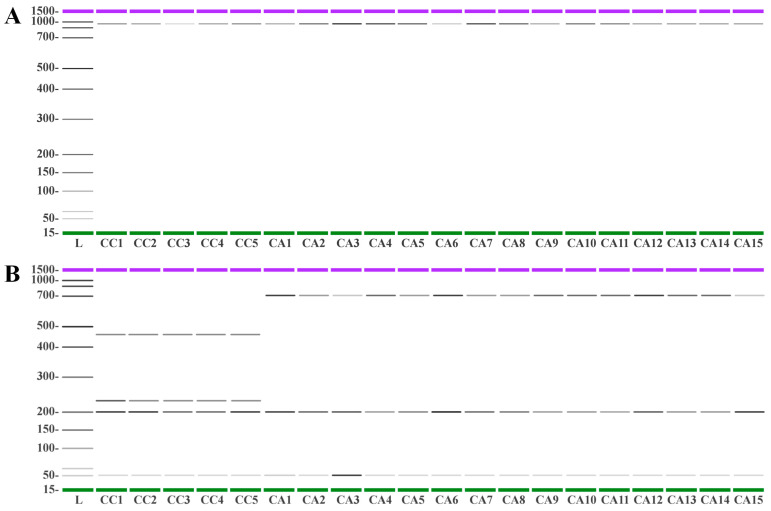
Capillary gel electrophoresis gel simulation analysis of *trnL-trnF* DNA from *C. canephora* (CC) and *C. arabica* (CA) accessions used in this study before (Panel **A**) and after (Panel **B**) digestion using *AluI* as restriction enzyme. The PCR products were separated using the Agilent 2100 Bioanalyzer, and the electropherograms are reported in Supplementary File S1.

In order to better characterize the coffee accessions, a PCR-RFLP method was applied using *AluI* as a restriction enzyme to selectively cleave the resulting amplicons. Before enzymatic cleavage, all accessions were found to possess a common *trnL-trnF* band of 984 bp (Figure 9A). Cleavage by *AluI* produced in all CC accessions four fragments of 489, 253, 207, and 50 bp, whereas the DNA cleavage of all CA accessions produced fragments of 742, 207, and 50 bp (Figure 9B). Interestingly, the cleavage of the caracol variety (CC5) showed the same bp fragmentation of all other *C. canephora* accessions, confirming that this mutation belongs to the *C. canephora* species. Therefore, *trnL-trnF*-RFLP analysis using *AluI* has proven to be an efficient molecular strategy to selectively differentiate between *C. canephora* and the *C. arabica* species.

**Figure 10:**
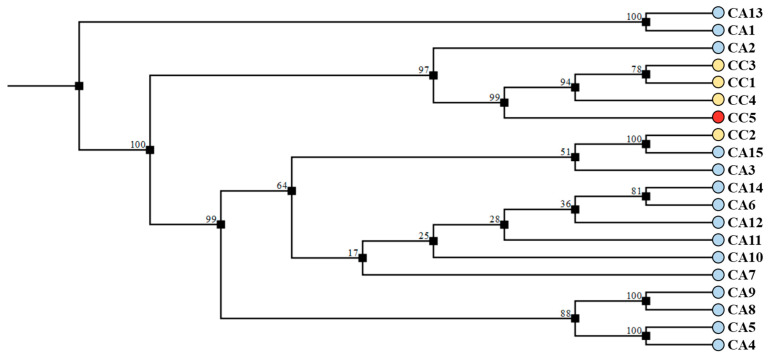
Cladogram of *5S-rRNA-NTS* sequences performed with CLC software using the neighbor joining (NJ) method on *C. canephora* (CC) and *C. arabica* (CA) accessions used in this study. The caracol variety (CC5) is evidenced in red. The percentage of replicate trees in which the associated taxa clustered together in the bootstrap test (500 replicates) are shown next to the branches. The tree is drawn to scale, with branch lengths in the same units as those of the evolutionary distances used to infer the phylogenetic tree.

Regarding the amplification of *5S-rRNA-NTS* region, almost all accessions produced a fragment of about 500 bp, with the exception of CA1 and CA13, for which larger fragments (~850 bps) were obtained. Sequences (deposited in NCBI GenBank with accession numbers OQ791415 to OQ791432) were aligned using CLC software (Qiagen, Hilden, Germany) (see Supplementary Figure S6). In general, the alignment showed moderate (88.07%) conservation of the amplified site. The *5S-rRNA-NTS* fragments were compared by BLAST alignment to other sequences deposited in GeneBank, and the analysis provided a match almost identical to *C. canephora* for CC samples or to *C. arabica* for CA samples (data not shown). The sequences were further analyzed using the neighbor joining (NJ) method to infer phylogenetic relationships among the green coffee accessions. The phylogenetic tree shows the presence of five clades; three of them gathered most of the CA accessions, whereas one separated CC1, CC2, CC3, and CC5 with a high bootstrap score, and another isolated CC2 and CA15 with a high bootstrap score (Figure 10).

The *5S-rRNA-NTS* region is present in all plant ribosomes, with the sole exception for mitochondria of a few species [84]. It is composed of tandem repeats of alternative arrays of sequences coding *5S-rRNA* and non-transcribed spacers (NTSs) located separately from the 18S-26S rRNA gene clusters [45]. Over the years, relevant results have been obtained using RFLP analysis in the *5S-rRNA-NTS* region [33,45,85,86,87]. Consequently, to obtain a DNA molecular fingerprinting of the different coffee accessions, the PCR-RFLP method was applied combining *MseI* and *Xhol* as restriction enzymes to selectively cleave the resulting amplicons.

**Figure 11:**
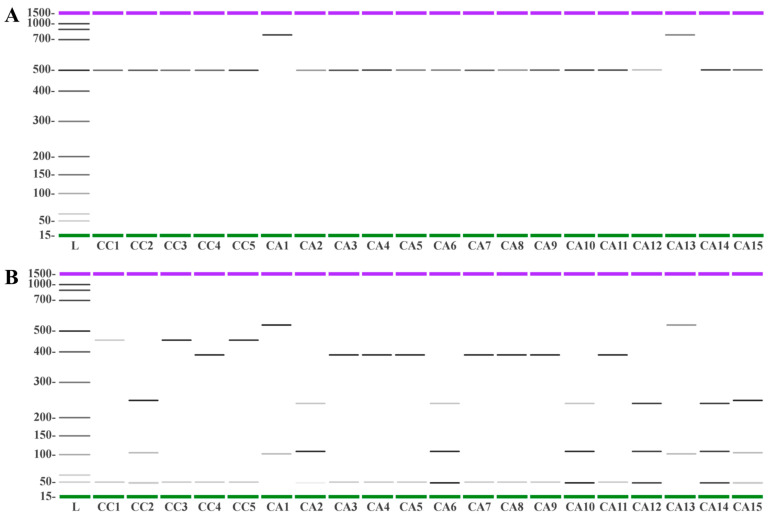
Capillary gel electrophoresis analysis of *5S-rRNA-NTS* from *C. canephora* (CC) and *C. arabica* (CA) accessions before (Panel **A**) and after (Panel **B**) digestion using *MseI* and *XholI* as restriction enzymes. The PCR products were separated using the Agilent 2100 Bioanalyzer, and the electropherograms are reported in Supplementary File S2.

As expected, gel electrophoresis of the *5S-rRNA-NTS* region produced a common band of about 500 bp in all accessions, with the exception of CA1 and CA13, for which a fragment of about 850 bp was observed (Figure 11A). The reason why CA1 and CA13 possess 5S-rRNA fragment sizes was not further investigated. However, it is likely that larger 5S rRNA fragments might be due to genetic or epigenetic differences in their respective 5S-rRNA gene clusters [88]. The combination of the restriction enzymes *MseI* and *XhoI* produced selective cleavages on the different accessions (Figure 11B). Specifically, *MseI* cleavage produced fragments of 445 bp and 50 bp in CC1, CC3, and CC5, whereas *XhoI* exclusively cleaved CA1 and CA13 *5S-rRNA-NTS*, producing fragments of 600 and 114 bp. All other accessions were cut at least three times, producing different fragment patterns. A first group consisted of CC2 and CA15 with fragments of 273 and 120 bp. A second group gathered CA2, CA6, CA10, CA12, and CA14, with a cleavage generating fragments of 257, 140, and 50 bp (the latter band consisting of two fragments of 57 and 54 bp; see electropherograms of Supplementary File S2). A third group was made by CC4 along with CA3, CA4, CA5, CA7, CA8, CA9, and CA11, characterized by the production of a fragment of 396 bp and one of 50 bp (in this case resulting from the overlapping of two fragments of 57 and 54 bp) (Figure 11B).

## 4. Conclusions

The results of this work indicate that a metabolomic approach can be useful for the discrimination of coffee species and accessions from different geographical origin. However, when excluding chlorogenic acids from the analysis, the best discrimination was obtained only using a high-throughput data analysis, by combining the quantitative data from spectrophotometric analyses and the qualitative and quantitative data from LC–MS and GC–MS analyses. In general, *C. canephora* (Robusta) accessions were confirmed to possess a higher antioxidant activity due to the high content of total phenolic compounds and caffeine when compared to *C. arabica* (Arabica). These data confirm our previous analysis performed on the chlorogenic acid distribution on the same green coffee accessions [28] and provide new data on the flavonoid and fatty acid content that contributed to the high-throughput discrimination among green coffee species and accessions. The phytochemical characterization of green coffee flavonoids also contributes to a better understanding of their health potential [52].

Our PCR-RFLP analysis through the combined action of different restriction enzymes allowed the identification of recognition markers for most of accession. By using *AluI* on *trnL-trnF,* a clear discrimination was found between *C. canephora* and *C. arabica*, although no difference was found according to the geographical origin. On the other hand, *MseI* and *XholI* cleavage of *5S-rRNA-NTS* produced a pattern of discrimination that can be summarized as follows: (i) a fragment of 445 bp, characteristic for the *C. canephora* accessions (CC1, CC3, and CC5); (ii) the combined presence of 600 bp and 114 bp, typical of CA1 (Kenya, Arabica low grade) and CA13 (Honduras, Catuahi); (iii) fragments of 257 and 140 bp, exclusive to CA2 (Kenya, Arabica AK3), CA6 (Peru, Jacamar), CA10 (Guatemala, HB ep), CA12 (Honduras, HG ep Margay), and CA14 (Honduras, HG ep Margay) accessions; (iv) bands of 273 bp and 120 bp, distinctive for CC2 (Uganda, Jolly Quartz) and CA15 (Honduras, HG ep Margay); (v) a 396 bp fragment, characterizing all remaining accessions.

In conclusion, using a combination of high-throughput metabolomics with phenolic compounds, fatty acids, xanthine derivatives, and melatonin, along with antioxidant power and DNA fingerprinting, we were able to discriminate the two coffee species and partition the individual accessions of the species according to their geographical origin.

## Figures and Tables

**Table 1:** Summary of code names of *C. canephora* (CC) and *C. arabica* (CA) accessions used for the study and their geographical origin.

Code	Geographical Origin		Species	Cultivar	Commercial Name	Accession No
**CC1**	Uganda	Africa	*C. canephora*	Jolly Quartz	Robusta	28337/1
**CC2**	Uganda	Africa	*C. canephora*	Jolly Quartz	Robusta	28336/1
**CC3**	Vietnam	Southeast Asia	*C. canephora*	Vietnam clean	Robusta	FV2583
**CC4**	Vietnam	Southeast Asia	*C. canephora*	Vietnam unwashed	Robusta	FV2583
**CC5**	Brazil	South America	*C. canephora*	Moka Fine C	Caracol	FV1147
**CA1**	Kenya	Africa	*C. arabica*	Arabica low grade	Arabica	6841A
**CA2**	Kenya	Africa	*C. arabica*	Arabica AK3	Arabica	AK-3
**CA3**	Kenya	Africa	*C. arabica*	Arabica AK2	Arabica	AK-2
**CA4**	Brazil	South America	*C. arabica*	Arabica Natural Terraforte	Arabica	401|CA0839
**CA5**	Peru	South America	*C. arabica*	Tinamous	Arabica	6890
**CA6**	Peru	South America	*C. arabica*	Jacamar	Arabica	6874
**CA7**	Colombia	South America	*C. arabica*	Pacorini Silocaf	Arabica	240229/1
**CA8**	Ethiopia	Africa	*C. arabica*	Sidamo Grade 2	Arabica	1168
**CA9**	Mexico	Central America	*C. arabica*	PW ep	Arabica	1190
**CA10**	Guatemala	Central America	*C. arabica*	HB ep	Arabica	1191
**CA11**	Honduras	Central America	*C. arabica*	HG ep “Margay”	Arabica	1195
**CA12**	Honduras	Central America	*C. arabica*	HG ep “Margay”	Arabica	1195
**CA13**	Honduras	Central America	*C. arabica*	Catuahi, Caturra, Icatu	Arabica	801
**CA14**	Honduras	Central America	*C. arabica*	HG ep “Margay”	Arabica	1145
**CA15**	Honduras	Central America	*C. arabica*	HG ep “Margay”	Arabica	1195

**Table 2:** Content of flavonoids, flavonols, and flava-3-ols in *C. arabica* and *C. canpehora* accessions from different geographical origin. Values are represented as the mean ± SD of three different biological replicates. The first column identifies the green bean coffee accessions (*C. canephora*: CC; *C. arabica*: CA) as indicated in Table 1. For each column, different lowercase letters indicate significant differences (*p* ≤ 0.05, as measured by Tukey’s multiple range test).

Code	TFdC (mg RE·100 g^−1^)	TFlC (mg QE·100 g^−1^)	TPAC (mg PACE·100 g^−1^)
**CC1**	125.59 ± 10.09 ^a^	68.65 ± 4.25 ^bc^	2.72 ± 0.22 ^a^
**CC2**	105.23 ± 12.99 ^b^	68.66 ± 9.38 ^bc^	2.33 ± 0.33 ^ab^
**CC3**	121.87 ± 8.54 ^ab^	70.69 ± 4.71 ^ab^	2.06 ± 0.12 ^cd^
**CC4**	83.26 ± 8.85 ^c^	81.12 ± 5.57 ^a^	2.05 ± 0.16 ^cd^
**CC5**	74.92 ± 7.45 ^hi^	42.58 ± 4.82 ^i^	1.91 ± 0.36 ^cd^
**CA1**	136.11 ± 18.43 ^a^	75.46 ± 5.19 ^ab^	1.92 ± 0.28 ^cd^
**CA2**	33.58 ± 3.77 ^l^	49.13 ± 5.84 ^fg^	1.61 ± 0.15 ^e^
**CA3**	38.83 ± 6.89 ^l^	47.46 ± 4.81 ^fg^	1.73 ± 0.35 ^de^
**CA4**	42.87 ± 3.59 ^il^	52.95 ± 1.44 ^ef^	1.86 ± 0.29 ^cd^
**CA5**	46.79 ± 4.82 ^hi^	64.79 ± 1.34 ^cd^	2.07 ± 0.28 ^cd^
**CA6**	39.89 ± 5.55 ^i^	56.29 ± 7.14 ^de^	2.15 ± 0.31 ^cd^
**CA7**	35.65 ± 1.87 ^i^	62.57 ± 8.29 ^cd^	2.33 ± 0.37 ^ab^
**CA8**	59.81 ± 1.02 ^ef^	53.52 ± 9.94 ^ef^	2.05 ± 0.21 ^cd^
**CA9**	31.23 ± 1.05 ^i^	64.76 ± 6.25 ^cd^	2.21 ± 0.29 ^ab^
**CA10**	57.94 ± 5.74 ^gh^	44.23 ± 3.63 ^hi^	2.64 ± 0.15 ^a^
**CA11**	78.01 ± 8.71 ^de^	60.57 ± 4.45 ^cd^	1.86 ± 0.28 ^cd^
**CA12**	39.01 ± 4.59 ^cd^	57.61 ± 3.72 ^de^	1.75 ± 0.22 ^de^
**CA13**	62.27 ± 6.47 ^l^	52.29 ± 5.34 ^ef^	1.91 ± 0.32 ^cd^
**CA14**	73.84 ± 8.22 ^cd^	46.42 ± 7.48 ^gh^	1.91 ± 0.23 ^cd^
**CA15**	79.79 ± 6.01 ^cd^	44.59 ± 7.11 ^hi^	1.73 ± 0.18 ^d^

TFdC: total flavonoid content; TFlC: total flavonol content; TPAC: total proanthocyanidin content; RE: rutin equivalent; QE: quercetin equivalent; PACE: proanthocyanidin A2 equivalent.

## Data Availability

Data are available as Supplementary Materials and on request.

## Supplementary Materials

The following supporting information can be downloaded at: https://www.mdpi.com/article/10.3390/antiox12051135/s1: Figure S1. Partitioning based on the PC1 and PC2 factor scores of CA and CC accessions according to spectrophotometric determination of bioactive compounds and antioxidant activity. See text for abbreviations; Figure S2. Partitioning based on the PC1 and PC2 factor scores of CA and CC accessions according to bioactive compounds by HPLC-DAD–ESI-MS/MS analysis; Figure S3. Partitioning based on the PC1 and PC2 factor scores of CA and CC accessions according to FAMEs by GC–MS an GC-FID analysis. See text for abbreviations; Figure S4. Partitioning based on the PC1 and PC2 factor scores of CA and CC accessions according to UV/Vis, HPLC, and GC data; Figure S5. Sequence alignment of trnL-trnF region using CLC software (Qiagen, Hilden, Germany) in CA and CC accessions. See Table 1 for coding of accessions; Figure S6. Sequence alignment of 5S-rRNA-NTS region using CLC software (Qiagen, Hilden, Germany) in CA and CC accessions. See Table 1 for the coding of accessions; Table S1. Qualitative and quantitative chemical analysis of compounds extracted from CA and CC accessions from different geographical origin and identified by HPLC–ESI-MS/MS analysis; Table S2. Qualitative and quantitative composition of fatty acids present in CA and CC accessions from different geographical origin and identified and quantified by GC–MS and GC-FID. Lowercase letters indicate statistical differences in the content as described by one-way ANOVA followed by Tuckey’s test. See text for abbreviations; File S1. PCR products of trnL-trnF region separated by the Agilent 2100 Bioanalyzer, reporting the electropherograms of CA and CC accessions before and after digestion with *AluI*; File S2. PCR products of 5S-rRNA-NTS region separated by the Agilent 2100 Bioanalyzer and reporting the electropherograms of CA and CC accessions before and after digestion with *MseI* and *XholI*.
