# Supplementary material for: Discrimination of Green Coffee (Coffea arabica and Coffea canephora) of Different Geographical Origin Based on Antioxidant Activity, High-Throughput Metabolomics, and DNA RFLP Fingerprinting
Source: Antioxidants (Basel). 2023 May 21;12(5):1135. doi: 10.3390/antiox12051135 (PMC10215230; doi:10.3390/antiox12051135)
Supplement: Supplementary file 1 [file antioxidants-12-01135-s001.zip › Supplementary Data File S1.pdf]

Supplementary Data S1: PCR products of *trnL-trnF* region separated by the Agilent 2100 Bioanalyzer and reporting the electropherograms of CA e CC accessions before and after digestion with *AluI*.

Assay Class: DNA 1000
Data Path: C:\...-30\2100 expert_DNA 1000_DE34903611_2023-03-06_TRN_1_10.xad

Created: 3/06/2023 4:21:53 PM
Modified: 3/06/2023 4:21:53 PM

Electrophoresis File Run Summary

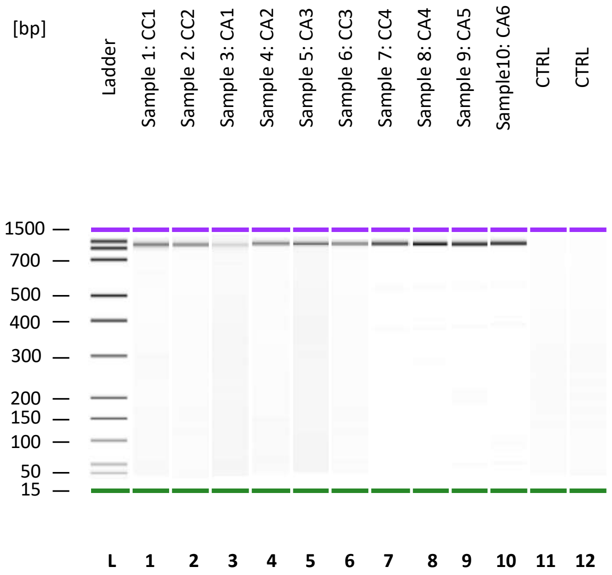

Instrument Information:
Instrument Name: DE34903611
Firmware: C.01.069
Serial #: DE34903611
Type: G2938C

Assay Information:
Assay Origin Path: C:\Program Files\Agilent\2100 bioanalyzer\2100 expert\assays\dsDNA\DNA 1000 Series II.xsy
Assay Class: DNA 1000
Version: 2.3
Assay Comments: DNA Analysis 2 - 1000 bp

© Copyright 2003-2009 Agilent Technologies, Inc.

Chip Information:
Chip Lot #:
Reagent Kit Lot #:
Chip Comments:

Sample #1: CC1

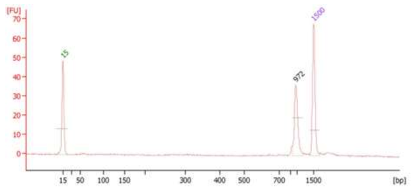

Sample #2: CC2

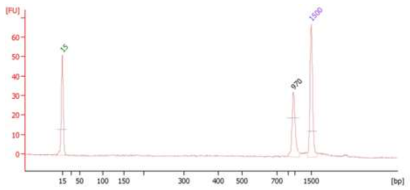

Sample #3: CA1

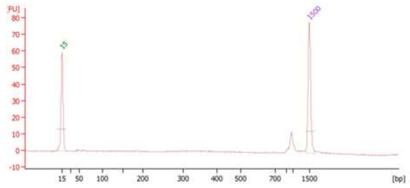

Sample #4: CA2

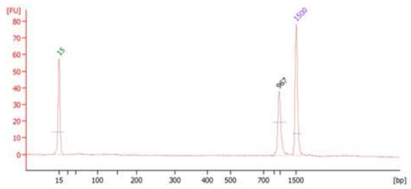

Sample #5: CA3

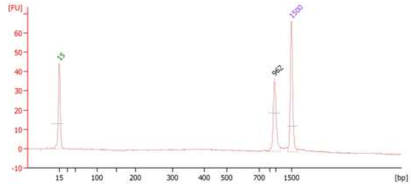

Sample 6: CC3

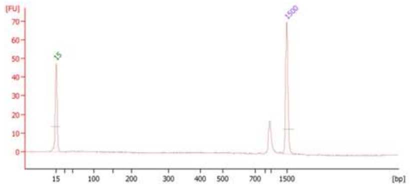

Sample #7: CC4

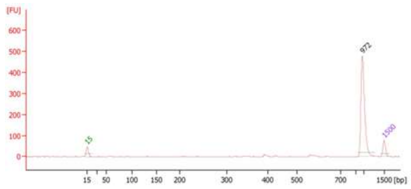

Sample #8: CA4

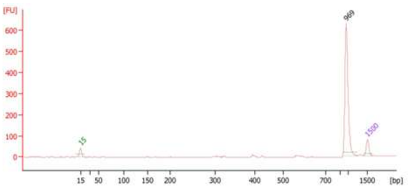

Sample #9: CA5

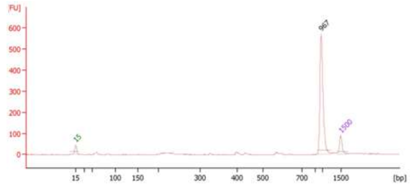

Sample #10: CA6

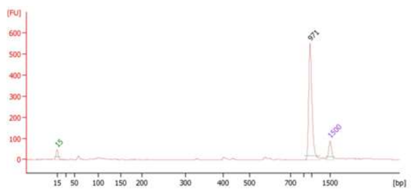

CTRL

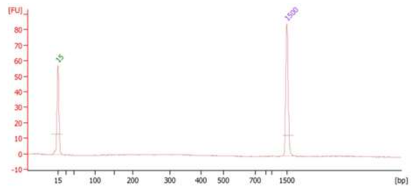

CTRL

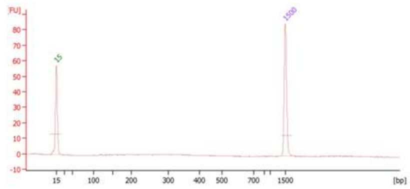

Assay Class: DNA 1000
Data Path: C:\...-30\2100 expert_DNA 1000_DE34903611_2023-03-06_TRN_1_10.xad

Created: 3/06/2023 4:21:53 PM
Modified: 3/06/2023 4:21:53 PM

Electrophoresis File Run Summary

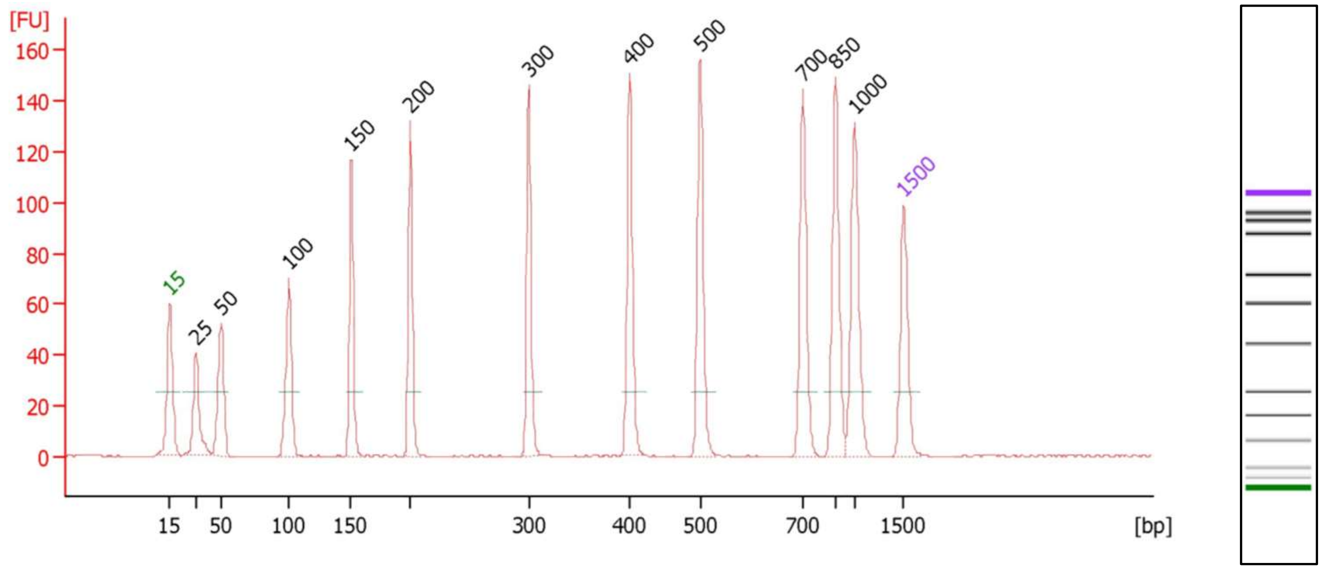

Peak table for Ladder :

Peak		Size [bp]	Conc. [ng/µl]	Molarity [nmol/l]	Observations
1		15	4.20	4.4.2	Lower Marker
2		25	4.00	242.4	Ladder Peak
3		50	4.00	121.2	Ladder Peak
4		100	4.00	60.6	Ladder Peak
5		150	4.00	40.3	Ladder Peak
6		200	4.00	30.3	Ladder Peak
7		300	4.00	20.2	Ladder Peak
8		400	4.00	15.2	Ladder Peak
9		500	4.00	12.1	Ladder Peak
10		700	4.00	8.7	Ladder Peak
11		850	4.00	7.1	Ladder Peak
12		1,000	4.00	6.1	Ladder Peak
13		1,500	2.10	2.1	Upper Marker

© Copyright 2003 - 2009 Agilent Technologies, Inc.

Assay Class: DNA 1000
Data Path: C:\...-30\2100 expert_DNA 1000_DE34903611_2023-03-06_TRN_1_10.xad

Created: 3/06/2023 4:21:53 PM
Modified: 3/06/2023 4:21:53 PM

Electrophoresis File Run Summary

Sample #1: CC1

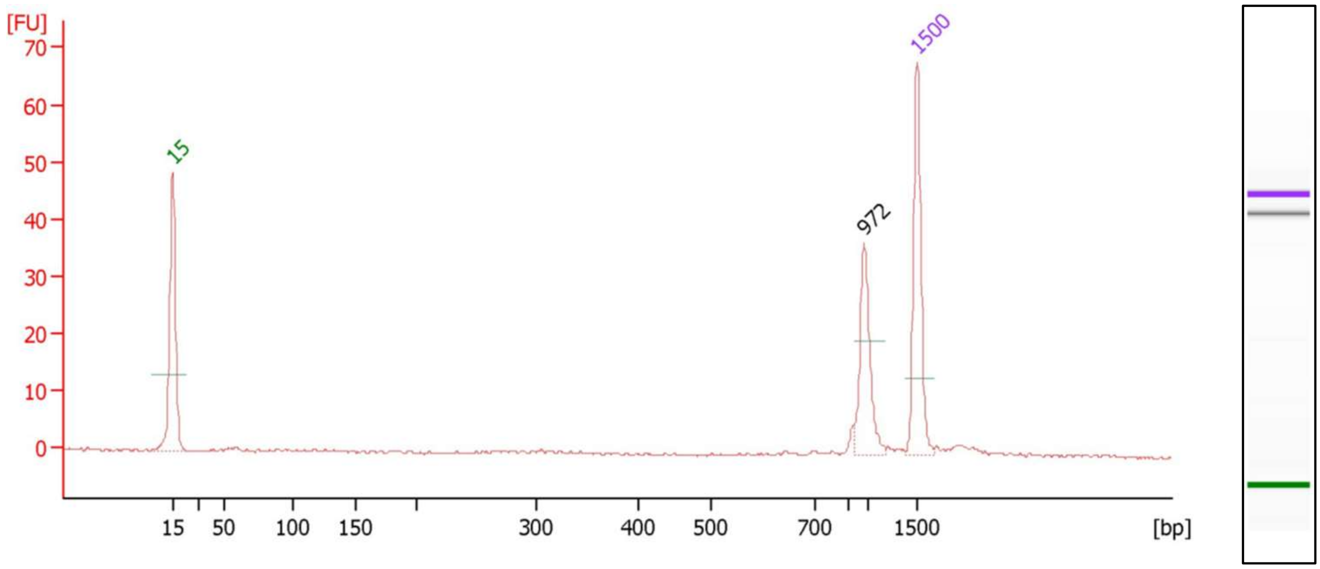

Peak table for Sample #1 :

Peak		Size [bp]	Conc. [ng/µl]	Molarity [nmol/l]	Observations
1		15	4.20	424.4	Lower Marker
2		972	1.63	2.5	Ladder Peak
3		1,500	2.10	2.2	Upper Marker

© Copyright 2003 - 2009 Agilent Technologies, Inc.

Assay Class: DNA 1000
Data Path: C:\...-30\2100 expert_DNA 1000_DE34903611_2023-03-06_TRN_1_10.xad

Created: 3/06/2023 4:21:53 PM
Modified: 3/06/2023 4:21:53 PM

Electrophoresis File Run Summary

Sample #2: CC2

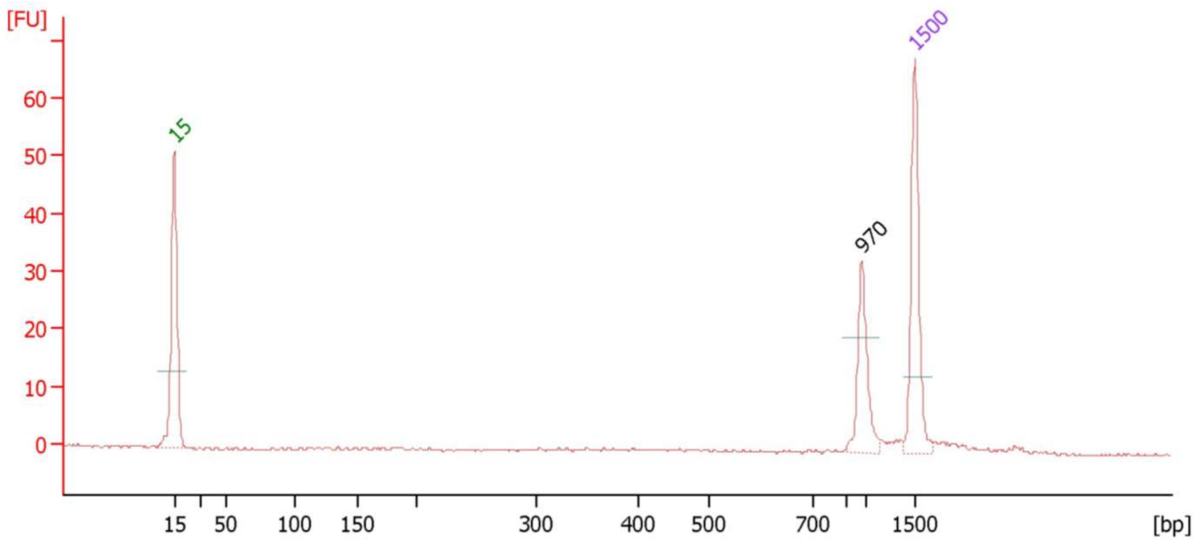

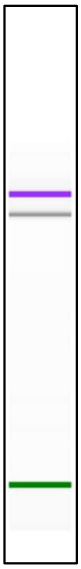

Peak table for Sample #2 :

Peak		Size [bp]	Conc. [ng/µl]	Molarity [nmol/l]	Observations
1		15	4.20	424.4	Lower Marker
2		970	1.41	2.2	Ladder Peak
3		1,500	2.10	2.2	Upper Marker

© Copyright 2003 - 2009 Agilent Technologies, Inc.

Assay Class: DNA 1000
Data Path: C:\...-30\2100 expert_DNA 1000_DE34903611_2023-03-06_TRN_1_10.xad

Created: 3/06/2023 4:21:53 PM
Modified: 3/06/2023 4:21:53 PM

Electrophoresis File Run Summary

Sample #3: CA1

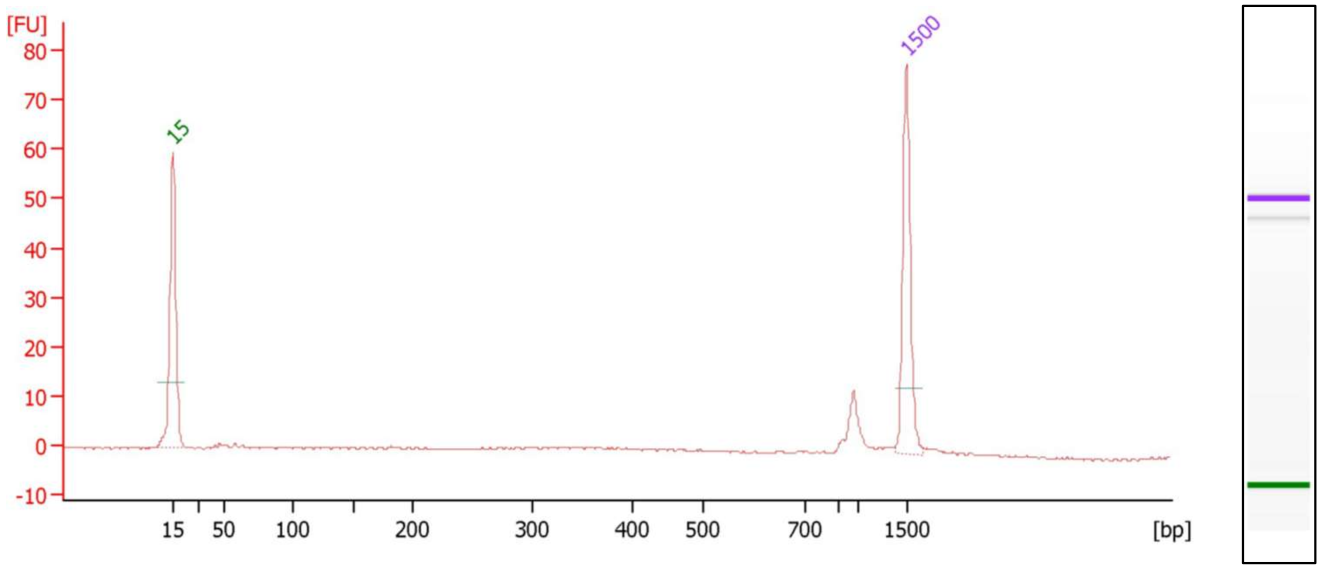

Peak table for Sample #3 :

Peak		Size [bp]	Conc. [ng/µl]	Molarity [nmol/l]	Observations
1	◀	15	4.20	424.4	Lower Marker
2	▶	1,500	2.10	2.1	Upper Marker

 © Copyright 2003 - 2009 Agilent Technologies, Inc.

Assay Class: DNA 1000
Data Path: C:\...-30\2100 expert_DNA 1000_DE34903611_2023-03-06_TRN_1_10.xad
Created: 3/06/2023 4:21:53 PM
Modified: 3/06/2023 4:21:53 PM

Electrophoresis File Run Summary

Sample #4: CA2

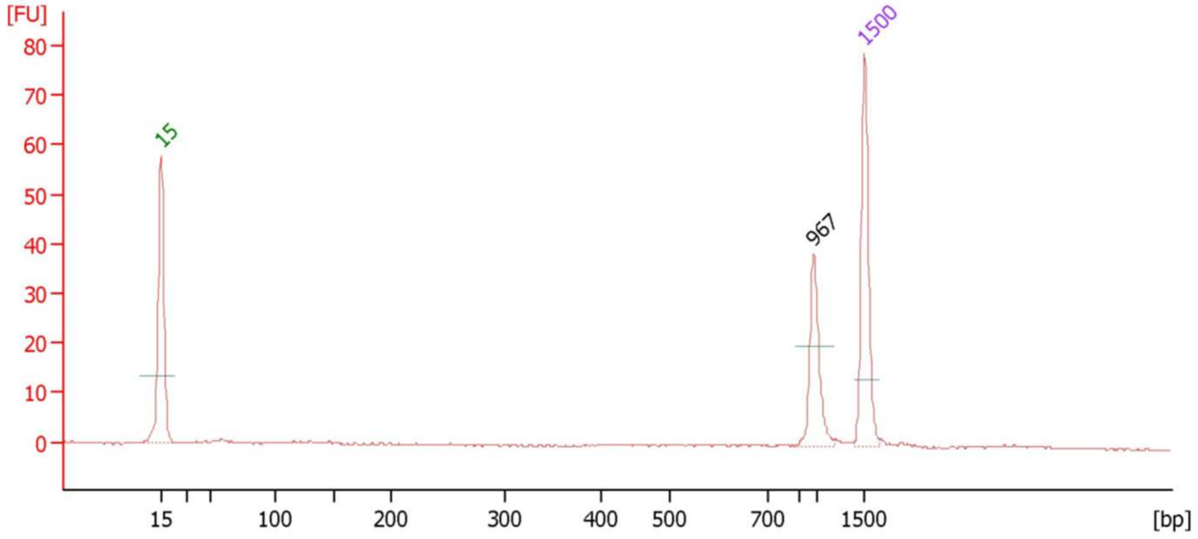

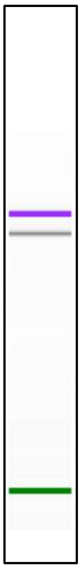

Peak table for Sample #4 :

Peak		Size [bp]	Conc. [ng/µl]	Molarity [nmol/l]	Observations
1	◀	15	4.20	424.2	Lower Marker
2	L	967	1.43	2.2	Ladder Peak
3	▶	1,500	2.10	2.1	Upper Marker

© Copyright 2003 - 2009 Agilent Technologies, Inc.

Assay Class: DNA 1000
Data Path: C:\...-30\2100 expert_DNA 1000_DE34903611_2023-03-06_TRN_1_10.xad

Created: 3/06/2023 4:21:53 PM
Modified: 3/06/2023 4:21:53 PM

Electrophoresis File Run Summary

Sample #5: CA3

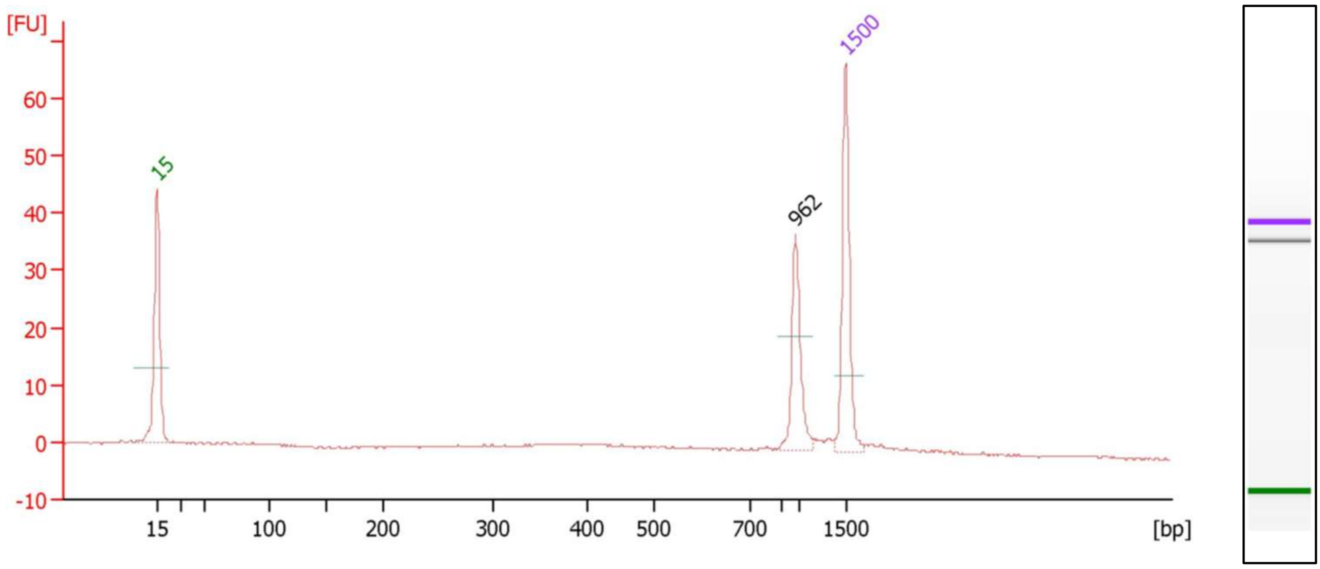

Peak table for Sample #5 :

Peak		Size [bp]	Conc. [ng/µl]	Molarity [nmol/l]	Observations
1		15	4.20	424.2	Lower Marker
2		962	1.58	2.5	Ladder Peak
3		1,500	2.10	2.1	Upper Marker

Assay Class: DNA 1000
Data Path: C:\...-30\2100 expert_DNA 1000_DE34903611_2023-03-06_TRN_1_10.xad

Created: 3/06/2023 4:21:53 PM
Modified: 3/06/2023 4:21:53 PM

Electrophoresis File Run Summary

Sample #6: CC3

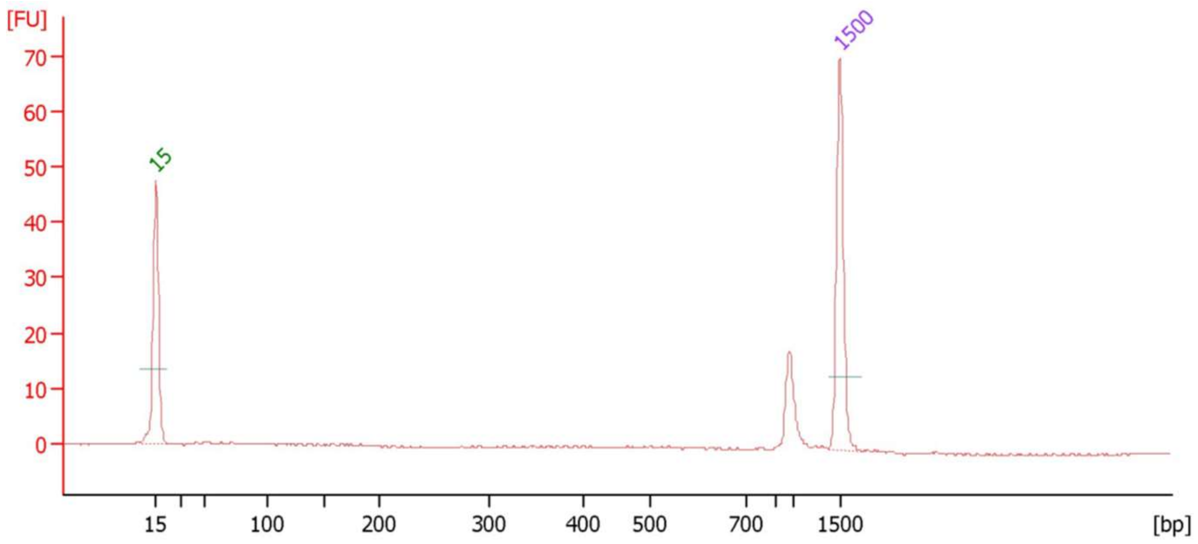

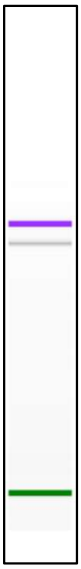

Peak table for Sample #6 :

Peak		Size [bp]	Conc. [ng/µl]	Molarity [nmol/l]	Observations
1	◀	15	4.20	424.4	Lower Marker
2	▶	1,500	2.10	2.1	Upper Marker

 © Copyright 2003 - 2009 Agilent Technologies, Inc.

Assay Class: DNA 1000
Data Path: C:\...-30\2100 expert_DNA 1000_DE34903611_2023-03-06_TRN_1_10.xad

Created: 3/06/2023 4:21:53 PM
Modified: 3/06/2023 4:21:53 PM

Electrophoresis File Run Summary

Sample #7: CC4

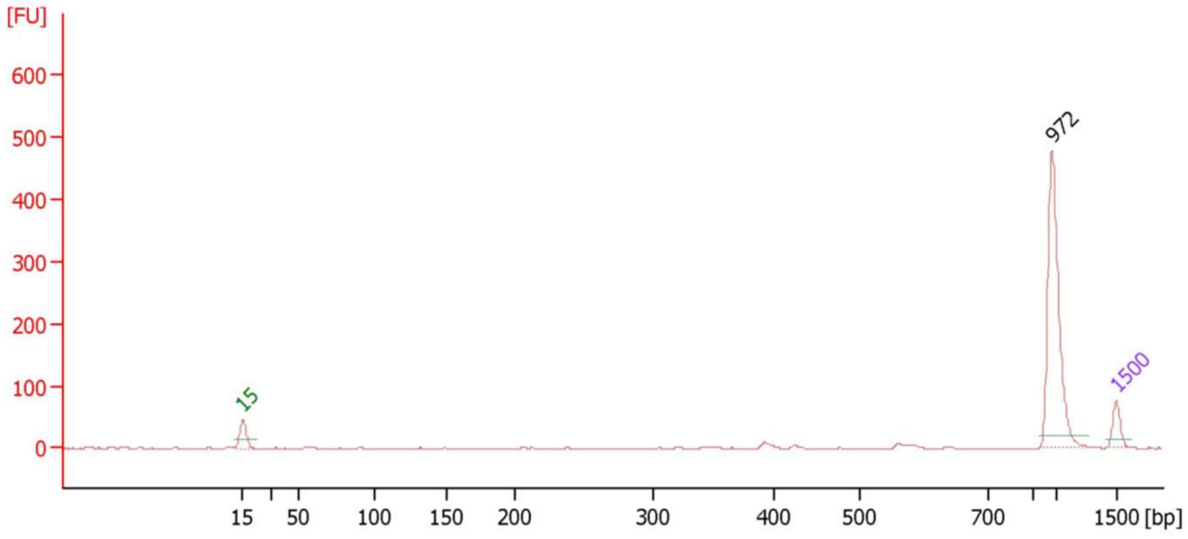

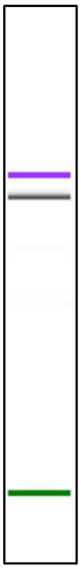

Peak table for Sample #7 :

Peak		Size [bp]	Conc. [ng/µl]	Molarity [nmol/l]	Observations
**1**		15	4.20	424.4	Lower Marker
**2**		972	20.68	32.2	Ladder Peak
**3**		1,500	2.10	2.2	Upper Marker

 © Copyright 2003 - 2009 Agilent Technologies, Inc.

Assay Class: DNA 1000
Data Path: C:\...-30\2100 expert_DNA 1000_DE34903611_2023-03-06_TRN_1_10.xad

Created: 3/06/2023 4:21:53 PM
Modified: 3/06/2023 4:21:53 PM

Electrophoresis File Run Summary

Sample #8: CA4

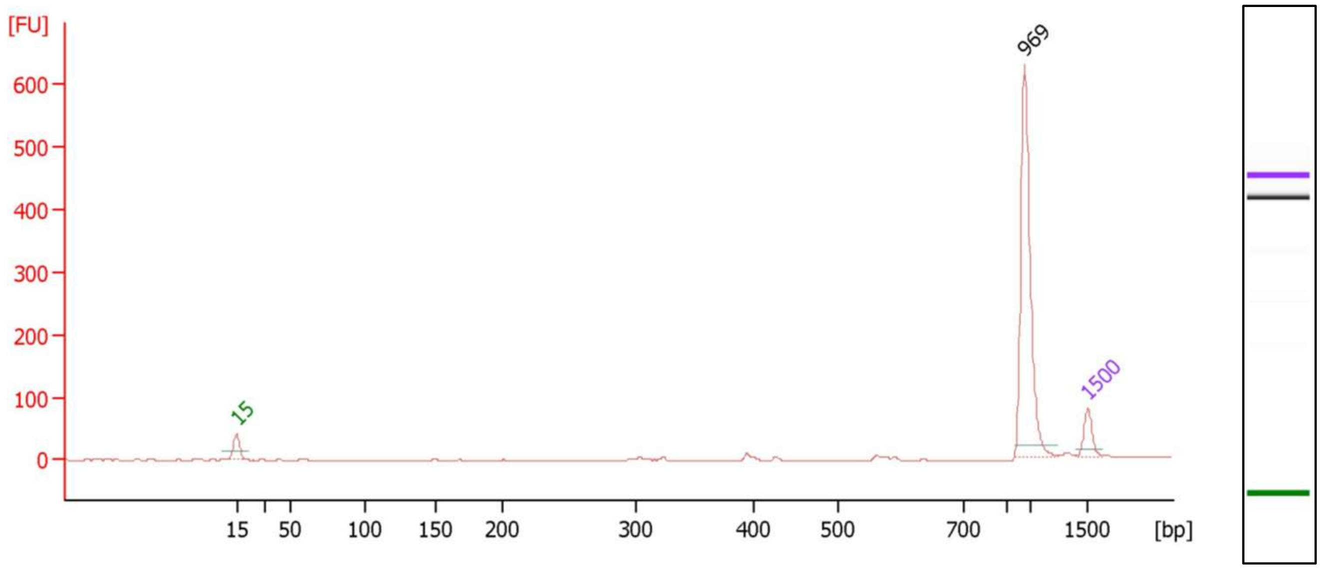

Peak table for Sample #8 :

Peak		Size [bp]	Conc. [ng/µl]	Molarity [nmol/l]	Observations
1	◀	15	4.20	424.4	Lower Marker
2	L	969	20.84	32.6	Ladder Peak
3	▶	1,500	2.10	2.2	Upper Marker

© Copyright 2003 - 2009 Agilent Technologies, Inc.

Assay Class: DNA 1000
Data Path: C:\...-30\2100 expert_DNA 1000_DE34903611_2023-03-06_TRN_1_10.xad

Created: 3/06/2023 4:21:53 PM
Modified: 3/06/2023 4:21:53 PM

Electrophoresis File Run Summary

Sample #9: CA5

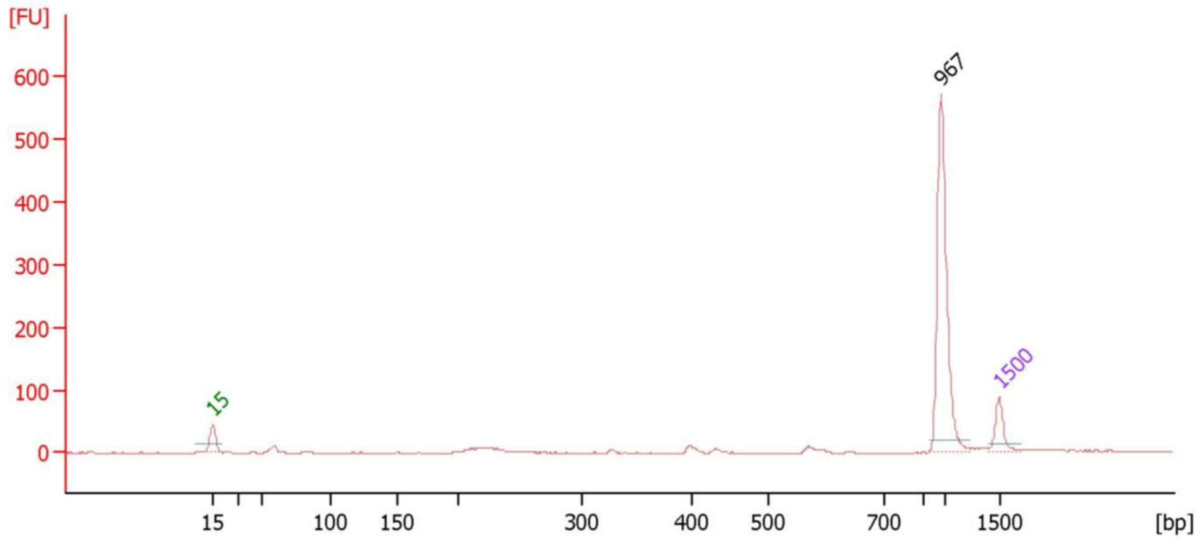

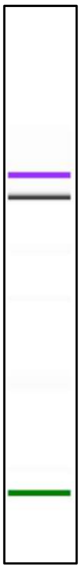

Peak table for Sample #9 :

Peak		Size [bp]	Conc. [ng/µl]	Molarity [nmol/l]	Observations
1		15	4.20	424.4	Lower Marker
2		967	16.84	26.4	Ladder Peak
3		1,500	2.10	2.2	Upper Marker

© Copyright 2003 - 2009 Agilent Technologies, Inc.

Assay Class: DNA 1000
Data Path: C:\...-30\2100 expert_DNA 1000_DE34903611_2023-03-06_TRN_1_10.xad

Created: 3/06/2023 4:21:53 PM
Modified: 3/06/2023 4:21:53 PM

Electrophoresis File Run Summary

Sample #10: CA6

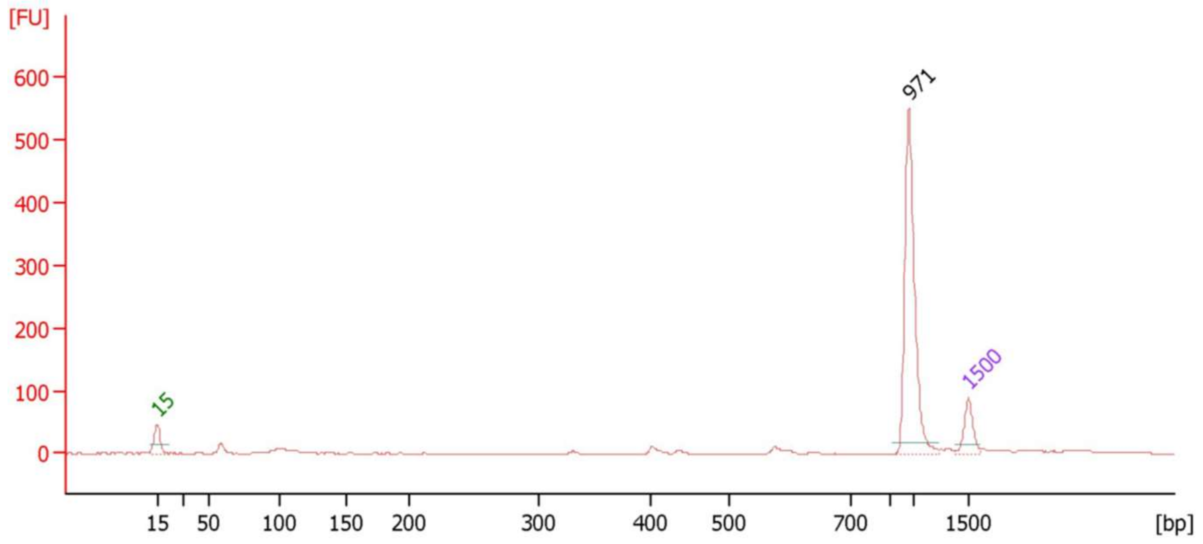

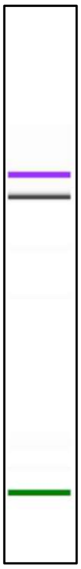

Peak table for Sample #10 :

Peak		Size [bp]	Conc. [ng/µl]	Molarity [nmol/l]	Observations
1	◀	15	4.20	424.4	Lower Marker
2	L	962	15.46	24.5	Ladder Peak
3	▶	1,500	2.10	2.1	Upper Marker

 © Copyright 2003 - 2009 Agilent Technologies, Inc.

Assay Class: DNA 1000
Data Path: C:\...-30\2100 expert_DNA 1000_DE34903611_2023-03-06_TRN_1_10.xad

Created: 3/06/2023 4:21:53 PM
Modified: 3/06/2023 4:21:53 PM

Electrophoresis File Run Summary

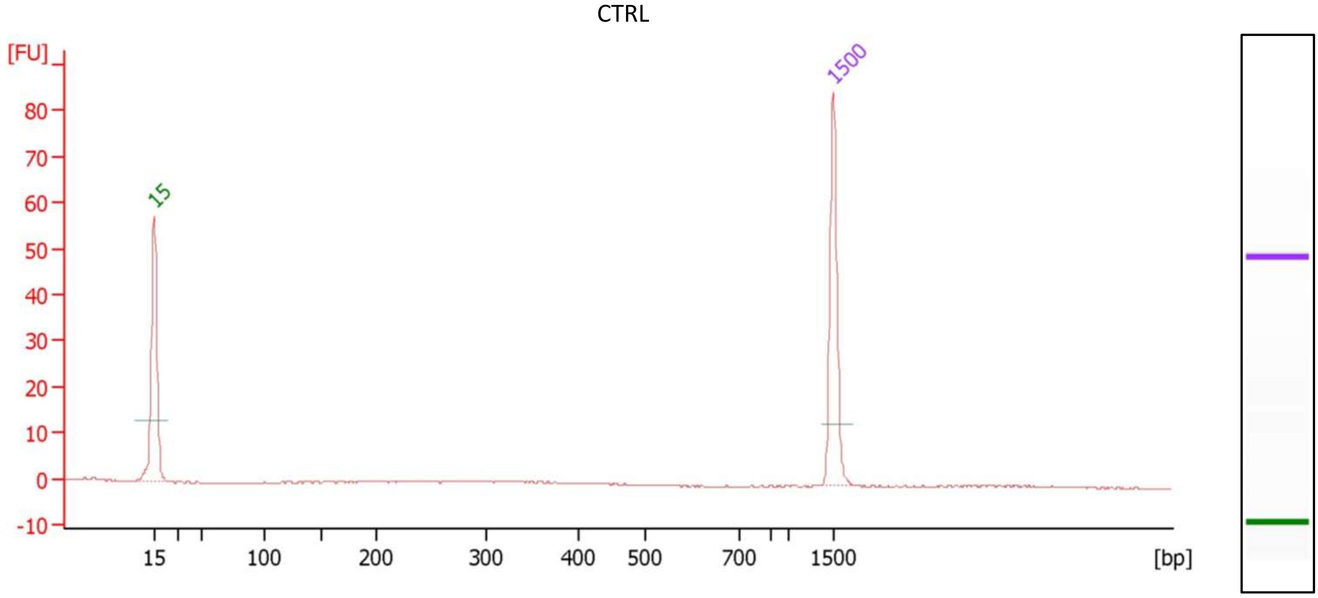

Peak table for Sample CTRL :

Peak		Size [bp]	Conc. [ng/µl]	Molarity [nmol/l]	Observations
1	◀	15	4.20	424.4	Lower Marker
2	▶	1,500	2.10	2.2	Upper Marker

 © Copyright 2003 - 2009 Agilent Technologies, Inc.

Assay Class: DNA 1000
Data Path: C:\...-30\2100 expert_DNA 1000_DE34903611_2023-03-06_TRN_1_10.xad

Created: 3/06/2023 4:21:53 PM
Modified: 3/06/2023 4:21:53 PM

Electrophoresis File Run Summary

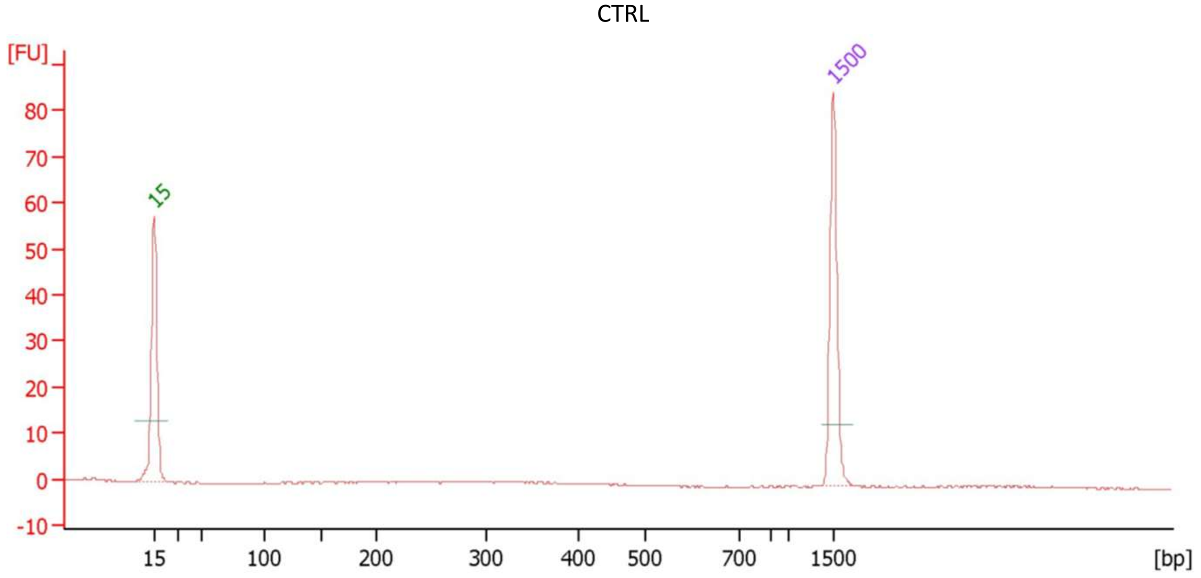

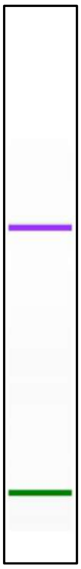

Peak table for Sample CTRL :

Peak		Size [bp]	Conc. [ng/µl]	Molarity [nmol/l]	Observations
1	◀	15	4.20	424.4	Lower Marker
2	▶	1,500	2.10	2.1	Upper Marker

 © Copyright 2003 - 2009 Agilent Technologies, Inc.

Assay Class: DNA 1000
Data Path: C:\...-30\2100 expert_DNA 1000_DE34903611_2023-03-06_TRN_1_10.xad

Created: 3/06/2023 4:21:53 PM
Modified: 3/06/2023 4:21:53 PM

Curves

Standard Curve

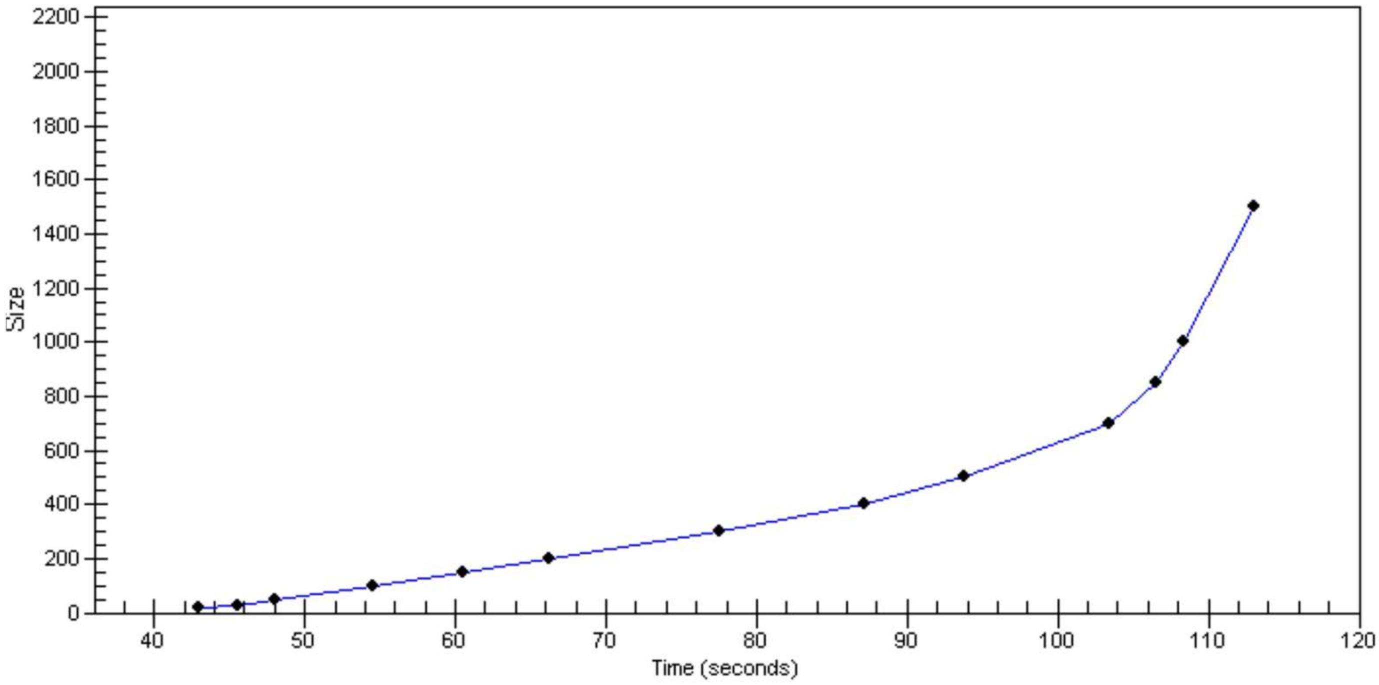

Assay Class: DNA 1000
Data Path: C:\...-30\2100 expert_DNA 1000_DE34903611_2023-03-06_TRN_1_10.xad

Created: 3/06/2023 4:21:53 PM
Modified: 3/06/2023 4:21:53 PM

Electrophoresis File Run Summary (Chip Summary)

Sample Name	Sample Content	Rest. Digest	Status	Observation	Result Label	Result Color
Sample #1		☐	✔			
Sample #2		☐	✔			
Sample #3		☐	✔			
Sample #4		☐	✔			
Sample #5		☐	✔			
Sample #6		☐	✔			
Sample #7		☐	✔			
Sample #8		☐	✔			
Sample #9		☐	✔			
Sample #10		☐	✔			
CTRL		☐	✔			
CTRL		☐	✔			

Chip Lot # **Reagent Kit Lot #**

Chip Comments :

© Copyright 2003 - 2009 Agilent Technologies, Inc.

Assay Class: DNA 1000
Data Path: C:\...-30\2100 expert_DNA 1000_DE34903611_2023-03-06_TRN_1_10.xad
Created: 3/06/2023 4:21:53 PM
Modified: 3/06/2023 4:21:53 PM

Electrophoresis Assay Details

General Analysis Settings

Result Label **Result Color**

Number of Available Sample and Ladder Wells (Max.) : 13
Minimum Visible Range [s] : 30
Maximum Visible Range [s] : 129
Start Analysis Time Range [s] : 30
End Analysis Time Range [s] : 128.95
Ladder Concentration [ng/µl] : 44
Uses Standard Area for Ladder Fragments
Lower Marker Concentration [ng/µl] : 4.2
Upper Marker Concentration [ng/µl] : 2.1
Used Upper Marker for Quantitation
Standard Curve Fit is Point to Point
Show Data Aligned to Lower and Upper Marker

Integrator Settings

Integration Start Time [s] : 30
Integration End Time [s] : 128.95
Slope Threshold : 0.5
Height Threshold [FU] : 20
Area Threshold : 0.1
Width Threshold [s] : 0.5
Baseline Plateau [s] : 0.5

Filter Settings

Filter Width [s] : 0.5
Polynomial Order : 4

Ladder

Ladder Peak	Size	Area
1	15	25
2	25	26
3	50	34
4	100	41
5	150	45
6	200	52
7	300	63
8	400	76
9	500	83
10	700	88
11	850	86
12	1000	90
13	1500	52

© Copyright 2003 - 2009 Agilent Technologies, Inc.

Assay Class: DNA 1000
Data Path: C:\...-30\2100 expert_DNA 1000_DE34903611_2023-03-06_TRN_11_20.xad

Created: 3/07/2023 9:17:24 AM
Modified: 3/07/2023 9:17:24 AM

Electrophoresis File Run Summary

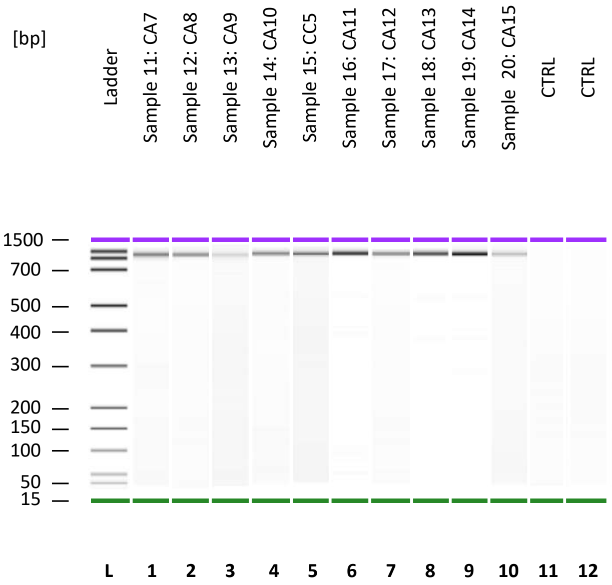

Instrument Information:

Instrument Name: DE34903611

Firmware: C.01.069

Serial #: DE34903611

Type: G2938C

Assay Information:

Assay Origin Path: C:\Program Files\Agilent\2100 bioanalyzer\2100 expert\assays\dsDNA\DNA 1000 Series II.xsy

Assay Class: DNA 1000

Version: 2.3

Assay Comments: DNA Analysis 2 - 1000 bp

© Copyright 2003-2009 Agilent Technologies, Inc.

Chip Information:

Chip Lot #:

Reagent Kit Lot #:

Chip Comments:

Sample 11: CA7

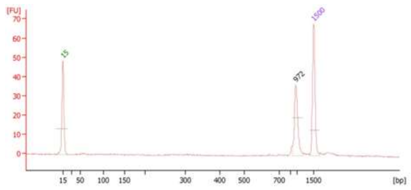

Sample 12: CA8

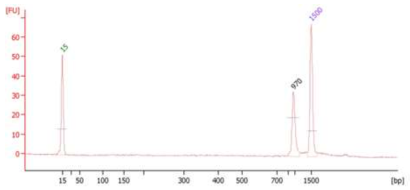

Sample 13: CA9

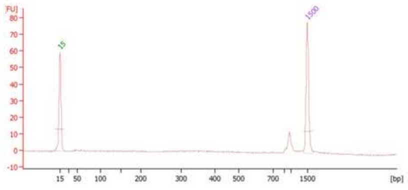

Sample 14: CA10

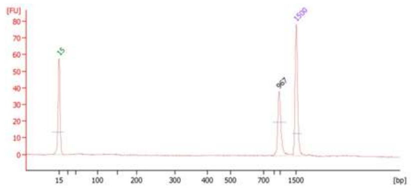

Sample 15: CC5

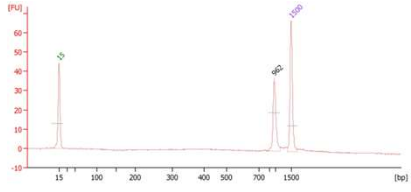

Sample 16: CA11

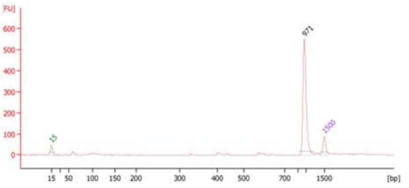

Sample 17: CA12

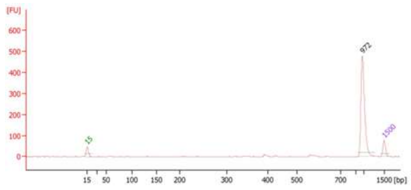

Sample 18: CA13

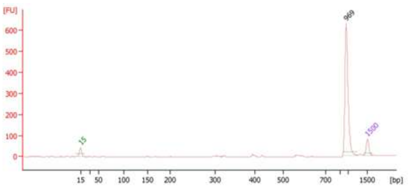

Sample 19: CA14

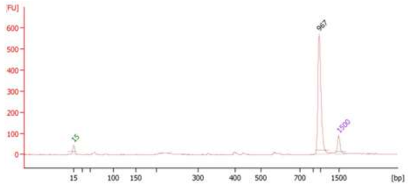

Sample 20: CA15

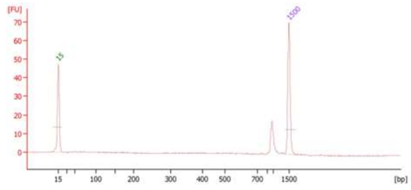

CTRL

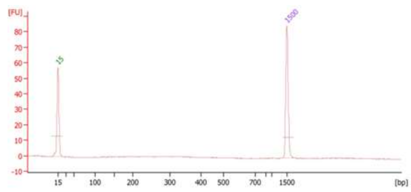

CTRL

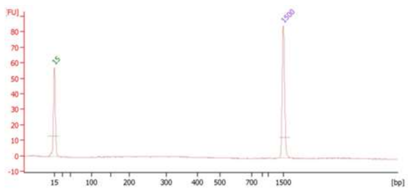

Assay Class: DNA 1000
Data Path: C:\...-30\2100 expert_DNA 1000_DE34903611_2023-03-06_TRN_11_20.xad

Created: 3/07/2023 9:17:24 AM
Modified: 3/07/2023 9:17:24 AM

Electrophoresis File Run Summary

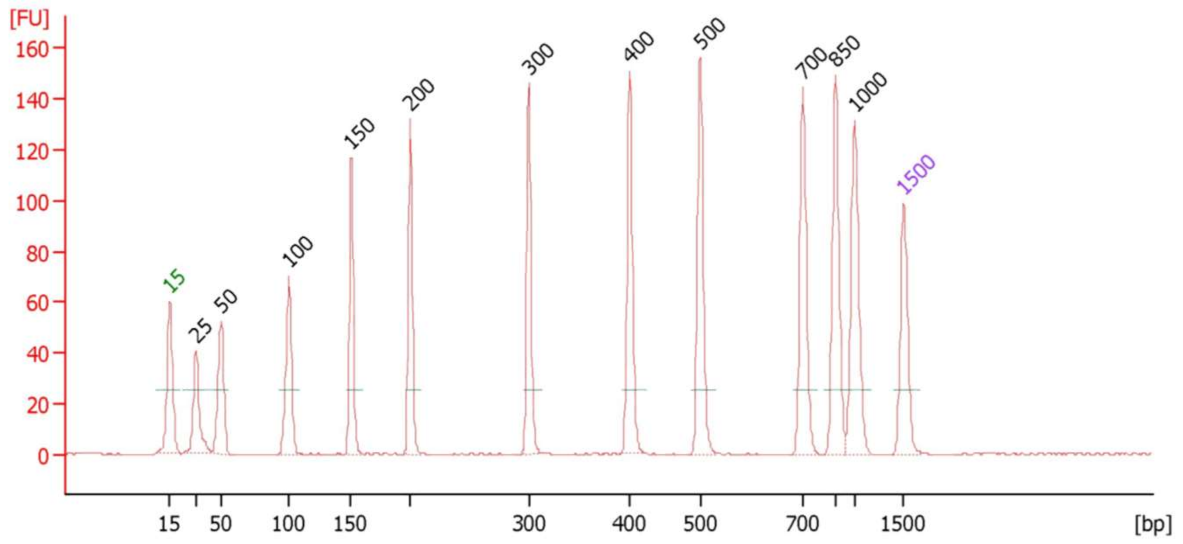

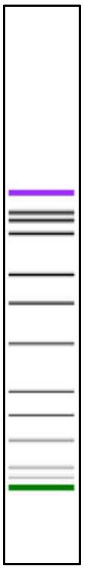

Peak table for Ladder :

Peak		Size [bp]	Conc. [ng/µl]	Molarity [nmol/l]	Observations
**1**	◀	15	4.20	4.4.2	Lower Marker
**2**	L	25	4.00	242.4	Ladder Peak
**3**	L	50	4.00	121.2	Ladder Peak
**4**	L	100	4.00	60.6	Ladder Peak
**5**	L	150	4.00	40.3	Ladder Peak
**6**	L	200	4.00	30.3	Ladder Peak
**7**	L	300	4.00	20.2	Ladder Peak
**8**	L	400	4.00	15.2	Ladder Peak
**9**	L	500	4.00	12.1	Ladder Peak
**10**	L	700	4.00	8.7	Ladder Peak
**11**	L	850	4.00	7.1	Ladder Peak
**12**	L	1,000	4.00	6.1	Ladder Peak
**13**	▶	1,500	2.10	2.1	Upper Marker

 © Copyright 2003 - 2009 Agilent Technologies, Inc.

Assay Class: DNA 1000
Data Path: C:\...-30\2100 expert_DNA 1000_DE34903611_2023-03-06_TRN_11_20.xad

Created: 3/07/2023 9:17:24 AM
Modified: 3/07/2023 9:17:24 AM

Electrophoresis File Run Summary

Sample #11: CA7

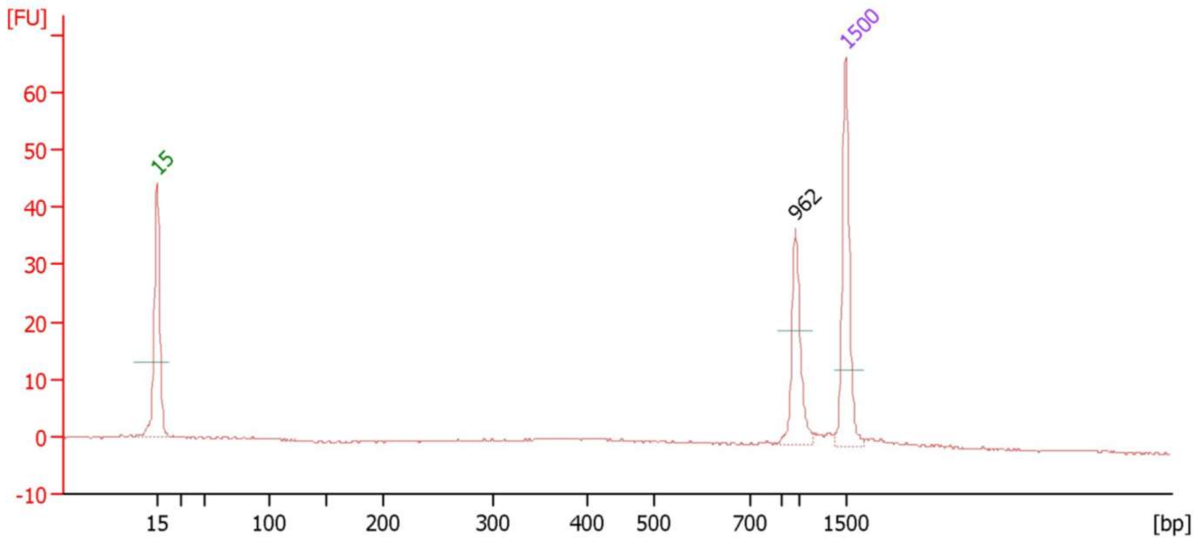

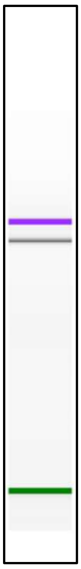

Peak table for Sample #11 :

Peak		Size [bp]	Conc. [ng/µl]	Molarity [nmol/l]	Observations
1	◀	15	4.20	424.2	Lower Marker
2	L	962	1.58	2.5	Ladder Peak
3	▶	1,500	2.10	2.1	Upper Marker

 © Copyright 2003 - 2009 Agilent Technologies, Inc.

Assay Class: DNA 1000
Data Path: C:\...-30\2100 expert_DNA 1000_DE34903611_2023-03-06_TRN_11_20.xad
Created: 3/07/2023 9:17:24 AM
Modified: 3/07/2023 9:17:24 AM

Electrophoresis File Run Summary

Sample #12: CA8

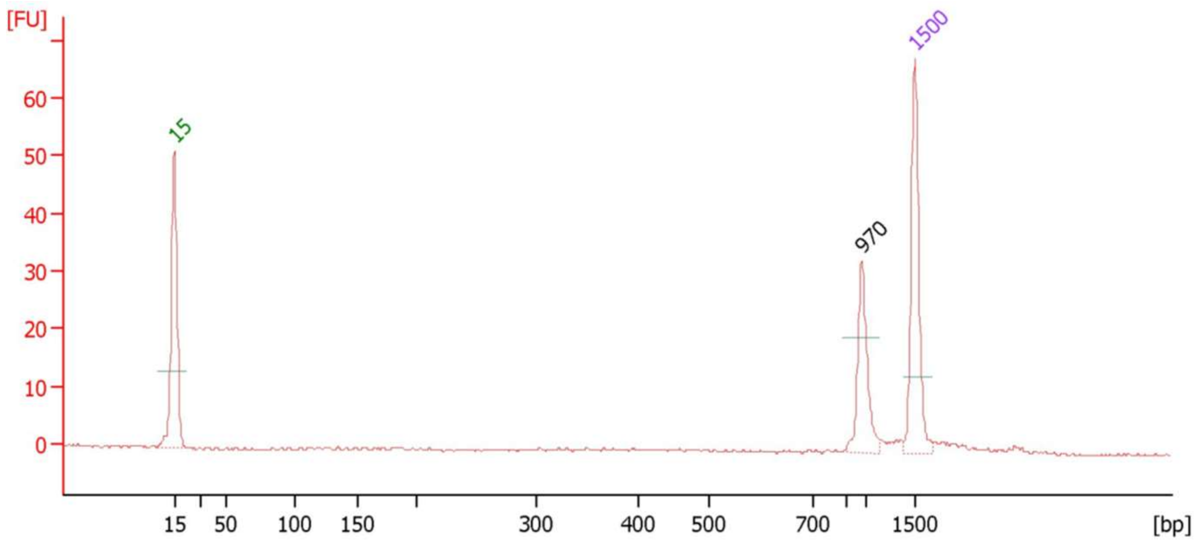

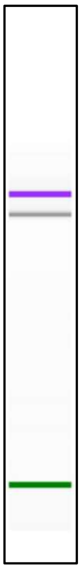

Peak table for Sample #12 :

Peak		Size [bp]	Conc. [ng/µl]	Molarity [nmol/l]	Observations
1	◀	15	4.20	424.4	Lower Marker
2	L	970	1.41	2.2	Ladder Peak
3	▶	1,500	2.10	2.2	Upper Marker

© Copyright 2003 - 2009 Agilent Technologies, Inc.

Assay Class: DNA 1000
Data Path: C:\...-30\2100 expert_DNA 1000_DE34903611_2023-03-06_TRN_11_20.xad
Created: 3/07/2023 9:17:24 AM
Modified: 3/07/2023 9:17:24 AM

Electrophoresis File Run Summary

Sample #13: CA9

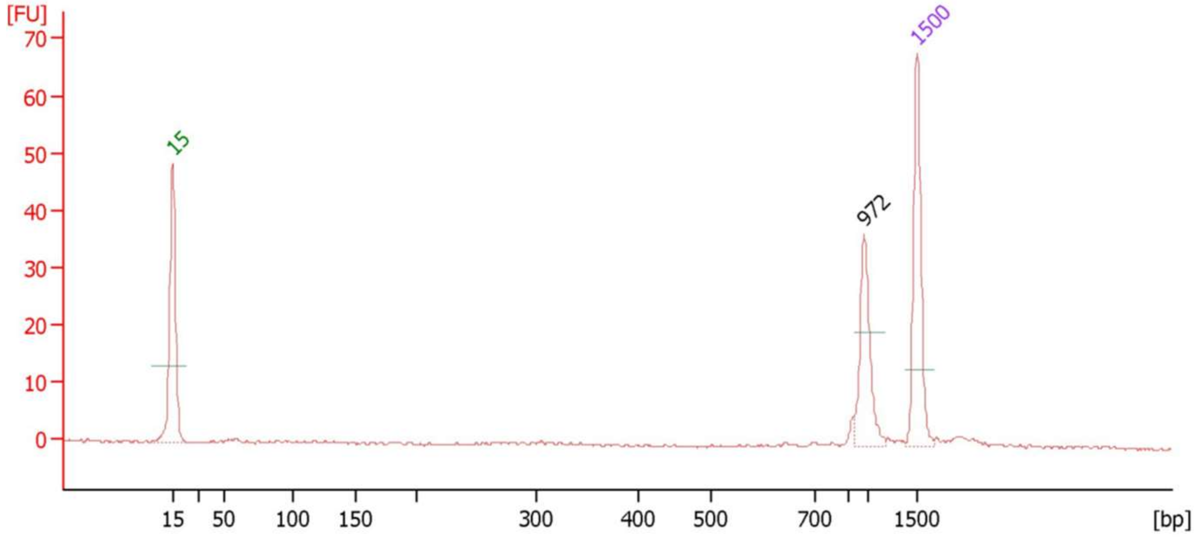

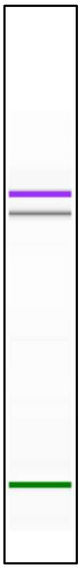

Peak table for Sample #13 :

Peak		Size [bp]	Conc. [ng/µl]	Molarity [nmol/l]	Observations
1		15	4.20	424.4	Lower Marker
2		972	1.63	2.5	Ladder Peak
3		1,500	2.10	2.2	Upper Marker

© Copyright 2003 - 2009 Agilent Technologies, Inc.

Assay Class: DNA 1000
Data Path: C:\...-30\2100 expert_DNA 1000_DE34903611_2023-03-06_TRN_11_20.xad

Created: 3/07/2023 9:17:24 AM
Modified: 3/07/2023 9:17:24 AM

Electrophoresis File Run Summary

Sample #14: CA10

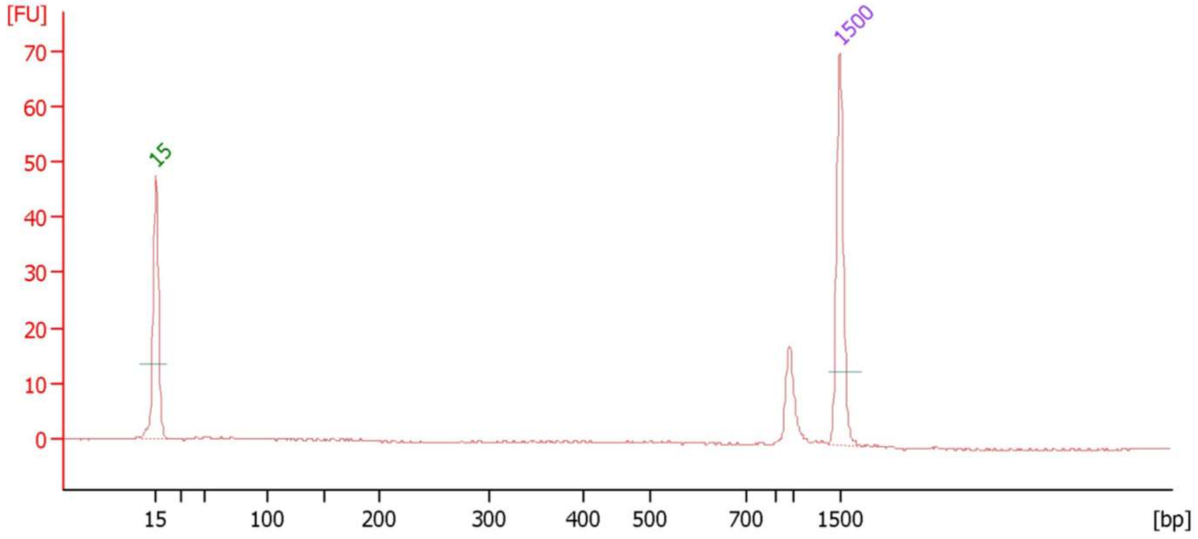

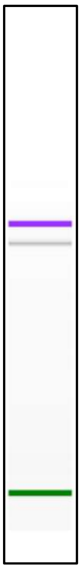

Peak table for Sample #14 :

Peak		Size [bp]	Conc. [ng/µl]	Molarity [nmol/l]	Observations
1	◀	15	4.20	424.4	Lower Marker
2	▶	1,500	2.10	2.1	Upper Marker

© Copyright 2003 - 2009 Agilent Technologies, Inc.

Assay Class: DNA 1000
Data Path: C:\...-30\2100 expert_DNA 1000_DE34903611_2023-03-06_TRN_11_20.xad

Created: 3/07/2023 9:17:24 AM
Modified: 3/07/2023 9:17:24 AM

Electrophoresis File Run Summary

Sample #15: CC5

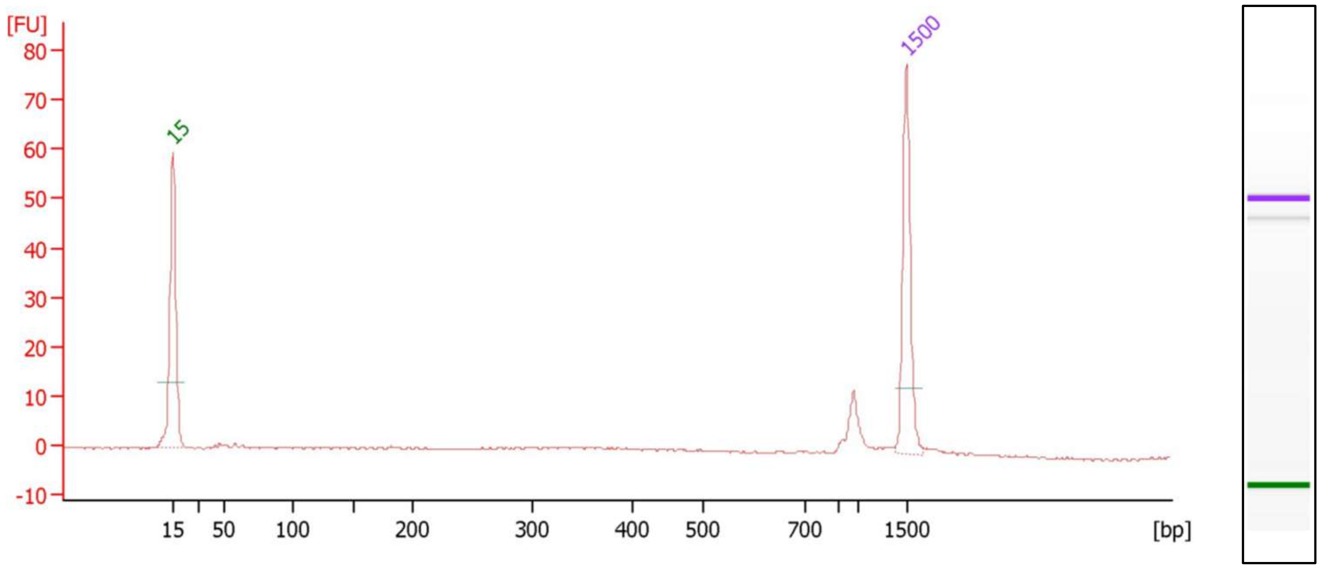

Peak table for Sample #15 :

Peak		Size [bp]	Conc. [ng/µl]	Molarity [nmol/l]	Observations
1	◀	15	4.20	424.4	Lower Marker
2	▶	1,500	2.10	2.1	Upper Marker

Assay Class: DNA 1000
Data Path: C:\...-30\2100 expert_DNA 1000_DE34903611_2023-03-06_TRN_11_20.xad

Created: 3/07/2023 9:17:24 AM
Modified: 3/07/2023 9:17:24 AM

Electrophoresis File Run Summary

Sample #16: CA11

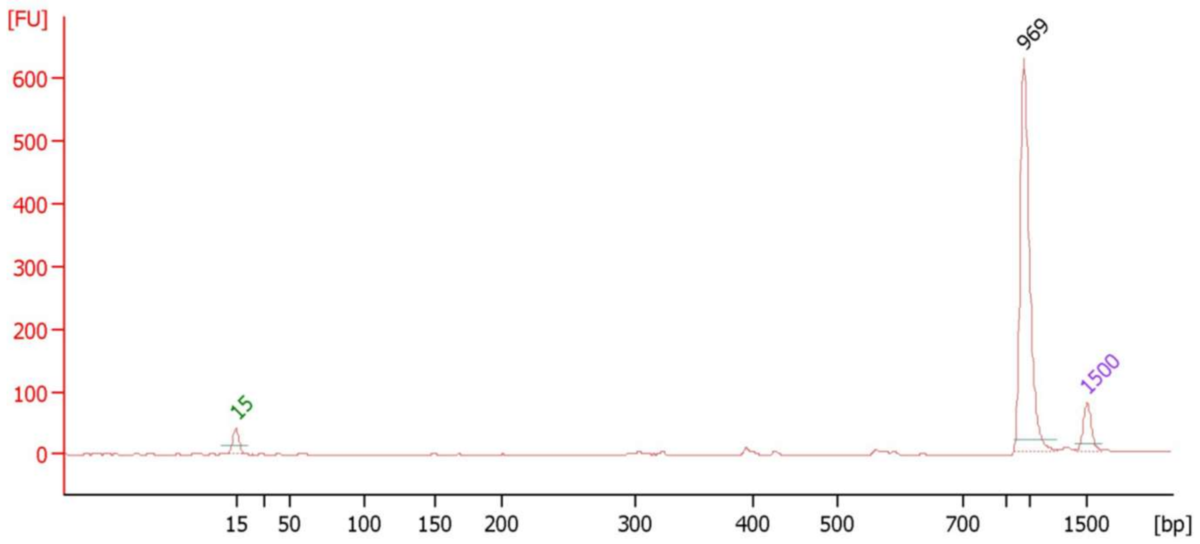

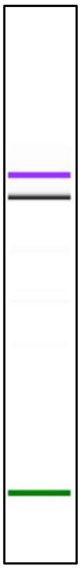

Peak table for Sample #16 :

Peak		Size [bp]	Conc. [ng/µl]	Molarity [nmol/l]	Observations
1	◀	15	4.20	424.4	Lower Marker
2	L	969	20.84	32.6	Ladder Peak
3	▶	1,500	2.10	2.2	Upper Marker

© Copyright 2003 - 2009 Agilent Technologies, Inc.

Assay Class: DNA 1000
Data Path: C:\...-30\2100 expert_DNA 1000_DE34903611_2023-03-06_TRN_11_20.xad

Created: 3/07/2023 9:17:24 AM
Modified: 3/07/2023 9:17:24 AM

Electrophoresis File Run Summary

Sample #17: CA12

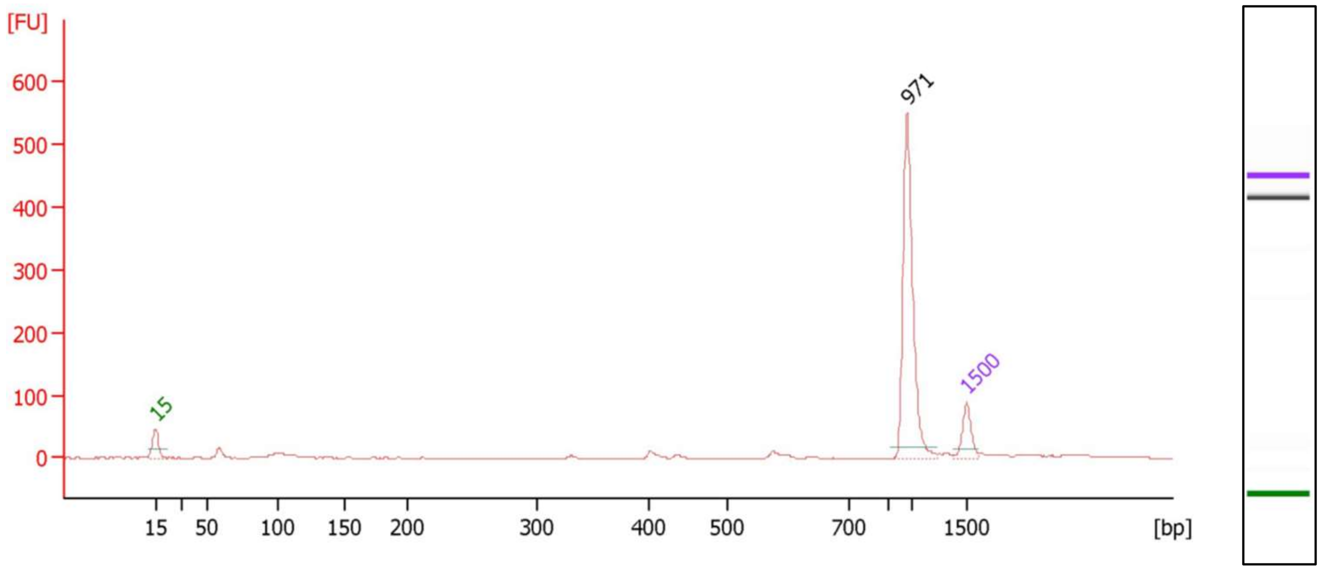

Peak table for Sample #17 :

Peak		Size [bp]	Conc. [ng/µl]	Molarity [nmol/l]	Observations
1		15	4.20	424.4	Lower Marker
2		962	15.46	24.5	Ladder Peak
3		1,500	2.10	2.1	Upper Marker

 © Copyright 2003 - 2009 Agilent Technologies, Inc.

Assay Class: DNA 1000
Data Path: C:\...-30\2100 expert_DNA 1000_DE34903611_2023-03-06_TRN_11_20.xad

Created: 3/07/2023 9:17:24 AM
Modified: 3/07/2023 9:17:24 AM

Electrophoresis File Run Summary

Sample #18: CA13

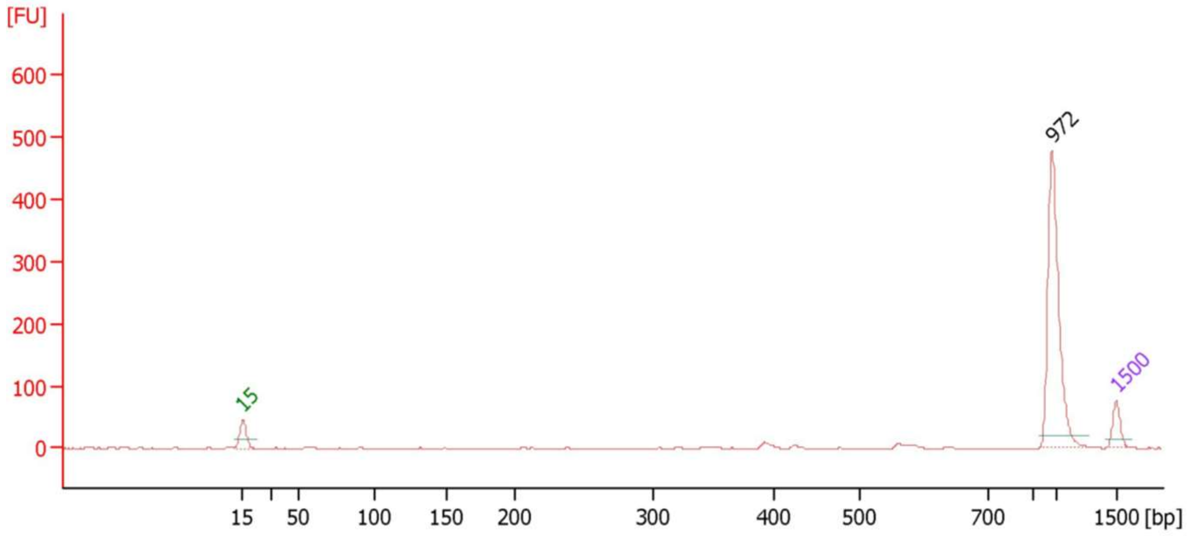

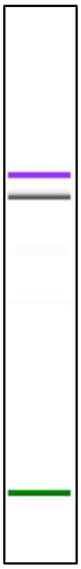

Peak table for Sample #7 :

Peak		Size [bp]	Conc. [ng/µl]	Molarity [nmol/l]	Observations
1		15	4.20	424.4	Lower Marker
2		972	20.68	32.2	Ladder Peak
3		1,500	2.10	2.2	Upper Marker

© Copyright 2003 - 2009 Agilent Technologies, Inc.

Assay Class: DNA 1000
Data Path: C:\...-30\2100 expert_DNA 1000_DE34903611_2023-03-06_TRN_11_20.xad
Created: 3/07/2023 9:17:24 AM
Modified: 3/07/2023 9:17:24 AM

Electrophoresis File Run Summary

Sample #19: CA14

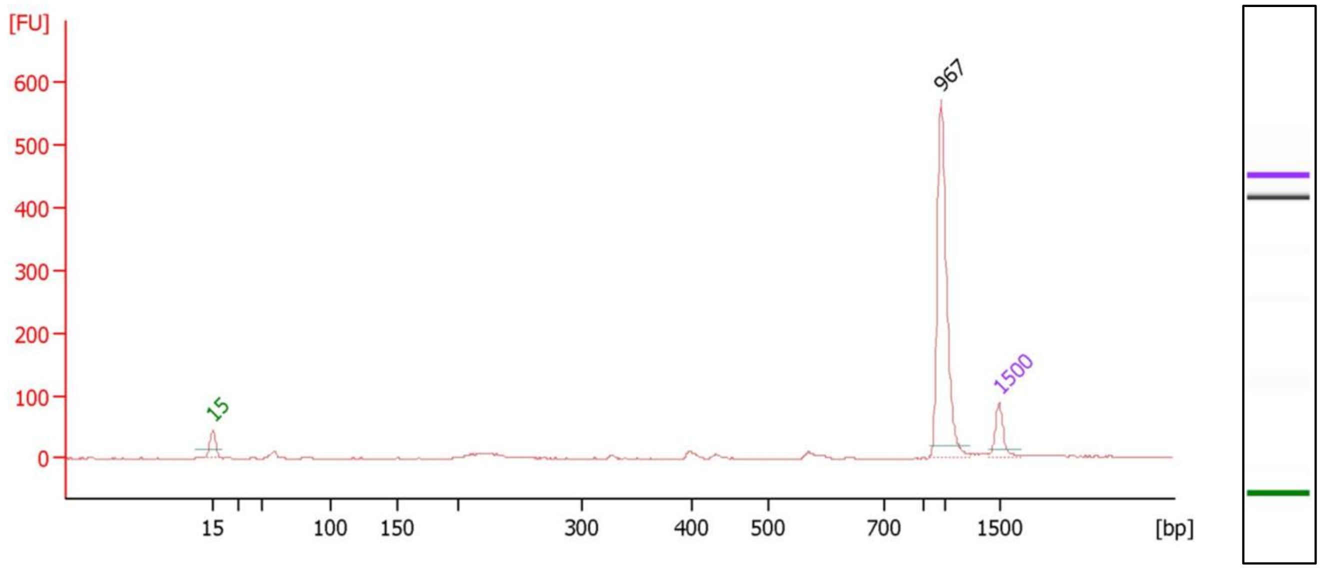

Peak table for Sample #9 :

Peak		Size [bp]	Conc. [ng/µl]	Molarity [nmol/l]	Observations
1	◀	15	4.20	424.4	Lower Marker
2	L	967	16.84	26.4	Ladder Peak
3	▶	1,500	2.10	2.2	Upper Marker

 © Copyright 2003 - 2009 Agilent Technologies, Inc.

Assay Class: DNA 1000
Data Path: C:\...-30\2100 expert_DNA 1000_DE34903611_2023-03-06_TRN_11_20.xad

Created: 3/07/2023 9:17:24 AM
Modified: 3/07/2023 9:17:24 AM

Electrophoresis File Run Summary

Sample #20: CA15

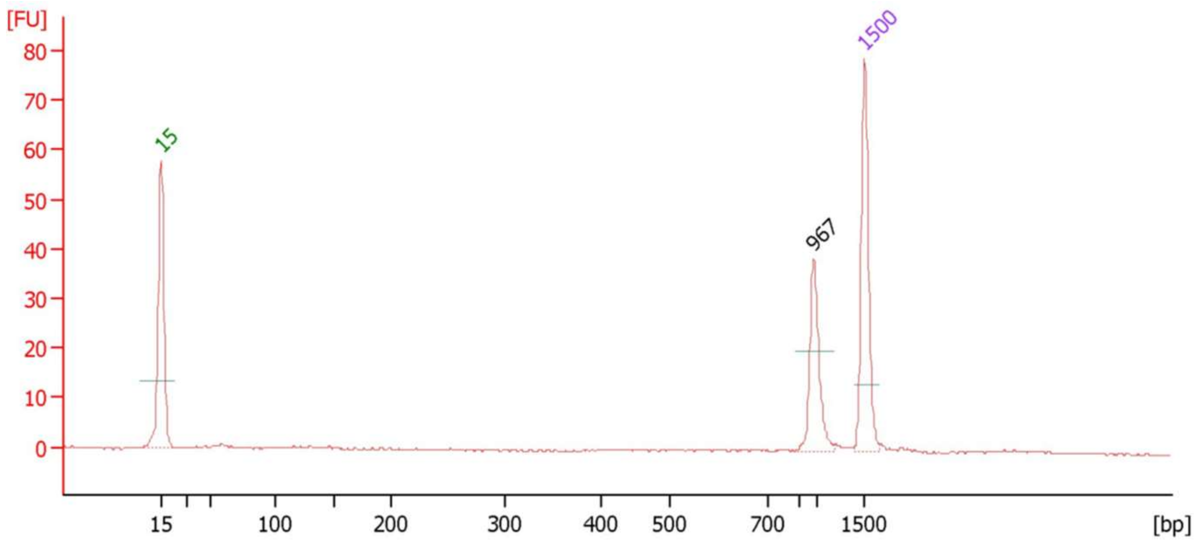

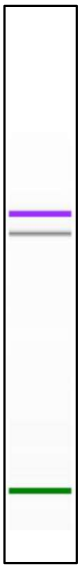

Peak table for Sample #4 :

Peak		Size [bp]	Conc. [ng/µl]	Molarity [nmol/l]	Observations
1	◀	15	4.20	424.2	Lower Marker
2	L	967	1.43	2.2	Ladder Peak
3	▶	1,500	2.10	2.1	Upper Marker

 © Copyright 2003 - 2009 Agilent Technologies, Inc.

Assay Class: DNA 1000
Data Path: C:\...-30\2100 expert_DNA 1000_DE34903611_2023-03-06_TRN_11_20.xad
Created: 3/07/2023 9:17:24 AM
Modified: 3/07/2023 9:17:24 AM

Electrophoresis File Run Summary

CTRL

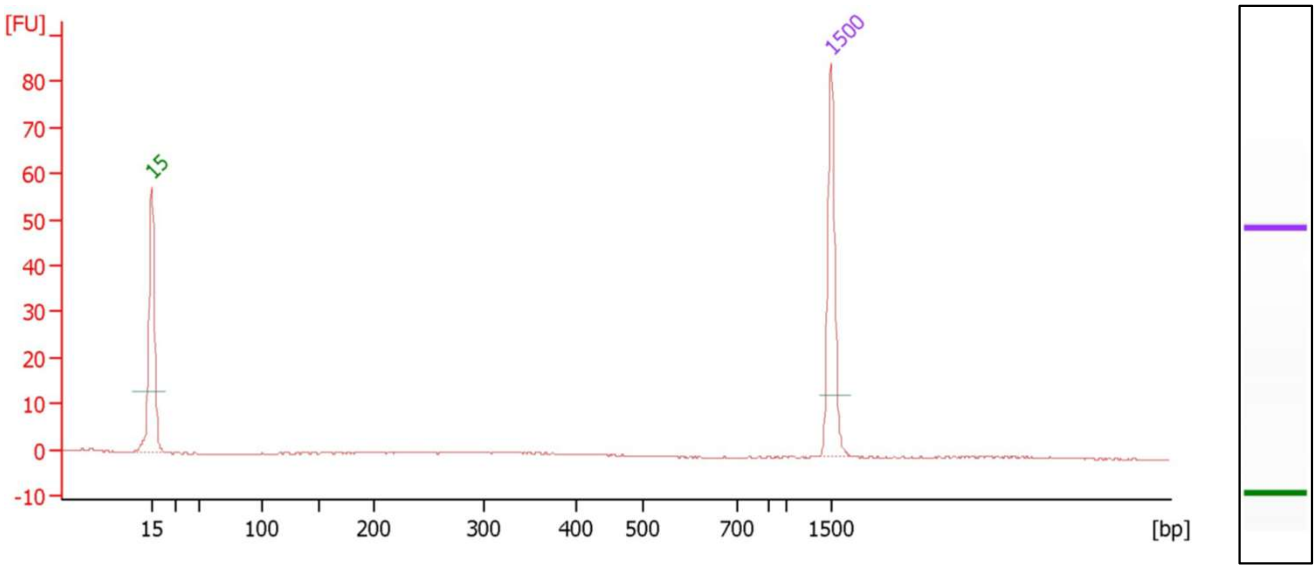

Peak table for Sample CTRL :

Peak		Size [bp]	Conc. [ng/µl]	Molarity [nmol/l]	Observations
1	◀	15	4.20	424.4	Lower Marker
2	▶	1,500	2.10	2.2	Upper Marker

Assay Class: DNA 1000
Data Path: C:\...-30\2100 expert_DNA 1000_DE34903611_2023-03-06_TRN_11_20.xad

Created: 3/07/2023 9:17:24 AM
Modified: 3/07/2023 9:17:24 AM

Electrophoresis File Run Summary

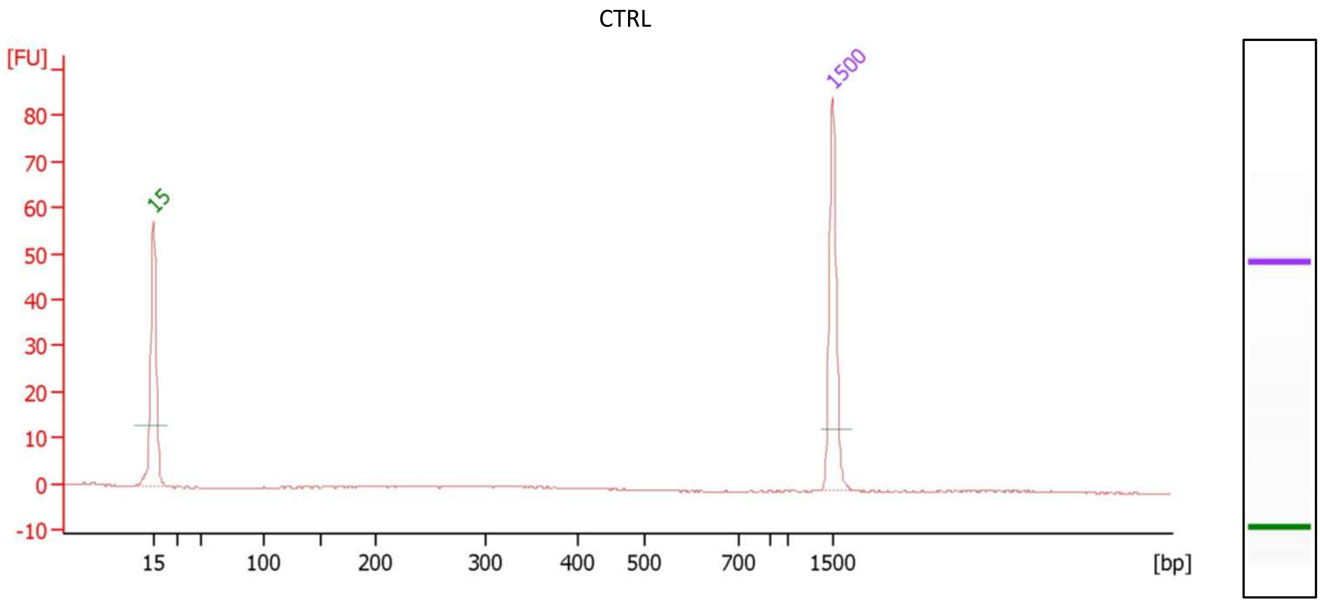

Peak table for Sample CTRL :

Peak		Size [bp]	Conc. [ng/µl]	Molarity [nmol/l]	Observations
1	◀	15	4.20	424.4	Lower Marker
2	▶	1,500	2.10	2.1	Upper Marker

 © Copyright 2003 - 2009 Agilent Technologies, Inc.

Assay Class: DNA 1000
Data Path: C:\...-30\2100 expert_DNA 1000_DE34903611_2023-03-06_TRN_11_20.xad
Created: 3/07/2023 9:17:24 AM
Modified: 3/07/2023 9:17:24 AM

Curves

Standard Curve

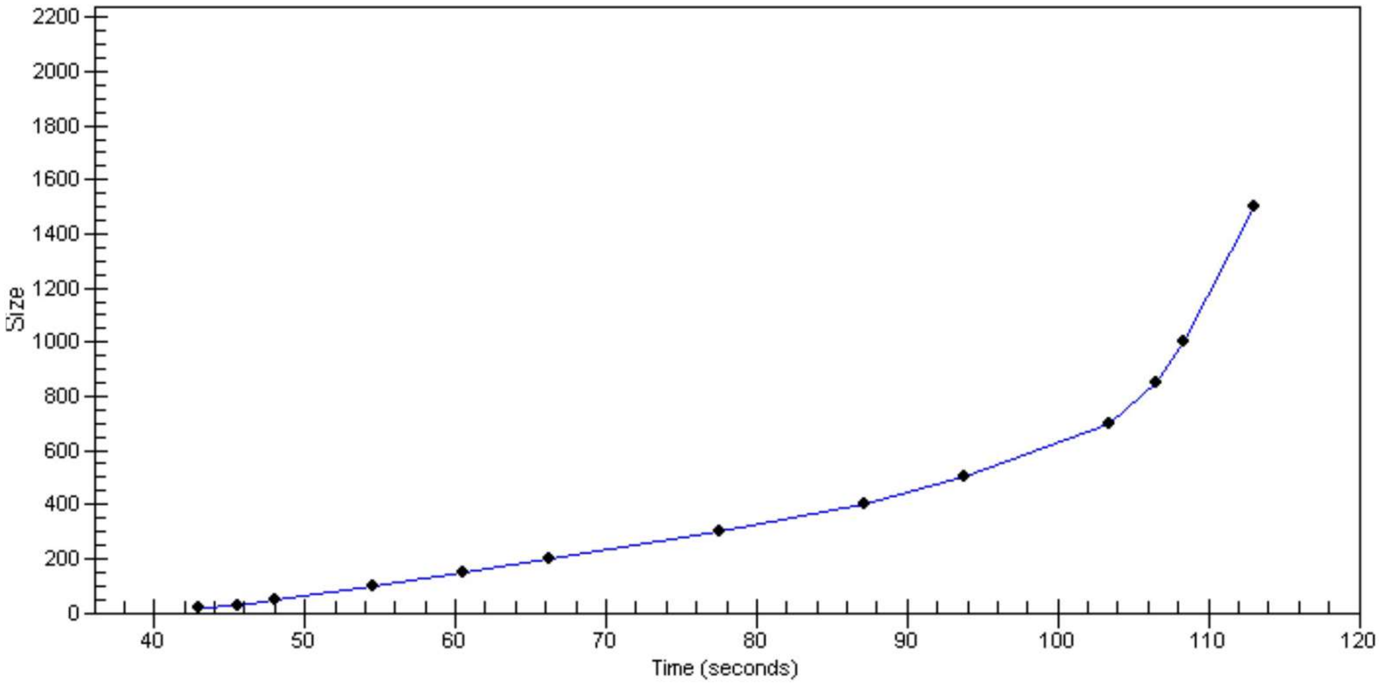

Assay Class: DNA 1000
Data Path: C:\...-30\2100 expert_DNA 1000_DE34903611_2023-03-06_TRN_11_20.xad

Created: 3/07/2023 9:17:24 AM
Modified: 3/07/2023 9:17:24 AM

Electrophoresis File Run Summary (Chip Summary)

Sample Name	Sample Content	Rest. Digest	Status	Observation	Result Label	Result Color
Sample #11		☐	✔			
Sample #12		☐	✔			
Sample #13		☐	✔			
Sample #14		☐	✔			
Sample #15		☐	✔			
Sample #16		☐	✔			
Sample #17		☐	✔			
Sample #18		☐	✔			
Sample #19		☐	✔			
Sample #20		☐	✔			
CTRL		☐	✔			
CTRL		☐	✔			

Chip Lot # **Reagent Kit Lot #**

Chip Comments :

 © Copyright 2003 - 2009 Agilent Technologies, Inc.

Assay Class: DNA 1000
Data Path: C:\...-30\2100 expert_DNA 1000_DE34903611_2023-03-06_TRN_11_20.xad
Created: 3/07/2023 9:17:24 AM
Modified: 3/07/2023 9:17:24 AM

Electrophoresis Assay Details

General Analysis Settings

Result Label **Result Color**

Number of Available Sample and Ladder Wells (Max.) : 13
Minimum Visible Range [s] : 30
Maximum Visible Range [s] : 129
Start Analysis Time Range [s] : 30
End Analysis Time Range [s] : 128.95
Ladder Concentration [ng/µl] : 44
Uses Standard Area for Ladder Fragments
Lower Marker Concentration [ng/µl] : 4.2
Upper Marker Concentration [ng/µl] : 2.1
Used Upper Marker for Quantitation
Standard Curve Fit is Point to Point
Show Data Aligned to Lower and Upper Marker

Integrator Settings

Integration Start Time [s] : 30
Integration End Time [s] : 128.95
Slope Threshold : 0.5
Height Threshold [FU] : 20
Area Threshold : 0.1
Width Threshold [s] : 0.5
Baseline Plateau [s] : 0.5

Filter Settings

Filter Width [s] : 0.5
Polynomial Order : 4

Ladder

Ladder Peak	Size	Area
1	15	25
2	25	26
3	50	34
4	100	41
5	150	45
6	200	52
7	300	63
8	400	76
9	500	83
10	700	88
11	850	86
12	1000	90
13	1500	52

 © Copyright 2003 - 2009 Agilent Technologies, Inc.

Assay Class: DNA 1000
Data Path: C:\...-30\2100 expert_DNA 1000_DE34903611_2023-03-06_TRN_AluI_1_10.xad

Created: 3/07/2023 9:17:20 PM
Modified: 3/07/2023 9:17:20 PM

Electrophoresis File Run Summary

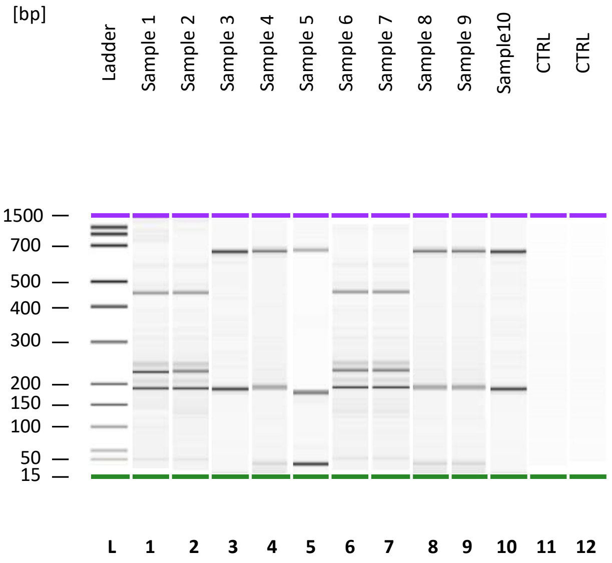

Instrument Information:

Instrument Name: DE34903611
Firmware: C.01.069
Serial #: DE34903611
Type: G2938C

Assay Information:

Assay Origin Path: C:\Program Files\Agilent\2100 bioanalyzer\2100 expert\assays\dsDNA\DNA 1000 Series II.xsy

Assay Class: DNA 1000

Version: 2.3

Assay Comments: DNA Analysis 2 - 1000 bp

© Copyright 2003-2009 Agilent Technologies, Inc.

Chip Information:

Chip Lot #:

Reagent Kit Lot #:

Chip Comments:

Sample #1

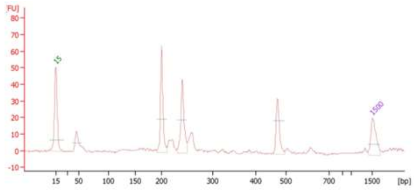

Sample 2

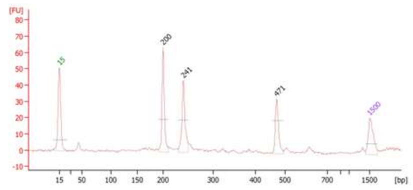

Sample 3

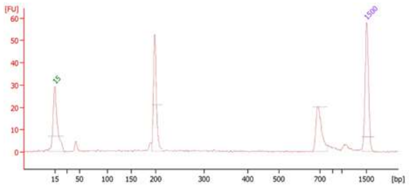

Sample #4

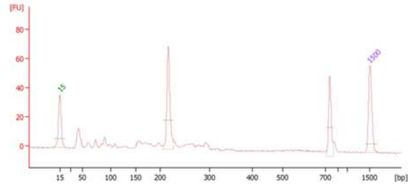

Sample 5

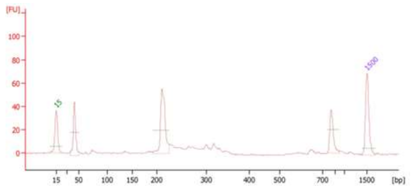

Sample 6

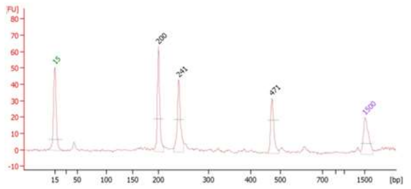

Sample #7

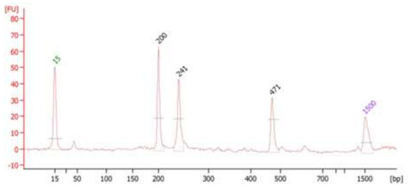

Sample 8

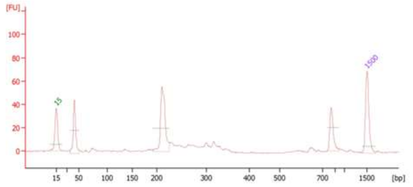

Sample 9

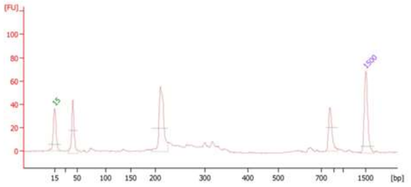

Sample #10

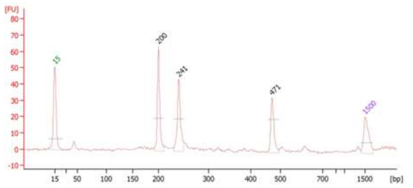

CTRL

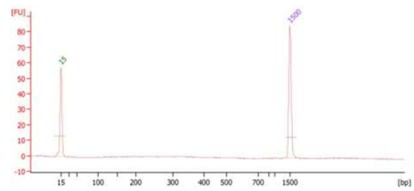

CTRL

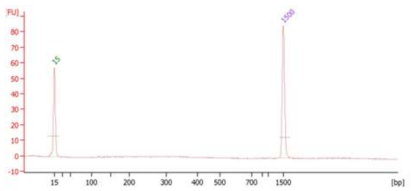

Assay Class: DNA 1000
Data Path: C:\...-30\2100 expert_DNA 1000_DE34903611_2023-03-06_TRN_AluI_1_10.xad
Created: 3/07/2023 9:17:20 PM
Modified: 3/07/2023 9:17:20 PM

Electrophoresis File Run Summary

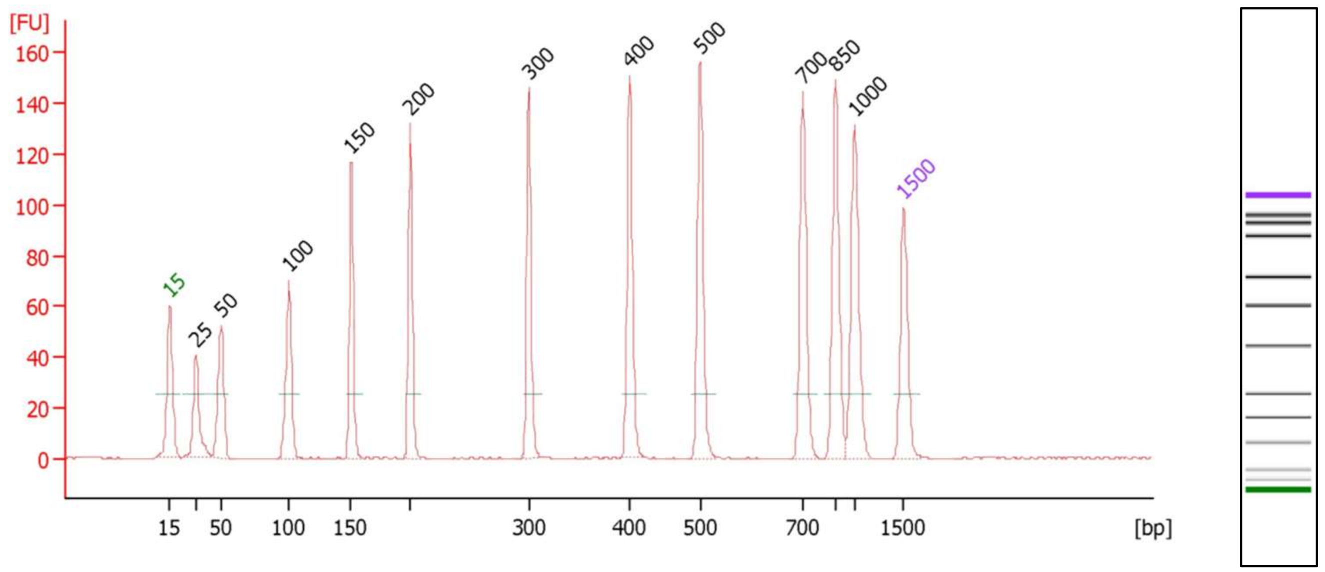

Peak table for Ladder :

Peak		Size [bp]	Conc. [ng/µl]	Molarity [nmol/l]	Observations
1		15	4.20	4.4.2	Lower Marker
2		25	4.00	242.4	Ladder Peak
3		50	4.00	121.2	Ladder Peak
4		100	4.00	60.6	Ladder Peak
5		150	4.00	40.3	Ladder Peak
6		200	4.00	30.3	Ladder Peak
7		300	4.00	20.2	Ladder Peak
8		400	4.00	15.2	Ladder Peak
9		500	4.00	12.1	Ladder Peak
10		700	4.00	8.7	Ladder Peak
11		850	4.00	7.1	Ladder Peak
12		1,000	4.00	6.1	Ladder Peak
13		1,500	2.10	2.1	Upper Marker

© Copyright 2003 - 2009 Agilent Technologies, Inc.

Assay Class: DNA 1000 Created: 3/07/2023 9:17:20 PM
Data Path: C:\...-30\2100 expert_DNA 1000_DE34903611_2023-03-06_TRN_AluI_1_10.xad Modified: 3/07/2023 9:17:20 PM

Electrophoresis File Run Summary

Sample #1

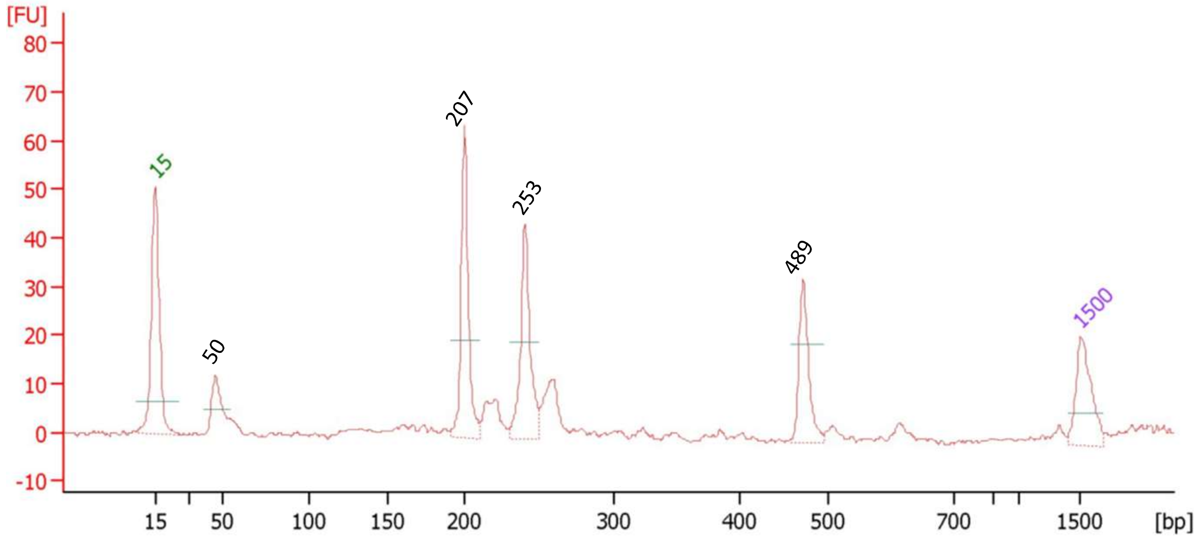

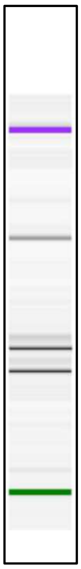

Peak table for Sample #1 :

Peak		Size [bp]	Conc. [ng/µl]	Molarity [nmol/l]	Observations
1	◀	15	4.20	424.4	Lower Marker
2	L	50	0.63	32.5	Ladder Peak
3	L	207	4.93	27.3	Ladder Peak
4	L	253	3.25	7.6	Ladder Peak
5	L	489	2.07	8.4	Ladder Peak
6	▶	1,500	2.10	2.2	Upper Marker

 © Copyright 2003 - 2009 Agilent Technologies, Inc.

Assay Class: DNA 1000
Data Path: C:\...-30\2100 expert_DNA 1000_DE34903611_2023-03-06_TRN_AluI_1_10.xad
Created: 3/07/2023 9:17:20 PM
Modified: 3/07/2023 9:17:20 PM

Electrophoresis File Run Summary

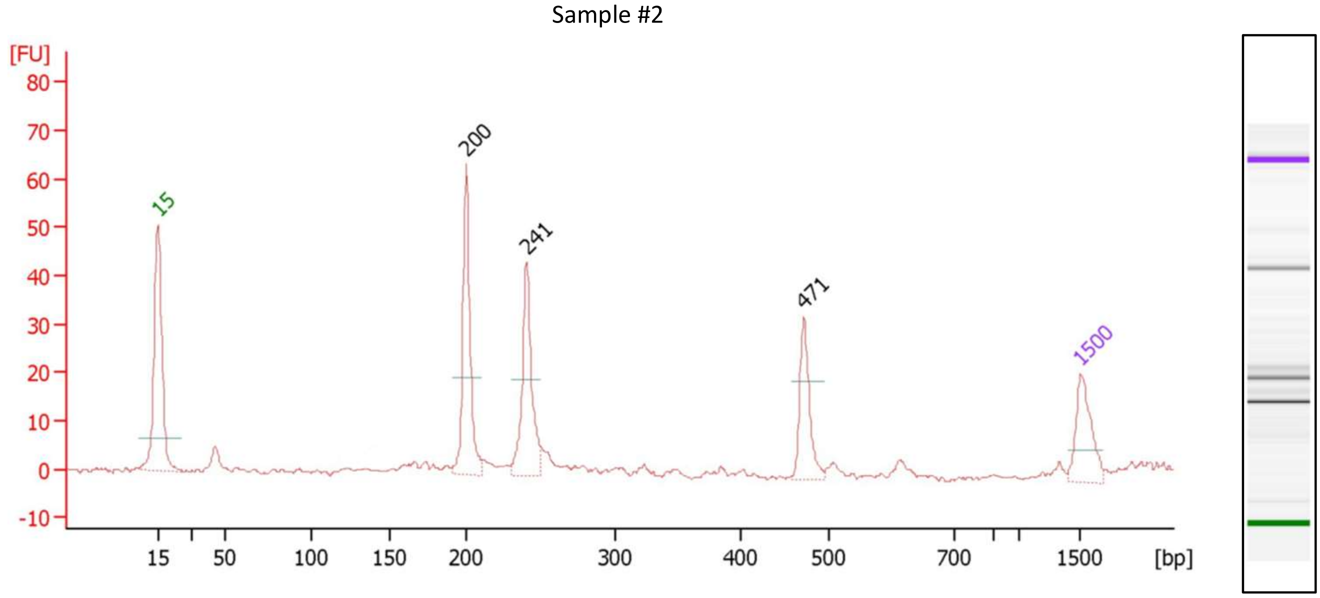

Peak table for Sample #2 :

Peak		Size [bp]	Conc. [ng/µl]	Molarity [nmol/l]	Observations
1	◀	15	4.20	424.4	Lower Marker
2	L	50	0.53	31.4	Ladder Peak
3	L	200	4.90	26.9	Ladder Peak
4	L	241	3.20	7.1	Ladder Peak
5	L	471	2.05	8.1	Ladder Peak
6	▶	1,500	2.10	2.2	Upper Marker

© Copyright 2003 - 2009 Agilent Technologies, Inc.

Assay Class: DNA 1000
Data Path: C:\...-30\2100 expert_DNA 1000_DE34903611_2023-03-06_TRN_AluI_1_10.xad
Created: 3/07/2023 9:17:20 PM
Modified: 3/07/2023 9:17:20 PM

Electrophoresis File Run Summary

Sample #3

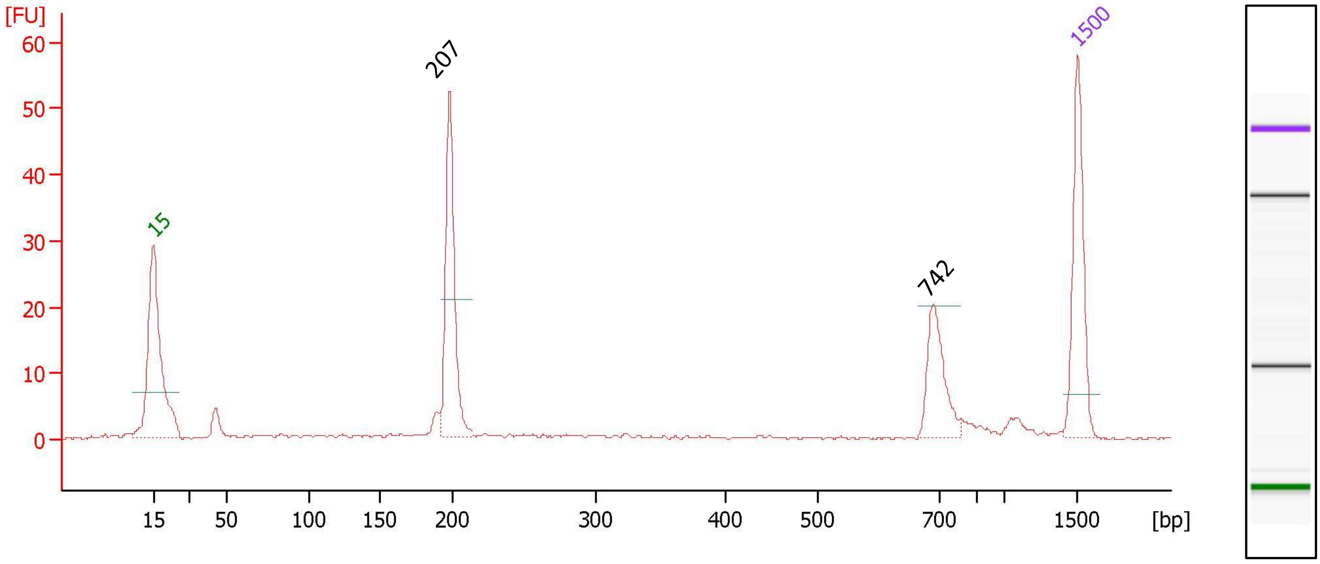

Peak table for Sample #3 :

Peak		Size [bp]	Conc. [ng/µl]	Molarity [nmol/l]	Observations
1	◀	15	4.20	424.4	Lower Marker
2	L	207	2.10	26.9	Ladder Peak
3	L	741	2.15	7.1	Ladder Peak
4	▶	1,500	2.10	2.2	Upper Marker

Assay Class: DNA 1000
Data Path: C:\...-30\2100 expert_DNA 1000_DE34903611_2023-03-06_TRN_AluI_1_10.xad
Created: 3/07/2023 9:17:20 PM
Modified: 3/07/2023 9:17:20 PM

Electrophoresis File Run Summary

Sample #4

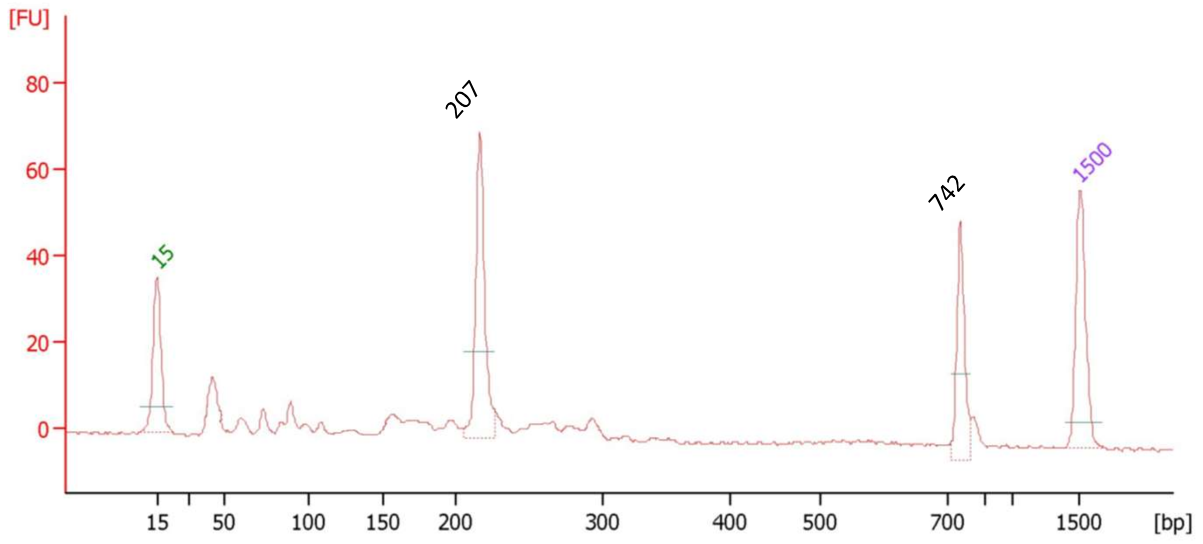

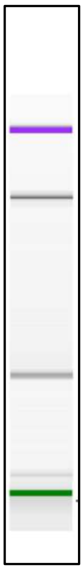

Peak table for Sample #4 :

Peak		Size [bp]	Conc. [ng/µl]	Molarity [nmol/l]	Observations
1		15	4.20	424.4	Lower Marker
2		50	1.00	1.23	Ladder Peak
3		207	2.80	16.9	Ladder Peak
4		742	2.95	17.1	Ladder Peak
5		1,500	2.10	2.2	Upper Marker

 © Copyright 2003 - 2009 Agilent Technologies, Inc.

Assay Class: DNA 1000
Data Path: C:\...-30\2100 expert_DNA 1000_DE34903611_2023-03-06_TRN_AluI_1_10.xad

Created: 3/07/2023 9:17:20 PM
Modified: 3/07/2023 9:17:20 PM

Electrophoresis File Run Summary

Sample #5

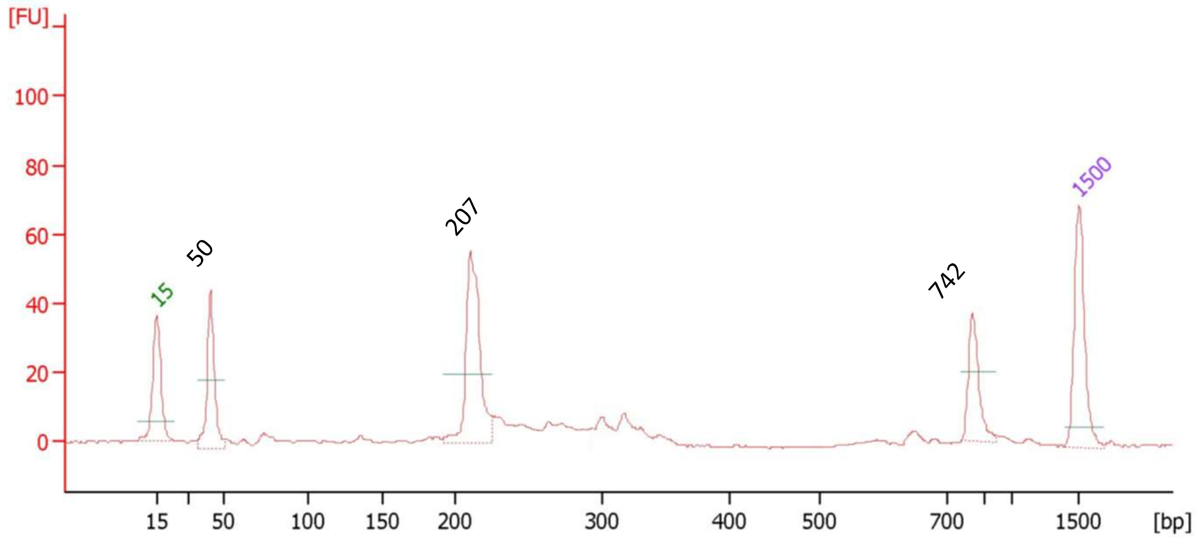

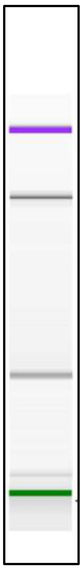

Peak table for Sample #5 :

Peak		Size [bp]	Conc. [ng/µl]	Molarity [nmol/l]	Observations
1	◀	15	4.20	424.4	Lower Marker
2	L	50	2.00	3.3	Ladder Peak
3	L	207	1.45	11.3	Ladder Peak
4	L	742	2.35	7.1	Ladder Peak
5	▶	1,500	2.10	2.2	Upper Marker

 © Copyright 2003 - 2009 Agilent Technologies, Inc.

Assay Class: DNA 1000
Data Path: C:\...-30\2100 expert_DNA 1000_DE34903611_2023-03-06_TRN_AluI_1_10.xad
Created: 3/07/2023 9:17:20 PM
Modified: 3/07/2023 9:17:20 PM

Electrophoresis File Run Summary

Sample #6

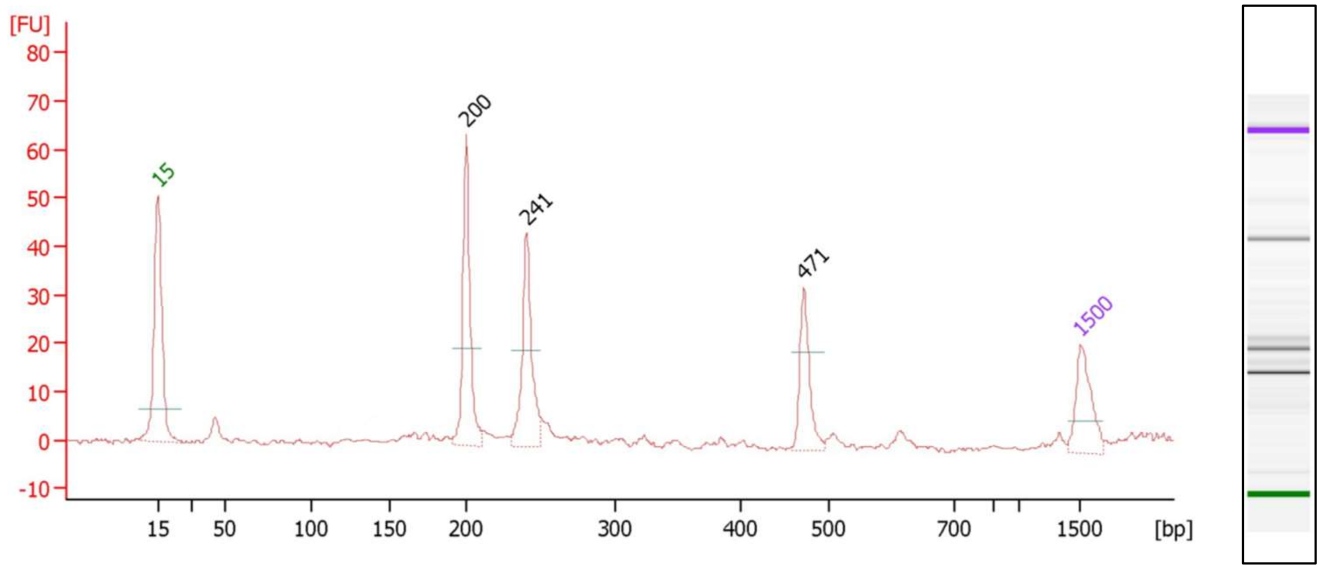

Peak table for Sample #6 :

Peak		Size [bp]	Conc. [ng/µl]	Molarity [nmol/l]	Observations
1	◀	15	4.20	424.4	Lower Marker
2	L	50	0.53	31.4	Ladder Peak
3	L	200	4.90	26.9	Ladder Peak
4	L	241	3.20	7.1	Ladder Peak
5	L	471	2.05	8.1	Ladder Peak
6	▶	1,500	2.10	2.2	Upper Marker

 © Copyright 2003 - 2009 Agilent Technologies, Inc.

Assay Class: DNA 1000
Data Path: C:\...-30\2100 expert_DNA 1000_DE34903611_2023-03-06_TRN_AluI_1_10.xad
Created: 3/07/2023 9:17:20 PM
Modified: 3/07/2023 9:17:20 PM

Electrophoresis File Run Summary

Sample #7

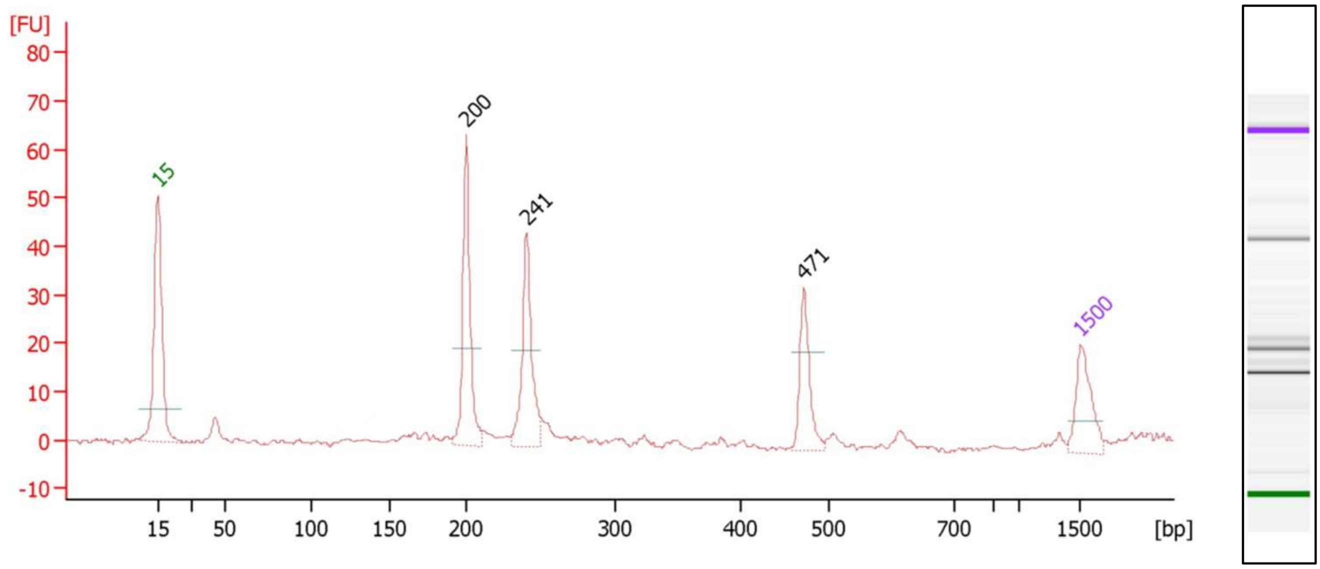

Peak table for Sample #7 :

Peak		Size [bp]	Conc. [ng/µl]	Molarity [nmol/l]	Observations
1	◀	15	4.20	424.4	Lower Marker
2	L	50	0.53	31.4	Ladder Peak
3	L	200	4.90	26.9	Ladder Peak
4	L	241	3.20	7.1	Ladder Peak
5	L	471	2.05	8.1	Ladder Peak
6	▶	1,500	2.10	2.2	Upper Marker

© Copyright 2003 - 2009 Agilent Technologies, Inc.

Assay Class: DNA 1000
Data Path: C:\...-30\2100 expert_DNA 1000_DE34903611_2023-03-06_TRN_AluI_1_10.xad
Created: 3/07/2023 9:17:20 PM
Modified: 3/07/2023 9:17:20 PM

Electrophoresis File Run Summary

Sample #8

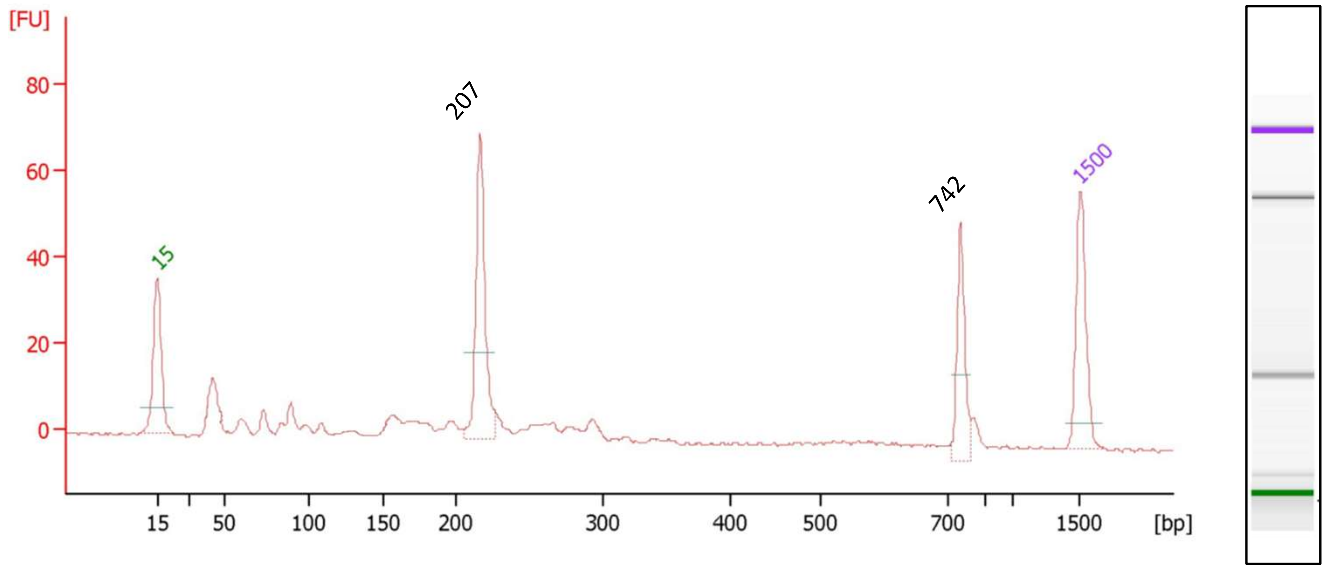

Peak table for Sample #8 :

Peak		Size [bp]	Conc. [ng/µl]	Molarity [nmol/l]	Observations
1		15	4.20	424.4	Lower Marker
2		50	1.00	1.23	Ladder Peak
3		207	2.80	16.9	Ladder Peak
4		742	2.95	17.1	Ladder Peak
5		1,500	2.10	2.2	Upper Marker

 © Copyright 2003 - 2009 Agilent Technologies, Inc.

Assay Class: DNA 1000
Data Path: C:\...-30\2100 expert_DNA 1000_DE34903611_2023-03-06_TRN_AluI_1_10.xad
Created: 3/07/2023 9:17:20 PM
Modified: 3/07/2023 9:17:20 PM

Electrophoresis File Run Summary

Sample #9

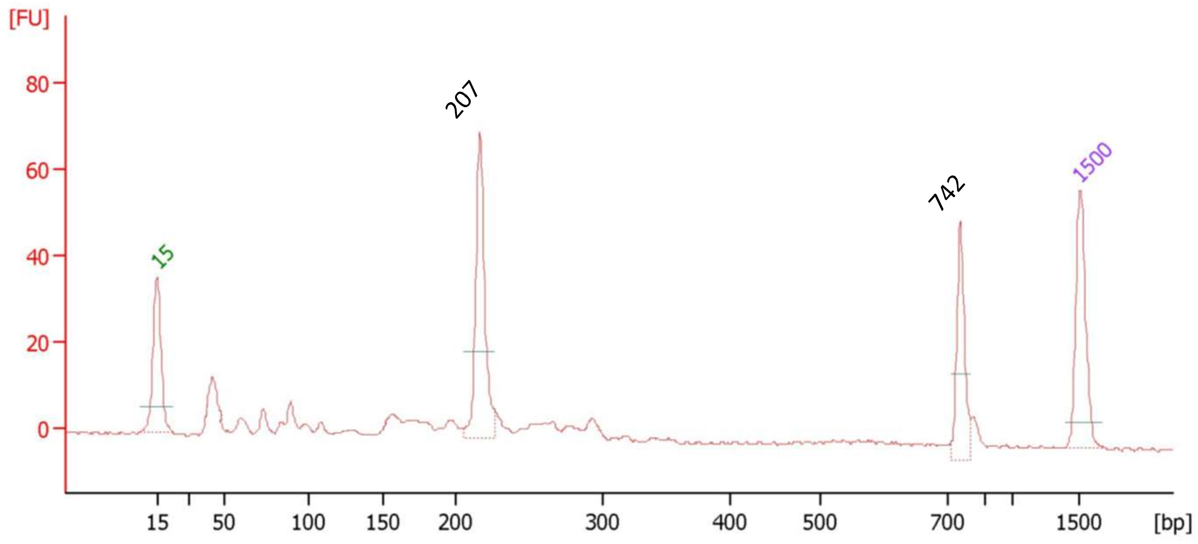

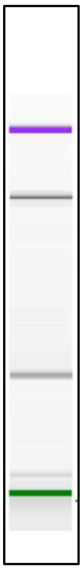

Peak table for Sample #9 :

Peak		Size [bp]	Conc. [ng/µl]	Molarity [nmol/l]	Observations
1	◀	15	4.20	424.4	Lower Marker
2	L	50	1.00	1.23	Ladder Peak
3	L	207	2.80	16.9	Ladder Peak
4	L	742	2.95	17.1	Ladder Peak
5	▶	1,500	2.10	2.2	Upper Marker

 © Copyright 2003 - 2009 Agilent Technologies, Inc.

Assay Class: DNA 1000
Data Path: C:\...-30\2100 expert_DNA 1000_DE34903611_2023-03-06_TRN_AluI_1_10.xad
Created: 3/07/2023 9:17:20 PM
Modified: 3/07/2023 9:17:20 PM

Electrophoresis File Run Summary

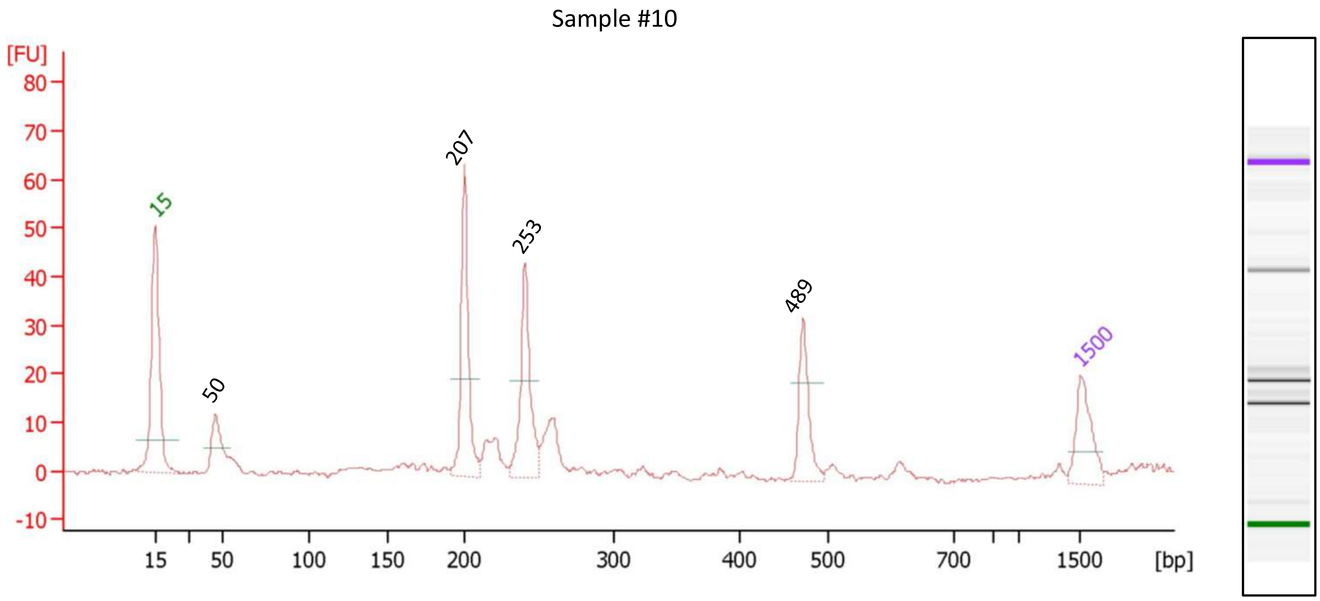

Peak table for Sample #10 :

Peak		Size [bp]	Conc. [ng/µl]	Molarity [nmol/l]	Observations
1	◀	15	4.20	424.4	Lower Marker
2	L	50	0.63	32.5	Ladder Peak
3	L	207	4.93	27.3	Ladder Peak
4	L	253	3.25	7.6	Ladder Peak
5	L	489	2.07	8.4	Ladder Peak
6	▶	1,500	2.10	2.2	Upper Marker

 © Copyright 2003 - 2009 Agilent Technologies, Inc.

Assay Class: DNA 1000
Data Path: C:\...-30\2100 expert_DNA 1000_DE34903611_2023-03-06_TRN_AluI_1_10.xad
Created: 3/07/2023 9:17:20 PM
Modified: 3/07/2023 9:17:20 PM

Electrophoresis File Run Summary

CTRL

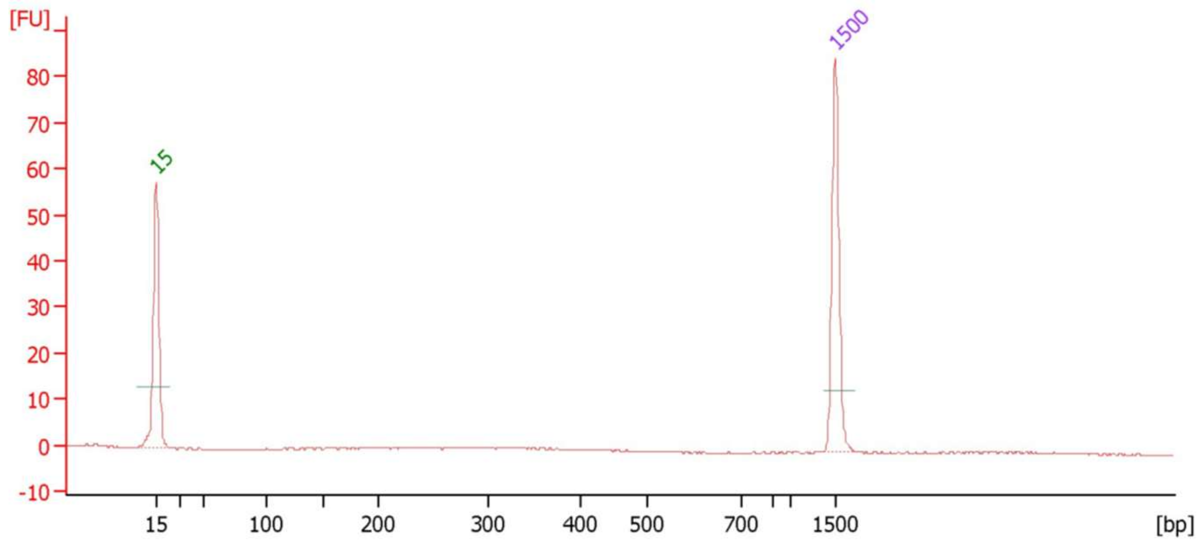

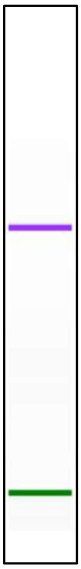

Peak table for Sample CTRL :

Peak		Size [bp]	Conc. [ng/µl]	Molarity [nmol/l]	Observations
1	◀	15	4.20	424.4	Lower Marker
2	▶	1,500	2.10	2.2	Upper Marker

Assay Class: DNA 1000
Data Path: C:\...-30\2100 expert_DNA 1000_DE34903611_2023-03-06_TRN_AluI_1_10.xad

Created: 3/07/2023 9:17:20 PM
Modified: 3/07/2023 9:17:20 PM

Electrophoresis File Run Summary

CTRL

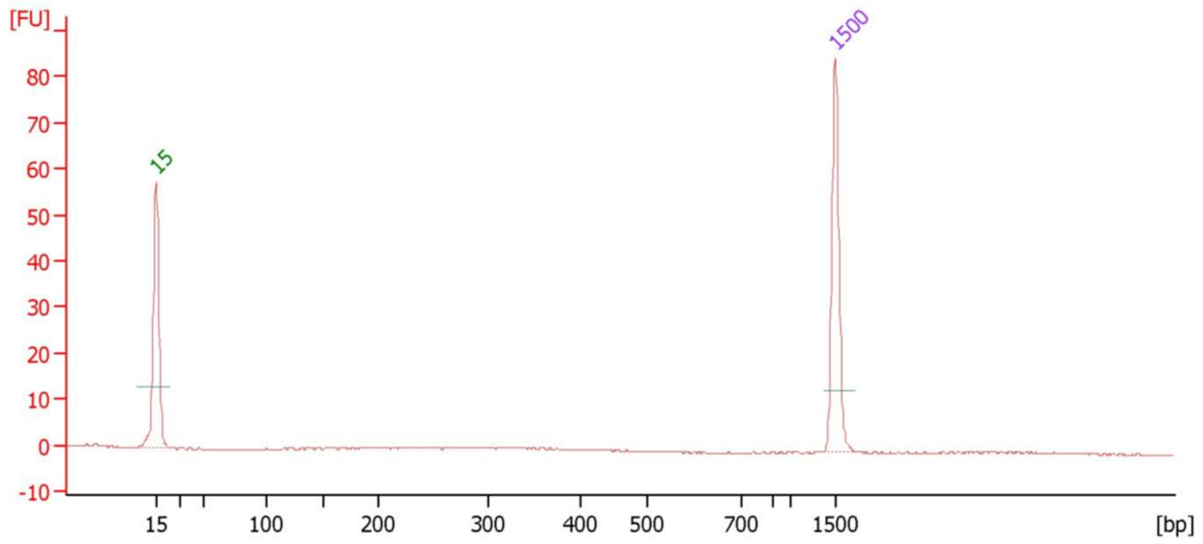

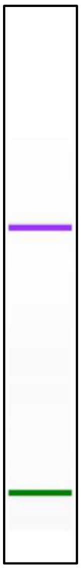

Peak table for Sample CTRL :

Peak		Size [bp]	Conc. [ng/µl]	Molarity [nmol/l]	Observations
1	◀	15	4.20	424.4	Lower Marker
2	▶	1,500	2.10	2.1	Upper Marker

 © Copyright 2003 - 2009 Agilent Technologies, Inc.

Assay Class: DNA 1000
Data Path: C:\...-30\2100 expert_DNA 1000_DE34903611_2023-03-06_TRN_AluI_1_10.xad
Created: 3/07/2023 9:17:20 PM
Modified: 3/07/2023 9:17:20 PM

Curves

Standard Curve

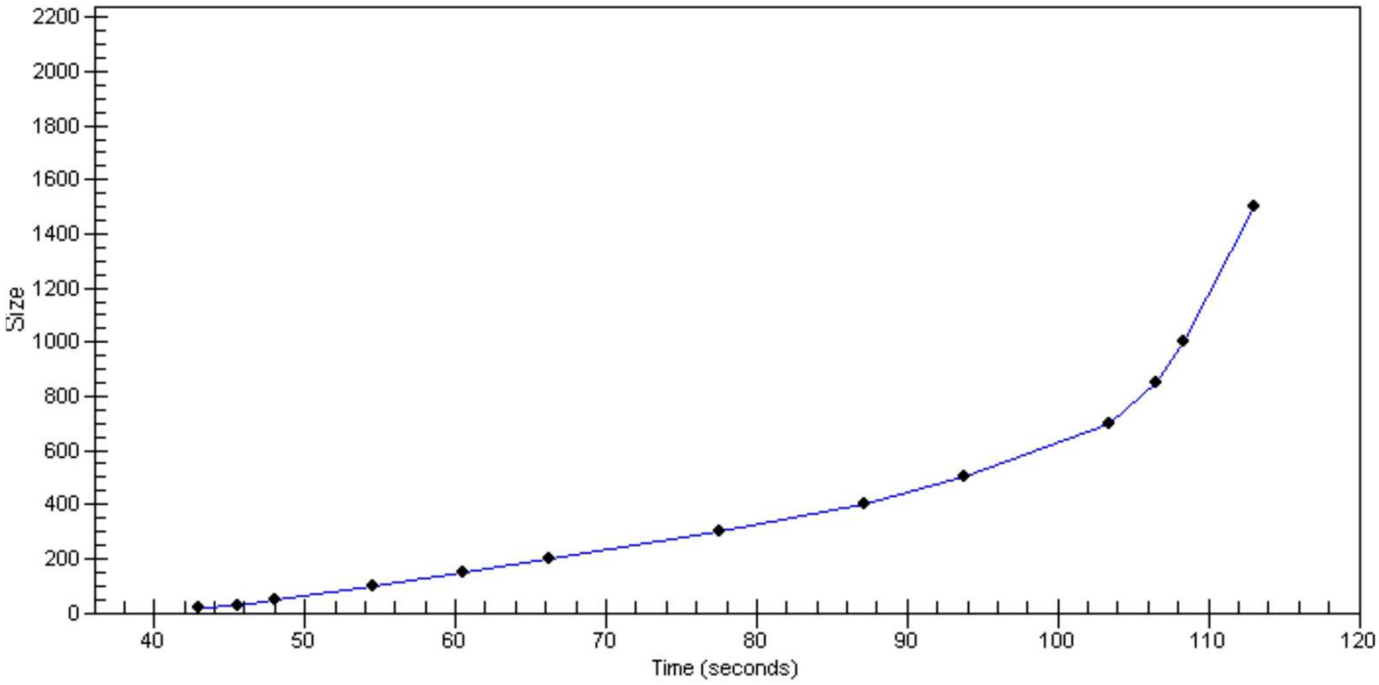

© Copyright 2003 - 2009 Agilent Technologies, Inc.

Assay Class: DNA 1000
Data Path: C:\...-30\2100 expert_DNA 1000_DE34903611_2023-03-06_TRN_AluI_1_10.xad
Created: 3/07/2023 9:17:20 PM
Modified: 3/07/2023 9:17:20 PM

Electrophoresis File Run Summary (Chip Summary)

Sample Name	Sample Content	Rest. Digest	Status	Observation	Result Label	Result Color
Sample #1		☐	✔			
Sample #2		☐	✔			
Sample #3		☐	✔			
Sample #4		☐	✔			
Sample #5		☐	✔			
Sample #6		☐	✔			
Sample #7		☐	✔			
Sample #8		☐	✔			
Sample #9		☐	✔			
Sample #10		☐	✔			
CTRL		☐	✔			
CTRL		☐	✔			

Chip Lot # **Reagent Kit Lot #**

Chip Comments :

 © Copyright 2003 - 2009 Agilent Technologies, Inc.

Assay Class: DNA 1000
Data Path: C:\...-30\2100 expert_DNA 1000_DE34903611_2023-03-06_TRN_AluI_1_10.xad
Created: 3/07/2023 9:17:20 PM
Modified: 3/07/2023 9:17:20 PM

Electrophoresis Assay Details

Result Label **Result Color**

General Analysis Settings

Number of Available Sample and Ladder Wells (Max.) : 13
Minimum Visible Range [s] : 30
Maximum Visible Range [s] : 129
Start Analysis Time Range [s] : 30
End Analysis Time Range [s] : 128.95
Ladder Concentration [ng/µl] : 44
Uses Standard Area for Ladder Fragments
Lower Marker Concentration [ng/µl] : 4.2
Upper Marker Concentration [ng/µl] : 2.1
Used Upper Marker for Quantitation
Standard Curve Fit is Point to Point
Show Data Aligned to Lower and Upper Marker

Integrator Settings

Integration Start Time [s] : 30
Integration End Time [s] : 128.95
Slope Threshold : 0.5
Height Threshold [FU] : 20
Area Threshold : 0.1
Width Threshold [s] : 0.5
Baseline Plateau [s] : 0.5

Filter Settings

Filter Width [s] : 0.5
Polynomial Order : 4

Ladder

Ladder Peak	Size	Area
1	15	25
2	25	26
3	50	34
4	100	41
5	150	45
6	200	52
7	300	63
8	400	76
9	500	83
10	700	88
11	850	86
12	1000	90
13	1500	52

© Copyright 2003 - 2009 Agilent Technologies, Inc.

Assay Class: DNA 1000
Data Path: C:\...-30\2100 expert_DNA 1000_DE34903611_2023-03-06_TRN_AluI_11_20.xad
Created: 3/08/2023 10:21:55 PM
Modified: 3/08/2023 10:21:55 PM

Electrophoresis File Run Summary

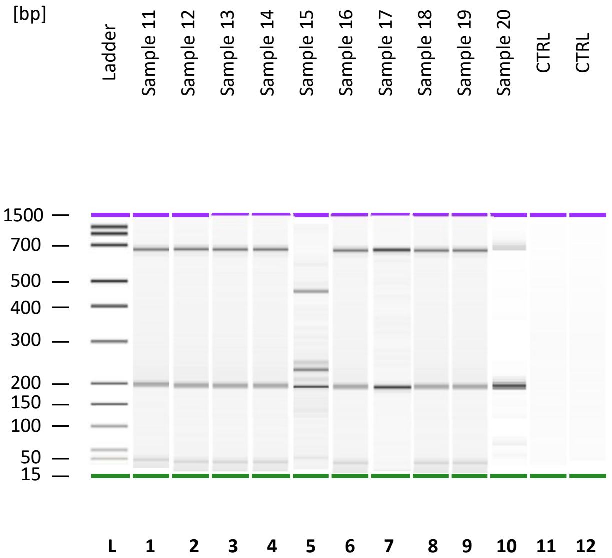

Instrument Information:

Instrument Name: DE34903611 Firmware: C.01.069
Serial #: DE34903611 Type: G2938C

Assay Information:

Assay Origin Path: C:\Program Files\Agilent\2100 bioanalyzer\2100 expert\assays\dsDNA\DNA 1000 Series II.xsy

Assay Class: DNA 1000

Version: 2.3

Assay Comments: DNA Analysis 2 - 1000 bp

© Copyright 2003-2009 Agilent Technologies, Inc.

Chip Information:

Chip Lot #:

Reagent Kit Lot #:

Chip Comments:

Sample #11

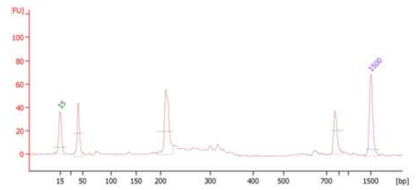

Sample 12

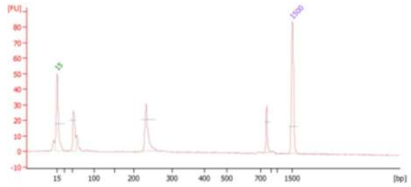

Sample 13

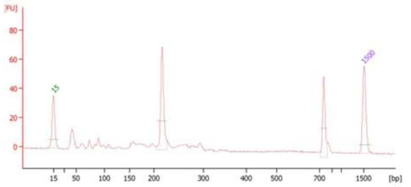

Sample #14

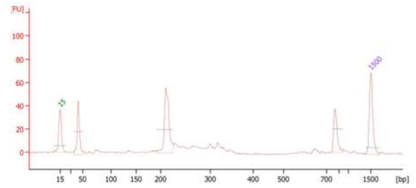

Sample 15

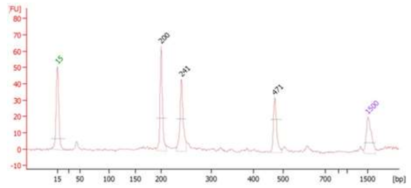

Sample 16

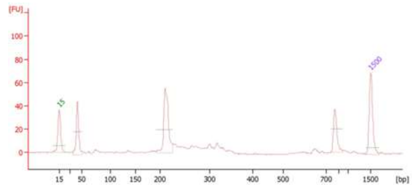

Sample #17

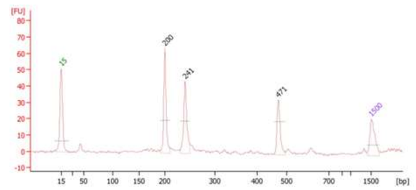

Sample 18

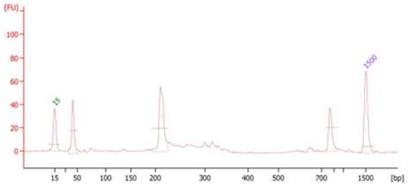

Sample 19

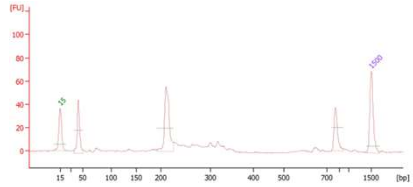

Sample #20

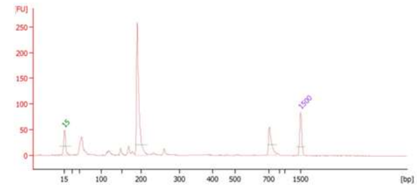

CTRL

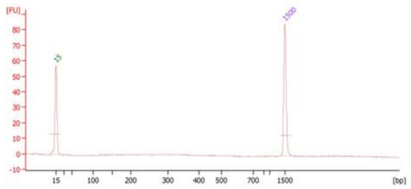

CTRL

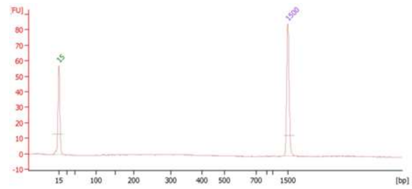

Assay Class: DNA 1000
Data Path: C:\...-30\2100 expert_DNA 1000_DE34903611_2023-03-06_TRN_AluI_11_20.xad

Created: 3/08/2023 10:21:55 PM
Modified: 3/08/2023 10:21:55 PM

Electrophoresis File Run Summary

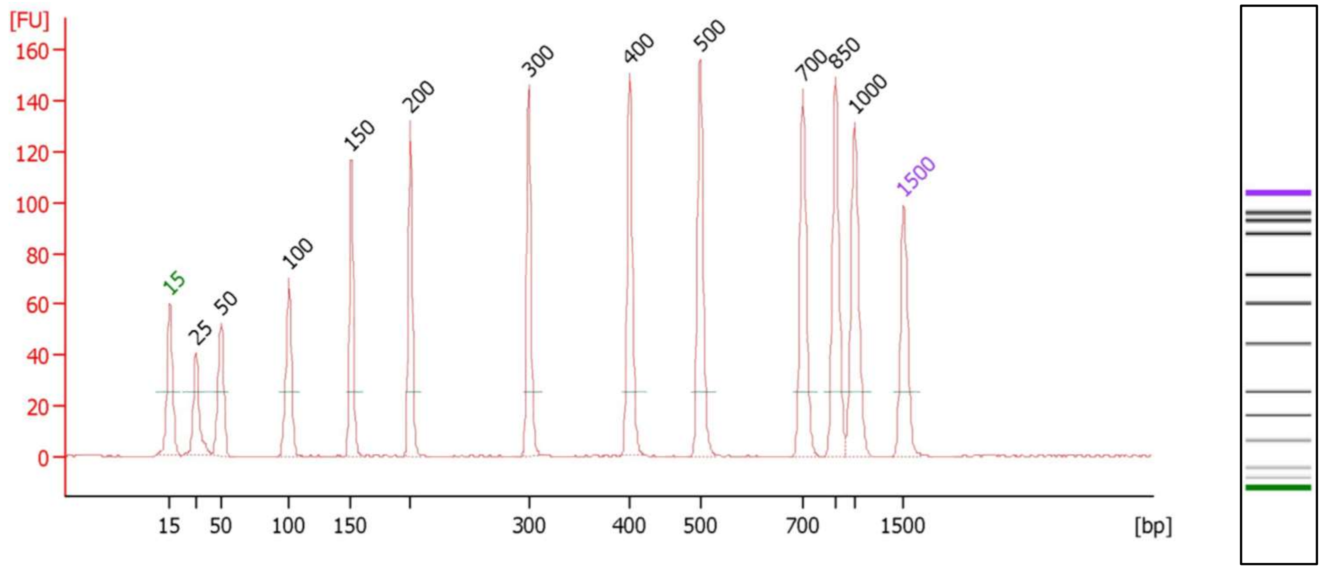

Peak table for Ladder :

Peak		Size [bp]	Conc. [ng/µl]	Molarity [nmol/l]	Observations
1	◀	15	4.20	4.4.2	Lower Marker
2	L	25	4.00	242.4	Ladder Peak
3	L	50	4.00	121.2	Ladder Peak
4	L	100	4.00	60.6	Ladder Peak
5	L	150	4.00	40.3	Ladder Peak
6	L	200	4.00	30.3	Ladder Peak
7	L	300	4.00	20.2	Ladder Peak
8	L	400	4.00	15.2	Ladder Peak
9	L	500	4.00	12.1	Ladder Peak
10	L	700	4.00	8.7	Ladder Peak
11	L	850	4.00	7.1	Ladder Peak
12	L	1,000	4.00	6.1	Ladder Peak
13	▶	1,500	2.10	2.1	Upper Marker

 © Copyright 2003 - 2009 Agilent Technologies, Inc.

Assay Class: DNA 1000
Data Path: C:\...-30\2100 expert_DNA 1000_DE34903611_2023-03-06_TRN_AluI_11_20.xad
Created: 3/08/2023 10:21:55 PM
Modified: 3/08/2023 10:21:55 PM

Electrophoresis File Run Summary

Sample #11

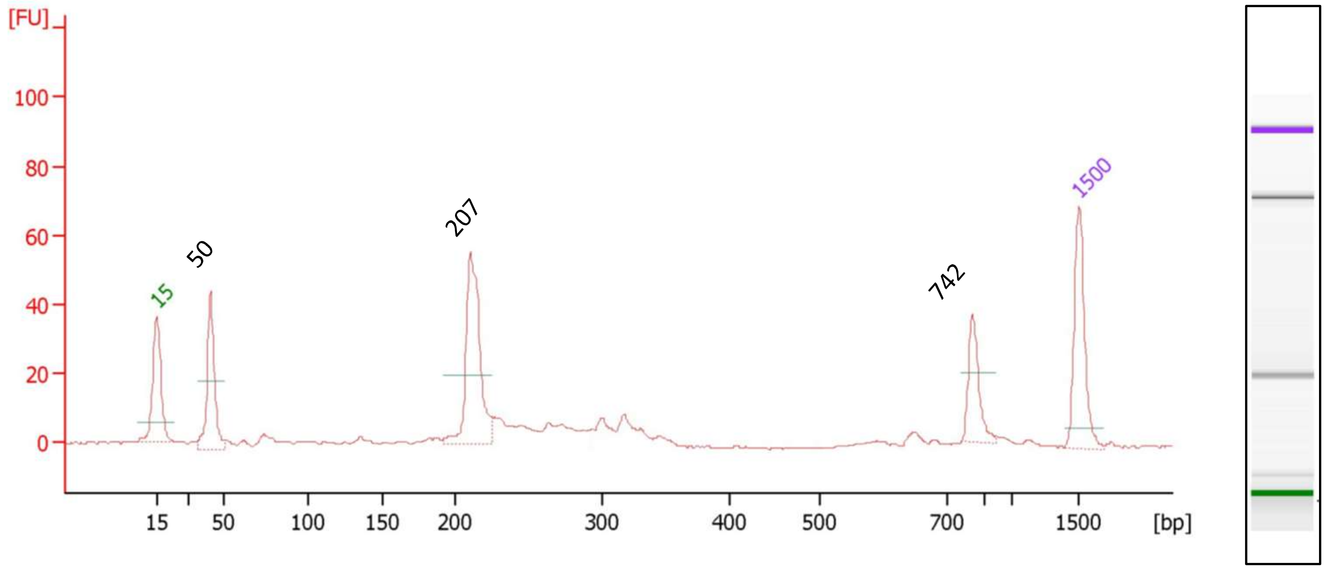

Peak table for Sample #11 :

Peak		Size [bp]	Conc. [ng/µl]	Molarity [nmol/l]	Observations
1	◀	15	4.20	424.4	Lower Marker
2	L	50	2.00	2.3	Ladder Peak
3	L	207	4.45	9.3	Ladder Peak
4	L	742	2.35	6.3	Ladder Peak
5	▶	1,500	2.10	2.2	Upper Marker

 © Copyright 2003 - 2009 Agilent Technologies, Inc.

Assay Class: DNA 1000
Data Path: C:\...-30\2100 expert_DNA 1000_DE34903611_2023-03-06_TRN_AluI_11_20.xad
Created: 3/08/2023 10:21:55 PM
Modified: 3/08/2023 10:21:55 PM

Electrophoresis File Run Summary

Sample #12

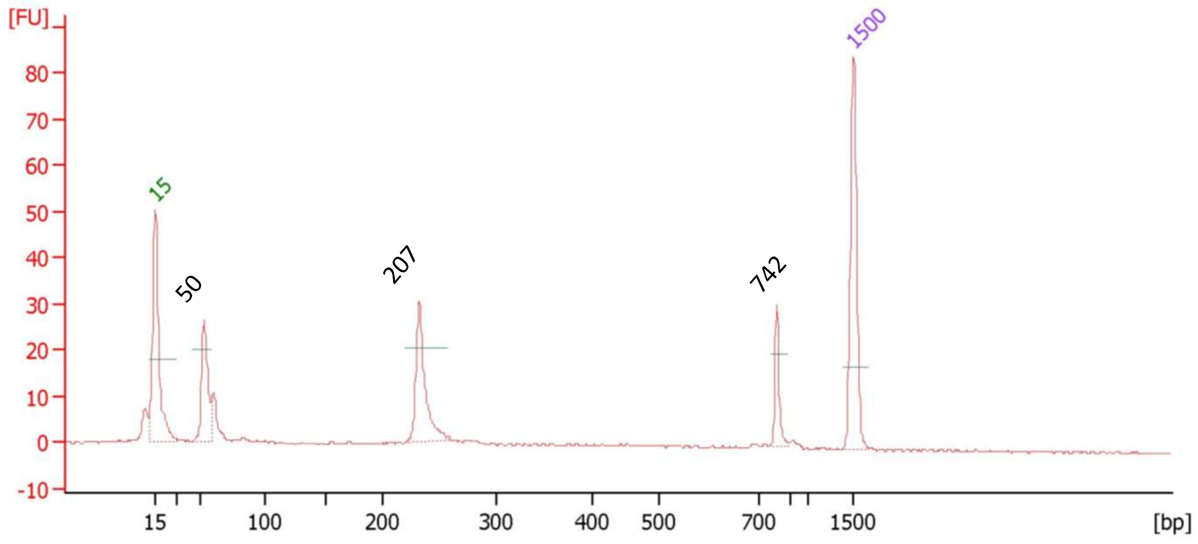

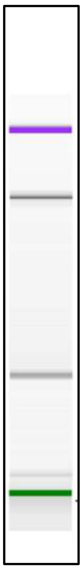

Peak table for Sample #12 :

Peak		Size [bp]	Conc. [ng/µl]	Molarity [nmol/l]	Observations
1	◄	15	4.20	424.4	Lower Marker
2	L	50	2.00	1.1	Ladder Peak
3	L	207	1.45	4.2	Ladder Peak
4	L	742	2.35	3.1	Ladder Peak
5	►	1,500	2.10	2.2	Upper Marker

 © Copyright 2003 - 2009 Agilent Technologies, Inc.

Assay Class: DNA 1000
Data Path: C:\...-30\2100 expert_DNA 1000_DE34903611_2023-03-06_TRN_AluI_11_20.xad

Created: 3/08/2023 10:21:55 PM
Modified: 3/08/2023 10:21:55 PM

Electrophoresis File Run Summary

Sample #13

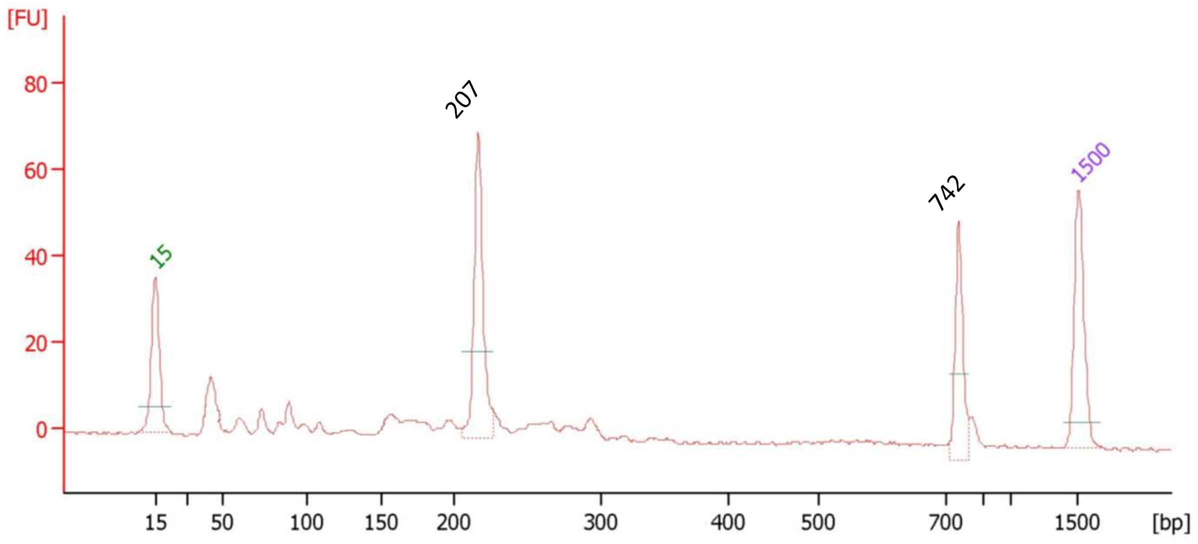

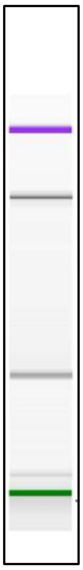

Peak table for Sample #13 :

Peak		Size [bp]	Conc. [ng/µl]	Molarity [nmol/l]	Observations
1	◀	15	4.20	424.4	Lower Marker
2	L	50	1.00	1.13	Ladder Peak
3	L	207	2.95	18.0	Ladder Peak
4	L	742	3.01	16.1	Ladder Peak
5	▶	1,500	2.10	2.2	Upper Marker

© Copyright 2003 - 2009 Agilent Technologies, Inc.

Assay Class: DNA 1000
Data Path: C:\...-30\2100 expert_DNA 1000_DE34903611_2023-03-06_TRN_AluI_11_20.xad
Created: 3/08/2023 10:21:55 PM
Modified: 3/08/2023 10:21:55 PM

Electrophoresis File Run Summary

Sample #14

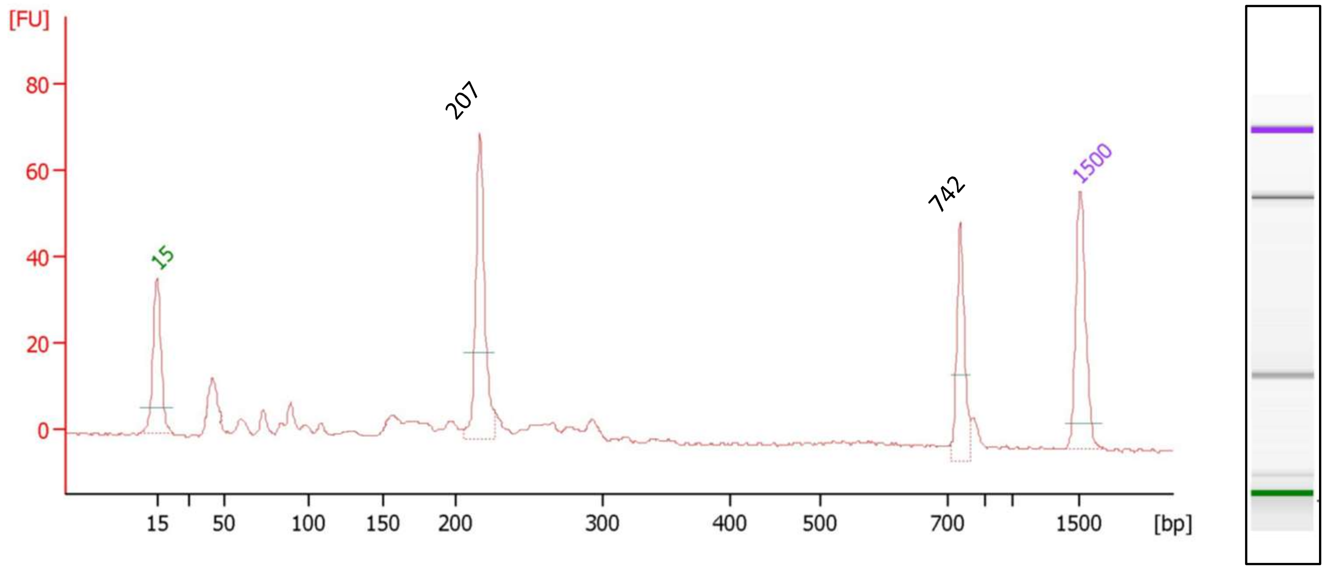

Peak table for Sample #14 :

Peak		Size [bp]	Conc. [ng/µl]	Molarity [nmol/l]	Observations
1		15	4.20	424.4	Lower Marker
2		50	1.00	1.23	Ladder Peak
3		207	2.80	16.9	Ladder Peak
4		742	2.95	17.1	Ladder Peak
5		1,500	2.10	2.2	Upper Marker

Assay Class: DNA 1000
Data Path: C:\...-30\2100 expert_DNA 1000_DE34903611_2023-03-06_TRN_AluI_11_20.xad
Created: 3/08/2023 10:21:55 PM
Modified: 3/08/2023 10:21:55 PM

Electrophoresis File Run Summary

Sample #15

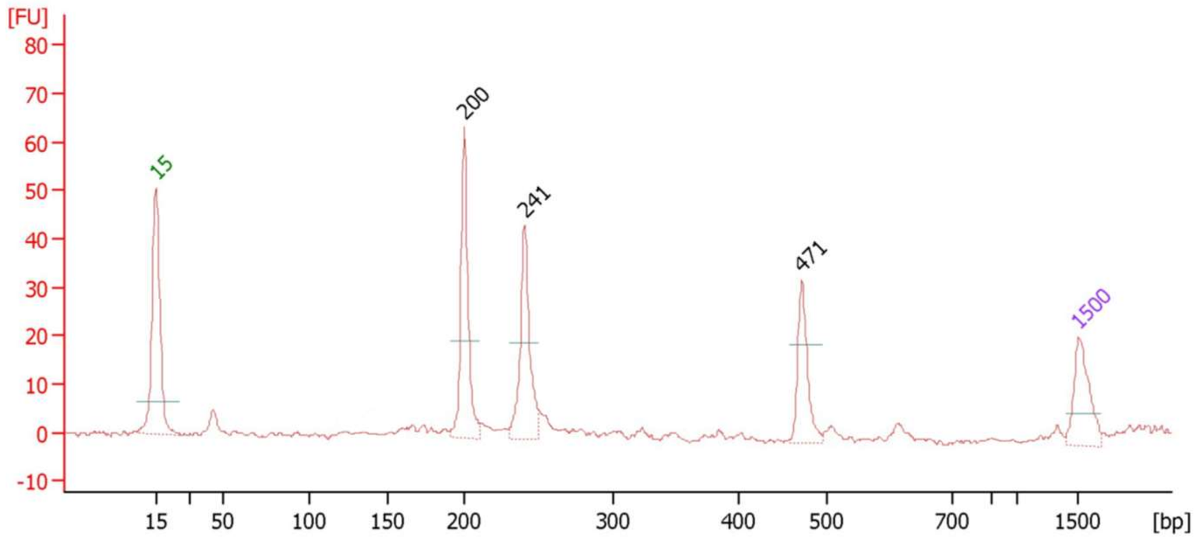

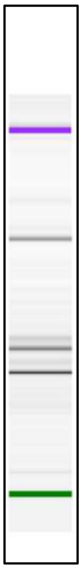

Peak table for Sample #15 :

Peak		Size [bp]	Conc. [ng/µl]	Molarity [nmol/l]	Observations
1	◀	15	4.20	424.4	Lower Marker
2	L	50	0.53	31.4	Ladder Peak
3	L	200	4.90	26.9	Ladder Peak
4	L	241	3.20	7.1	Ladder Peak
5	L	471	2.05	8.1	Ladder Peak
6	▶	1,500	2.10	2.2	Upper Marker

© Copyright 2003 - 2009 Agilent Technologies, Inc.

Assay Class: DNA 1000
Data Path: C:\...-30\2100 expert_DNA 1000_DE34903611_2023-03-06_TRN_AluI_11_20.xad
Created: 3/08/2023 10:21:55 PM
Modified: 3/08/2023 10:21:55 PM

Electrophoresis File Run Summary

Sample #16

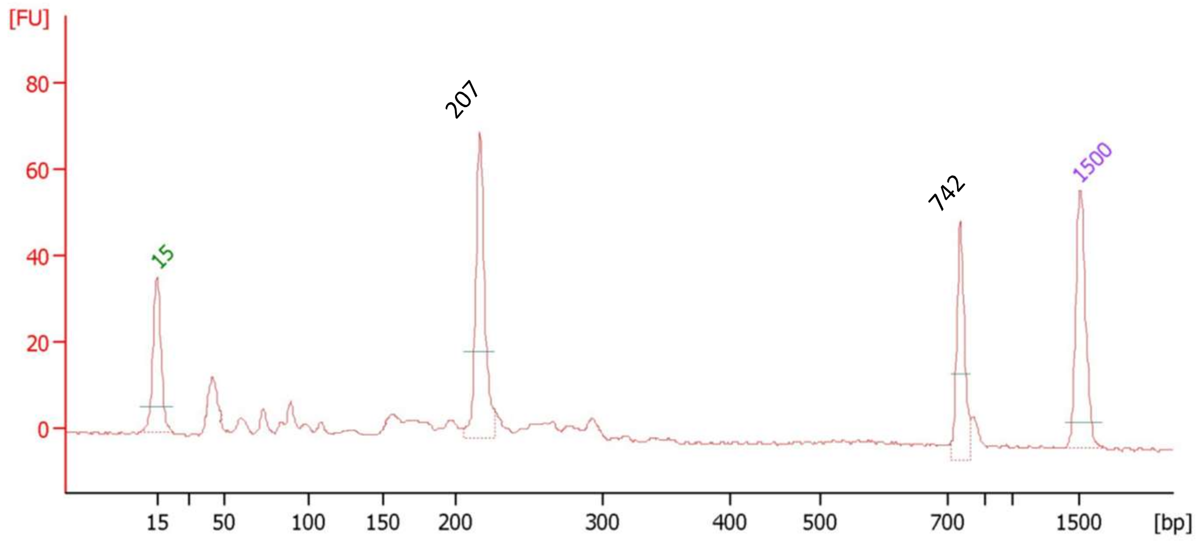

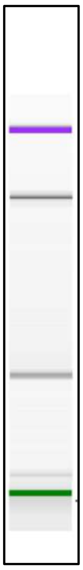

Peak table for Sample #16 :

Peak		Size [bp]	Conc. [ng/µl]	Molarity [nmol/l]	Observations
1	◀	15	4.20	424.4	Lower Marker
2	L	207	2.80	16.9	Ladder Peak
3	L	742	2.95	17.1	Ladder Peak
4	▶	1,500	2.10	2.2	Upper Marker

© Copyright 2003 - 2009 Agilent Technologies, Inc.

Assay Class: DNA 1000
Data Path: C:\...-30\2100 expert_DNA 1000_DE34903611_2023-03-06_TRN_AluI_11_20.xad
Created: 3/08/2023 10:21:55 PM
Modified: 3/08/2023 10:21:55 PM

Electrophoresis File Run Summary

Sample #17

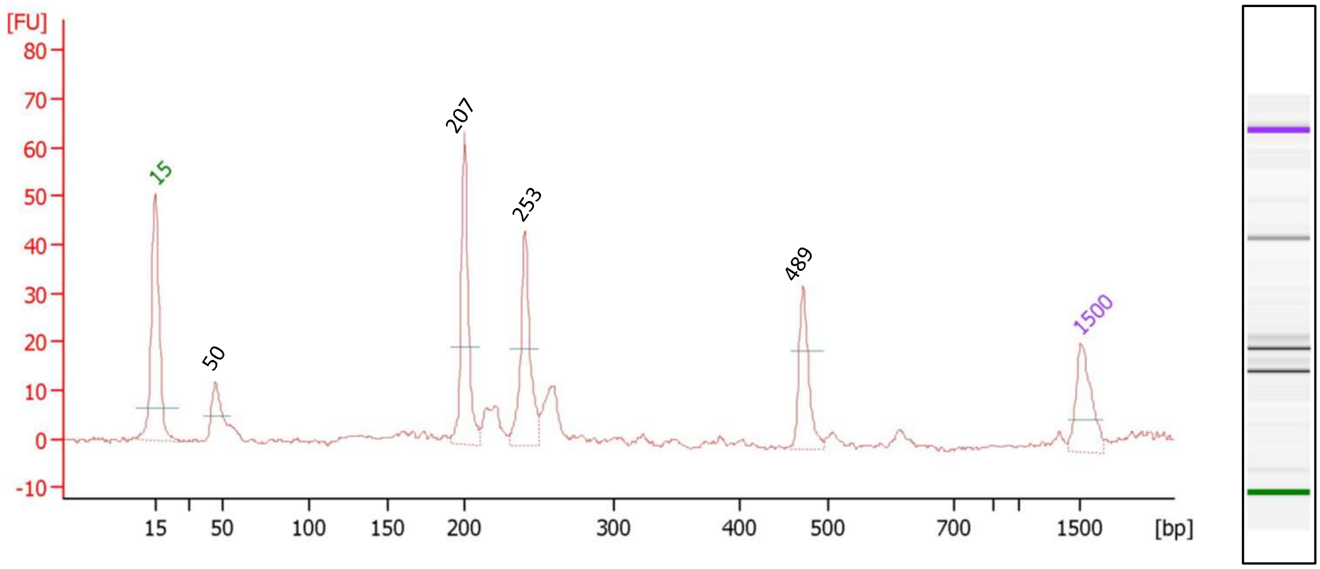

Peak table for Sample #17 :

Peak		Size [bp]	Conc. [ng/µl]	Molarity [nmol/l]	Observations
1	◀	15	4.20	424.4	Lower Marker
2	L	50	0.63	32.5	Ladder Peak
3	L	207	4.93	27.3	Ladder Peak
4	L	253	3.25	7.6	Ladder Peak
5	L	489	2.07	8.4	Ladder Peak
6	▶	1,500	2.10	2.2	Upper Marker

 © Copyright 2003 - 2009 Agilent Technologies, Inc.

Assay Class: DNA 1000 Created: 3/08/2023 10:21:55 PM
Data Path: C:\...-30\2100 expert_DNA 1000_DE34903611_2023-03-06_TRN_AluI_11_20.xad Modified: 3/08/2023 10:21:55 PM

Electrophoresis File Run Summary

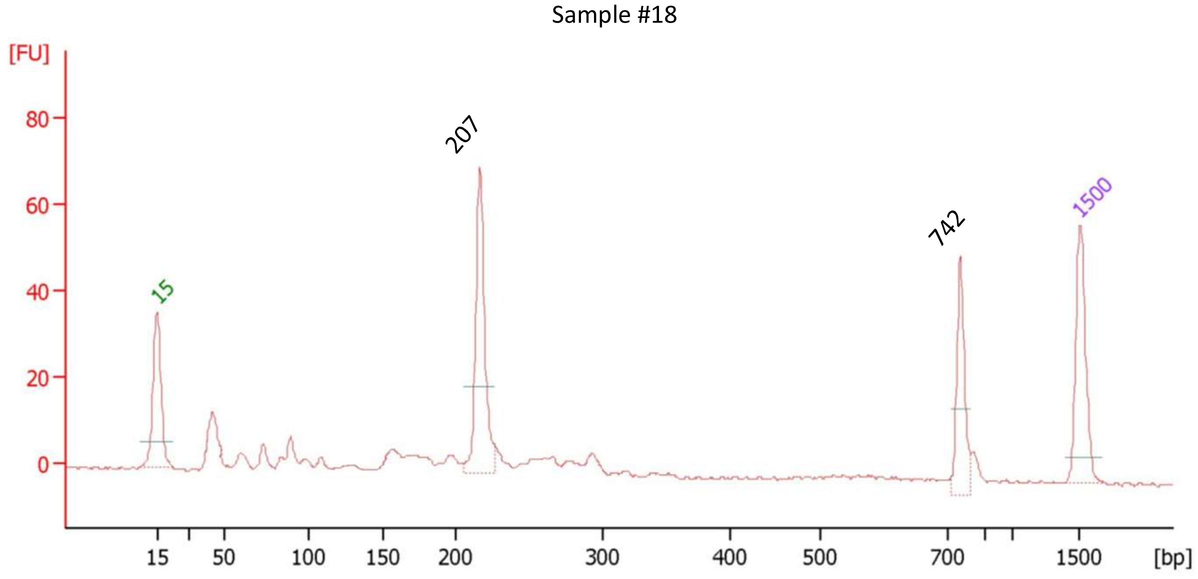

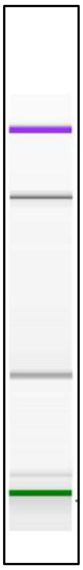

Peak table for Sample #18 :

Peak		Size [bp]	Conc. [ng/µl]	Molarity [nmol/l]	Observations
1	◀	15	4.20	424.4	Lower Marker
2	L	207	2.80	16.9	Ladder Peak
3	L	742	2.95	17.1	Ladder Peak
4	▶	1,500	2.10	2.2	Upper Marker

 © Copyright 2003 - 2009 Agilent Technologies, Inc.

Assay Class: DNA 1000
Data Path: C:\...-30\2100 expert_DNA 1000_DE34903611_2023-03-06_TRN_AluI_11_20.xad
Created: 3/08/2023 10:21:55 PM
Modified: 3/08/2023 10:21:55 PM

Electrophoresis File Run Summary

Sample #19

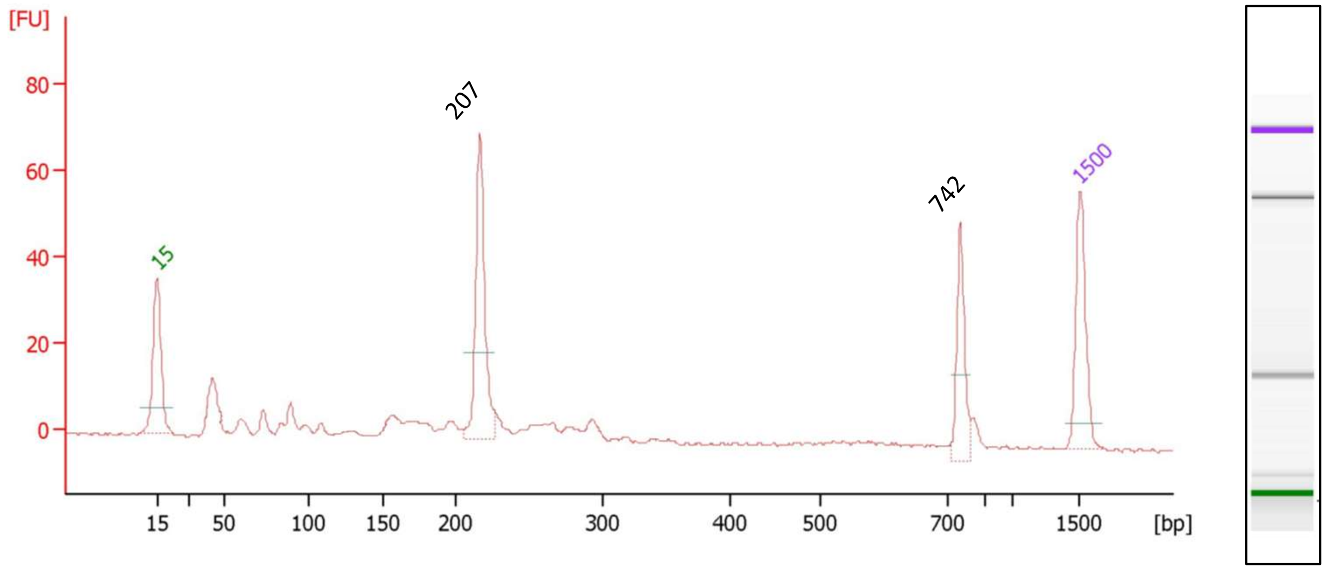

Peak table for Sample #19 :

Peak		Size [bp]	Conc. [ng/µl]	Molarity [nmol/l]	Observations
1	◀	15	4.20	424.4	Lower Marker
2	L	50	1.00	1.23	Ladder Peak
3	L	207	2.80	16.9	Ladder Peak
4	L	742	2.95	17.1	Ladder Peak
5	▶	1,500	2.10	2.2	Upper Marker

 © Copyright 2003 - 2009 Agilent Technologies, Inc.

Assay Class: DNA 1000
Data Path: C:\...-30\2100 expert_DNA 1000_DE34903611_2023-03-06_TRN_AluI_11_20.xad
Created: 3/08/2023 10:21:55 PM
Modified: 3/08/2023 10:21:55 PM

Electrophoresis File Run Summary

Sample #20

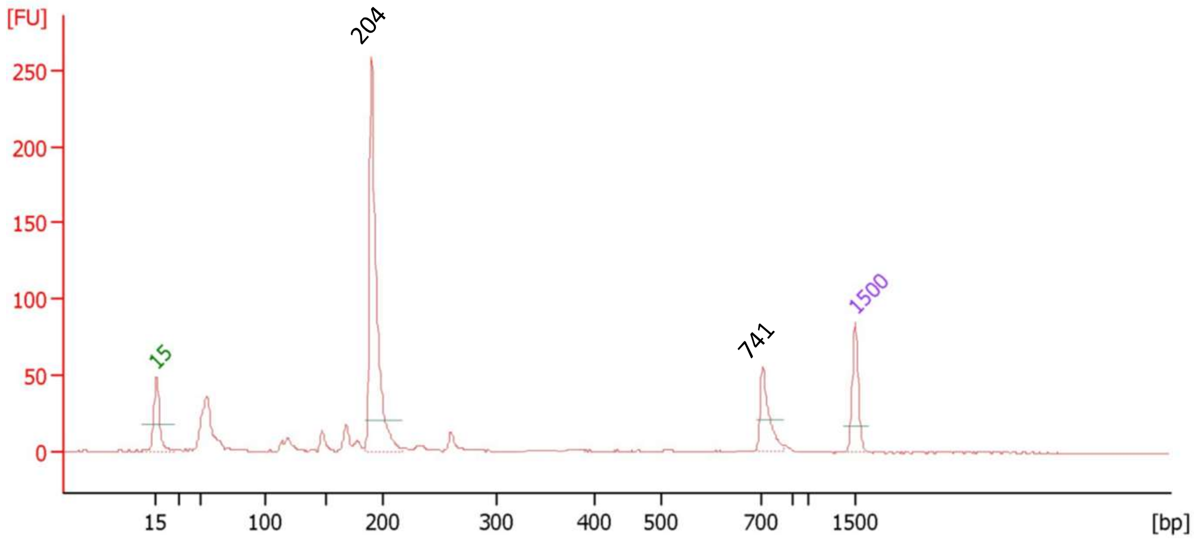

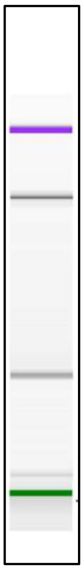

Peak table for Sample #20 :

Peak		Size [bp]	Conc. [ng/µl]	Molarity [nmol/l]	Observations
1	◀	15	4.20	424.4	Lower Marker
2	L	204	1.45	18.9	Ladder Peak
3	L	741	2.35	6.3	Ladder Peak
4	▶	1,500	2.10	2.2	Upper Marker

 © Copyright 2003 - 2009 Agilent Technologies, Inc.

Assay Class: DNA 1000
Data Path: C:\...-30\2100 expert_DNA 1000_DE34903611_2023-03-06_TRN_AluI_11_20.xad
Created: 3/08/2023 10:21:55 PM
Modified: 3/08/2023 10:21:55 PM

Electrophoresis File Run Summary

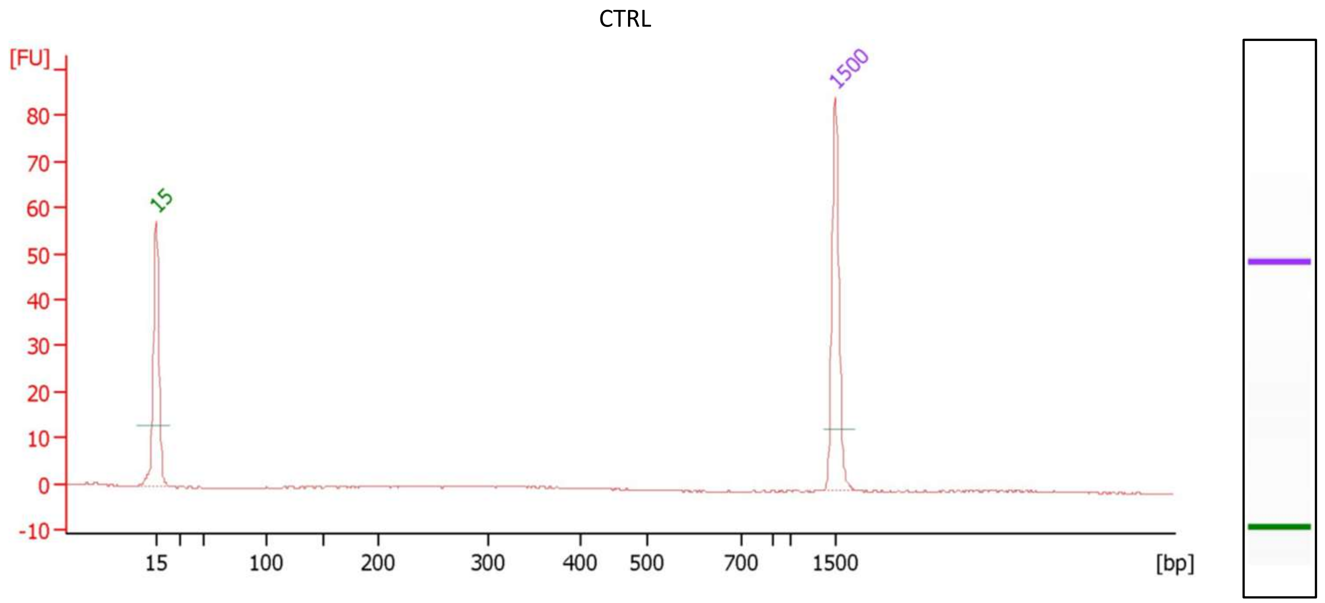

Peak table for Sample CTRL :

Peak		Size [bp]	Conc. [ng/µl]	Molarity [nmol/l]	Observations
1	◀	15	4.20	424.4	Lower Marker
2	▶	1,500	2.10	2.2	Upper Marker

Assay Class: DNA 1000
Data Path: C:\...-30\2100 expert_DNA 1000_DE34903611_2023-03-06_TRN_AluI_11_20.xad
Created: 3/08/2023 10:21:55 PM
Modified: 3/08/2023 10:21:55 PM

Electrophoresis File Run Summary

CTRL

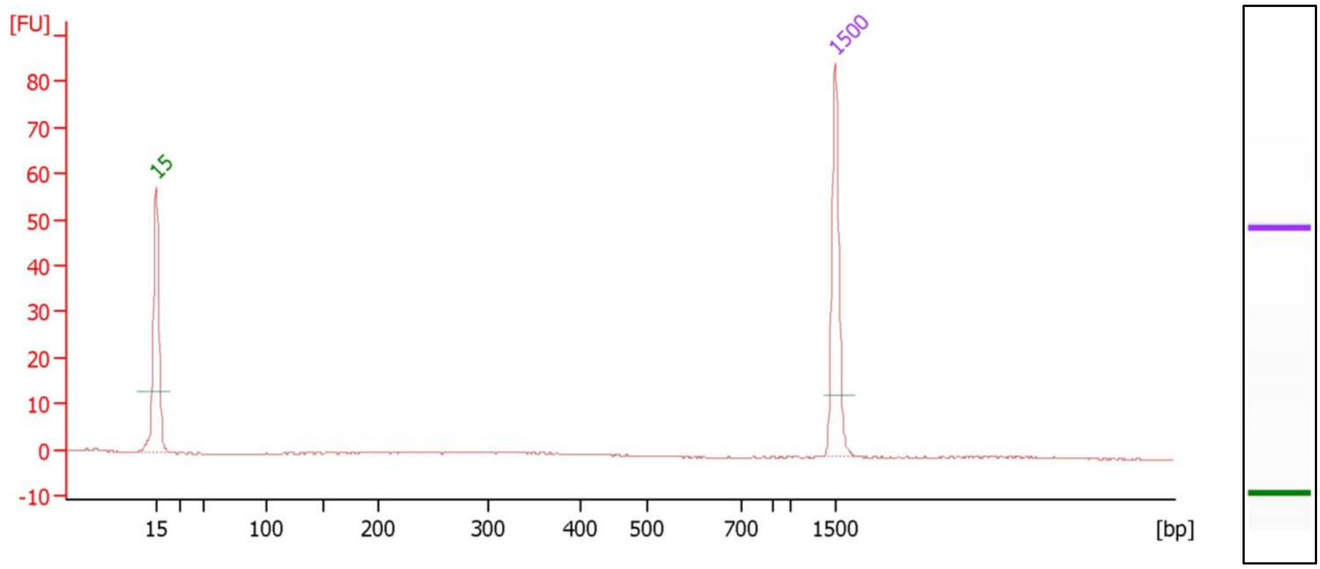

Peak table for Sample CTRL :

Peak		Size [bp]	Conc. [ng/µl]	Molarity [nmol/l]	Observations
1	◀	15	4.20	424.4	Lower Marker
2	▶	1,500	2.10	2.1	Upper Marker

Assay Class: DNA 1000
Data Path: C:\...-30\2100 expert_DNA 1000_DE34903611_2023-03-06_TRN_AluI_11_20.xad
Created: 3/08/2023 10:21:55 PM
Modified: 3/08/2023 10:21:55 PM

Curves

Standard Curve

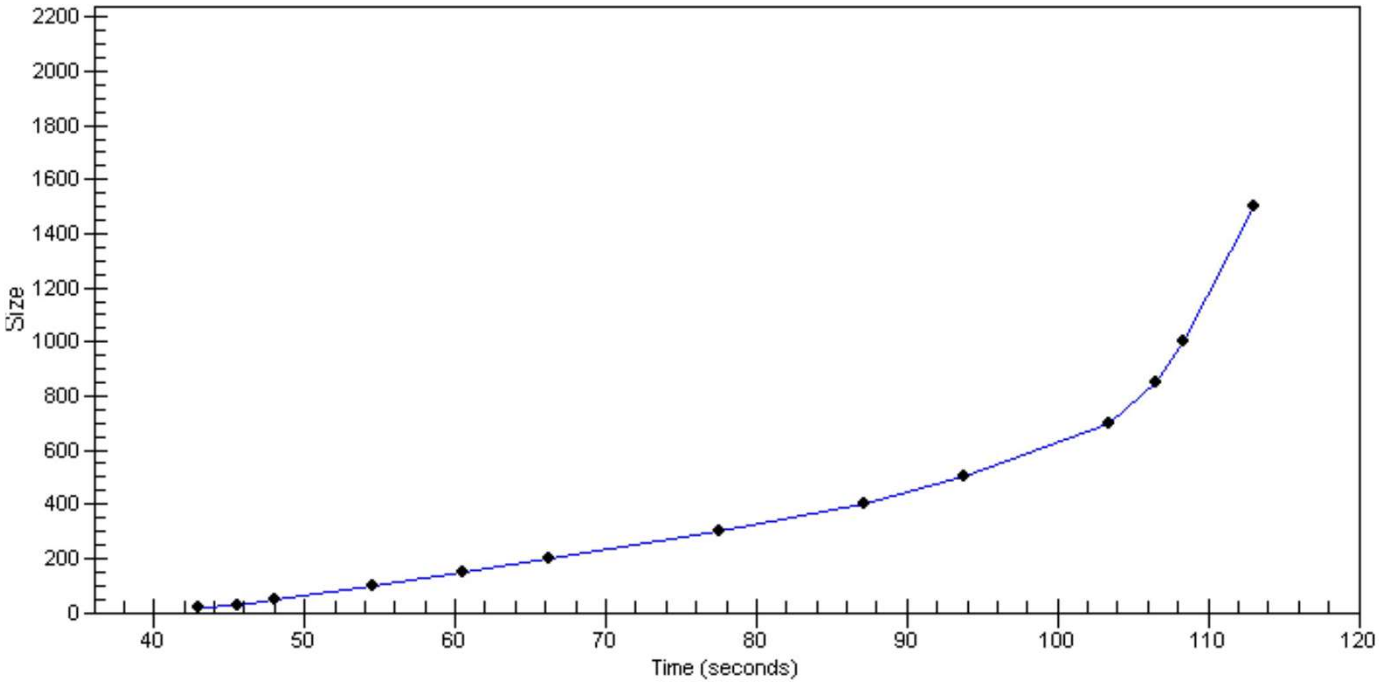

© Copyright 2003 - 2009 Agilent Technologies, Inc.

Assay Class: DNA 1000
Data Path: C:\...-30\2100 expert_DNA 1000_DE34903611_2023-03-06_TRN_AluI_11_20.xad
Created: 3/08/2023 10:21:55 PM
Modified: 3/08/2023 10:21:55 PM

Electrophoresis File Run Summary (Chip Summary)

Sample Name	Sample Content	Rest. Digest	Status	Observation	Result Label	Result Color
Sample #11		☐	✔			
Sample #12		☐	✔			
Sample #13		☐	✔			
Sample #14		☐	✔			
Sample #15		☐	✔			
Sample #16		☐	✔			
Sample #17		☐	✔			
Sample #18		☐	✔			
Sample #19		☐	✔			
Sample #20		☐	✔			
CTRL		☐	✔			
CTRL		☐	✔			

Chip Lot # **Reagent Kit Lot #**

Chip Comments :

 © Copyright 2003 - 2009 Agilent Technologies, Inc.

Assay Class: DNA 1000
Data Path: C:\...-30\2100 expert_DNA 1000_DE34903611_2023-03-06_TRN_AluI_11_20.xad
Created: 3/08/2023 10:21:55 PM
Modified: 3/08/2023 10:21:55 PM

Electrophoresis Assay Details

General Analysis Settings

Result Label **Result Color**

Number of Available Sample and Ladder Wells (Max.) : 13
Minimum Visible Range [s] : 30
Maximum Visible Range [s] : 129
Start Analysis Time Range [s] : 30
End Analysis Time Range [s] : 128.95
Ladder Concentration [ng/µl] : 44
Uses Standard Area for Ladder Fragments
Lower Marker Concentration [ng/µl] : 4.2
Upper Marker Concentration [ng/µl] : 2.1
Used Upper Marker for Quantitation
Standard Curve Fit is Point to Point
Show Data Aligned to Lower and Upper Marker

Integrator Settings

Integration Start Time [s] : 30
Integration End Time [s] : 128.95
Slope Threshold : 0.5
Height Threshold [FU] : 20
Area Threshold : 0.1
Width Threshold [s] : 0.5
Baseline Plateau [s] : 0.5

Filter Settings

Filter Width [s] : 0.5
Polynomial Order : 4

Ladder

Ladder Peak	Size	Area
1	15	25
2	25	26
3	50	34
4	100	41
5	150	45
6	200	52
7	300	63
8	400	76
9	500	83
10	700	88
11	850	86
12	1000	90
13	1500	52

© Copyright 2003 - 2009 Agilent Technologies, Inc.
