# Supplementary material for: Discrimination of Green Coffee (Coffea arabica and Coffea canephora) of Different Geographical Origin Based on Antioxidant Activity, High-Throughput Metabolomics, and DNA RFLP Fingerprinting
Source: Antioxidants (Basel). 2023 May 21;12(5):1135. doi: 10.3390/antiox12051135 (PMC10215230; doi:10.3390/antiox12051135)
Supplement: Supplementary file 1 [file antioxidants-12-01135-s001.zip › Supplementary Data File S2.pdf]

Supplementary Data S2: PCR products of *5S-rRNA-NTS* region separated by the Agilent 2100 Bioanalyzer and reporting the electropherograms of CA e CC accessions before and after digestion with *MseI* and *XhoII*.

Assay Class: DNA 1000
Data Path: C:\...-30\2100 expert_DNA 1000_DE34903611_2023-03-06_NTS_1_10.xad

Created: 3/08/2023 8:30:21 AM
Modified: 3/08/2023 8:30:21 AM

Electrophoresis File Run Summary

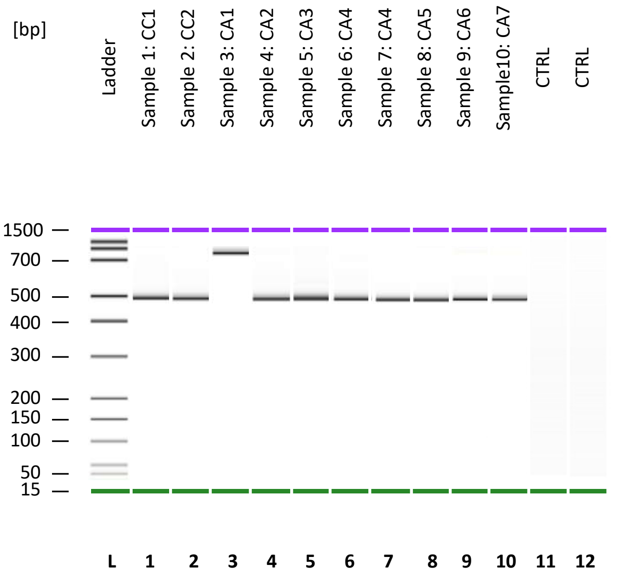

Instrument Information:

Instrument Name: DE34903611

Firmware: C.01.069

Serial #: DE34903611

Type: G2938C

Assay Information:

Assay Origin Path: C:\Program Files\Agilent\2100 bioanalyzer\2100 expert\assays\dsDNA\DNA 1000 Series II.xsy

Assay Class: DNA 1000

Version: 2.3

Assay Comments: DNA Analysis 2 - 1000 bp

© Copyright 2003-2009 Agilent Technologies, Inc.

Chip Information:

Chip Lot #:

Reagent Kit Lot #:

Chip Comments:

Sample #1: CC1

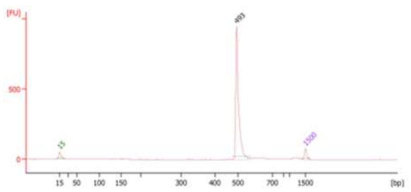

Sample 2: CC2

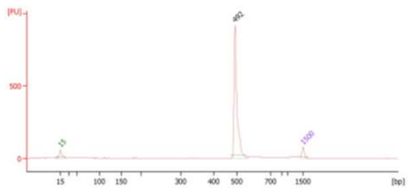

Sample 3: CA1

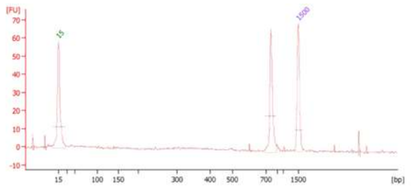

Sample #4: CA2

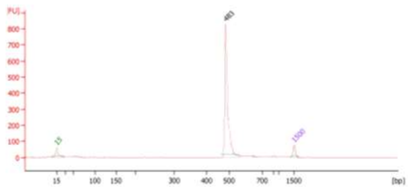

Sample 5: CA3

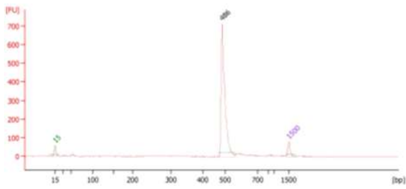

Sample 6: CC3

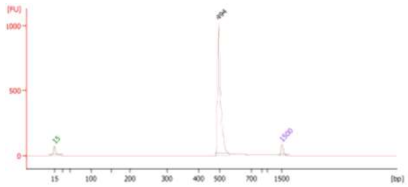

Sample #7: CC4

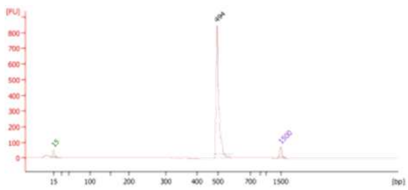

Sample 8: CA4

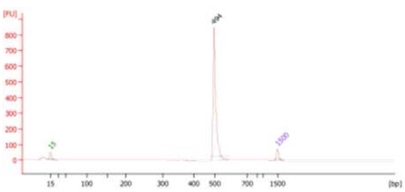

Sample 9: CA5

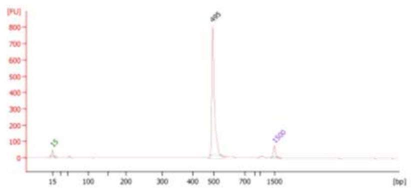

Sample #10: CA6

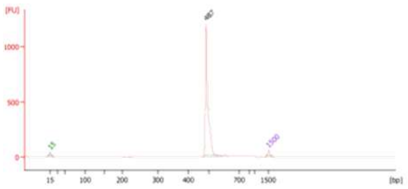

CTRL

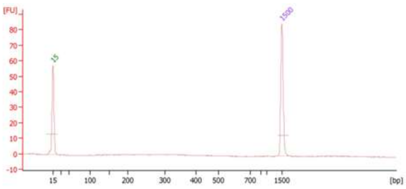

CTRL

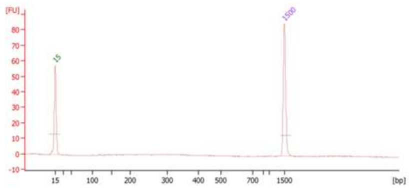

Assay Class: DNA 1000
Data Path: C:\...-30\2100 expert_DNA 1000_DE34903611_2023-03-06_NTS_1_10.xad

Created: 3/08/2023 8:30:21 AM
Modified: 3/08/2023 8:30:21 AM

Electrophoresis File Run Summary

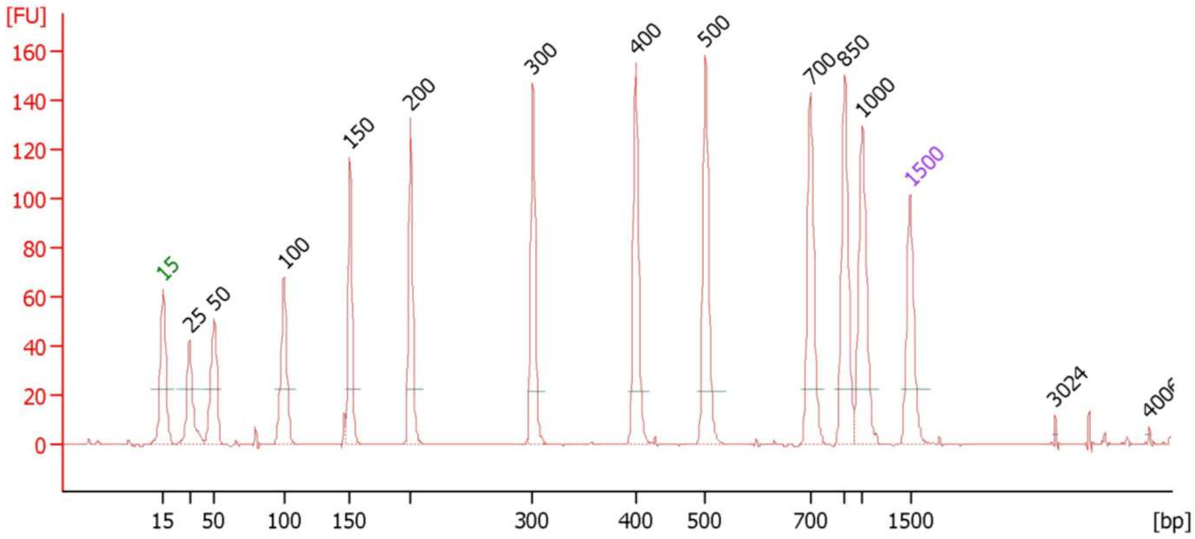

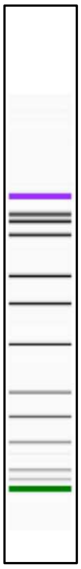

Peak table for Ladder :

Peak		Size [bp]	Conc. [ng/µl]	Molarity [nmol/l]	Observations
1	◀	15	4.20	4.4.2	Lower Marker
2	L	25	4.00	242.4	Ladder Peak
3	L	50	4.00	121.2	Ladder Peak
4	L	100	4.00	60.6	Ladder Peak
5	L	150	4.00	40.3	Ladder Peak
6	L	200	4.00	30.3	Ladder Peak
7	L	300	4.00	20.2	Ladder Peak
8	L	400	4.00	15.2	Ladder Peak
9	L	500	4.00	12.1	Ladder Peak
10	L	700	4.00	8.7	Ladder Peak
11	L	850	4.00	7.1	Ladder Peak
12	L	1,000	4.00	6.1	Ladder Peak
13	▶	1,500	2.10	2.1	Upper Marker
14		3,024	0.00	0.00	
15		4,006	0.00	0.00	

 © Copyright 2003 - 2009 Agilent Technologies, Inc.

Assay Class: DNA 1000
Data Path: C:\...-30\2100 expert_DNA 1000_DE34903611_2023-03-06_NTS_1_10.xad

Created: 3/08/2023 8:30:21 AM
Modified: 3/08/2023 8:30:21 AM

Electrophoresis File Run Summary

Sample #1: CC1

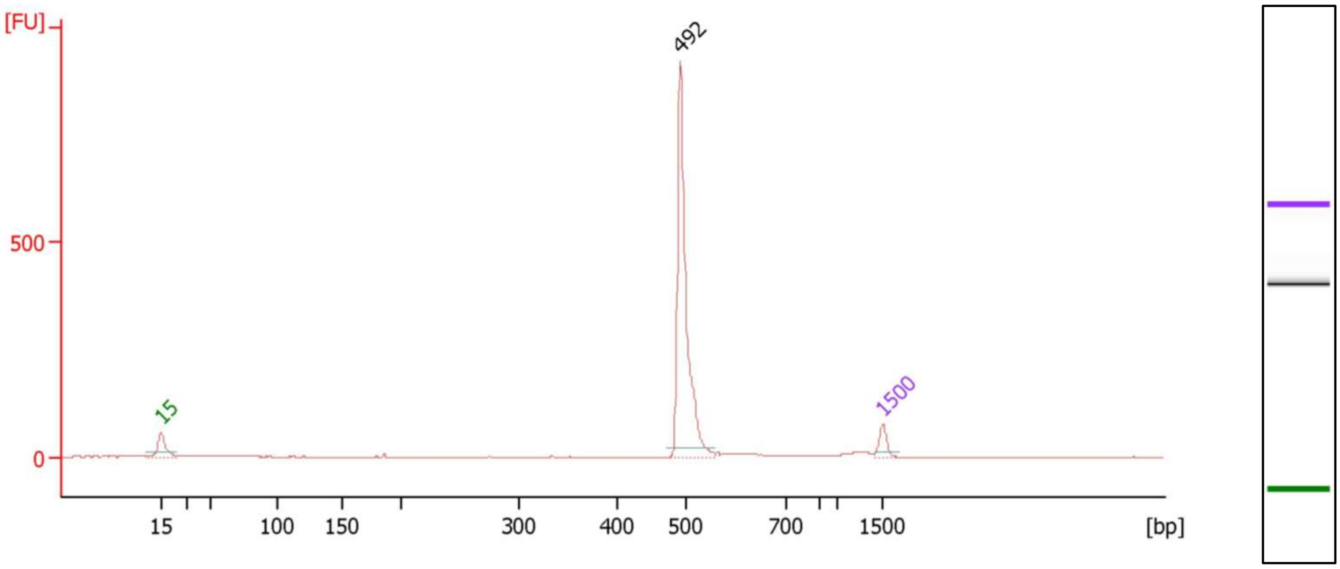

Peak table for Sample #1 :

Peak		Size [bp]	Conc. [ng/µl]	Molarity [nmol/l]	Observations
1		15	4.20	424.4	Lower Marker
2		492	33.52	103.2	Ladder Peak
3		1,500	2.10	2.2	Upper Marker

 © Copyright 2003 - 2009 Agilent Technologies, Inc.

Assay Class: DNA 1000
Data Path: C:\...-30\2100 expert_DNA 1000_DE34903611_2023-03-06_NTS_1_10.xad

Created: 3/08/2023 8:30:21 AM
Modified: 3/08/2023 8:30:21 AM

Electrophoresis File Run Summary

Sample #2: CC2

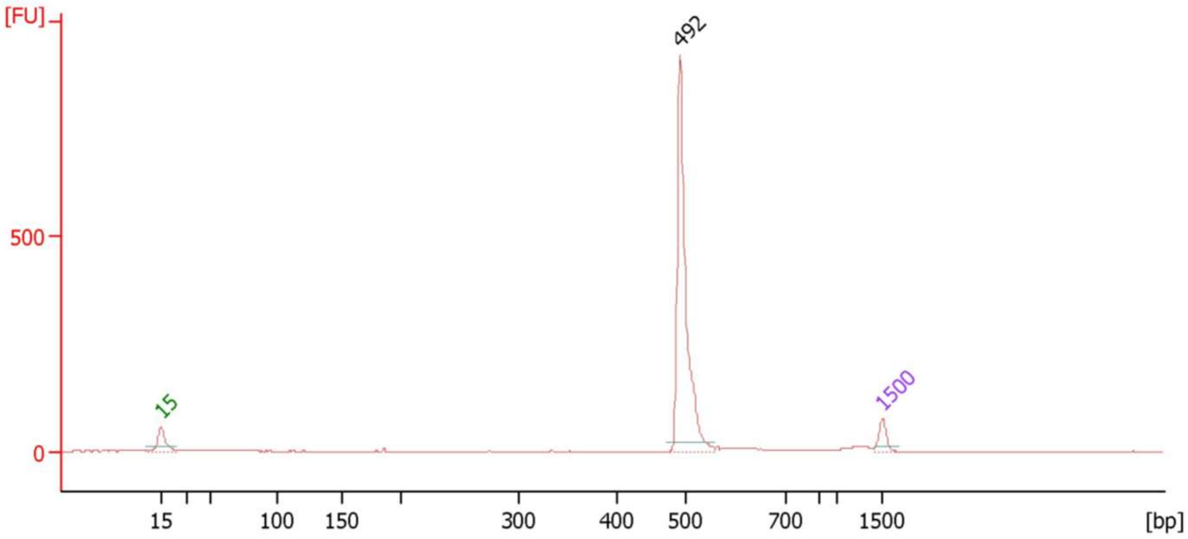

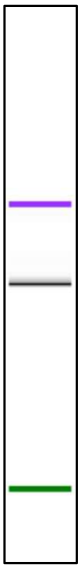

Peak table for Sample #2 :

Peak		Size [bp]	Conc. [ng/µl]	Molarity [nmol/l]	Observations
1	◀	15	4.20	424.4	Lower Marker
2	L	492	33.52	103.2	Ladder Peak
3	▶	1,500	2.10	2.2	Upper Marker

© Copyright 2003 - 2009 Agilent Technologies, Inc.

Assay Class: DNA 1000
Data Path: C:\...-30\2100 expert_DNA 1000_DE34903611_2023-03-06_NTS_1_10.xad

Created: 3/08/2023 8:30:21 AM
Modified: 3/08/2023 8:30:21 AM

Electrophoresis File Run Summary

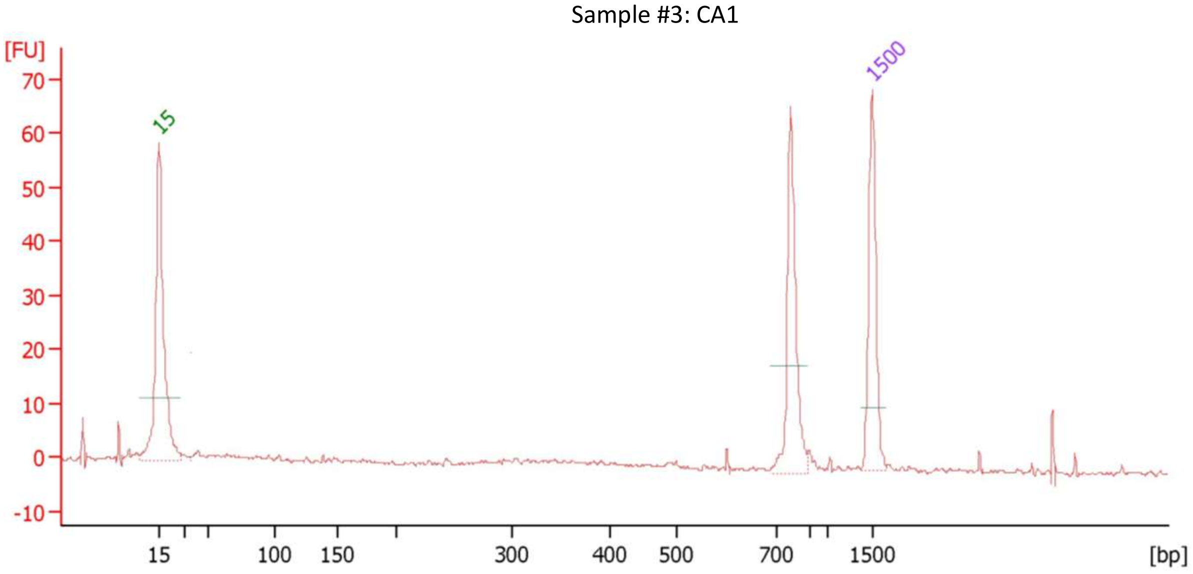

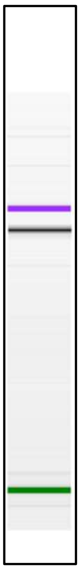

Peak table for Sample #3 :

Peak		Size [bp]	Conc. [ng/µl]	Molarity [nmol/l]	Observations
1	◀	15	4.20	424.4	Lower Marker
2	L	725	35.91	110.3	Ladder Peak
3	▶	1,500	2.10	2.1	Upper Marker

© Copyright 2003 - 2009 Agilent Technologies, Inc.

Assay Class: DNA 1000
Data Path: C:\...-30\2100 expert_DNA 1000_DE34903611_2023-03-06_NTS_1_10.xad

Created: 3/08/2023 8:30:21 AM
Modified: 3/08/2023 8:30:21 AM

Electrophoresis File Run Summary

Sample #4: CA2

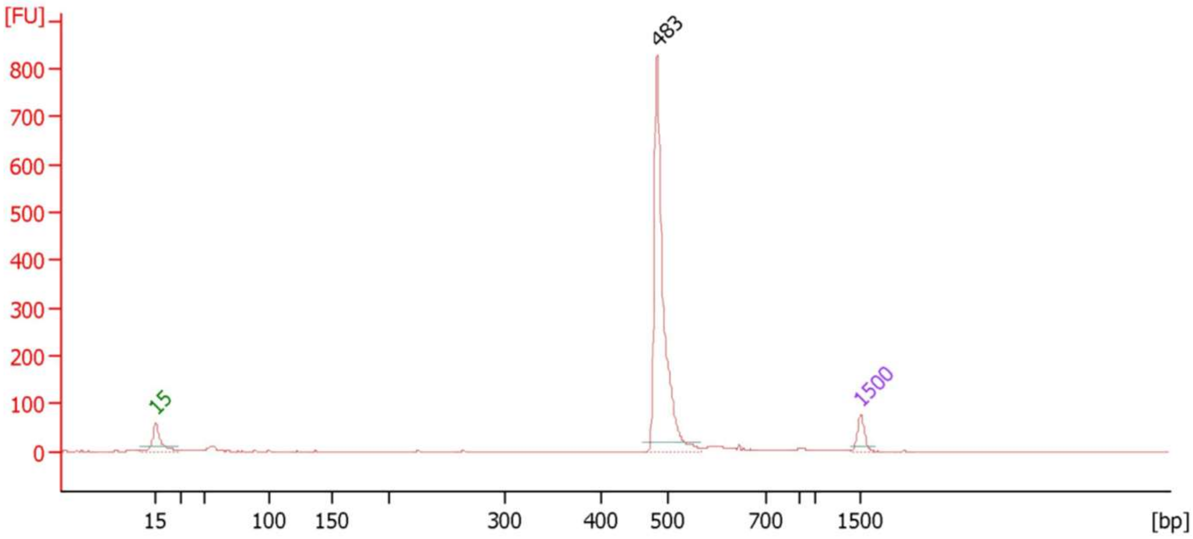

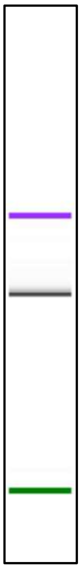

Peak table for Sample #4 :

Peak		Size [bp]	Conc. [ng/µl]	Molarity [nmol/l]	Observations
1	◀	15	4.20	424.2	Lower Marker
2	L	483	32.87	102.4	Ladder Peak
3	▶	1,500	2.10	2.1	Upper Marker

 © Copyright 2003 - 2009 Agilent Technologies, Inc.

Assay Class: DNA 1000
Data Path: C:\...-30\2100 expert_DNA 1000_DE34903611_2023-03-06_NTS_1_10.xad

Created: 3/08/2023 8:30:21 AM
Modified: 3/08/2023 8:30:21 AM

Electrophoresis File Run Summary

Sample #5: CA3

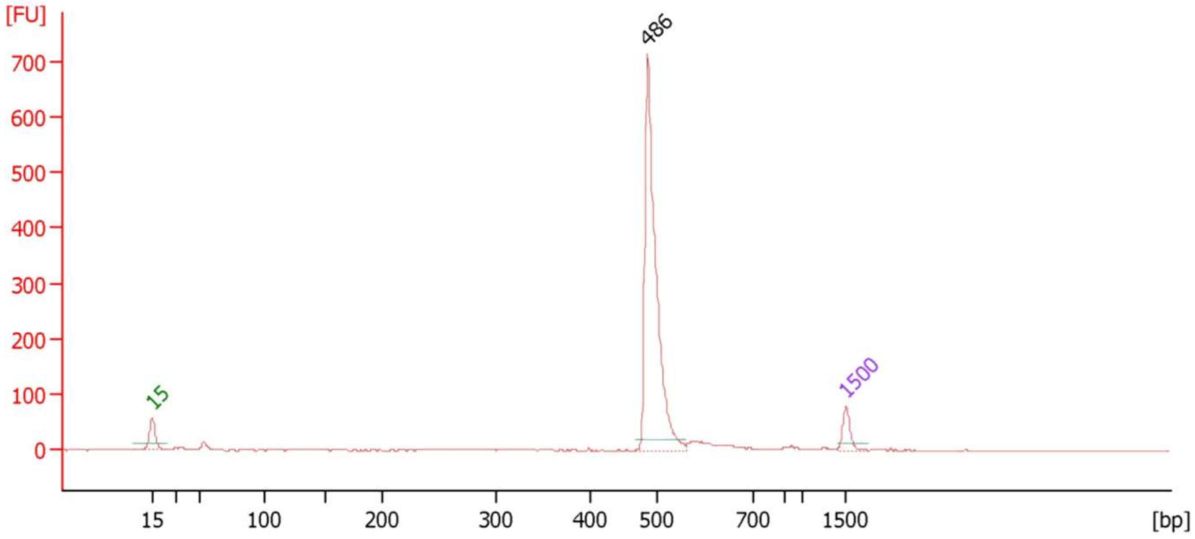

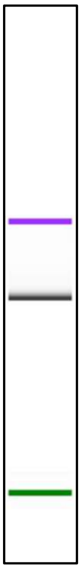

Peak table for Sample #5 :

Peak		Size [bp]	Conc. [ng/µl]	Molarity [nmol/l]	Observations
1	◀	15	4.20	424.2	Lower Marker
2	L	486	32.87	102.4	Ladder Peak
3	▶	1,500	2.10	2.1	Upper Marker

 © Copyright 2003 - 2009 Agilent Technologies, Inc.

Assay Class: DNA 1000
Data Path: C:\...-30\2100 expert_DNA 1000_DE34903611_2023-03-06_NTS_1_10.xad

Created: 3/08/2023 8:30:21 AM
Modified: 3/08/2023 8:30:21 AM

Electrophoresis File Run Summary

Sample #6: CC3

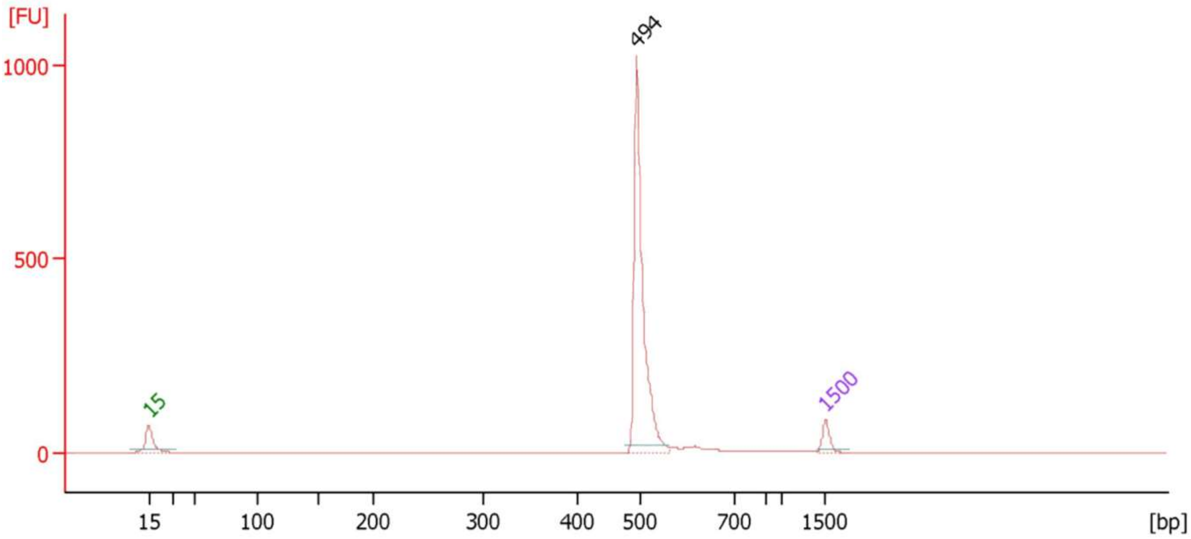

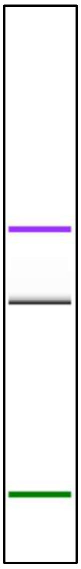

Peak table for Sample #6 :

Peak		Size [bp]	Conc. [ng/µl]	Molarity [nmol/l]	Observations
1	◀	15	4.20	424.2	Lower Marker
2	L	494	34.19	104.9	Ladder Peak
3	▶	1,500	2.10	2.1	Upper Marker

 © Copyright 2003 - 2009 Agilent Technologies, Inc.

Assay Class: DNA 1000
Data Path: C:\...-30\2100 expert_DNA 1000_DE34903611_2023-03-06_NTS_1_10.xad

Created: 3/08/2023 8:30:21 AM
Modified: 3/08/2023 8:30:21 AM

Electrophoresis File Run Summary

Sample #7: CC4

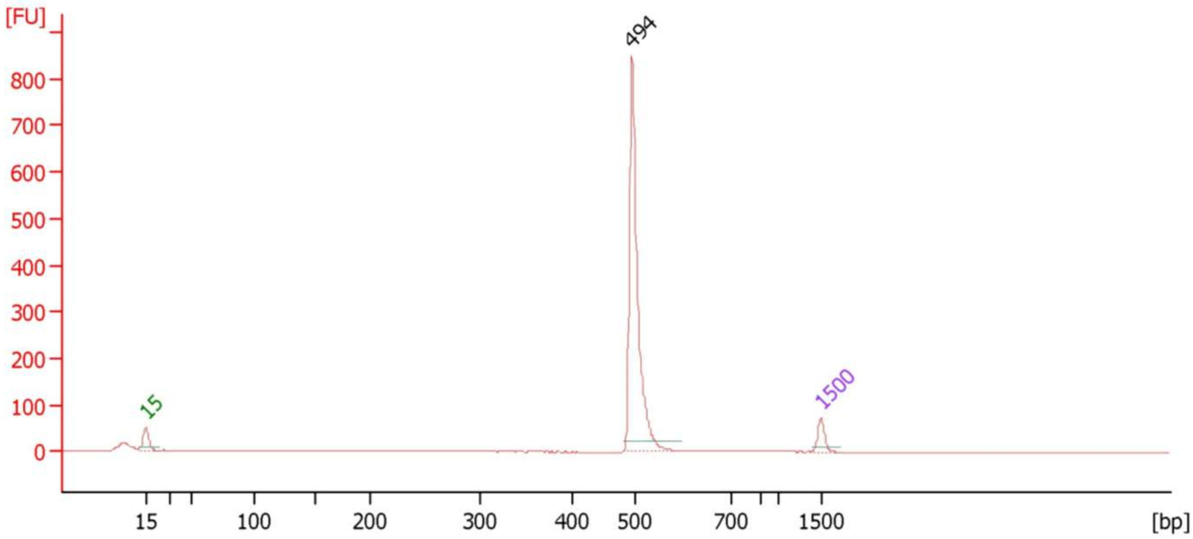

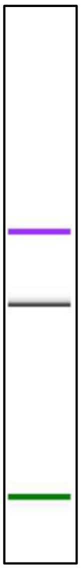

Peak table for Sample #7 :

Peak		Size [bp]	Conc. [ng/µl]	Molarity [nmol/l]	Observations
1	◀	15	4.20	424.4	Lower Marker
2	L	494	33.90	104.0	Ladder Peak
3	▶	1,500	2.10	2.2	Upper Marker

 © Copyright 2003 - 2009 Agilent Technologies, Inc.

Assay Class: DNA 1000
Data Path: C:\...-30\2100 expert_DNA 1000_DE34903611_2023-03-06_NTS_1_10.xad

Created: 3/08/2023 8:30:21 AM
Modified: 3/08/2023 8:30:21 AM

Electrophoresis File Run Summary

Sample #8: CA4

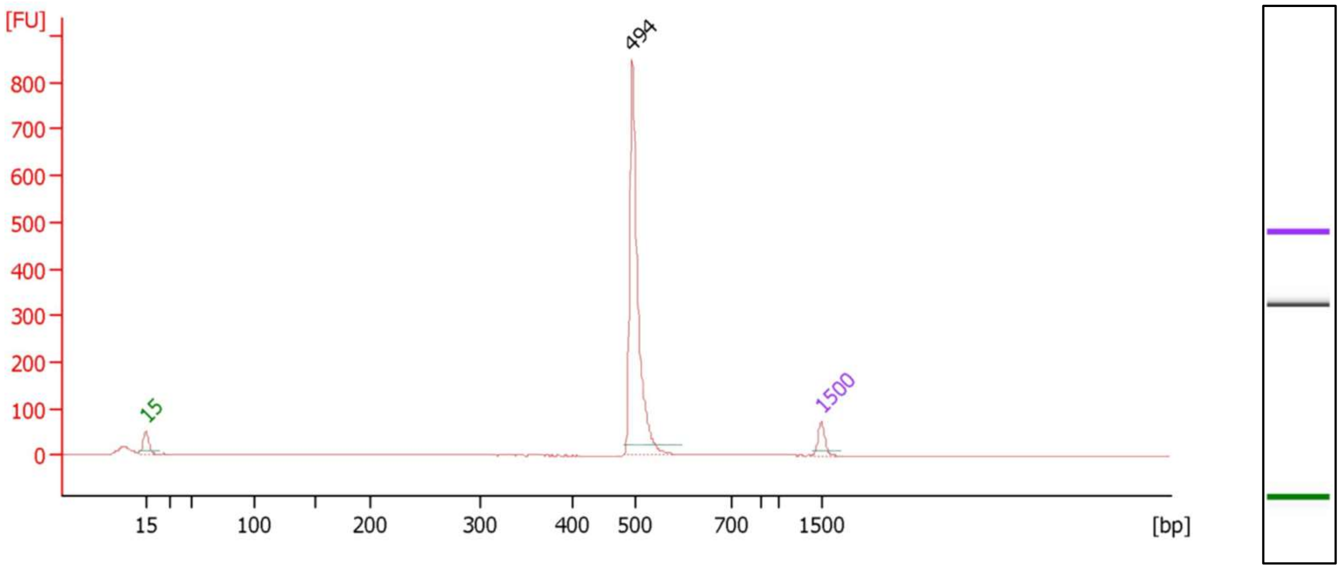

Peak table for Sample #8 :

Peak		Size [bp]	Conc. [ng/µl]	Molarity [nmol/l]	Observations
1	◀	15	4.20	424.4	Lower Marker
2	L	494	33.90	104.0	Ladder Peak
3	▶	1,500	2.10	2.2	Upper Marker

© Copyright 2003 - 2009 Agilent Technologies, Inc.

Assay Class: DNA 1000
Data Path: C:\...-30\2100 expert_DNA 1000_DE34903611_2023-03-06_NTS_1_10.xad

Created: 3/08/2023 8:30:21 AM
Modified: 3/08/2023 8:30:21 AM

Electrophoresis File Run Summary

Sample #9: CA5

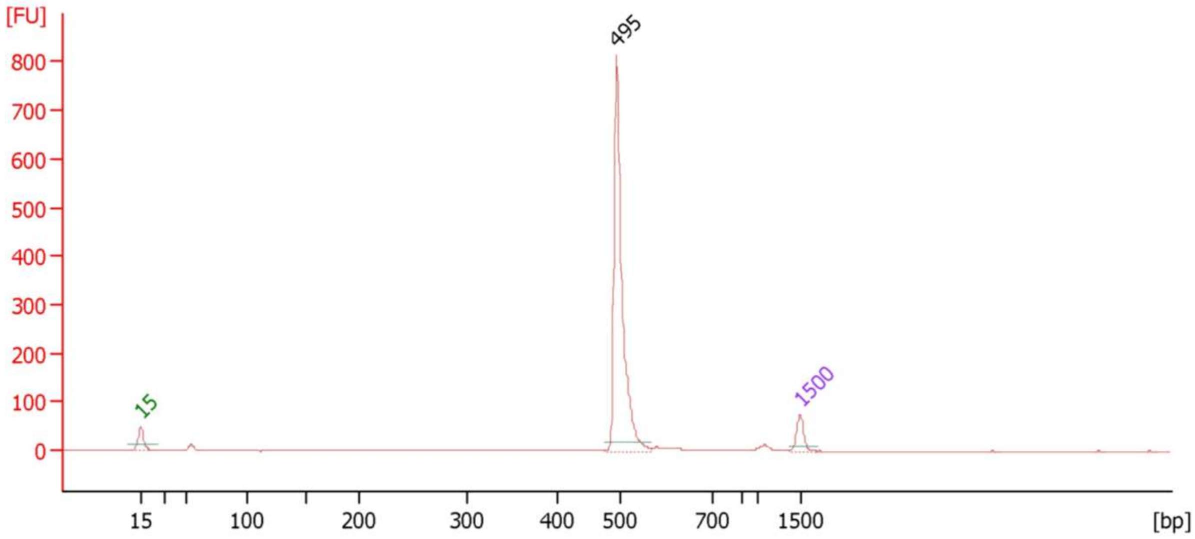

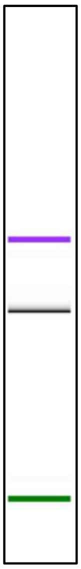

Peak table for Sample #9 :

Peak		Size [bp]	Conc. [ng/µl]	Molarity [nmol/l]	Observations
1		15	4.20	424.4	Lower Marker
2		495	29.43	90.1	Ladder Peak
3		1,500	2.10	2.2	Upper Marker

© Copyright 2003 - 2009 Agilent Technologies, Inc.

Assay Class: DNA 1000
Data Path: C:\...-30\2100 expert_DNA 1000_DE34903611_2023-03-06_NTS_1_10.xad

Created: 3/08/2023 8:30:21 AM
Modified: 3/08/2023 8:30:21 AM

Electrophoresis File Run Summary

Sample #10: CA6

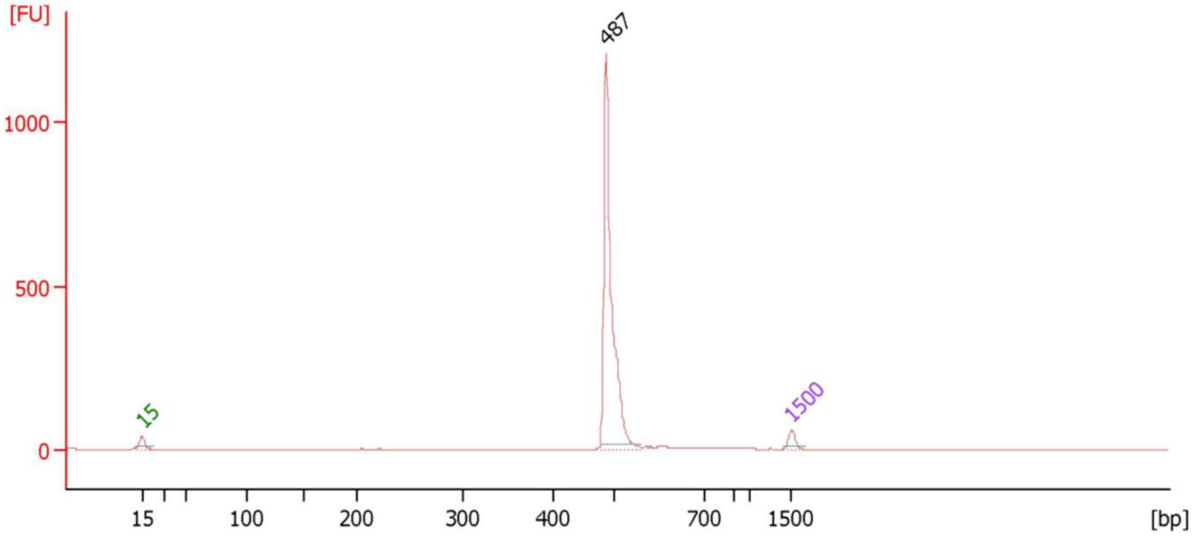

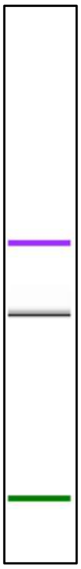

Peak table for Sample #10 :

Peak		Size [bp]	Conc. [ng/µl]	Molarity [nmol/l]	Observations
1	◀	15	4.20	424.4	Lower Marker
2	L	487	55.17	171.6	Ladder Peak
3	▶	1,500	2.10	2.1	Upper Marker

 © Copyright 2003 - 2009 Agilent Technologies, Inc.

Assay Class: DNA 1000
Data Path: C:\...-30\2100 expert_DNA 1000_DE34903611_2023-03-06_NTS_1_10.xad
Created: 3/08/2023 8:30:21 AM
Modified: 3/08/2023 8:30:21 AM

Electrophoresis File Run Summary

CTRL

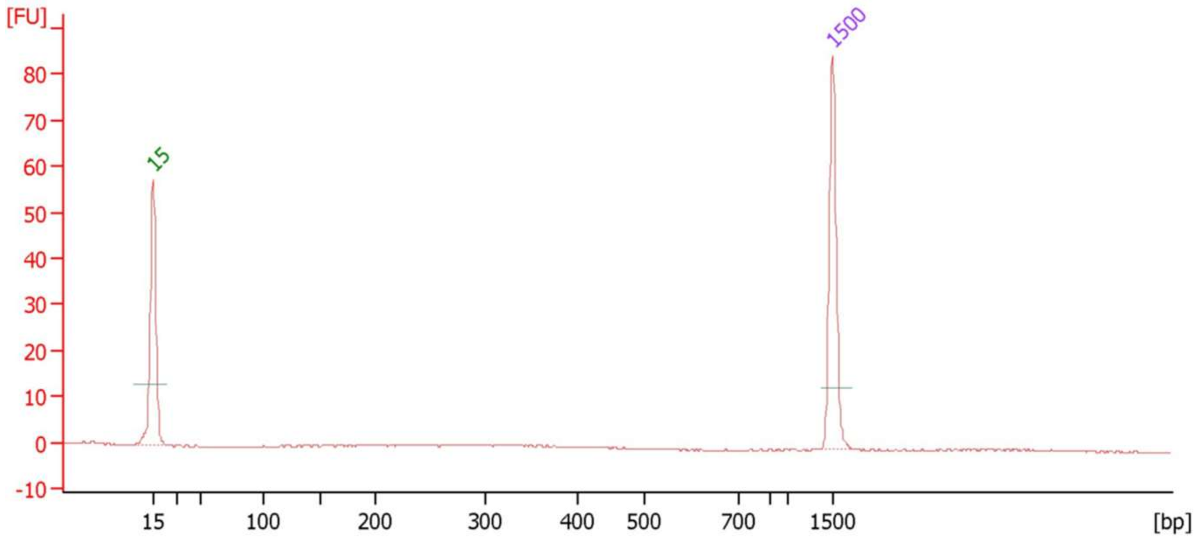

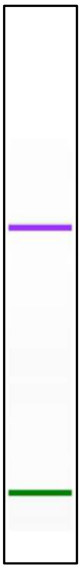

Peak table for Sample CTRL :

Peak		Size [bp]	Conc. [ng/µl]	Molarity [nmol/l]	Observations
**1**	◀	15	4.20	424.4	Lower Marker
**2**	▶	1,500	2.10	2.2	Upper Marker

Assay Class: DNA 1000
Data Path: C:\...-30\2100 expert_DNA 1000_DE34903611_2023-03-06_NTS_1_10.xad

Created: 3/08/2023 8:30:21 AM
Modified: 3/08/2023 8:30:21 AM

Electrophoresis File Run Summary

CTRL

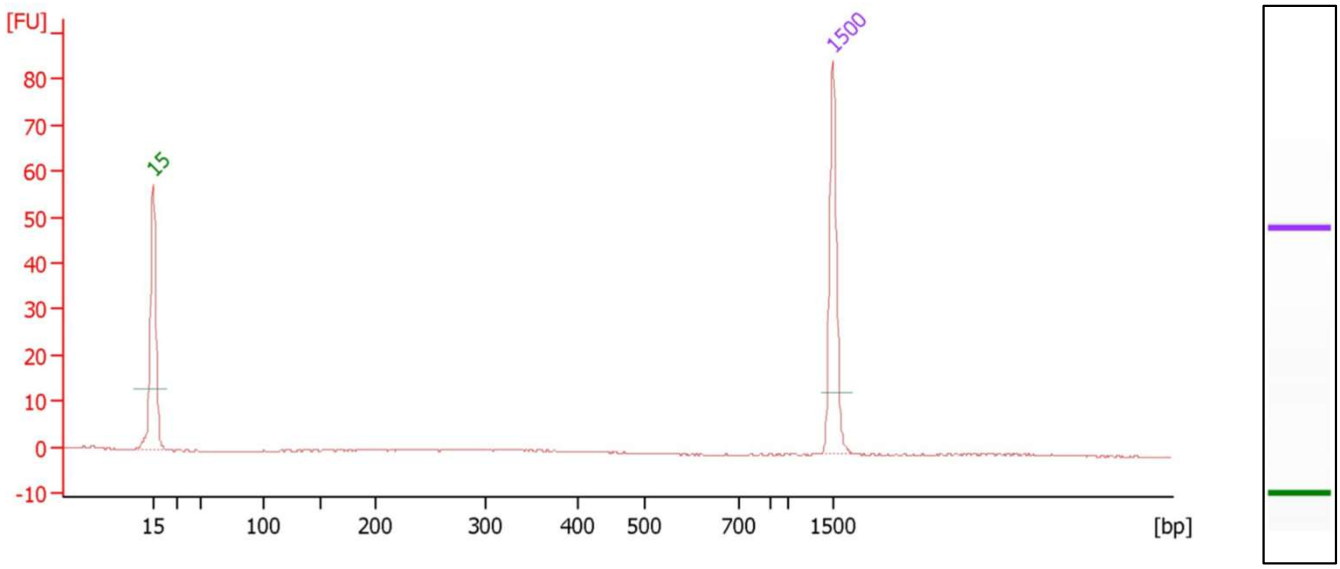

Peak table for Sample CTRL :

Peak		Size [bp]	Conc. [ng/µl]	Molarity [nmol/l]	Observations
1	◀	15	4.20	424.4	Lower Marker
2	▶	1,500	2.10	2.1	Upper Marker

 © Copyright 2003 - 2009 Agilent Technologies, Inc.

Assay Class: DNA 1000
Data Path: C:\...-30\2100 expert_DNA 1000_DE34903611_2023-03-06_NTS_1_10.xad

Created: 3/08/2023 8:30:21 AM
Modified: 3/08/2023 8:30:21 AM

Curves

Standard Curve

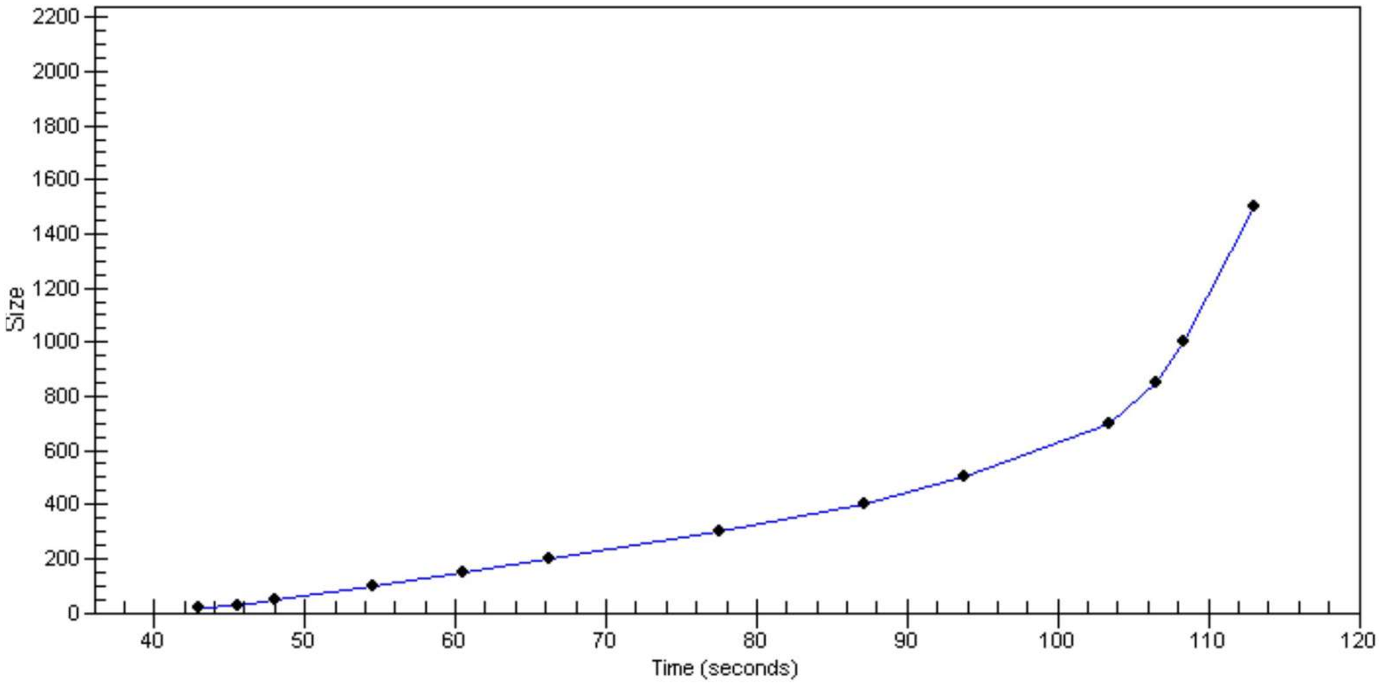

 © Copyright 2003 - 2009 Agilent Technologies, Inc.

Assay Class: DNA 1000
Data Path: C:\...-30\2100 expert_DNA 1000_DE34903611_2023-03-06_NTS_1_10.xad

Created: 3/08/2023 8:30:21 AM
Modified: 3/08/2023 8:30:21 AM

Electrophoresis File Run Summary (Chip Summary)

Sample Name	Sample Content	Rest. Digest	Status	Observation	Result Label	Result Color
Sample #1		☐	✔			
Sample #2		☐	✔			
Sample #3		☐	✔			
Sample #4		☐	✔			
Sample #5		☐	✔			
Sample #6		☐	✔			
Sample #7		☐	✔			
Sample #8		☐	✔			
Sample #9		☐	✔			
Sample #10		☐	✔			
CTRL		☐	✔			
CTRL		☐	✔			

Chip Lot # **Reagent Kit Lot #**

Chip Comments :

 © Copyright 2003 - 2009 Agilent Technologies, Inc.

Assay Class: DNA 1000
Data Path: C:\...-30\2100 expert_DNA 1000_DE34903611_2023-03-06_NTS_1_10.xad
Created: 3/08/2023 8:30:21 AM
Modified: 3/08/2023 8:30:21 AM

Electrophoresis Assay Details

General Analysis Settings

Result Label **Result Color**

Number of Available Sample and Ladder Wells (Max.) : 13
Minimum Visible Range [s] : 30
Maximum Visible Range [s] : 129
Start Analysis Time Range [s] : 30
End Analysis Time Range [s] : 128.95
Ladder Concentration [ng/µl] : 44
Uses Standard Area for Ladder Fragments
Lower Marker Concentration [ng/µl] : 4.2
Upper Marker Concentration [ng/µl] : 2.1
Used Upper Marker for Quantitation
Standard Curve Fit is Point to Point
Show Data Aligned to Lower and Upper Marker

Integrator Settings

Integration Start Time [s] : 30
Integration End Time [s] : 128.95
Slope Threshold : 0.5
Height Threshold [FU] : 20
Area Threshold : 0.1
Width Threshold [s] : 0.5
Baseline Plateau [s] : 0.5

Filter Settings

Filter Width [s] : 0.5
Polynomial Order : 4

Ladder

Ladder Peak	Size	Area
1	15	25
2	25	26
3	50	34
4	100	41
5	150	45
6	200	52
7	300	63
8	400	76
9	500	83
10	700	88
11	850	86
12	1000	90
13	1500	52

 © Copyright 2003 - 2009 Agilent Technologies, Inc.

Assay Class: DNA 1000
Data Path: C:\...-30\2100 expert_DNA 1000_DE34903611_2023-03-06_NTS_11_20.xad

Created: 3/08/2023 11:25:12 AM
Modified: 3/08/2023 11:25:12 AM

Electrophoresis File Run Summary

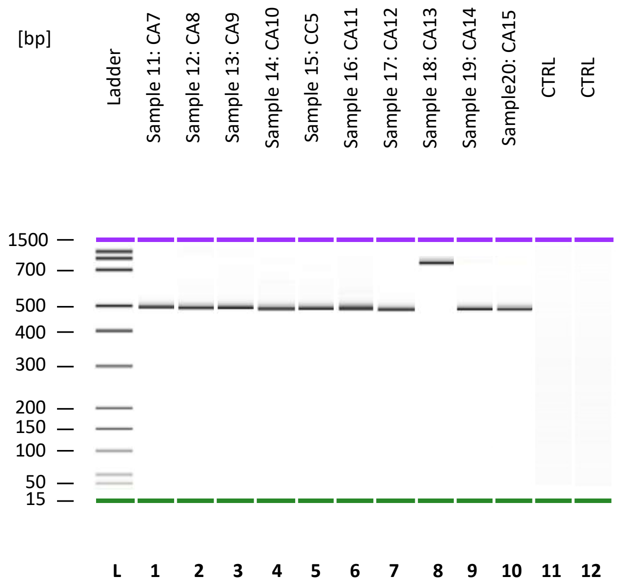

Instrument Information:

Instrument Name: DE34903611
Firmware: C.01.069
Serial #: DE34903611
Type: G2938C

Assay Information:

Assay Origin Path: C:\Program Files\Agilent\2100 bioanalyzer\2100 expert\assays\dsDNA\DNA 1000 Series II.xsy

Assay Class: DNA 1000

Version: 2.3

Assay Comments: DNA Analysis 2 - 1000 bp

© Copyright 2003-2009 Agilent Technologies, Inc.

Chip Information:

Chip Lot #:

Reagent Kit Lot #:

Chip Comments:

Sample #11: CA7

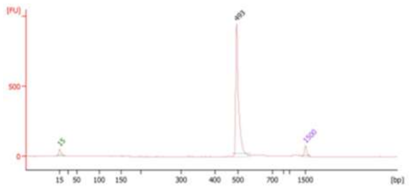

Sample 12: CA8

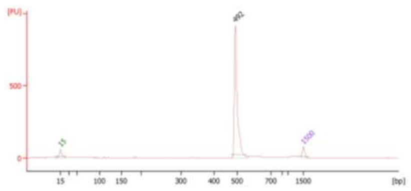

Sample 13: CA9

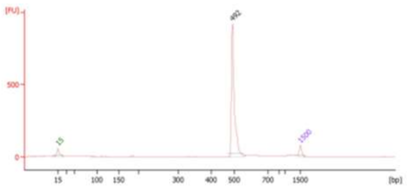

Sample #14: CA10

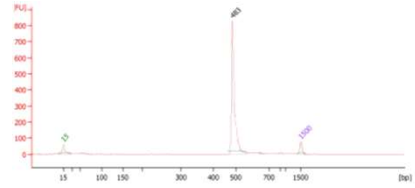

Sample 15: CC5

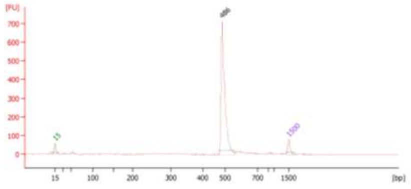

Sample 16: CA11

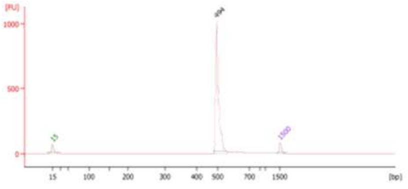

Sample #17: CA12

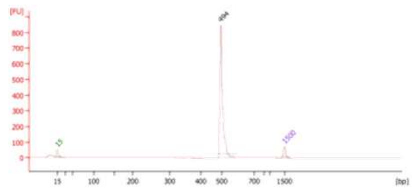

Sample 18: CA13

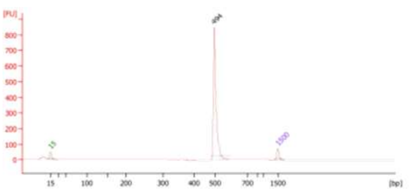

Sample 19: CA14

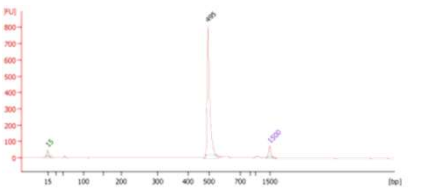

Sample #20: CA15

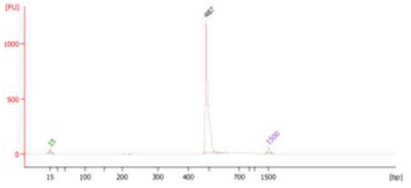

CTRL

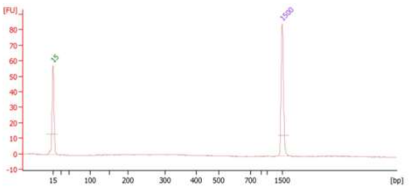

CTRL

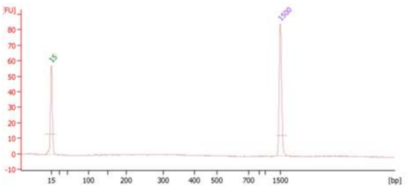

Assay Class: DNA 1000
Data Path: C:\...-30\2100 expert_DNA 1000_DE34903611_2023-03-06_NTS_11_20.xad

Created: 3/08/2023 11:25:12 AM
Modified: 3/08/2023 11:25:12 AM

Electrophoresis File Run Summary

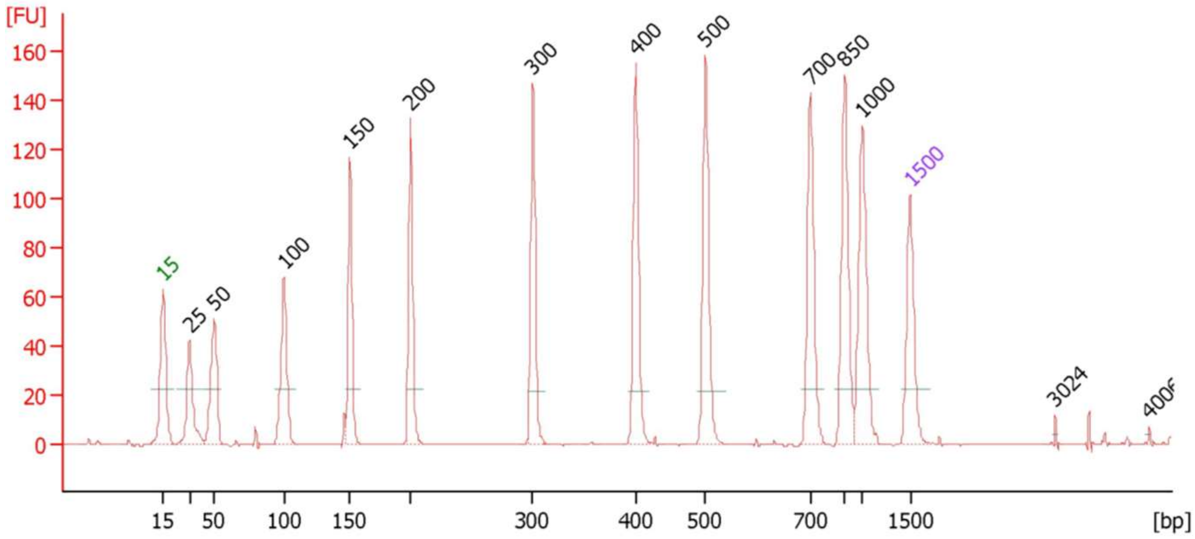

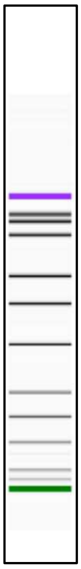

Peak table for Ladder :

Peak		Size [bp]	Conc. [ng/µl]	Molarity [nmol/l]	Observations
1		15	4.20	4.4.2	Lower Marker
2		25	4.00	242.4	Ladder Peak
3		50	4.00	121.2	Ladder Peak
4		100	4.00	60.6	Ladder Peak
5		150	4.00	40.3	Ladder Peak
6		200	4.00	30.3	Ladder Peak
7		300	4.00	20.2	Ladder Peak
8		400	4.00	15.2	Ladder Peak
9		500	4.00	12.1	Ladder Peak
10		700	4.00	8.7	Ladder Peak
11		850	4.00	7.1	Ladder Peak
12		1,000	4.00	6.1	Ladder Peak
13		1,500	2.10	2.1	Upper Marker
14		3,024	0.00	0.00	
15		4,006	0.00	0.00	

 © Copyright 2003 - 2009 Agilent Technologies, Inc.

Assay Class: DNA 1000
Data Path: C:\...-30\2100 expert_DNA 1000_DE34903611_2023-03-06_NTS_11_20.xad

Created: 3/08/2023 11:25:12 AM
Modified: 3/08/2023 11:25:12 AM

Electrophoresis File Run Summary

Sample #11: CA7

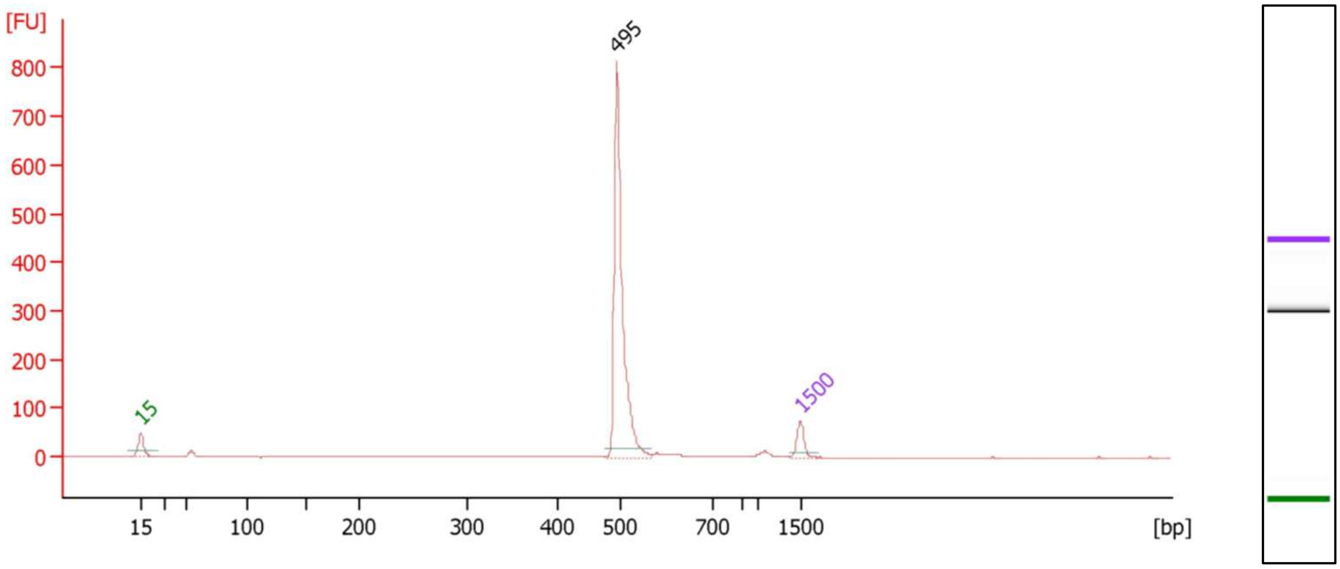

Peak table for Sample #11 :

Peak		Size [bp]	Conc. [ng/µl]	Molarity [nmol/l]	Observations
1		15	4.20	424.4	Lower Marker
2		495	22.23	40.1	Ladder Peak
3		1,500	2.10	2.1	Upper Marker

 © Copyright 2003 - 2009 Agilent Technologies, Inc.

Assay Class: DNA 1000
Data Path: C:\...-30\2100 expert_DNA 1000_DE34903611_2023-03-06_NTS_11_20.xad

Created: 3/08/2023 11:25:12 AM
Modified: 3/08/2023 11:25:12 AM

Electrophoresis File Run Summary

Sample #12: CA8

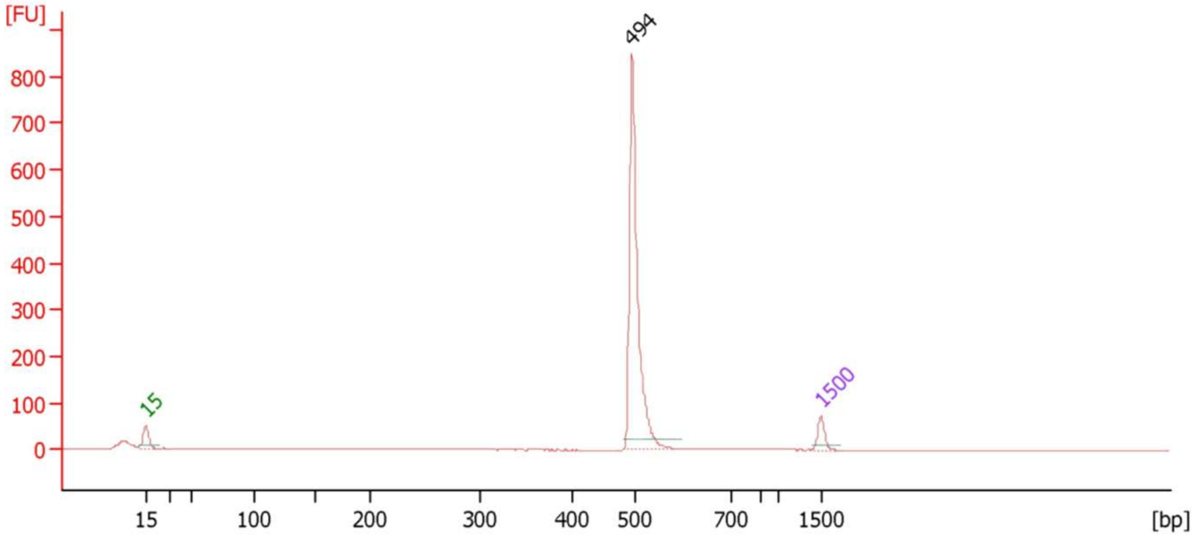

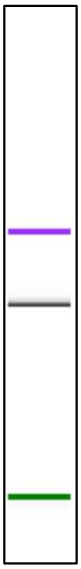

Peak table for Sample #12 :

Peak		Size [bp]	Conc. [ng/µl]	Molarity [nmol/l]	Observations
1		15	4.20	424.4	Lower Marker
2		494	35.70	94.0	Ladder Peak
3		1,500	2.10	2.2	Upper Marker

 © Copyright 2003 - 2009 Agilent Technologies, Inc.

Assay Class: DNA 1000
Data Path: C:\...-30\2100 expert_DNA 1000_DE34903611_2023-03-06_NTS_11_20.xad

Created: 3/08/2023 11:25:12 AM
Modified: 3/08/2023 11:25:12 AM

Electrophoresis File Run Summary

Sample #13: CA9

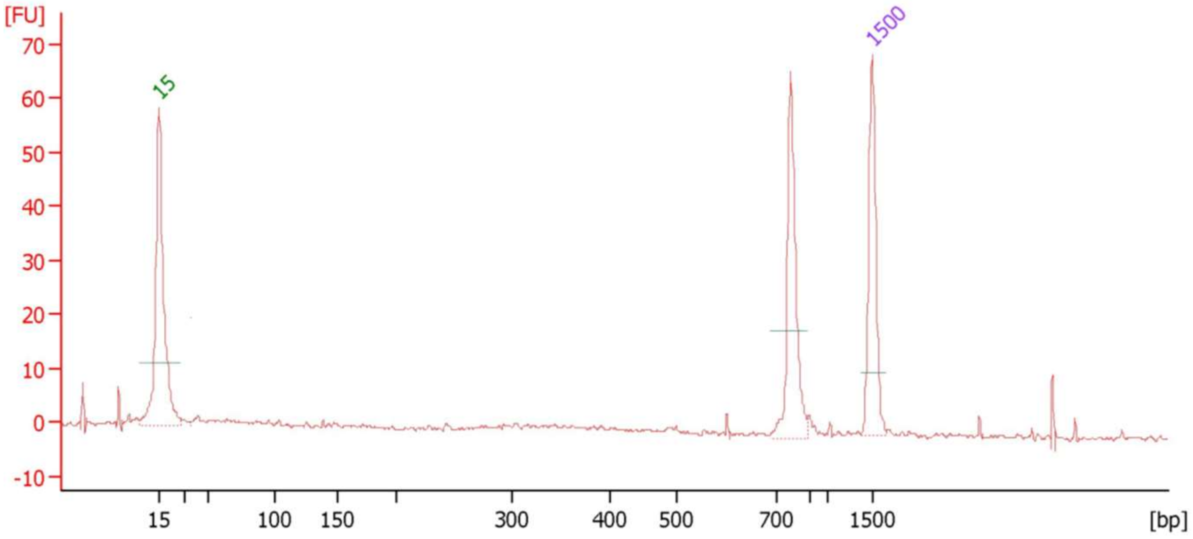

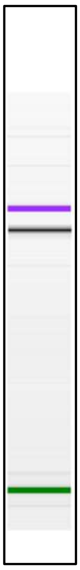

Peak table for Sample #13 :

Peak		Size [bp]	Conc. [ng/µl]	Molarity [nmol/l]	Observations
1	◀	15	4.20	424.4	Lower Marker
2	L	725	5.91	10.3	Ladder Peak
3	▶	1,500	2.10	2.1	Upper Marker

 © Copyright 2003 - 2009 Agilent Technologies, Inc.

Assay Class: DNA 1000
Data Path: C:\...-30\2100 expert_DNA 1000_DE34903611_2023-03-06_NTS_11_20.xad

Created: 3/08/2023 11:25:12 AM
Modified: 3/08/2023 11:25:12 AM

Electrophoresis File Run Summary

Sample #14: CA10

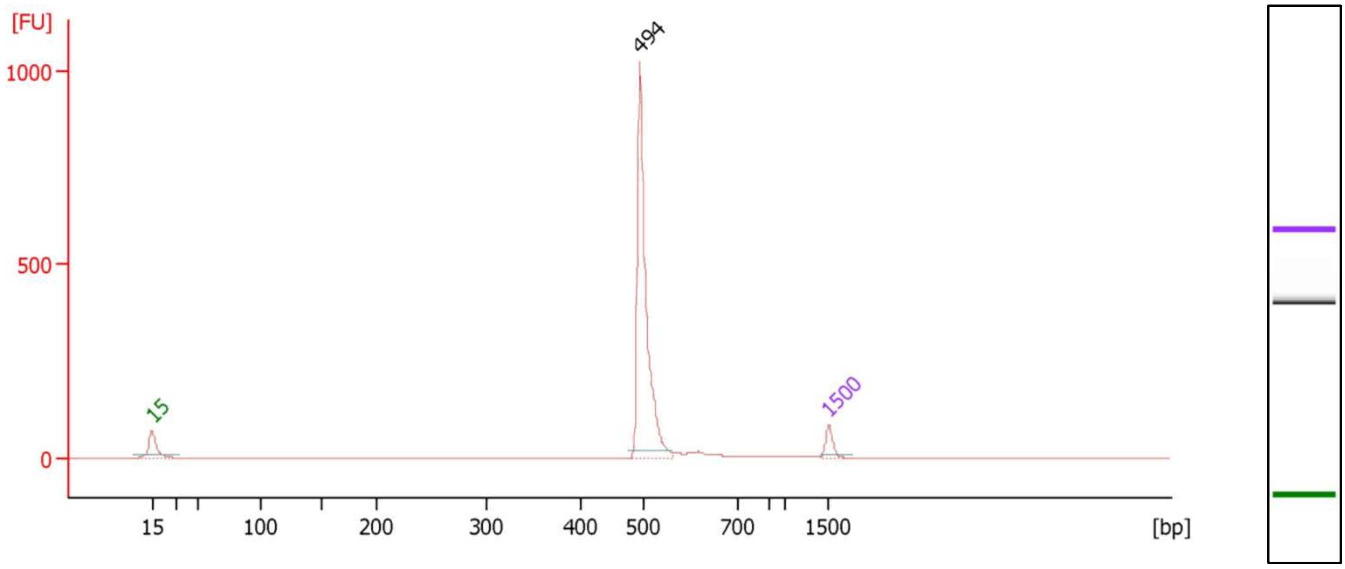

Peak table for Sample #14 :

Peak		Size [bp]	Conc. [ng/µl]	Molarity [nmol/l]	Observations
1		15	4.20	424.2	Lower Marker
2		494	24.19	91.9	Ladder Peak
3		1,500	2.10	2.1	Upper Marker

© Copyright 2003 - 2009 Agilent Technologies, Inc.

Assay Class: DNA 1000
Data Path: C:\...-30\2100 expert_DNA 1000_DE34903611_2023-03-06_NTS_11_20.xad

Created: 3/08/2023 11:25:12 AM
Modified: 3/08/2023 11:25:12 AM

Electrophoresis File Run Summary

Sample #15: CC5

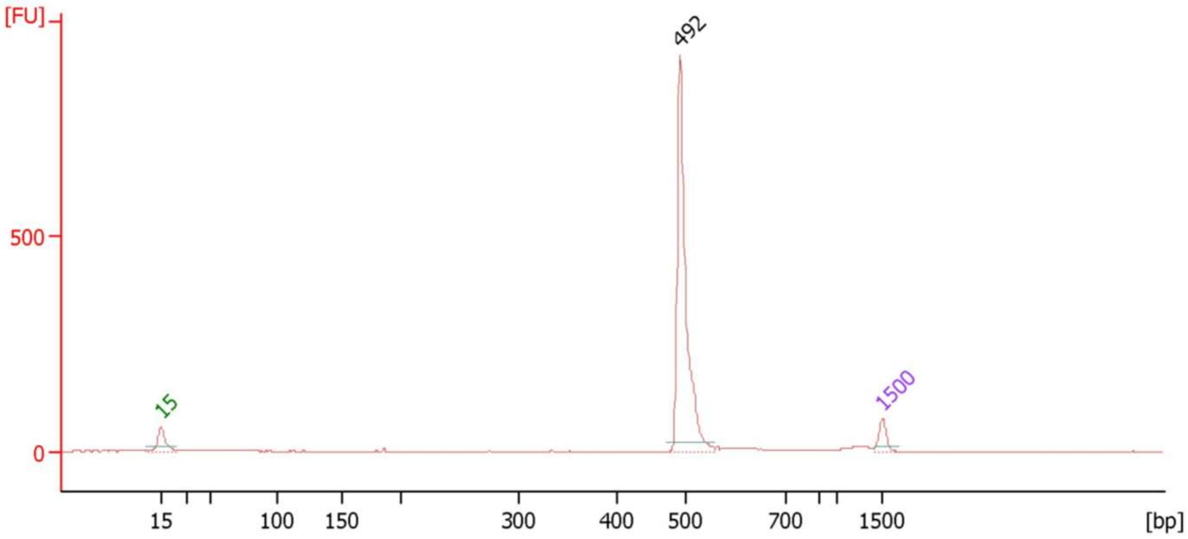

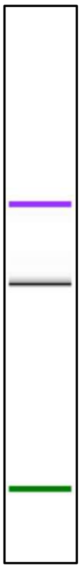

Peak table for Sample #15 :

Peak		Size [bp]	Conc. [ng/µl]	Molarity [nmol/l]	Observations
1	◀	15	4.20	424.4	Lower Marker
2	L	492	31.20	80.5	Ladder Peak
3	▶	1,500	2.10	2.2	Upper Marker

 © Copyright 2003 - 2009 Agilent Technologies, Inc.

Assay Class: DNA 1000
Data Path: C:\...-30\2100 expert_DNA 1000_DE34903611_2023-03-06_NTS_11_20.xad

Created: 3/08/2023 11:25:12 AM
Modified: 3/08/2023 11:25:12 AM

Electrophoresis File Run Summary

Sample #16: CA11

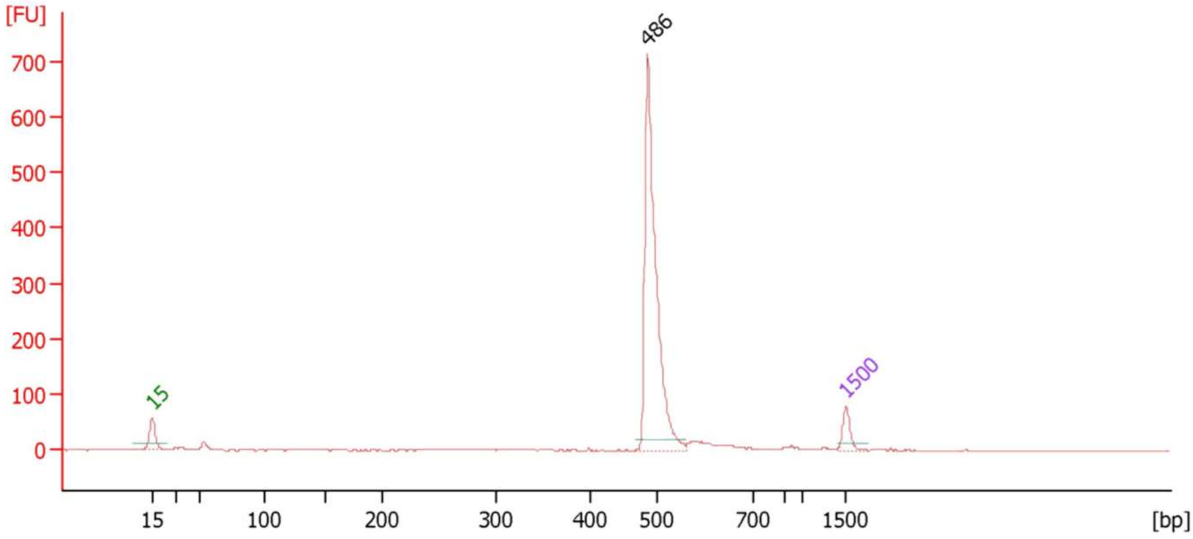

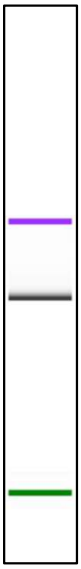

Peak table for Sample #16 :

Peak		Size [bp]	Conc. [ng/µl]	Molarity [nmol/l]	Observations
1		15	4.21	424.4	Lower Marker
2		486	36.32	112.74	Ladder Peak
3		1,500	2.12	2.2	Upper Marker

 © Copyright 2003 - 2009 Agilent Technologies, Inc.

Assay Class: DNA 1000
Data Path: C:\...-30\2100 expert_DNA 1000_DE34903611_2023-03-06_NTS_11_20.xad

Created: 3/08/2023 11:25:12 AM
Modified: 3/08/2023 11:25:12 AM

Electrophoresis File Run Summary

Sample #17: CA12

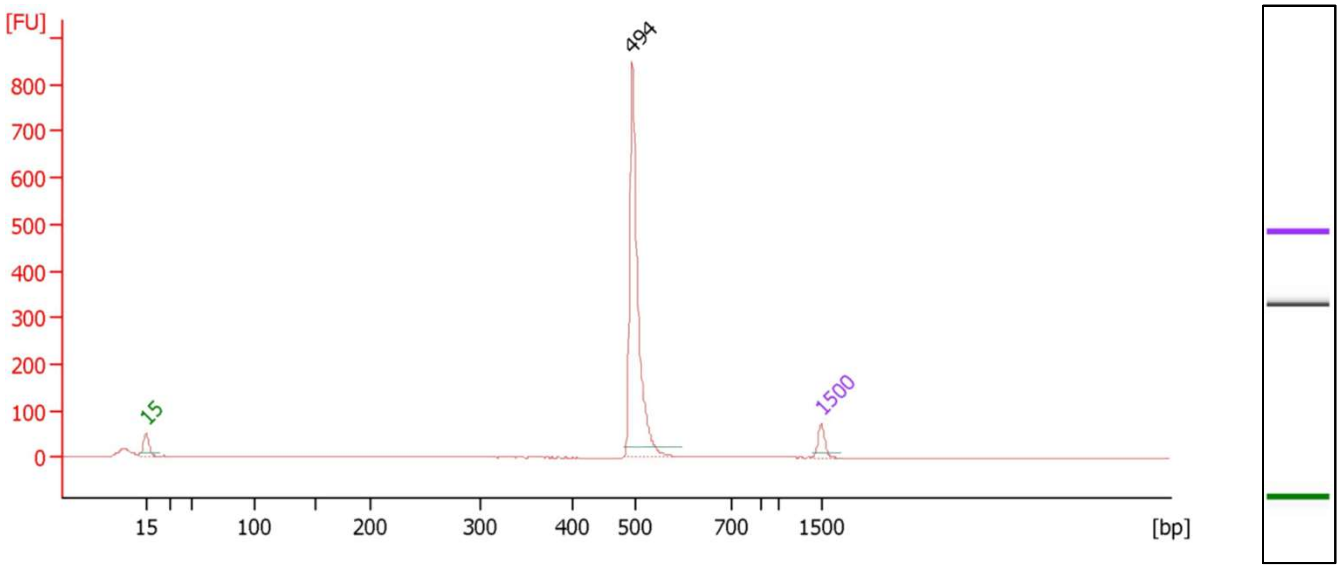

Peak table for Sample #17 :

Peak		Size [bp]	Conc. [ng/µl]	Molarity [nmol/l]	Observations
1		15	4.20	424.4	Lower Marker
2		494	33.85	104.1	Ladder Peak
3		1,500	2.10	2.2	Upper Marker

© Copyright 2003 - 2009 Agilent Technologies, Inc.

Assay Class: DNA 1000
Data Path: C:\...-30\2100 expert_DNA 1000_DE34903611_2023-03-06_NTS_11_20.xad

Created: 3/08/2023 11:25:12 AM
Modified: 3/08/2023 11:25:12 AM

Electrophoresis File Run Summary

Sample #18: CA13

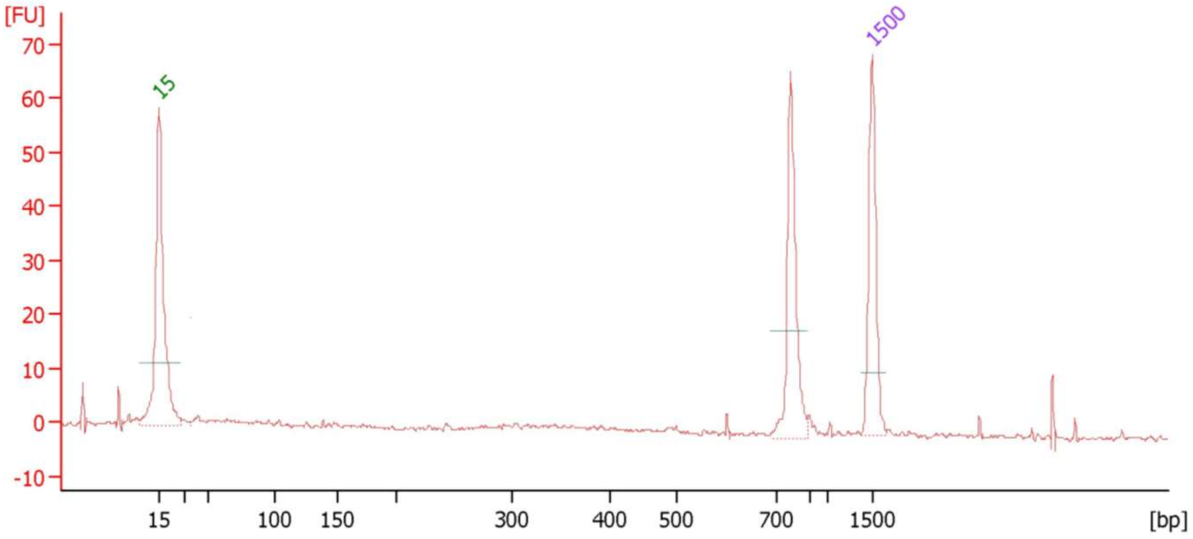

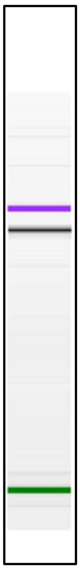

Peak table for Sample #18 :

Peak		Size [bp]	Conc. [ng/µl]	Molarity [nmol/l]	Observations
1		15	4.20	424.4	Lower Marker
2		725	35.91	110.3	Ladder Peak
3		1,500	2.10	2.1	Upper Marker

 © Copyright 2003 - 2009 Agilent Technologies, Inc.

Assay Class: DNA 1000
Data Path: C:\...-30\2100 expert_DNA 1000_DE34903611_2023-03-06_NTS_11_20.xad

Created: 3/08/2023 11:25:12 AM
Modified: 3/08/2023 11:25:12 AM

Electrophoresis File Run Summary

Sample #19: CA14

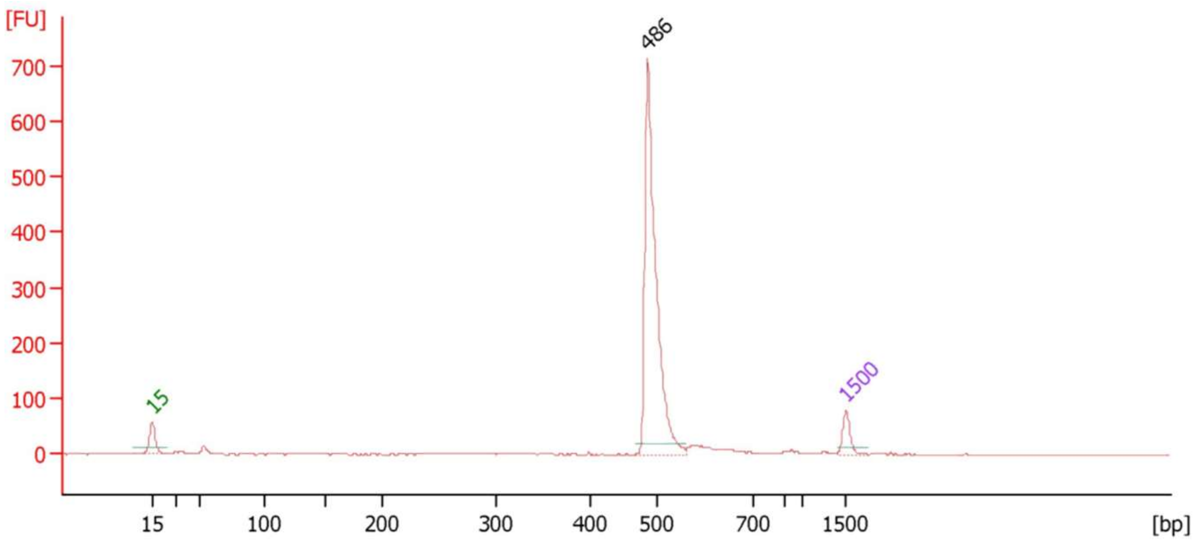

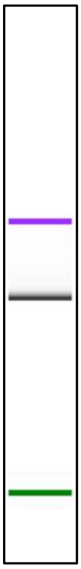

Peak table for Sample #19 :

Peak		Size [bp]	Conc. [ng/µl]	Molarity [nmol/l]	Observations
1	◀	15	4.20	424.2	Lower Marker
2	L	486	30.21	60.2	Ladder Peak
3	▶	1,500	2.10	2.1	Upper Marker

 © Copyright 2003 - 2009 Agilent Technologies, Inc.

Assay Class: DNA 1000
Data Path: C:\...-30\2100 expert_DNA 1000_DE34903611_2023-03-06_NTS_11_20.xad

Created: 3/08/2023 11:25:12 AM
Modified: 3/08/2023 11:25:12 AM

Electrophoresis File Run Summary

Sample #20: CA15

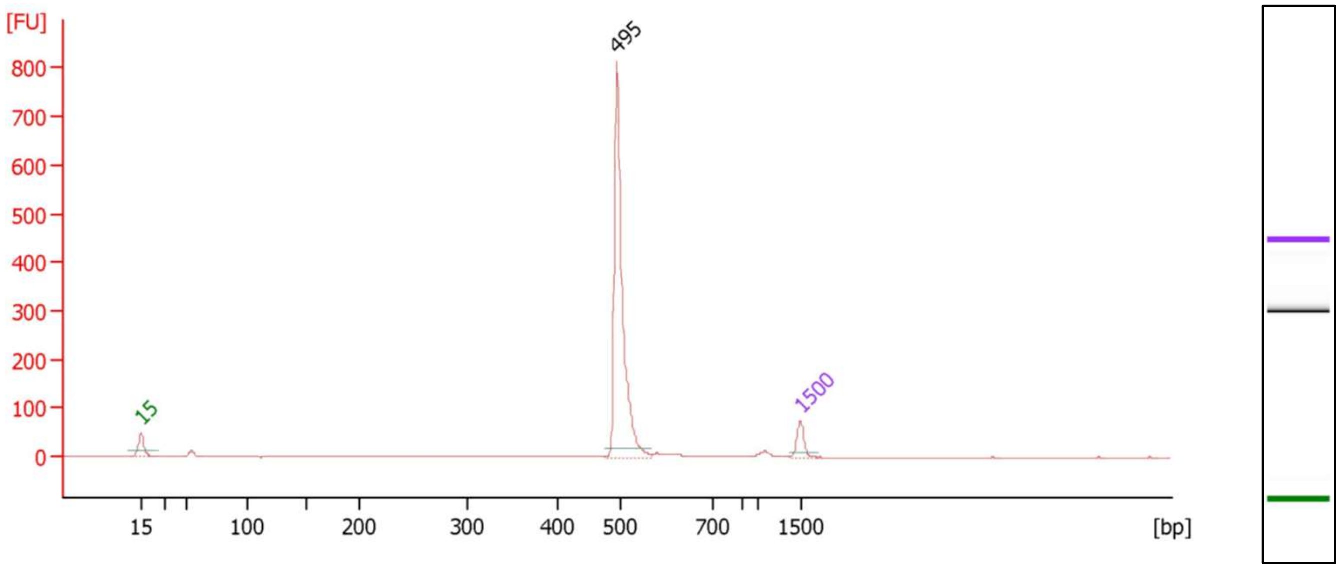

Peak table for Sample #20 :

Peak		Size [bp]	Conc. [ng/µl]	Molarity [nmol/l]	Observations
1	◀	15	4.20	424.4	Lower Marker
2	L	495	22.23	40.1	Ladder Peak
3	▶	1,500	2.10	2.1	Upper Marker

© Copyright 2003 - 2009 Agilent Technologies, Inc.

Assay Class: DNA 1000
Data Path: C:\...-30\2100 expert_DNA 1000_DE34903611_2023-03-06_NTS_11_20.xad

Created: 3/08/2023 11:25:12 AM
Modified: 3/08/2023 11:25:12 AM

Electrophoresis File Run Summary

CTRL

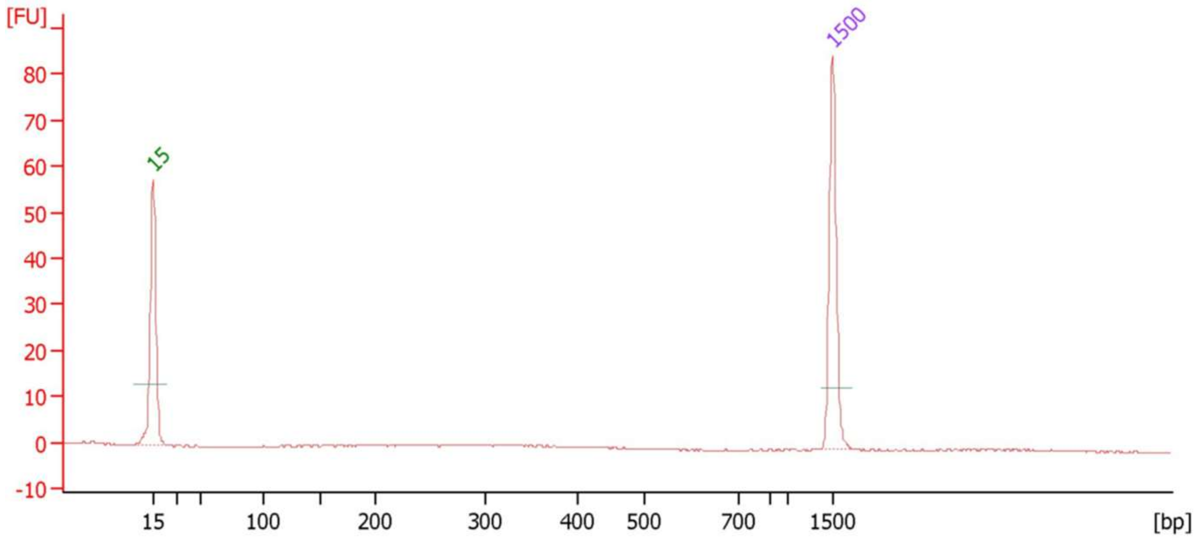

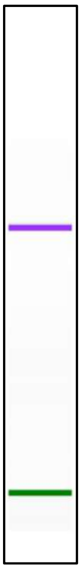

Peak table for Sample CTRL :

Peak		Size [bp]	Conc. [ng/µl]	Molarity [nmol/l]	Observations
1	◀	15	4.20	424.4	Lower Marker
2	▶	1,500	2.10	2.2	Upper Marker

 © Copyright 2003 - 2009 Agilent Technologies, Inc.

Assay Class: DNA 1000
Data Path: C:\...-30\2100 expert_DNA 1000_DE34903611_2023-03-06_NTS_11_20.xad

Created: 3/08/2023 11:25:12 AM
Modified: 3/08/2023 11:25:12 AM

Electrophoresis File Run Summary

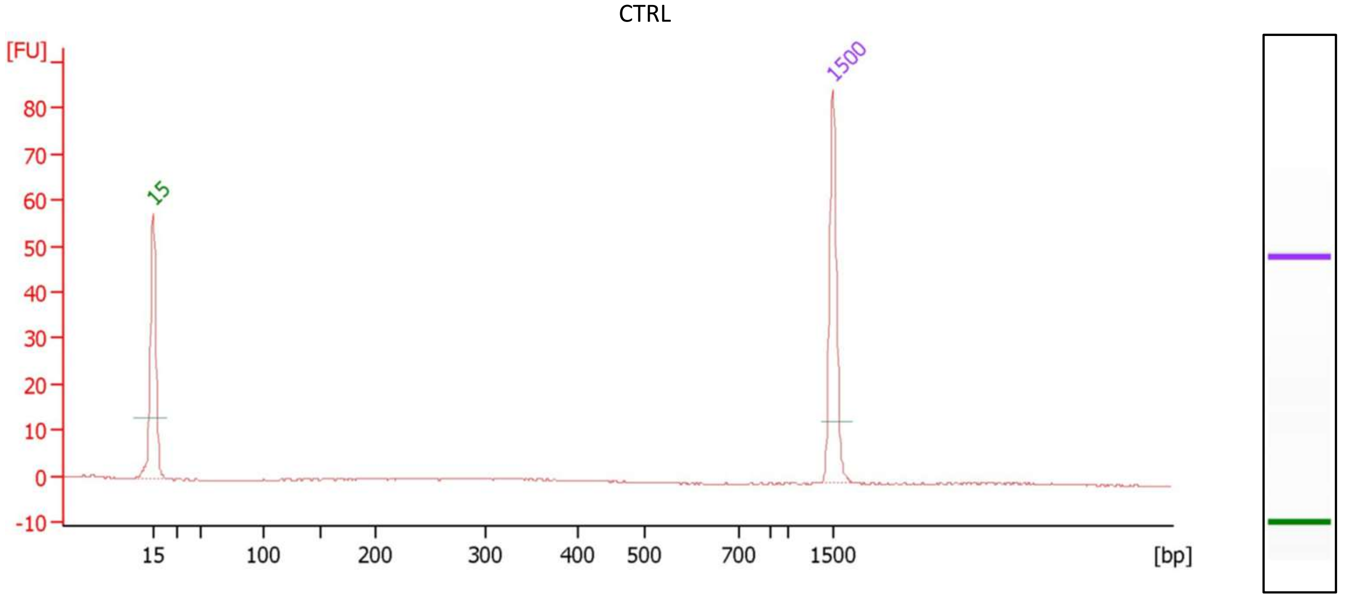

Peak table for Sample CTRL :

Peak		Size [bp]	Conc. [ng/µl]	Molarity [nmol/l]	Observations
1	◀	15	4.20	424.4	Lower Marker
2	▶	1,500	2.10	2.1	Upper Marker

 © Copyright 2003 - 2009 Agilent Technologies, Inc.

Assay Class: DNA 1000
Data Path: C:\...-30\2100 expert_DNA 1000_DE34903611_2023-03-06_NTS_11_20.xad
Created: 3/08/2023 11:25:12 AM
Modified: 3/08/2023 11:25:12 AM

Curves

Standard Curve

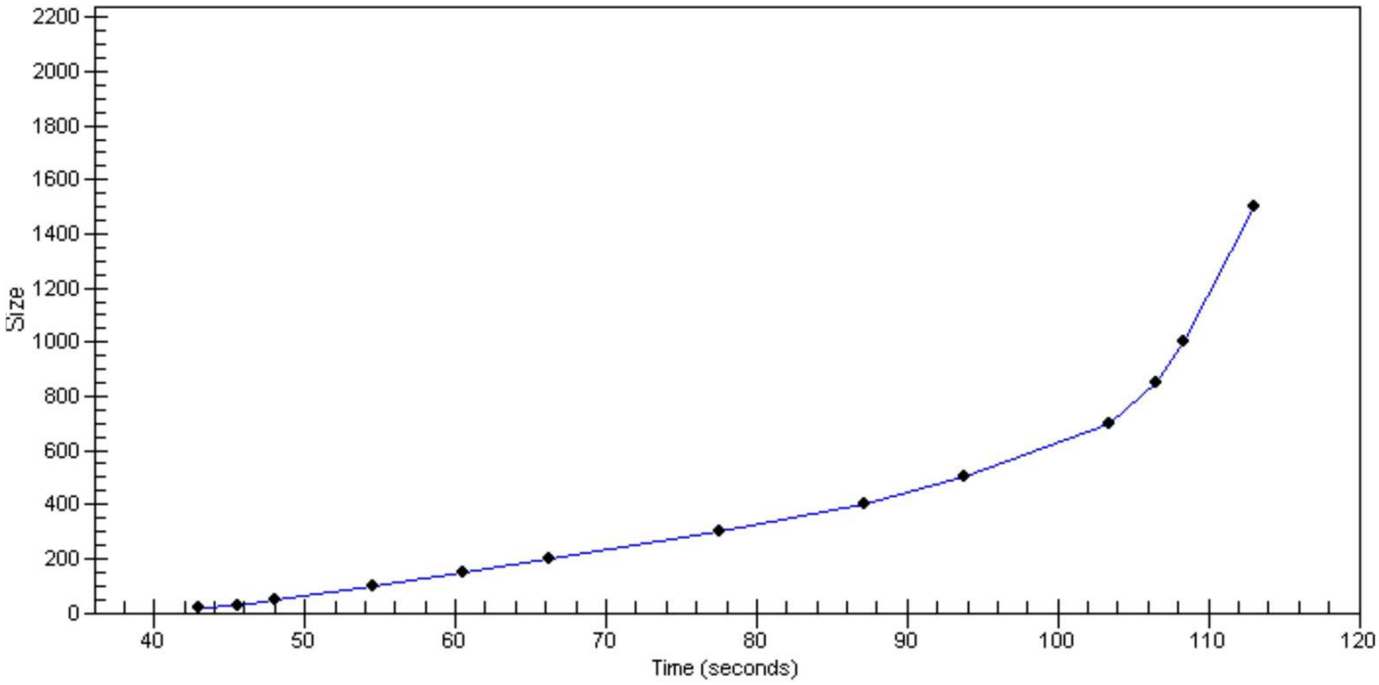

© Copyright 2003 - 2009 Agilent Technologies, Inc.

Assay Class: DNA 1000
Data Path: C:\...-30\2100 expert_DNA 1000_DE34903611_2023-03-06_NTS_11_20.xad
Created: 3/08/2023 11:25:12 AM
Modified: 3/08/2023 11:25:12 AM

Electrophoresis File Run Summary (Chip Summary)

Sample Name	Sample Content	Rest. Digest	Status	Observation	Result Label	Result Color
Sample #1		☐	✔			
Sample #2		☐	✔			
Sample #3		☐	✔			
Sample #4		☐	✔			
Sample #5		☐	✔			
Sample #6		☐	✔			
Sample #7		☐	✔			
Sample #8		☐	✔			
Sample #9		☐	✔			
Sample #10		☐	✔			
CTRL		☐	✔			
CTRL		☐	✔			

Chip Lot # **Reagent Kit Lot #**

Chip Comments :

© Copyright 2003 - 2009 Agilent Technologies, Inc.

Assay Class: DNA 1000
Data Path: C:\...-30\2100 expert_DNA 1000_DE34903611_2023-03-06_NTS_11_20.xad
Created: 3/08/2023 11:25:12 AM
Modified: 3/08/2023 11:25:12 AM

Electrophoresis Assay Details

General Analysis Settings

Result Label **Result Color**

Number of Available Sample and Ladder Wells (Max.) : 13
Minimum Visible Range [s] : 30
Maximum Visible Range [s] : 129
Start Analysis Time Range [s] : 30
End Analysis Time Range [s] : 128.95
Ladder Concentration [ng/µl] : 44
Uses Standard Area for Ladder Fragments
Lower Marker Concentration [ng/µl] : 4.2
Upper Marker Concentration [ng/µl] : 2.1
Used Upper Marker for Quantitation
Standard Curve Fit is Point to Point
Show Data Aligned to Lower and Upper Marker

Integrator Settings

Integration Start Time [s] : 30
Integration End Time [s] : 128.95
Slope Threshold : 0.5
Height Threshold [FU] : 20
Area Threshold : 0.1
Width Threshold [s] : 0.5
Baseline Plateau [s] : 0.5

Filter Settings

Filter Width [s] : 0.5
Polynomial Order : 4

Ladder

Ladder Peak	Size	Area
1	15	25
2	25	26
3	50	34
4	100	41
5	150	45
6	200	52
7	300	63
8	400	76
9	500	83
10	700	88
11	850	86
12	1000	90
13	1500	52

© Copyright 2003 - 2009 Agilent Technologies, Inc.

Assay Class: DNA 1000
Data Path: C:\...-4903611_2023-03-06_NTS_1_10_XhoI_MseI.xad

Created: 3/14/2023 9:38:10 AM
Modified: 3/14/2023 9:38:10 AM

Electrophoresis File Run Summary

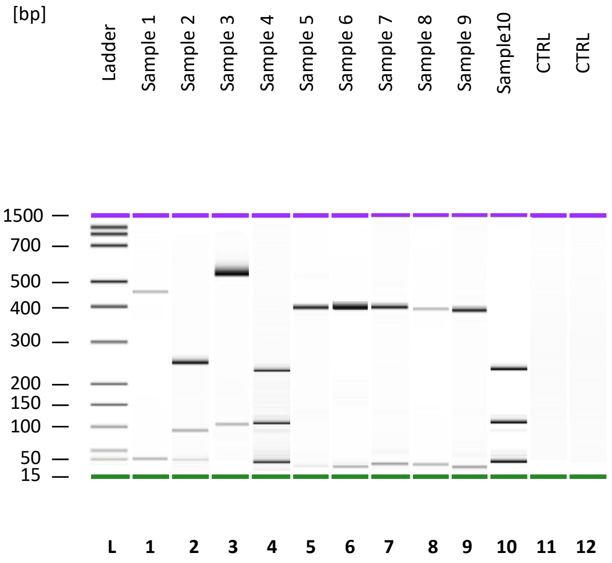

Instrument Information:

Instrument Name: DE34903611

Firmware: C.01.069

Serial #: DE34903611

Type: G2938C

Assay Information:

Assay Origin Path: C:\Program Files\Agilent\2100 bioanalyzer\2100 expert\assays\dsDNA\DNA 1000 Series II.xsy

Assay Class: DNA 1000

Version: 2.3

Assay Comments: DNA Analysis 2 - 1000 bp

© Copyright 2003-2009 Agilent Technologies, Inc.

Chip Information:

Chip Lot #:

Reagent Kit Lot #:

Chip Comments:

Sample #1

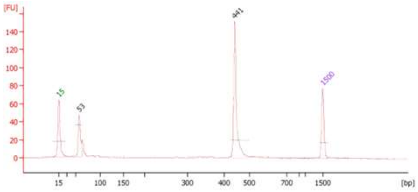

Sample 2

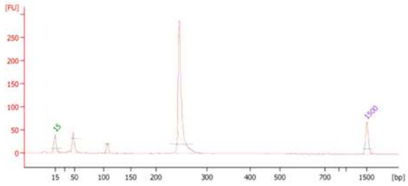

Sample 3

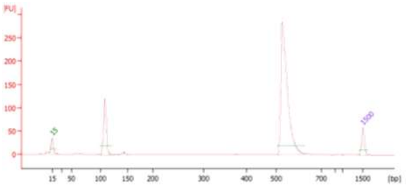

Sample #4

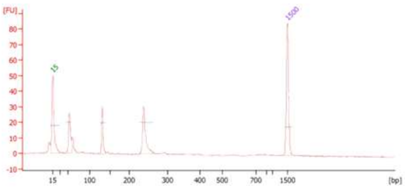

Sample 5

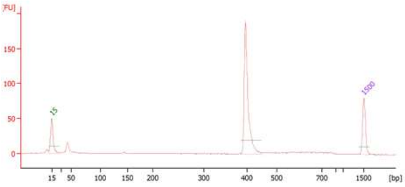

Sample 6

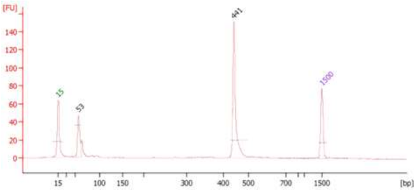

Sample #7

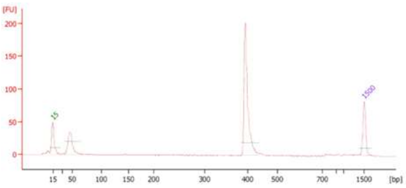

Sample 8

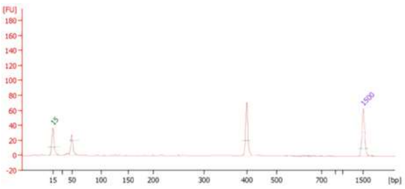

Sample 9

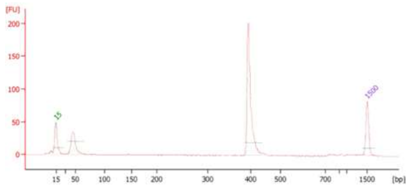

Sample #10

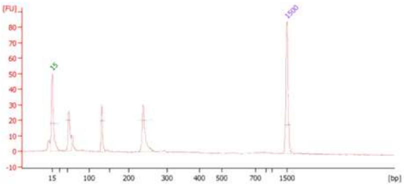

CTRL

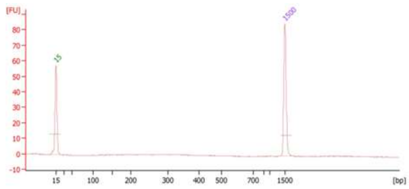

CTRL

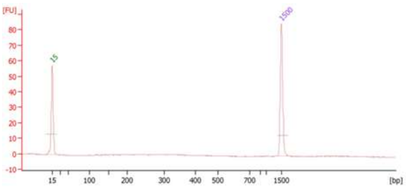

Assay Class: DNA 1000
Data Path: C:\...-4903611_2023-03-06_NTS_1_10_Xhol_MseI.xad

Created: 3/14/2023 9:38:10 AM
Modified: 3/14/2023 9:38:10 AM

Electrophoresis File Run Summary

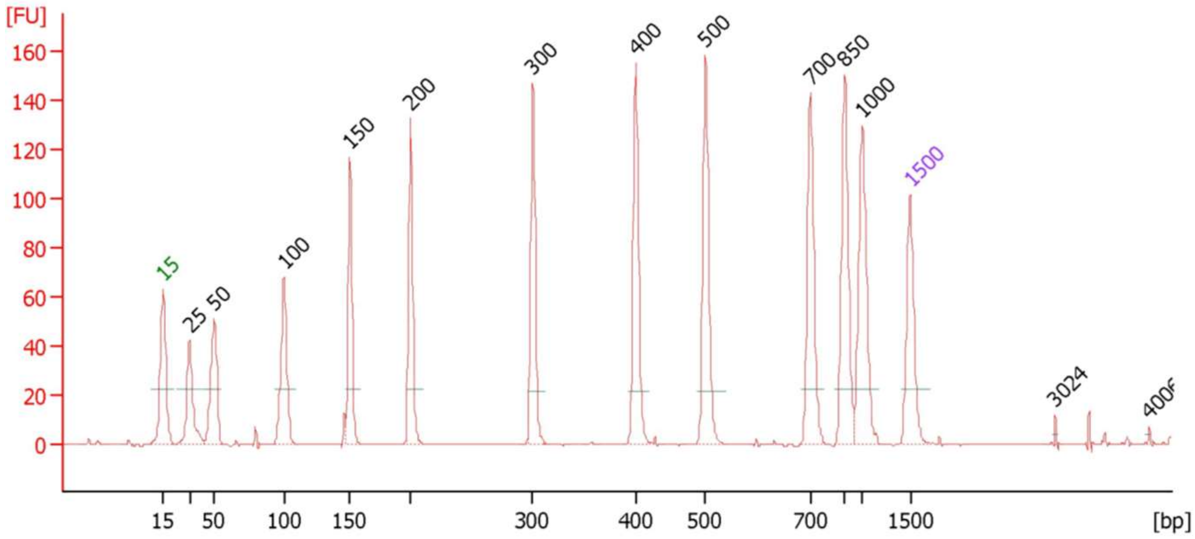

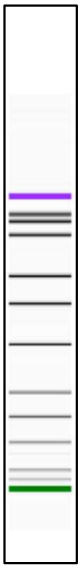

Peak table for Ladder :

Peak	Size [bp]	Conc. [ng/µl]	Molarity [nmol/l]	Observations
1	15	4.20	4.4.2	Lower Marker
2	25	4.00	242.4	Ladder Peak
3	50	4.00	121.2	Ladder Peak
4	100	4.00	60.6	Ladder Peak
5	150	4.00	40.3	Ladder Peak
6	200	4.00	30.3	Ladder Peak
7	300	4.00	20.2	Ladder Peak
8	400	4.00	15.2	Ladder Peak
9	500	4.00	12.1	Ladder Peak
10	700	4.00	8.7	Ladder Peak
11	850	4.00	7.1	Ladder Peak
12	1,000	4.00	6.1	Ladder Peak
13	1,500	2.10	2.1	Upper Marker
14	3,024	0.00	0.00	
15	4,006	0.00	0.00	

 © Copyright 2003 - 2009 Agilent Technologies, Inc.

Assay Class: DNA 1000
Data Path: C:\...-4903611_2023-03-06_NTS_1_10_XhoI_MseI.xad

Created: 3/14/2023 9:38:10 AM
Modified: 3/14/2023 9:38:10 AM

Electrophoresis File Run Summary

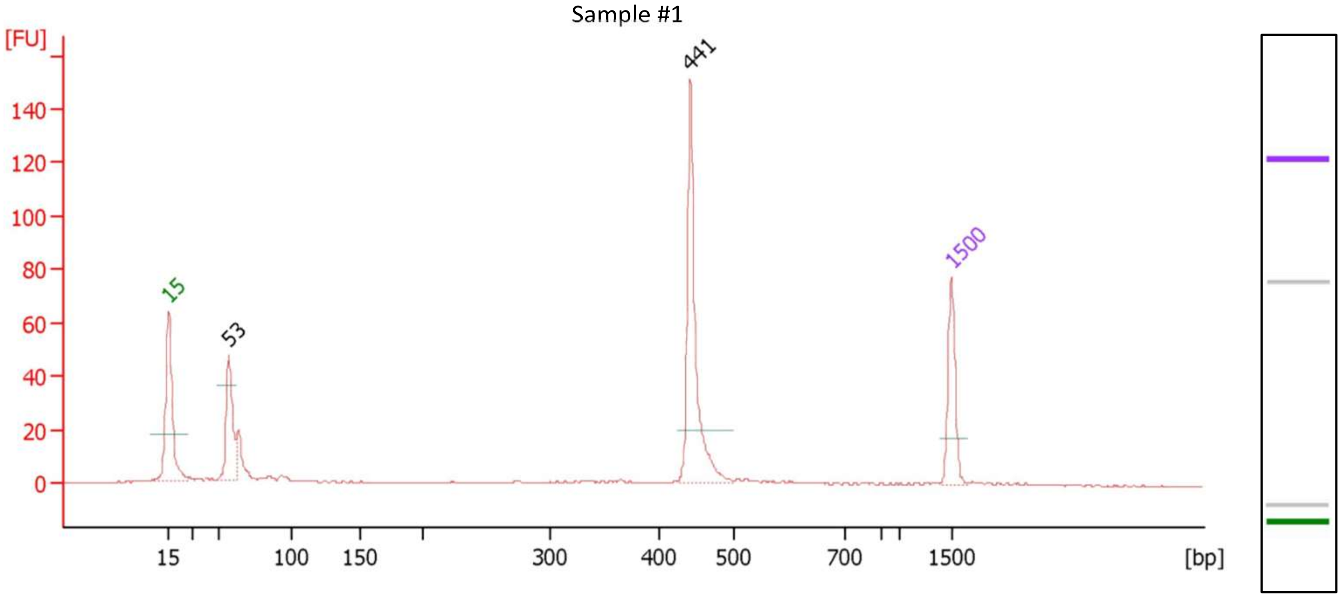

Peak table for Sample #1 :

Peak		Size [bp]	Conc. [ng/µl]	Molarity [nmol/l]	Observations
1		15	4.20	424.4	Lower Marker
2		53	13.97	84.22	Ladder Peak
3		441	27.25	91.33	Ladder Peak
4		1,500	2.10	2.2	Upper Marker

Assay Class: DNA 1000
Data Path: C:\...-4903611_2023-03-06_NTS_1_10_XhoI_MseI.xad

Created: 3/14/2023 9:38:10 AM
Modified: 3/14/2023 9:38:10 AM

Electrophoresis File Run Summary

Sample #2

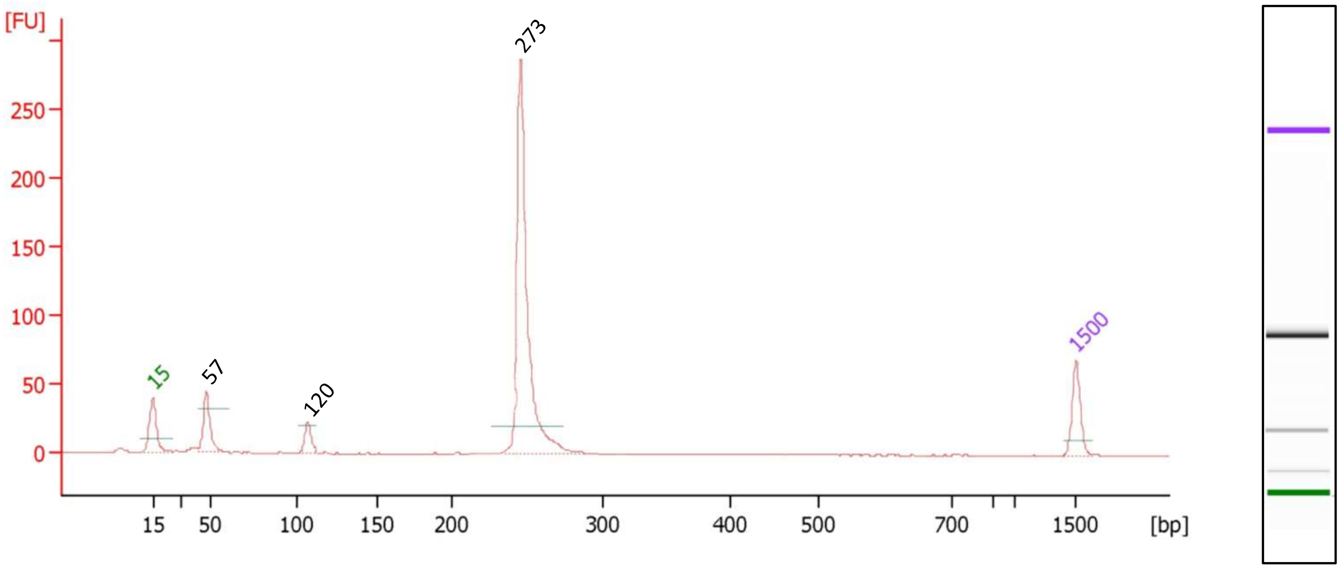

Peak table for Sample #2 :

Peak		Size [bp]	Conc. [ng/µl]	Molarity [nmol/l]	Observations
1	◀	15	4.20	424.4	Lower Marker
2	L	57	3.21	84.12	Ladder Peak
3	L	120	2.23	87.22	Ladder Peak
4	L	273	31.25	95.36	Ladder Peak
5	▶	1,500	2.10	2.2	Upper Marker

 © Copyright 2003 - 2009 Agilent Technologies, Inc.

Assay Class: DNA 1000
Data Path: C:\...-4903611_2023-03-06_NTS_1_10_XhoI_MseI.xad

Created: 3/14/2023 9:38:10 AM
Modified: 3/14/2023 9:38:10 AM

Electrophoresis File Run Summary

Sample #3

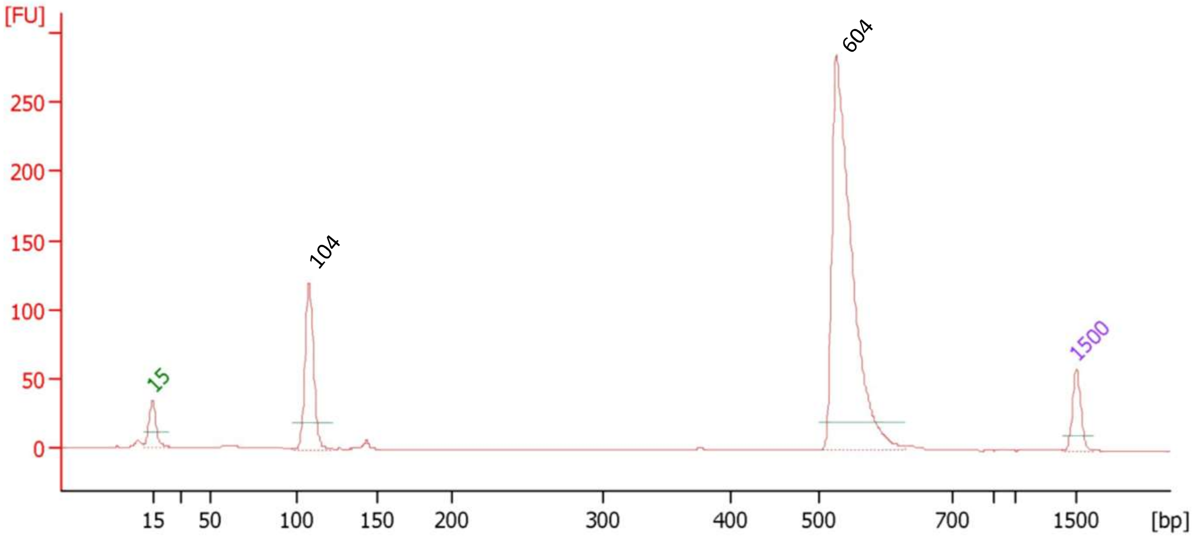

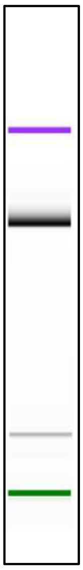

Peak table for Sample #3 :

Peak		Size [bp]	Conc. [ng/µl]	Molarity [nmol/l]	Observations
1		15	4.20	424.4	Lower Marker
2		604	17.21	86.7	Ladder Peak
3		104	11.11	22.51	Ladder Peak
4		1,500	2.10	2.1	Upper Marker

 © Copyright 2003 - 2009 Agilent Technologies, Inc.

Assay Class: DNA 1000
Data Path: C:\...-4903611_2023-03-06_NTS_1_10_XhoI_MseI.xad

Created: 3/14/2023 9:38:10 AM
Modified: 3/14/2023 9:38:10 AM

Electrophoresis File Run Summary

Sample #4

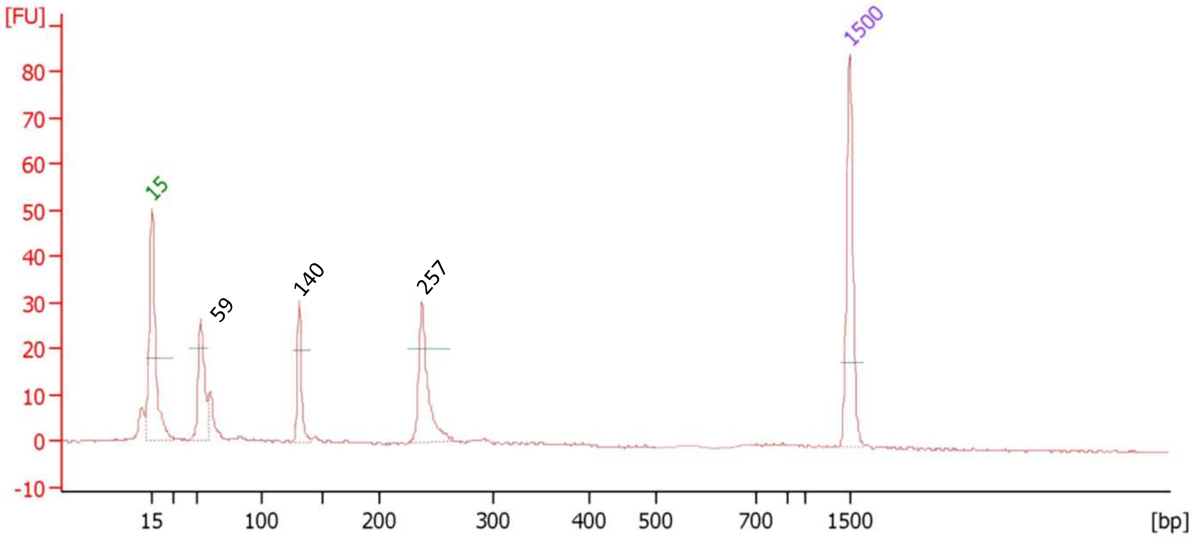

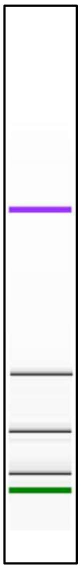

Peak table for Sample #4 :

Peak	Size [bp]	Conc. [ng/µl]	Molarity [nmol/l]	Observations
1	15	4.20	424.2	Lower Marker
2	59	8.98	31.87	Ladder Peak
3	140	7.85	30.69	Ladder Peak
4	257	29.99	105.69	Ladder Peak
5	1,500	2.10	2.1	Upper Marker

© Copyright 2003 - 2009 Agilent Technologies, Inc.

Assay Class: DNA 1000 Created: 3/14/2023 9:38:10 AM
Data Path: C:\...-4903611_2023-03-06_NTS_1_10_XhoI_MseI.xad Modified: 3/14/2023 9:38:10 AM

Electrophoresis File Run Summary

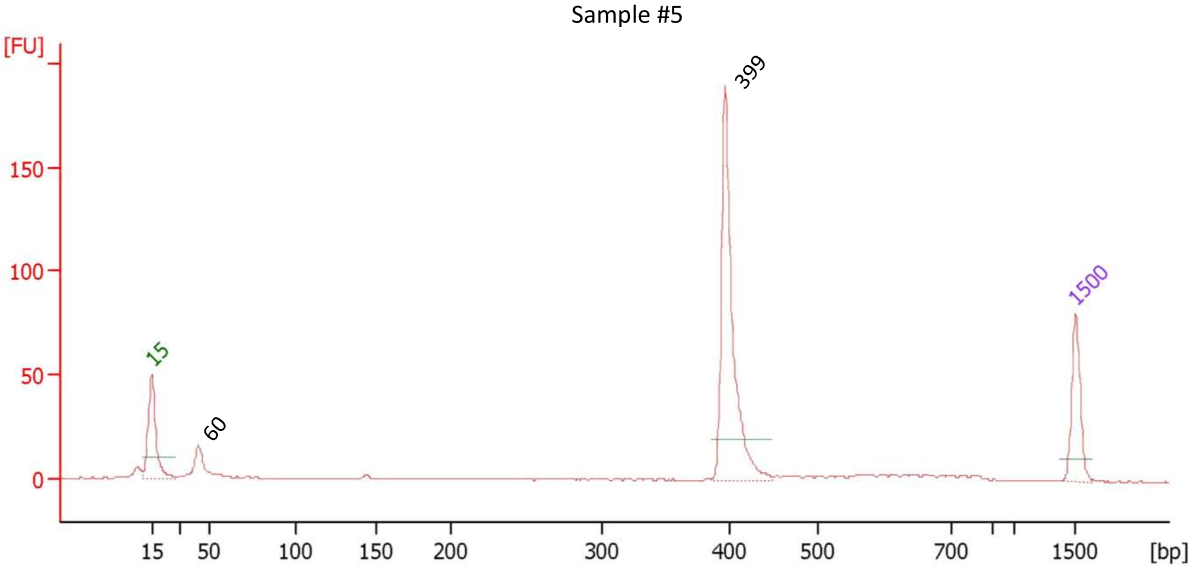

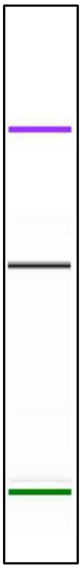

Peak table for Sample #5 :

Peak		Size [bp]	Conc. [ng/µl]	Molarity [nmol/l]	Observations
1	◀	15	4.20	424.2	Lower Marker
2	L	60	1.25	25.6	Ladder Peak
3	L	399	25.34	112.3	Ladder Peak
4	▶	1,500	2.10	2.1	Upper Marker

 © Copyright 2003 - 2009 Agilent Technologies, Inc.

Assay Class: DNA 1000
Data Path: C:\...-4903611_2023-03-06_NTS_1_10_XhoI_MseI.xad

Created: 3/14/2023 9:38:10 AM
Modified: 3/14/2023 9:38:10 AM

Electrophoresis File Run Summary

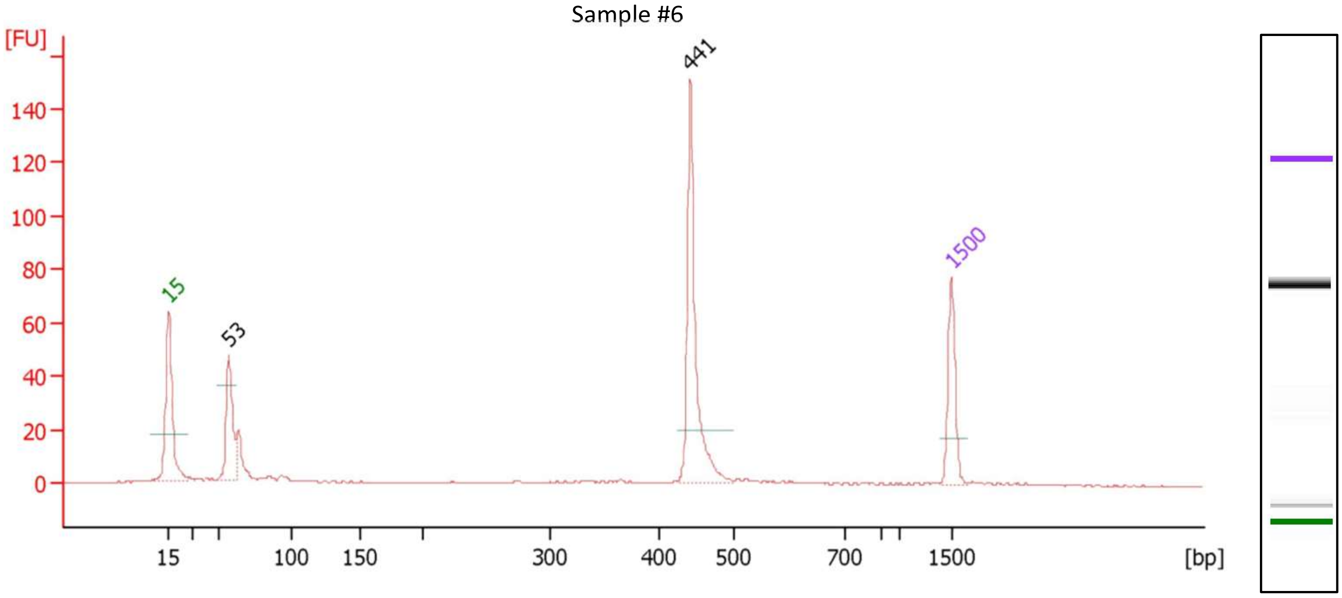

Peak table for Sample #6 :

Peak		Size [bp]	Conc. [ng/µl]	Molarity [nmol/l]	Observations
1		15	4.20	424.4	Lower Marker
2		53	12.32	47.56	Ladder Peak
3		441	25.96	84.52	Ladder Peak
4		1,500	2.10	2.2	Upper Marker

© Copyright 2003 - 2009 Agilent Technologies, Inc.

Assay Class: DNA 1000
Data Path: C:\...-4903611_2023-03-06_NTS_1_10_XhoI_MseI.xad

Created: 3/14/2023 9:38:10 AM
Modified: 3/14/2023 9:38:10 AM

Electrophoresis File Run Summary

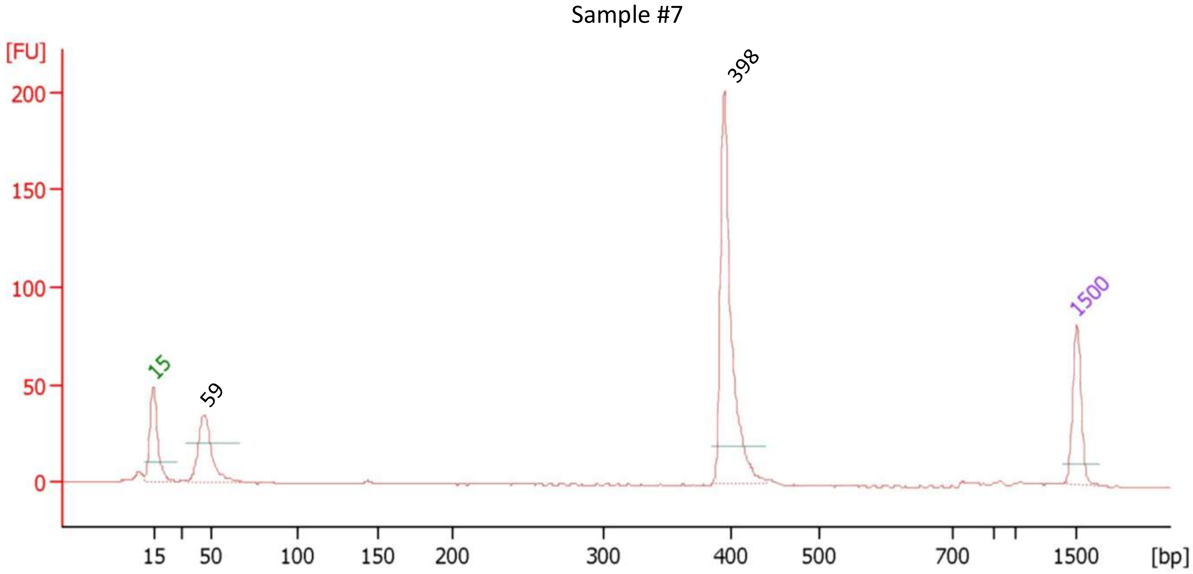

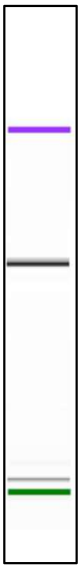

Peak table for Sample #7 :

Peak		Size [bp]	Conc. [ng/µl]	Molarity [nmol/l]	Observations
1	◀	15	4.20	424.2	Lower Marker
2	L	59	3.58	32.9	Ladder Peak
3	L	398	36.87	121.2	Ladder Peak
4	▶	1,500	2.10	2.1	Upper Marker

Assay Class: DNA 1000
Data Path: C:\...-4903611_2023-03-06_NTS_1_10_XhoI_MseI.xad

Created: 3/14/2023 9:38:10 AM
Modified: 3/14/2023 9:38:10 AM

Electrophoresis File Run Summary

Sample #8

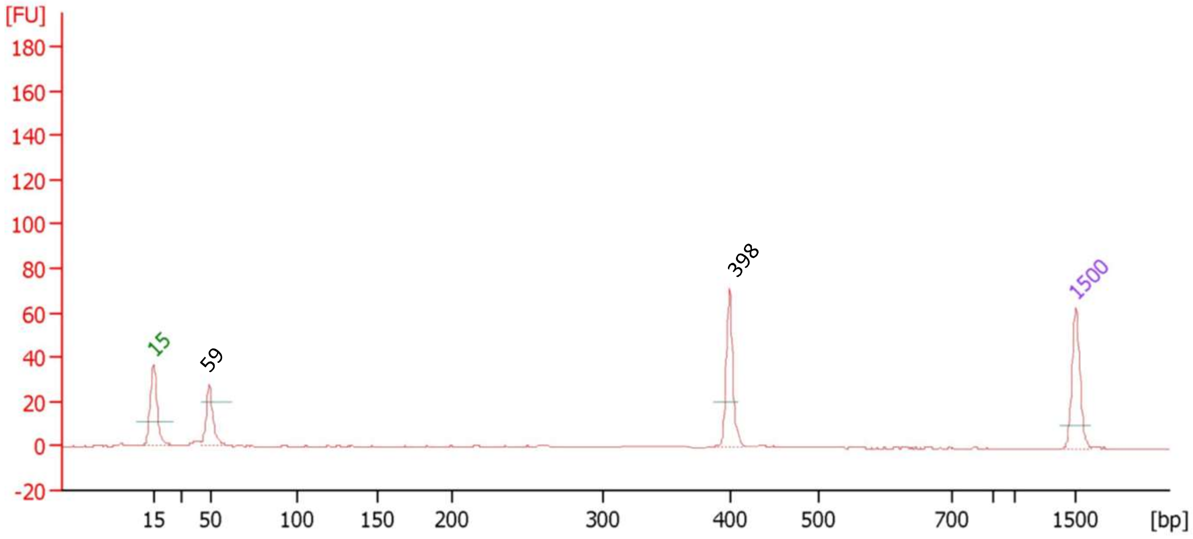

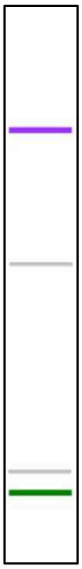

Peak table for Sample #8 :

Peak		Size [bp]	Conc. [ng/µl]	Molarity [nmol/l]	Observations
1	◀	15	4.20	424.4	Lower Marker
2	L	59	5.58	54.8	Ladder Peak
3	L	398	7.85	42.1	Ladder Peak
4	▶	1,500	2.10	2.2	Upper Marker

 © Copyright 2003 - 2009 Agilent Technologies, Inc.

Assay Class: DNA 1000
Data Path: C:\...-4903611_2023-03-06_NTS_1_10_XhoI_MseI.xad

Created: 3/14/2023 9:38:10 AM
Modified: 3/14/2023 9:38:10 AM

Electrophoresis File Run Summary

Sample #9

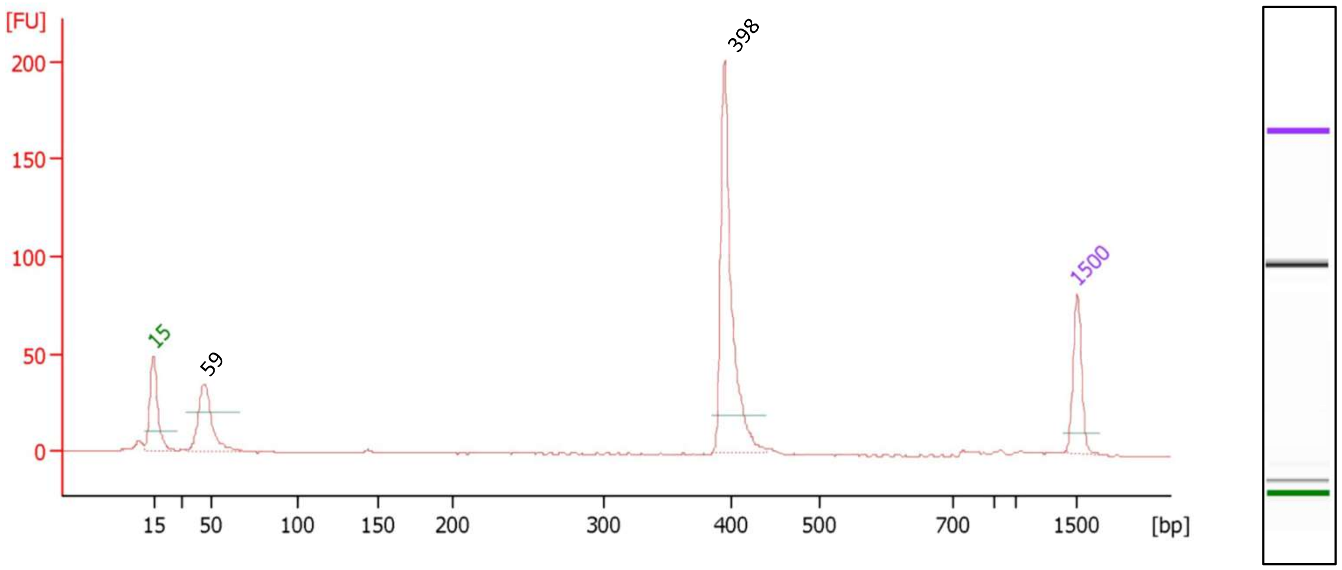

Peak table for Sample #9 :

Peak		Size [bp]	Conc. [ng/µl]	Molarity [nmol/l]	Observations
1	◀	15	4.20	424.2	Lower Marker
2	L	59	3.58	32.9	Ladder Peak
3	L	398	36.87	121.2	Ladder Peak
4	▶	1,500	2.10	2.1	Upper Marker

 © Copyright 2003 - 2009 Agilent Technologies, Inc.

Assay Class: DNA 1000
Data Path: C:\...-4903611_2023-03-06_NTS_1_10_XhoI_MseI.xad

Created: 3/14/2023 9:38:10 AM
Modified: 3/14/2023 9:38:10 AM

Electrophoresis File Run Summary

Sample #10

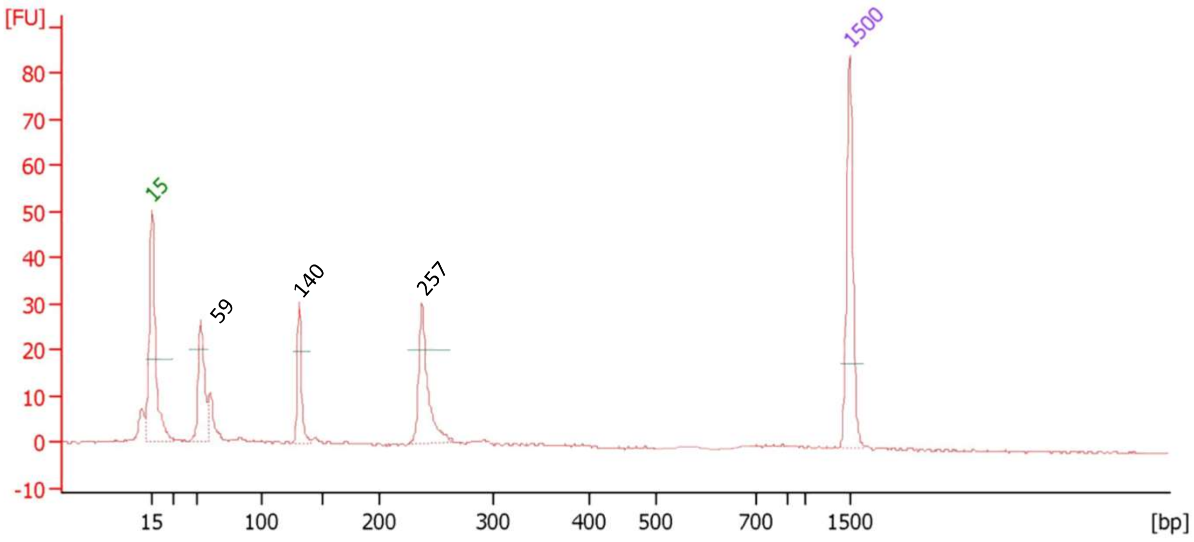

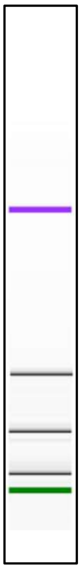

Peak table for Sample #10 :

Peak		Size [bp]	Conc. [ng/µl]	Molarity [nmol/l]	Observations
1		15	4.20	424.2	Lower Marker
2		59	7.41	35.87	Ladder Peak
3		140	8.44	33.69	Ladder Peak
4		257	29.58	102.4	Ladder Peak
5		1,500	2.10	2.1	Upper Marker

 © Copyright 2003 - 2009 Agilent Technologies, Inc.

Assay Class: DNA 1000
Data Path: C:\...-4903611_2023-03-06_NTS_1_10_XhoI_MseI.xad

Created: 3/14/2023 9:38:10 AM
Modified: 3/14/2023 9:38:10 AM

Electrophoresis File Run Summary

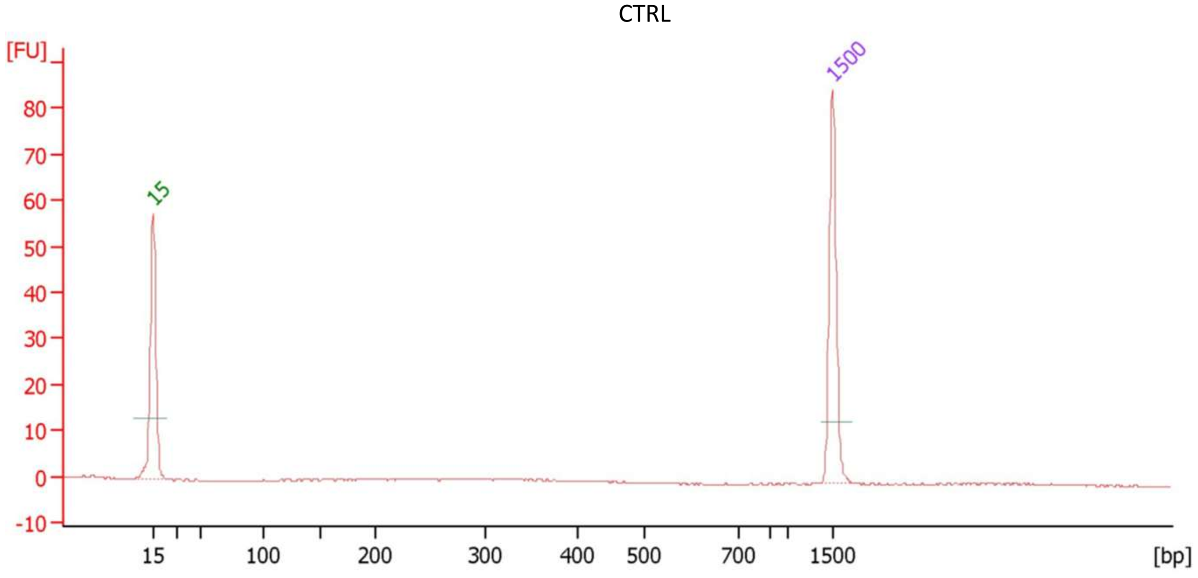

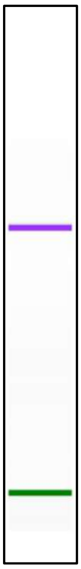

Peak table for Sample CTRL :

Peak		Size [bp]	Conc. [ng/µl]	Molarity [nmol/l]	Observations
**1**	◀	15	4.20	424.4	Lower Marker
**2**	▶	1,500	2.10	2.2	Upper Marker

 © Copyright 2003 - 2009 Agilent Technologies, Inc.

Assay Class: DNA 1000
Data Path: C:\...-4903611_2023-03-06_NTS_1_10_XhoI_MseI.xad

Created: 3/14/2023 9:38:10 AM
Modified: 3/14/2023 9:38:10 AM

Electrophoresis File Run Summary

CTRL

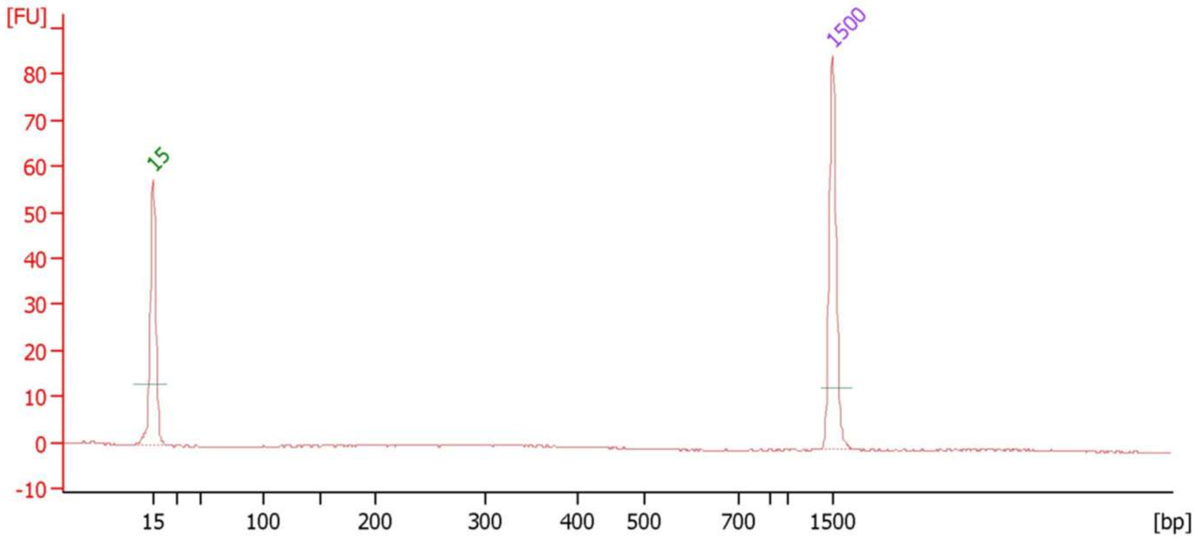

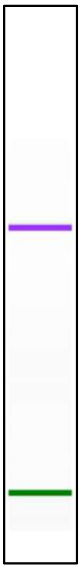

Peak table for Sample CTRL :

Peak		Size [bp]	Conc. [ng/µl]	Molarity [nmol/l]	Observations
1	◀	15	4.20	424.4	Lower Marker
2	▶	1,500	2.10	2.1	Upper Marker

Assay Class: DNA 1000
Data Path: C:\...-4903611_2023-03-06_NTS_1_10_XhoI_MseI.xad

Created: 3/14/2023 9:38:10 AM
Modified: 3/14/2023 9:38:10 AM

Curves

Standard Curve

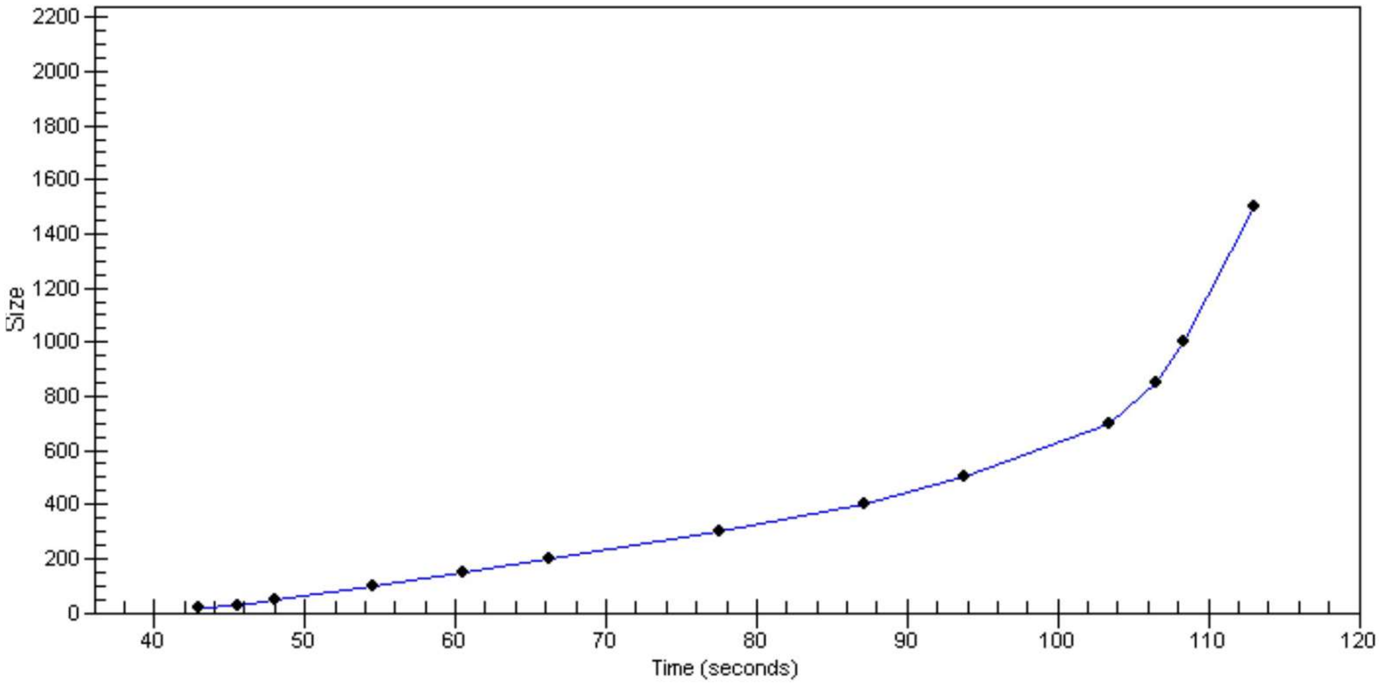

 © Copyright 2003 - 2009 Agilent Technologies, Inc.

Assay Class: DNA 1000
Data Path: C:\...-4903611_2023-03-06_NTS_1_10_Xhol_MseI.xad

Created: 3/14/2023 9:38:10 AM
Modified: 3/14/2023 9:38:10 AM

Electrophoresis File Run Summary (Chip Summary)

Sample Name	Sample Content	Rest. Digest	Status	Observation	Result Label	Result Color
Sample #1		☐	✔			
Sample #2		☐	✔			
Sample #3		☐	✔			
Sample #4		☐	✔			
Sample #5		☐	✔			
Sample #6		☐	✔			
Sample #7		☐	✔			
Sample #8		☐	✔			
Sample #9		☐	✔			
Sample #10		☐	✔			
CTRL		☐	✔			
CTRL		☐	✔			

Chip Lot # **Reagent Kit Lot #**

Chip Comments :

 © Copyright 2003 - 2009 Agilent Technologies, Inc.

Assay Class: DNA 1000
Data Path: C:\...-4903611_2023-03-06_NTS_1_10_Xhol_MseI.xad

Created: 3/14/2023 9:38:10 AM
Modified: 3/14/2023 9:38:10 AM

Electrophoresis Assay Details

General Analysis Settings

Result Label | Result Color

Number of Available Sample and Ladder Wells (Max.) : 13
Minimum Visible Range [s] : 30
Maximum Visible Range [s] : 129
Start Analysis Time Range [s] : 30
End Analysis Time Range [s] : 128.95
Ladder Concentration [ng/µl] : 44
Uses Standard Area for Ladder Fragments
Lower Marker Concentration [ng/µl] : 4.2
Upper Marker Concentration [ng/µl] : 2.1
Used Upper Marker for Quantitation
Standard Curve Fit is Point to Point
Show Data Aligned to Lower and Upper Marker

Integrator Settings

Integration Start Time [s] : 30
Integration End Time [s] : 128.95
Slope Threshold : 0.5
Height Threshold [FU] : 20
Area Threshold : 0.1
Width Threshold [s] : 0.5
Baseline Plateau [s] : 0.5

Filter Settings

Filter Width [s] : 0.5
Polynomial Order : 4

Ladder

Ladder Peak	Size	Area
1	15	25
2	25	26
3	50	34
4	100	41
5	150	45
6	200	52
7	300	63
8	400	76
9	500	83
10	700	88
11	850	86
12	1000	90
13	1500	52

 © Copyright 2003 - 2009 Agilent Technologies, Inc.

Assay Class: DNA 1000
Data Path: C:\...-4903611_2023-03-06_NTS_11_20_XhoI_MseI.xad

Created: 3/14/2023 13:38:45 PM
Modified: 3/14/2023 13:38:45 PM

Electrophoresis File Run Summary

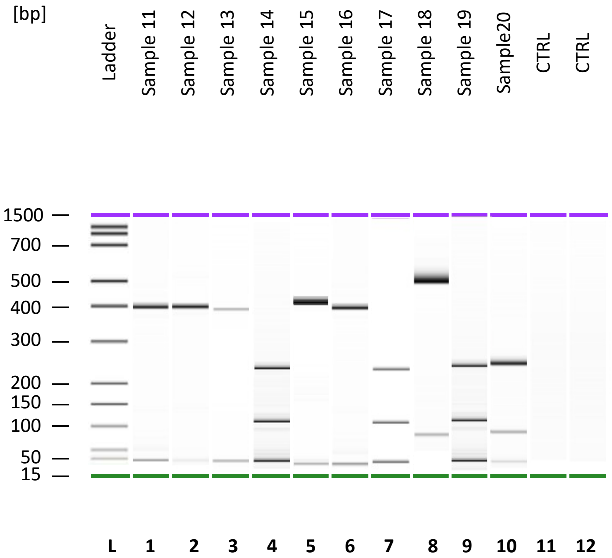

Instrument Information:

Instrument Name: DE34903611
Firmware: C.01.069
Serial #: DE34903611
Type: G2938C

Assay Information:

Assay Origin Path: C:\Program Files\Agilent\2100 bioanalyzer\2100 expert\assays\dsDNA\DNA 1000 Series II.xsy

Assay Class: DNA 1000

Version: 2.3

Assay Comments: DNA Analysis 2 - 1000 bp

© Copyright 2003-2009 Agilent Technologies, Inc.

Chip Information:

Chip Lot #:

Reagent Kit Lot #:

Chip Comments:

Sample #11

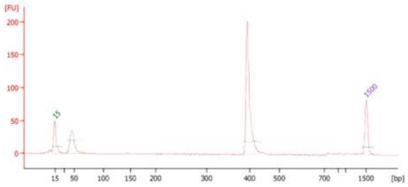

Sample 12

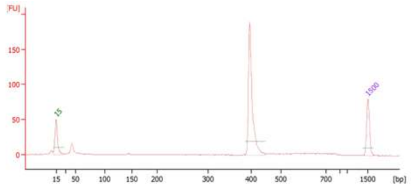

Sample 13

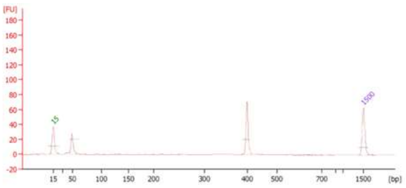

Sample #14

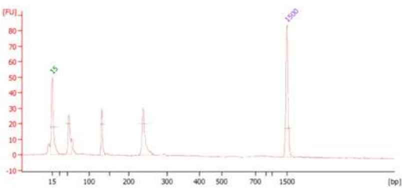

Sample 15

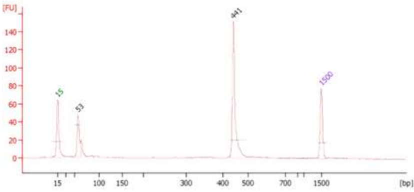

Sample 16

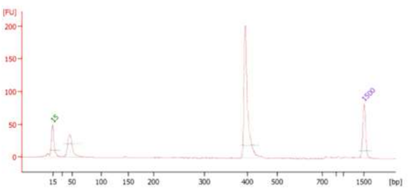

Sample #17

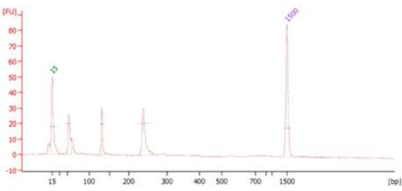

Sample 18

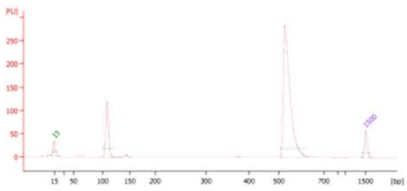

Sample 19

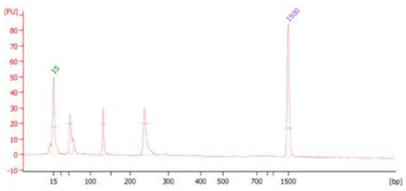

Sample #20

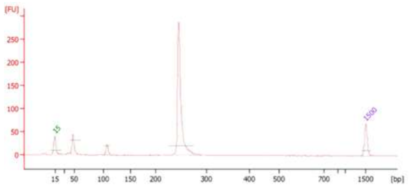

CTRL

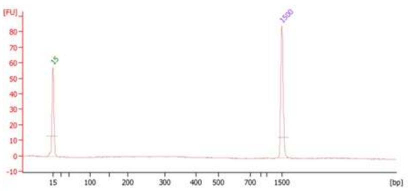

CTRL

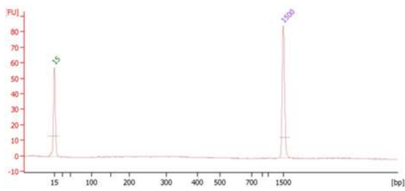

Assay Class: DNA 1000
Data Path: C:\...-4903611_2023-03-06_NTS_11_20_XhoI_MseI.xad

Created: 3/14/2023 13:38:45 PM
Modified: 3/14/2023 13:38:45 PM

Electrophoresis File Run Summary

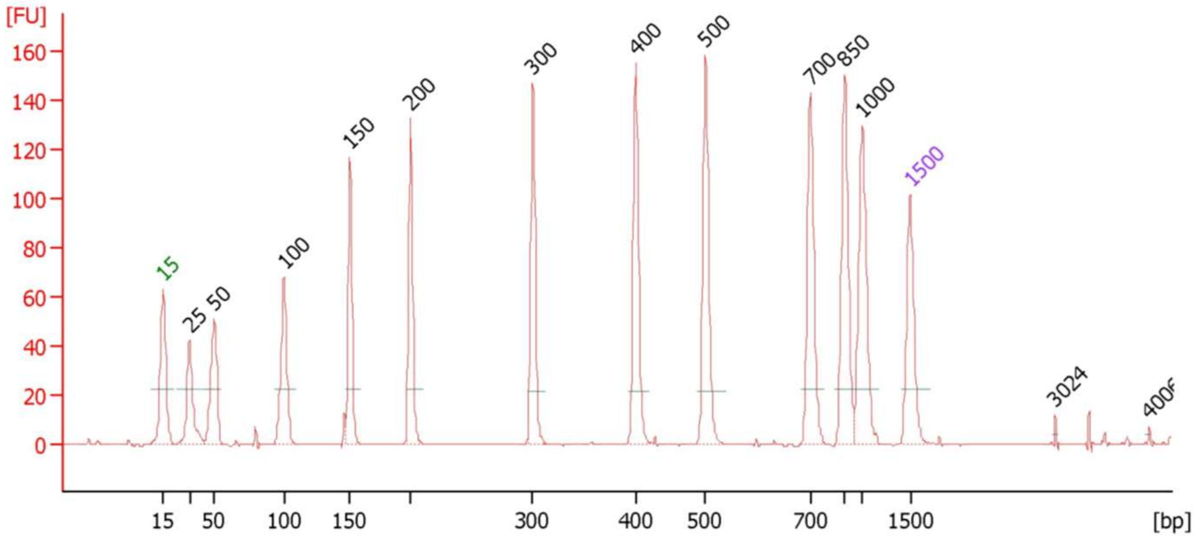

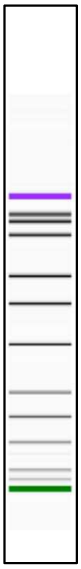

Peak table for Ladder :

Peak		Size [bp]	Conc. [ng/µl]	Molarity [nmol/l]	Observations
1	◀	15	4.20	4.4.2	Lower Marker
2	L	25	4.00	242.4	Ladder Peak
3	L	50	4.00	121.2	Ladder Peak
4	L	100	4.00	60.6	Ladder Peak
5	L	150	4.00	40.3	Ladder Peak
6	L	200	4.00	30.3	Ladder Peak
7	L	300	4.00	20.2	Ladder Peak
8	L	400	4.00	15.2	Ladder Peak
9	L	500	4.00	12.1	Ladder Peak
10	L	700	4.00	8.7	Ladder Peak
11	L	850	4.00	7.1	Ladder Peak
12	L	1,000	4.00	6.1	Ladder Peak
13	▶	1,500	2.10	2.1	Upper Marker
14		3,024	0.00	0.00	
15		4,006	0.00	0.00	

 © Copyright 2003 - 2009 Agilent Technologies, Inc.

Assay Class: DNA 1000
Data Path: C:\...-4903611_2023-03-06_NTS_11_20_XhoI_MseI.xad

Created: 3/14/2023 13:38:45 PM
Modified: 3/14/2023 13:38:45 PM

Electrophoresis File Run Summary

Sample #11

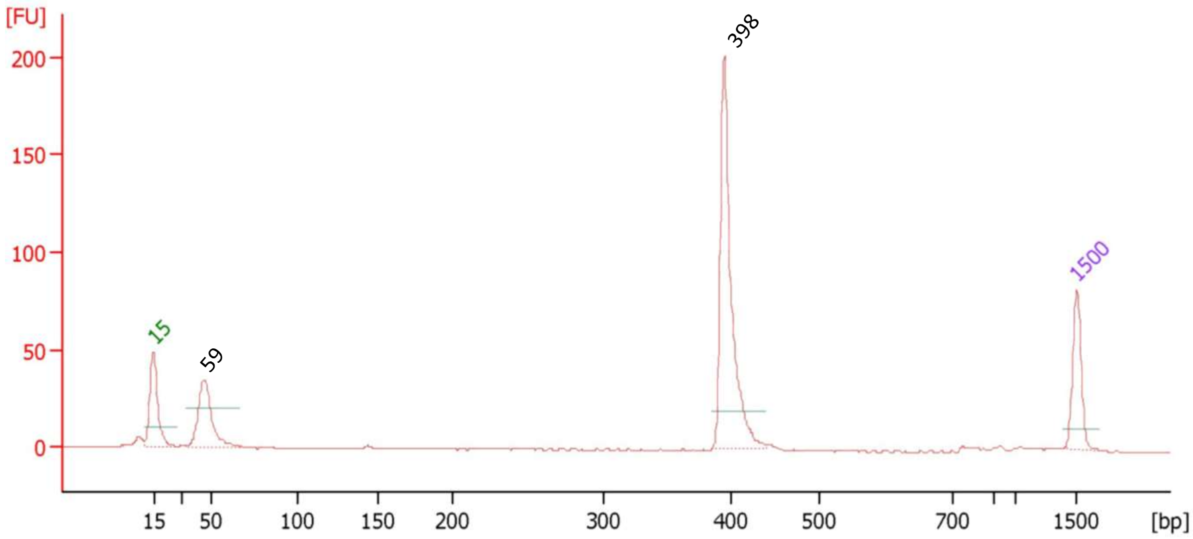

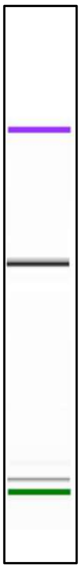

Peak table for Sample #11 :

Peak		Size [bp]	Conc. [ng/µl]	Molarity [nmol/l]	Observations
1		15	4.20	424.2	Lower Marker
2		59	3.58	32.9	Ladder Peak
3		398	36.87	121.2	Ladder Peak
4		1,500	2.10	2.1	Upper Marker

Assay Class: DNA 1000
Data Path: C:\...-4903611_2023-03-06_NTS_11_20_XhoI_MseI.xad

Created: 3/14/2023 13:38:45 PM
Modified: 3/14/2023 13:38:45 PM

Electrophoresis File Run Summary

Sample #12

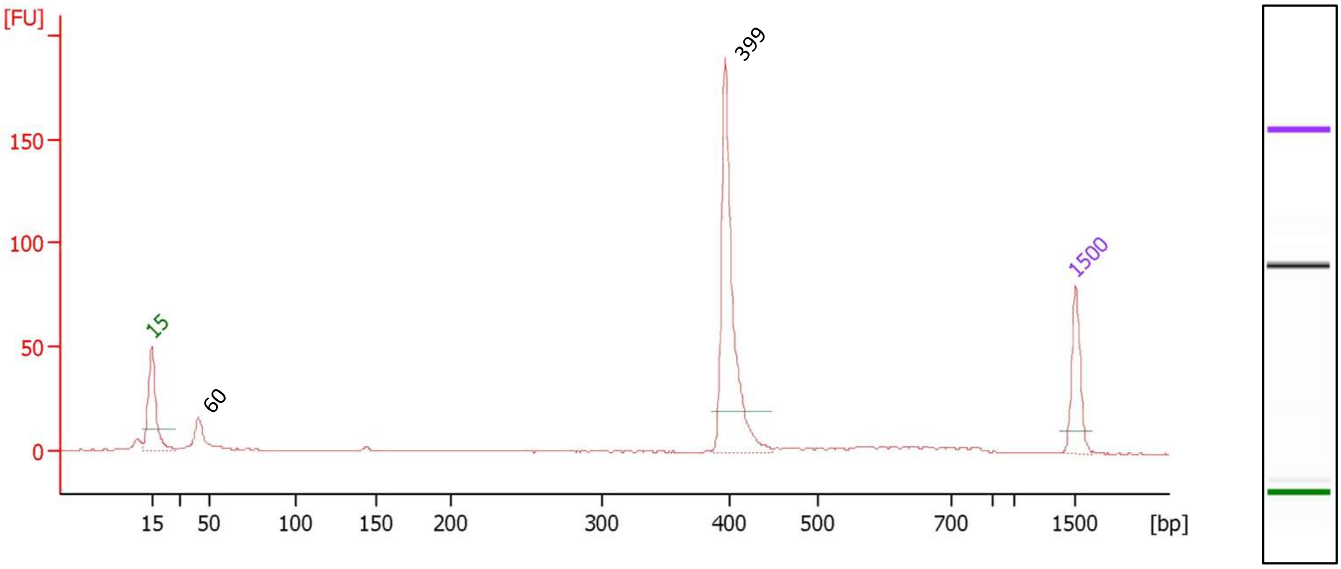

Peak table for Sample #12 :

Peak		Size [bp]	Conc. [ng/µl]	Molarity [nmol/l]	Observations
1	◀	15	4.20	424.2	Lower Marker
2	L	60	1.25	25.6	Ladder Peak
3	L	399	25.34	112.3	Ladder Peak
4	▶	1,500	2.10	2.1	Upper Marker

© Copyright 2003 - 2009 Agilent Technologies, Inc.

Assay Class: DNA 1000
Data Path: C:\...-4903611_2023-03-06_NTS_11_20_XhoI_MseI.xad

Created: 3/14/2023 13:38:45 PM
Modified: 3/14/2023 13:38:45 PM

Electrophoresis File Run Summary

Sample #13

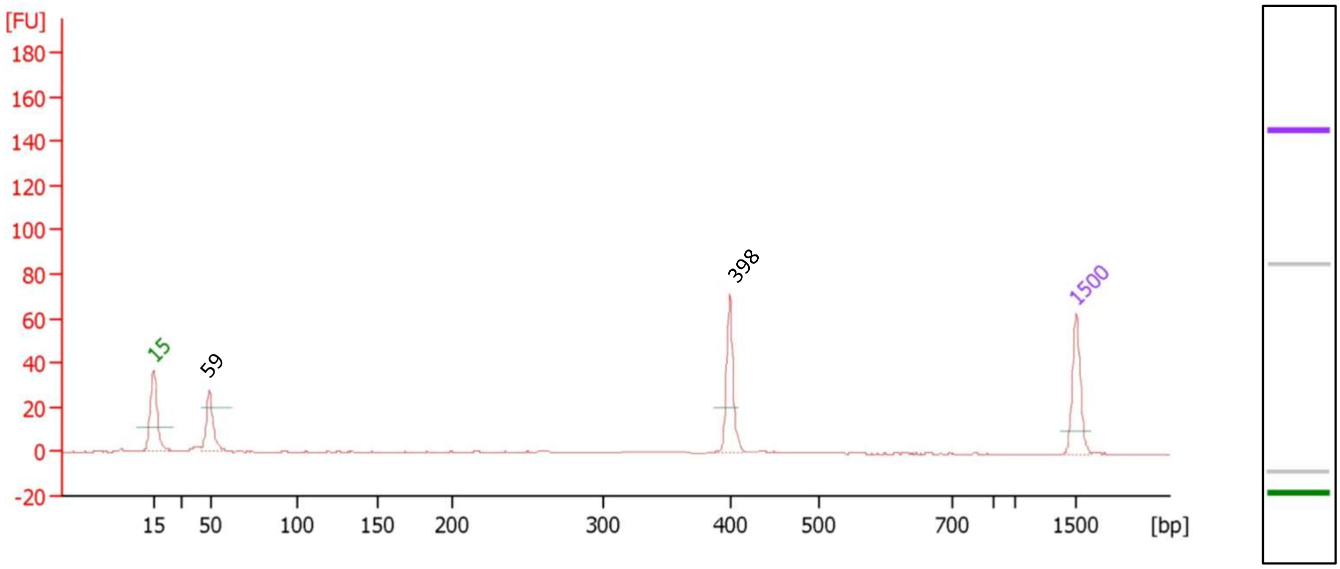

Peak table for Sample #13 :

Peak		Size [bp]	Conc. [ng/µl]	Molarity [nmol/l]	Observations
1	◀	15	4.20	424.4	Lower Marker
2	L	59	5.58	54.8	Ladder Peak
3	L	398	7.85	42.1	Ladder Peak
4	▶	1,500	2.10	2.2	Upper Marker

© Copyright 2003 - 2009 Agilent Technologies, Inc.

Assay Class: DNA 1000
Data Path: C:\...-4903611_2023-03-06_NTS_11_20_XhoI_MseI.xad

Created: 3/14/2023 13:38:45 PM
Modified: 3/14/2023 13:38:45 PM

Electrophoresis File Run Summary

Sample #14

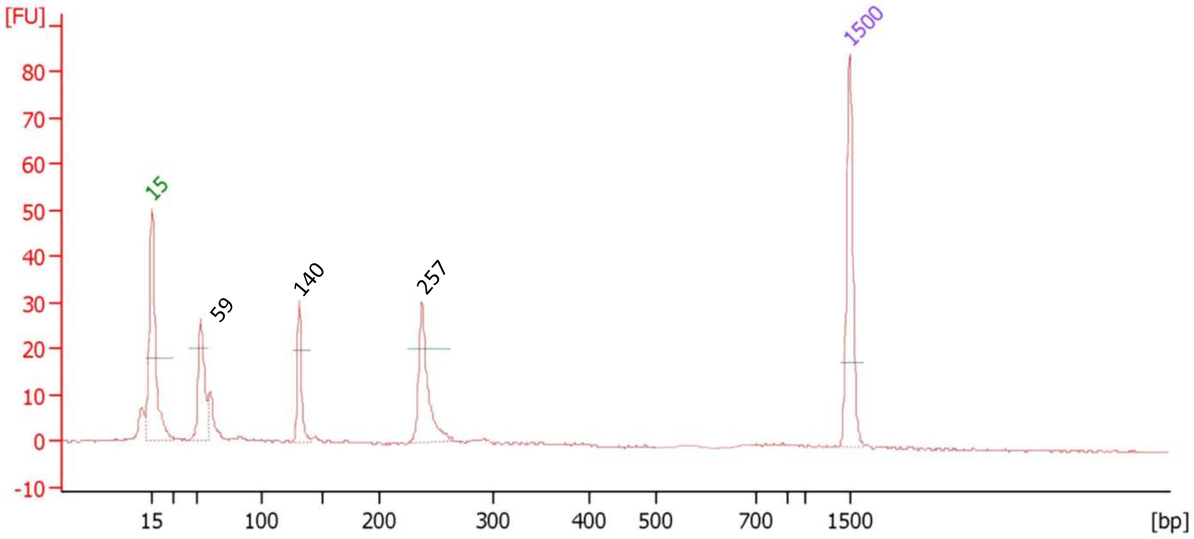

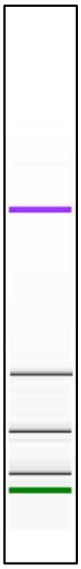

Peak table for Sample #14 :

Peak		Size [bp]	Conc. [ng/µl]	Molarity [nmol/l]	Observations
1		15	4.20	424.2	Lower Marker
2		59	7.41	27.85	Ladder Peak
3		140	8.84	33.69	Ladder Peak
4		257	30.12	87.41	Ladder Peak
5		1,500	2.10	2.1	Upper Marker

 © Copyright 2003 - 2009 Agilent Technologies, Inc.

Assay Class: DNA 1000
Data Path: C:\...-4903611_2023-03-06_NTS_11_20_XhoI_MseI.xad

Created: 3/14/2023 13:38:45 PM
Modified: 3/14/2023 13:38:45 PM

Electrophoresis File Run Summary

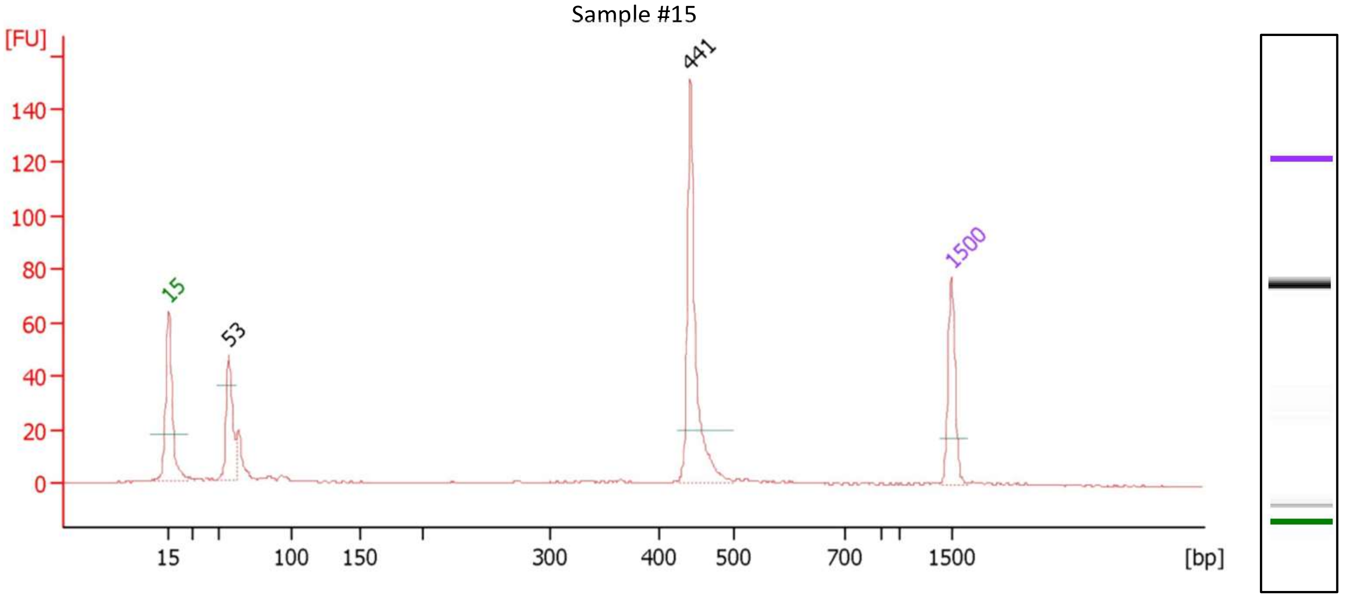

Peak table for Sample #15 :

Peak		Size [bp]	Conc. [ng/µl]	Molarity [nmol/l]	Observations
1	◀	15	4.20	424.4	Lower Marker
2	L	53	12.58	81.2	Ladder Peak
3	L	441	25.55	99.6	Ladder Peak
4	▶	1,500	2.10	2.2	Upper Marker

 © Copyright 2003 - 2009 Agilent Technologies, Inc.

Assay Class: DNA 1000
Data Path: C:\...-4903611_2023-03-06_NTS_11_20_XhoI_MseI.xad

Created: 3/14/2023 13:38:45 PM
Modified: 3/14/2023 13:38:45 PM

Electrophoresis File Run Summary

Sample #16

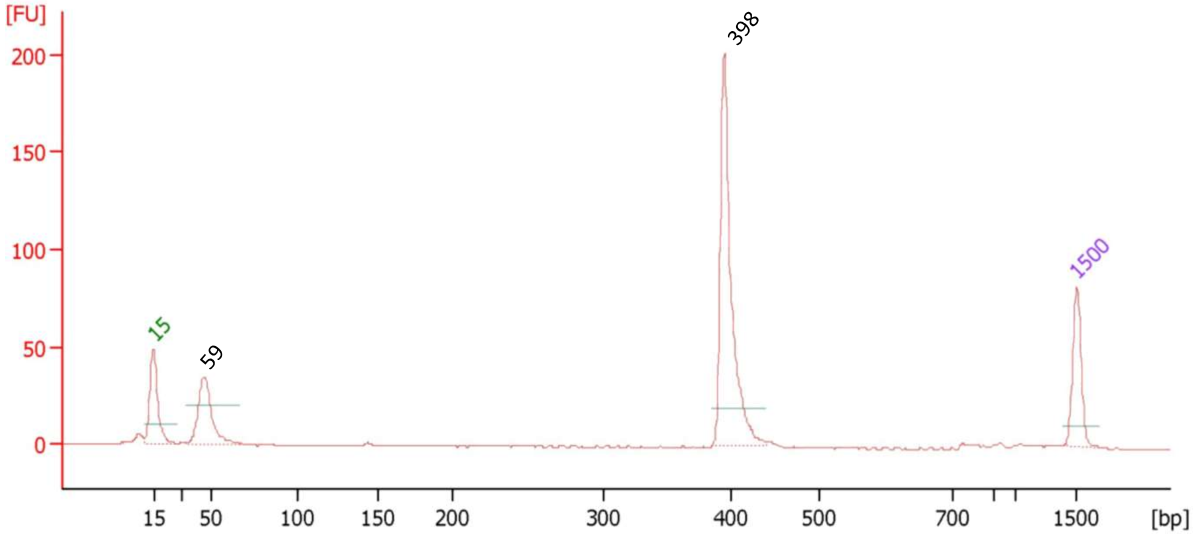

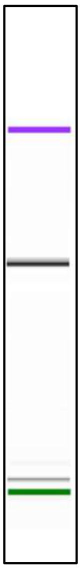

Peak table for Sample #16 :

Peak		Size [bp]	Conc. [ng/µl]	Molarity [nmol/l]	Observations
1	◀	15	4.20	424.2	Lower Marker
2	L	59	3.58	32.9	Ladder Peak
3	L	398	36.87	121.2	Ladder Peak
4	▶	1,500	2.10	2.1	Upper Marker

 © Copyright 2003 - 2009 Agilent Technologies, Inc.

Assay Class: DNA 1000
Data Path: C:\...-4903611_2023-03-06_NTS_11_20_XhoI_MseI.xad

Created: 3/14/2023 13:38:45 PM
Modified: 3/14/2023 13:38:45 PM

Electrophoresis File Run Summary

Sample #17

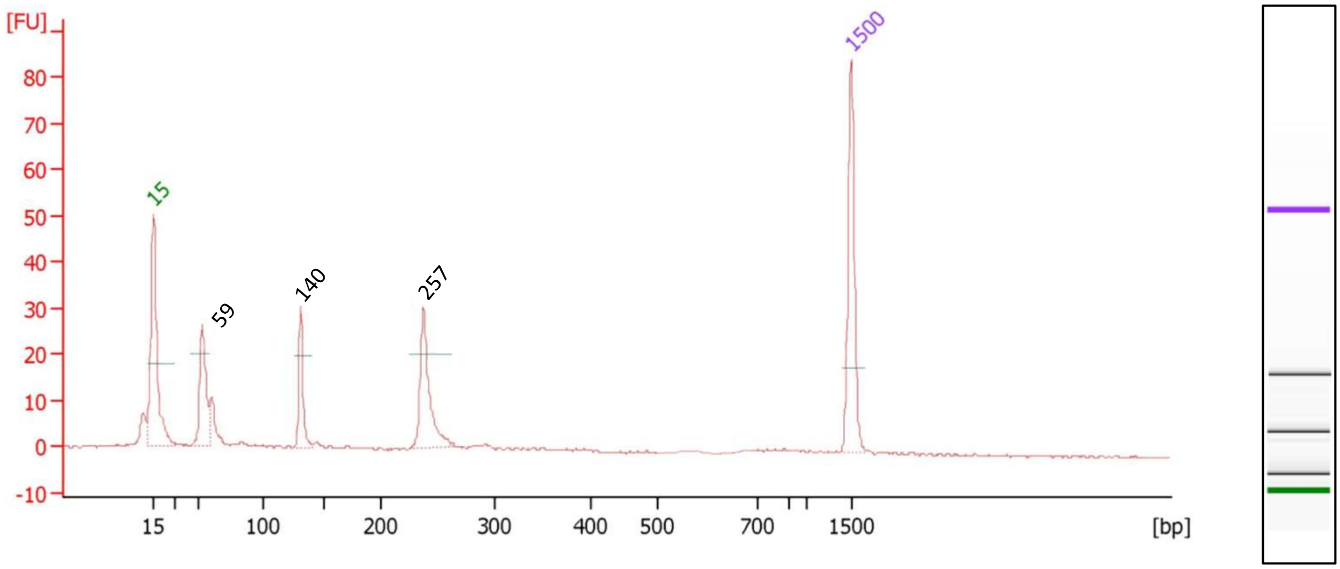

Peak table for Sample #17 :

Peak		Size [bp]	Conc. [ng/µl]	Molarity [nmol/l]	Observations
1	◀	15	4.20	424.2	Lower Marker
2	L	59	7.58	31.2	Ladder Peak
3	L	140	8.47	31.2	Ladder Peak
4	L	257	29.58	99.3	Ladder Peak
5	▶	1,500	2.10	2.1	Upper Marker

 © Copyright 2003 - 2009 Agilent Technologies, Inc.

Assay Class: DNA 1000
Data Path: C:\...-4903611_2023-03-06_NTS_11_20_XhoI_MseI.xad

Created: 3/14/2023 13:38:45 PM
Modified: 3/14/2023 13:38:45 PM

Electrophoresis File Run Summary

Sample #18

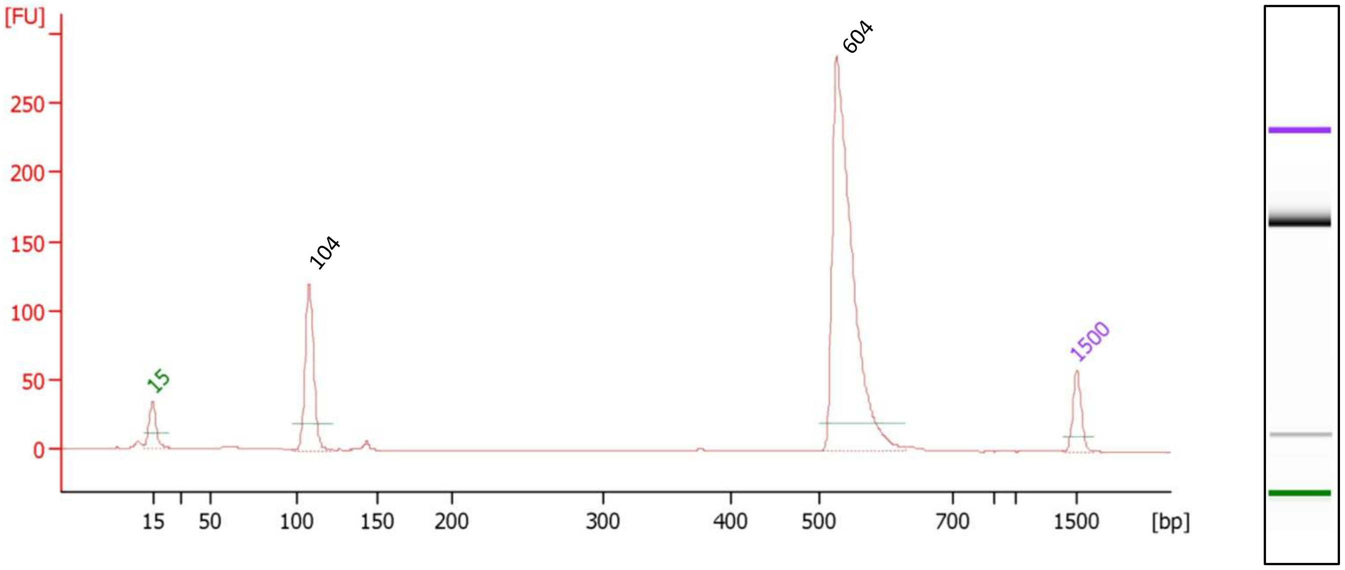

Peak table for Sample #18 :

Peak		Size [bp]	Conc. [ng/µl]	Molarity [nmol/l]	Observations
1		15	4.20	424.4	Lower Marker
2		604	22.32	95.1	Ladder Peak
3		104	15.22	25.4	Ladder Peak
4		1,500	2.10	2.1	Upper Marker

© Copyright 2003 - 2009 Agilent Technologies, Inc.

Assay Class: DNA 1000
Data Path: C:\...-4903611_2023-03-06_NTS_11_20_XhoI_MseI.xad

Created: 3/14/2023 13:38:45 PM
Modified: 3/14/2023 13:38:45 PM

Electrophoresis File Run Summary

Sample #19

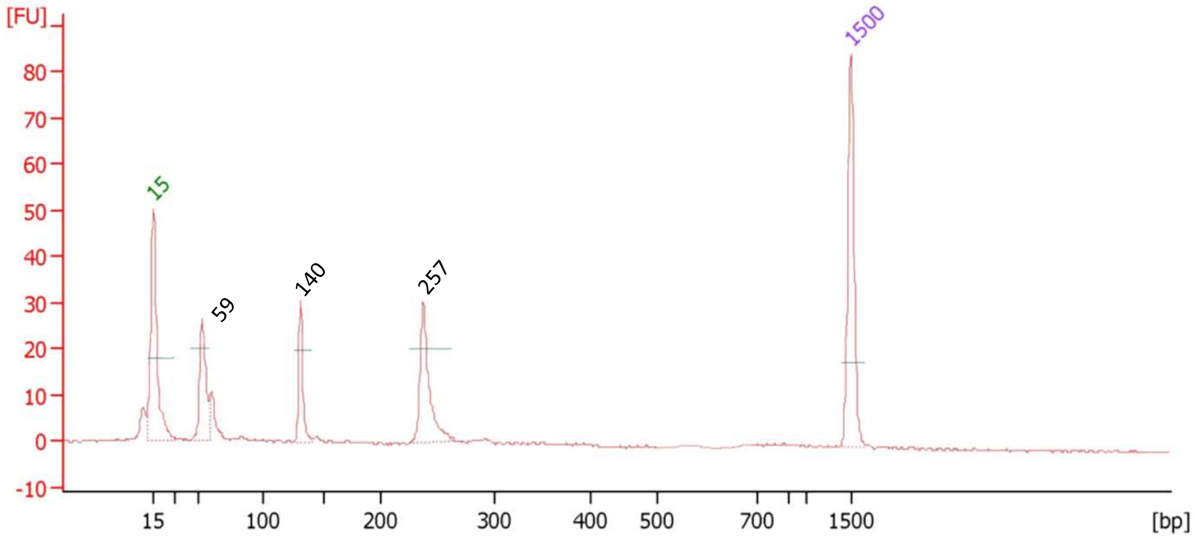

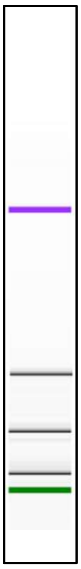

Peak table for Sample #19 :

Peak		Size [bp]	Conc. [ng/µl]	Molarity [nmol/l]	Observations
1	◀	15	4.20	424.2	Lower Marker
2	L	59	8.98	35.8	Ladder Peak
3	L	140	8.47	33.6	Ladder Peak
4	L	257	32.87	102.4	Ladder Peak
5	▶	1,500	2.10	2.1	Upper Marker

 © Copyright 2003 - 2009 Agilent Technologies, Inc.

Assay Class: DNA 1000
Data Path: C:\...-4903611_2023-03-06_NTS_11_20_XhoI_MseI.xad

Created: 3/14/2023 13:38:45 PM
Modified: 3/14/2023 13:38:45 PM

Electrophoresis File Run Summary

Sample #20

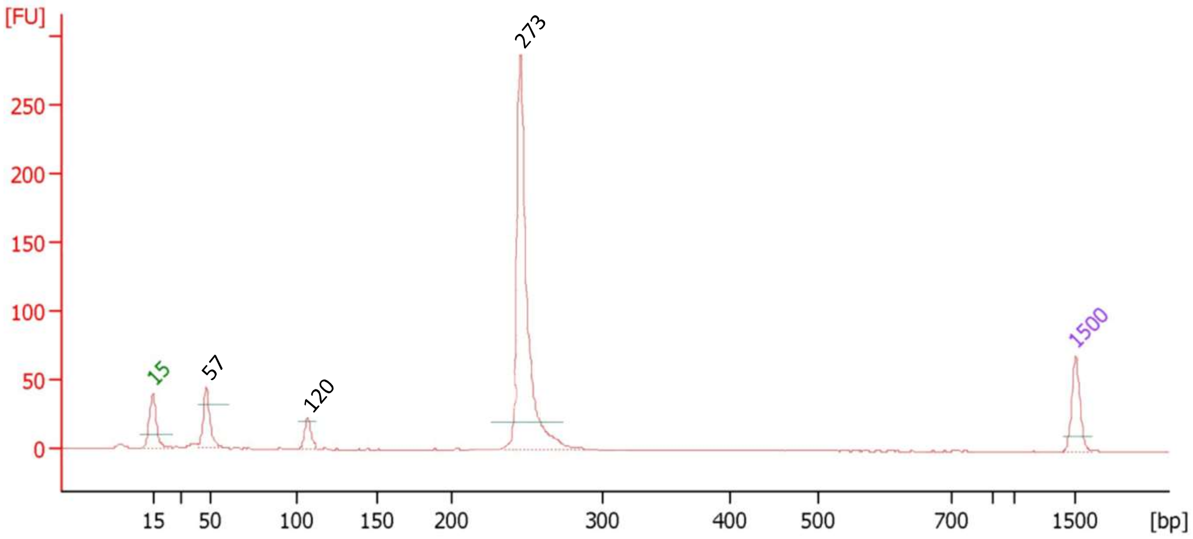

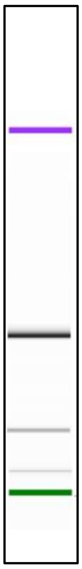

Peak table for Sample #20 :

Peak		Size [bp]	Conc. [ng/µl]	Molarity [nmol/l]	Observations
1		15	4.20	424.4	Lower Marker
2		57	3.21	84.12	Ladder Peak
3		120	2.23	87.22	Ladder Peak
4		273	31.25	95.36	Ladder Peak
5		1,500	2.10	2.2	Upper Marker

Assay Class: DNA 1000
Data Path: C:\...-4903611_2023-03-06_NTS_11_20_XhoI_MseI.xad

Created: 3/14/2023 13:38:45 PM
Modified: 3/14/2023 13:38:45 PM

Electrophoresis File Run Summary

CTRL

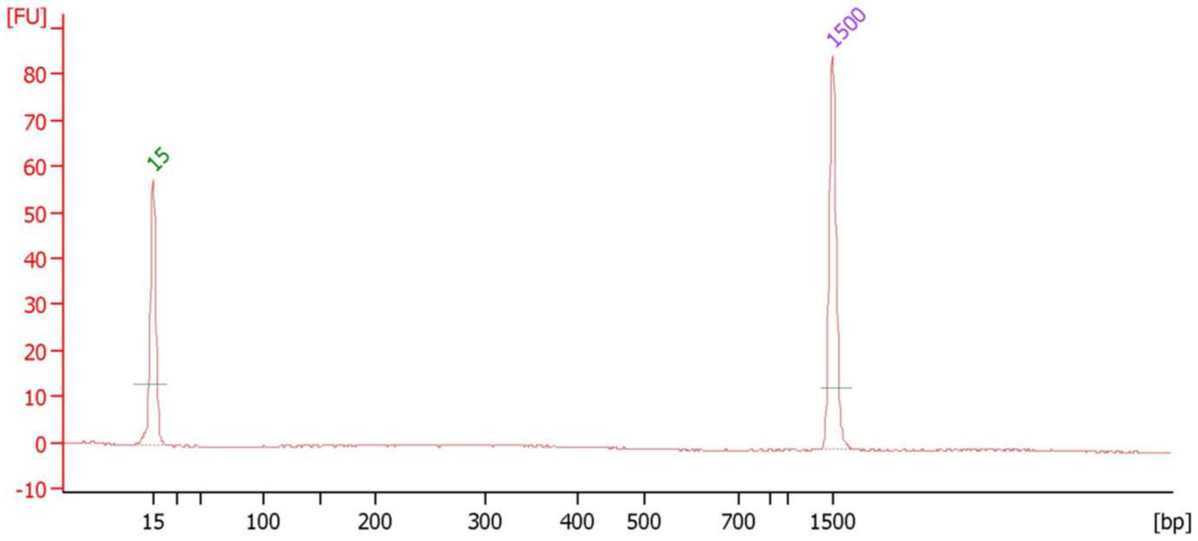

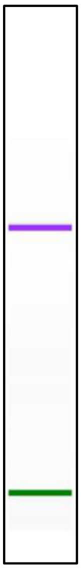

Peak table for Sample CTRL :

Peak		Size [bp]	Conc. [ng/µl]	Molarity [nmol/l]	Observations
**1**	◀	15	4.20	424.4	Lower Marker
**2**	▶	1,500	2.10	2.2	Upper Marker

© Copyright 2003 - 2009 Agilent Technologies, Inc.

Assay Class: DNA 1000
Data Path: C:\...-4903611_2023-03-06_NTS_11_20_XhoI_MseI.xad

Created: 3/14/2023 13:38:45 PM
Modified: 3/14/2023 13:38:45 PM

Electrophoresis File Run Summary

CTRL

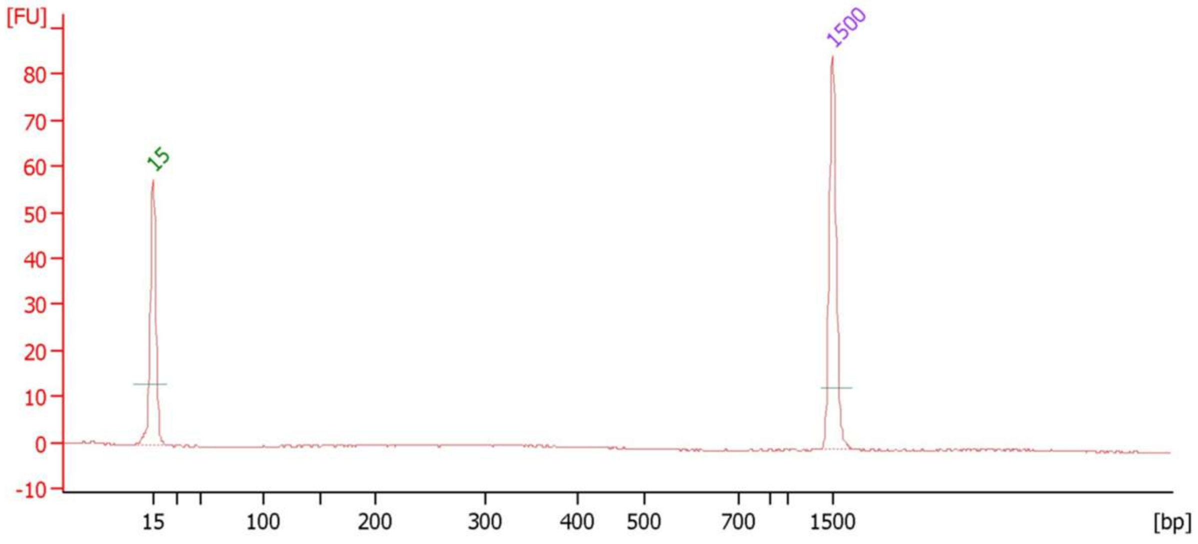

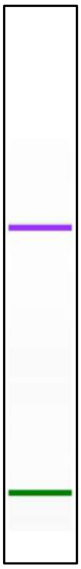

Peak table for Sample CTRL :

Peak		Size [bp]	Conc. [ng/µl]	Molarity [nmol/l]	Observations
**1**	◀	15	4.20	424.4	Lower Marker
**2**	▶	1,500	2.10	2.1	Upper Marker

 © Copyright 2003 - 2009 Agilent Technologies, Inc.

Assay Class: DNA 1000
Data Path: C:\...-4903611_2023-03-06_NTS_11_20_XhoI_MseI.xad

Created: 3/14/2023 13:38:45 PM
Modified: 3/14/2023 13:38:45 PM

Curves

Standard Curve

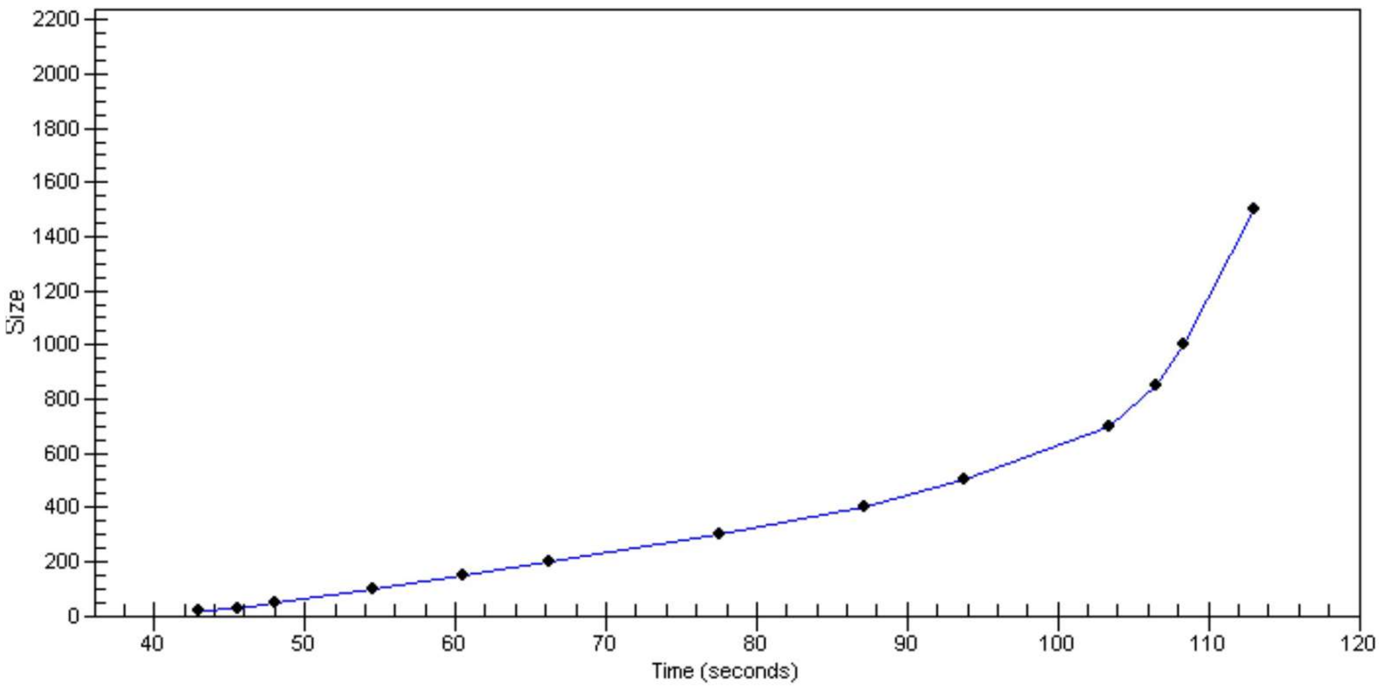

Assay Class: DNA 1000
Data Path: C:\...-4903611_2023-03-06_NTS_11_20_XhoI_MseI.xad

Created: 3/14/2023 13:38:45 PM
Modified: 3/14/2023 13:38:45 PM

Electrophoresis File Run Summary (Chip Summary)

Sample Name	Sample Content	Rest. Digest	Status	Observation	Result Label	Result Color
Sample #1		☐	✓			
Sample #2		☐	✓			
Sample #3		☐	✓			
Sample #4		☐	✓			
Sample #5		☐	✓			
Sample #6		☐	✓			
Sample #7		☐	✓			
Sample #8		☐	✓			
Sample #9		☐	✓			
Sample #10		☐	✓			
CTRL		☐	✓			
CTRL		☐	✓			

Chip Lot # **Reagent Kit Lot #**

Chip Comments :

© Copyright 2003 - 2009 Agilent Technologies, Inc.

Assay Class: DNA 1000
Data Path: C:\...-4903611_2023-03-06_NTS_11_20_XhoI_MseI.xad

Created: 3/14/2023 13:38:45 PM
Modified: 3/14/2023 13:38:45 PM

Electrophoresis Assay Details

Result Label **Result Color**

General Analysis Settings

Number of Available Sample and Ladder Wells (Max.) : 13
Minimum Visible Range [s] : 30
Maximum Visible Range [s] : 129
Start Analysis Time Range [s] : 30
End Analysis Time Range [s] : 128.95
Ladder Concentration [ng/µl] : 44
Uses Standard Area for Ladder Fragments
Lower Marker Concentration [ng/µl] : 4.2
Upper Marker Concentration [ng/µl] : 2.1
Used Upper Marker for Quantitation
Standard Curve Fit is Point to Point
Show Data Aligned to Lower and Upper Marker

Integrator Settings

Integration Start Time [s] : 30
Integration End Time [s] : 128.95
Slope Threshold : 0.5
Height Threshold [FU] : 20
Area Threshold : 0.1
Width Threshold [s] : 0.5
Baseline Plateau [s] : 0.5

Filter Settings

Filter Width [s] : 0.5
Polynomial Order : 4

Ladder

Ladder Peak	Size	Area
1	15	25
2	25	26
3	50	34
4	100	41
5	150	45
6	200	52
7	300	63
8	400	76
9	500	83
10	700	88
11	850	86
12	1000	90
13	1500	52

 © Copyright 2003 - 2009 Agilent Technologies, Inc.
