# Supplementary material for: Discrimination of Green Coffee (Coffea arabica and Coffea canephora) of Different Geographical Origin Based on Antioxidant Activity, High-Throughput Metabolomics, and DNA RFLP Fingerprinting
Source: Antioxidants (Basel). 2023 May 21;12(5):1135. doi: 10.3390/antiox12051135 (PMC10215230; doi:10.3390/antiox12051135)

Supplementary Figure S3: Partitioning on the PC1 and PC2 factor scores of CA e CC accessions according to FAME by GC-MS an GC-FID analysis. See text for abbreviations.

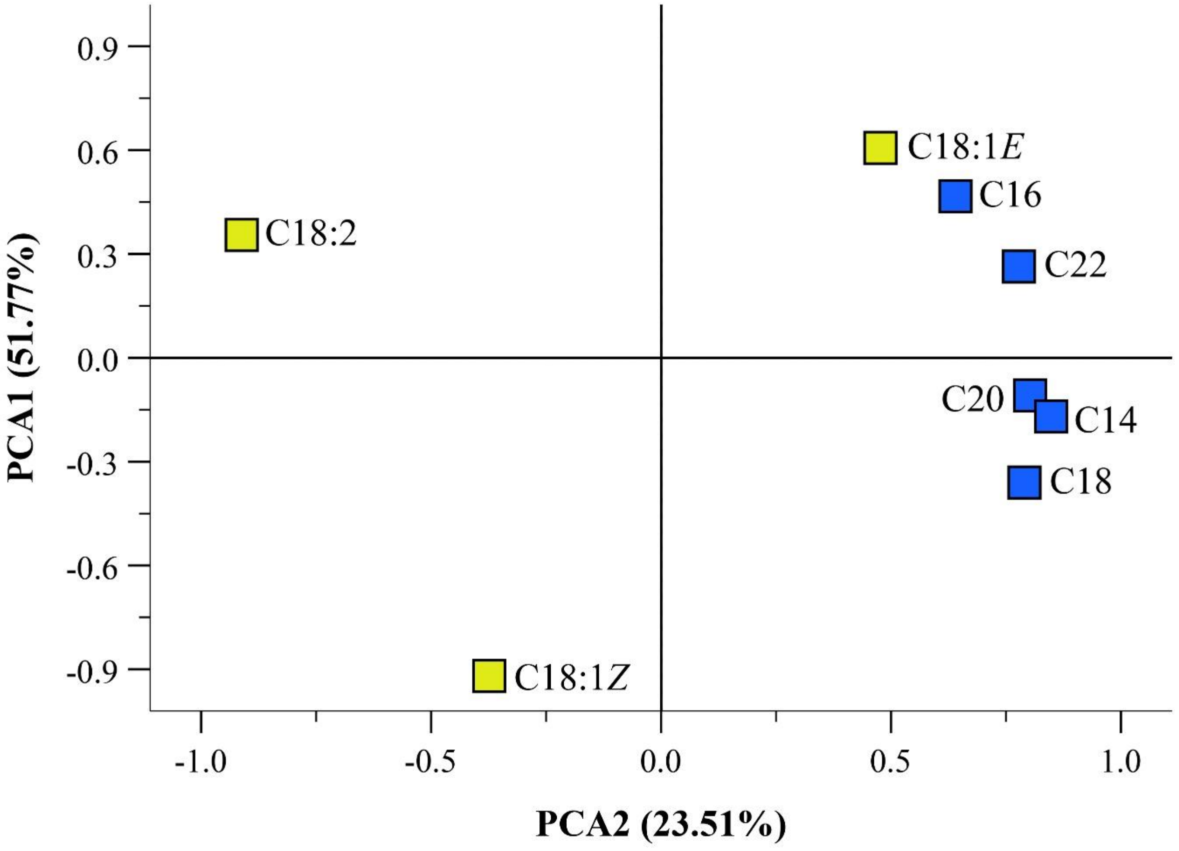

Supplement: Supplementary file 1 [file antioxidants-12-01135-s001.zip › Supplementary-Figure S3.pdf]
