# Supplementary material for: Discrimination of Green Coffee (Coffea arabica and Coffea canephora) of Different Geographical Origin Based on Antioxidant Activity, High-Throughput Metabolomics, and DNA RFLP Fingerprinting
Source: Antioxidants (Basel). 2023 May 21;12(5):1135. doi: 10.3390/antiox12051135 (PMC10215230; doi:10.3390/antiox12051135)

Supplementary Figure S6: Sequence alignment of *5S-rRNA-NTS* region using CLC software (Qiagen, Hilden, Germany). in CA and CC accessions. See Table 1 for the coding of accessions.

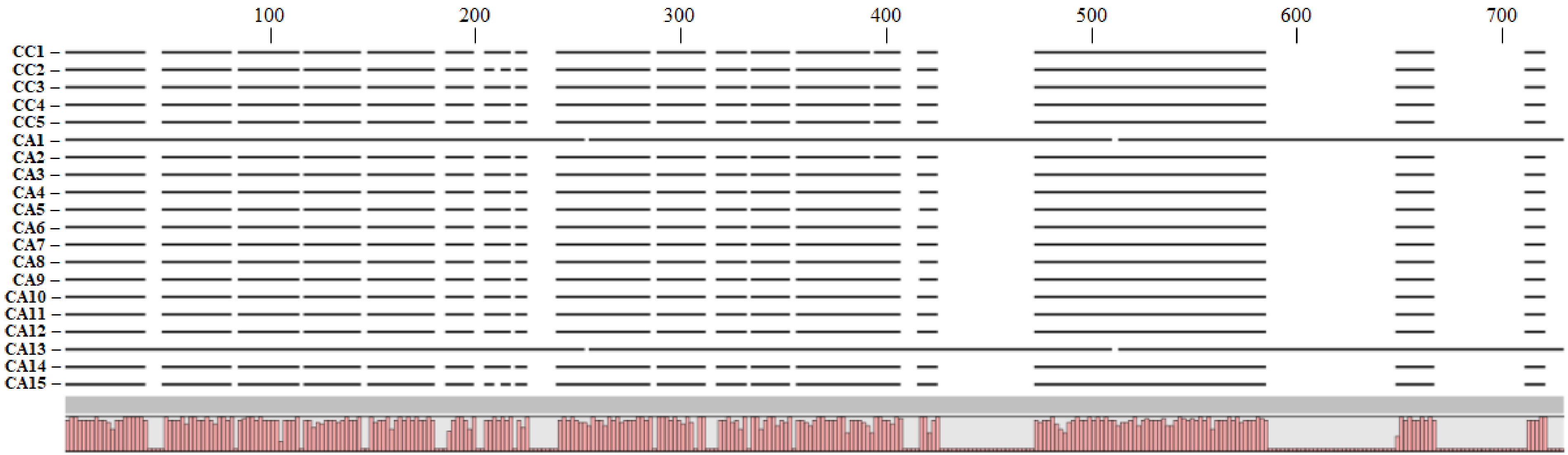

Supplement: Supplementary file 1 [file antioxidants-12-01135-s001.zip › Supplementary-Figure S6.pdf]
