# Supplementary material for: Discrimination of Green Coffee (Coffea arabica and Coffea canephora) of Different Geographical Origin Based on Antioxidant Activity, High-Throughput Metabolomics, and DNA RFLP Fingerprinting
Source: Antioxidants (Basel). 2023 May 21;12(5):1135. doi: 10.3390/antiox12051135 (PMC10215230; doi:10.3390/antiox12051135)
Supplement: Supplementary file 1 [file antioxidants-12-01135-s001.zip › Supplementary-Table S1.pdf]

Supplementary Table S1: Qualitative and quantitative chemical analysis of compounds extracted from CA and CC accessions from different geographical origin and identified by HPLC-ESI-MS/MS analysis.

Compound	CC1	CC2	CC3	CC4	CC5	CA1	CA2	CA3	CA4	CA5	CA6	CA7	CA8	CA9	CA10	CA11	CA12	CA13	CA14	CA15
naringenin (µg/100g)	1.43 ± 0.03	5.19 ± 0.08	10.65 ± 0.41	2.27 ± 0.03	3.2 ± 0.05	5.9 ± 0.14	3.38 ± 0.05	5.91 ± 0.1	2.83 ± 0.08	5.12 ± 0.12	3.36 ± 0.07	7.59 ± 0.21	3.22 ± 0.07	2.79 ± 0.03	8.85 ± 0.3	9.96 ± 0.28	11.81 ± 0.22	3.9 ± 0.14	10.62 ± 0.21	11.04 ± 0.35
kaempferol (µg/100g)	2.49 ± 0.06	1.6 ± 0.05	2.88 ± 0.06	4.55 ± 0.13	9.17 ± 0.2	6.7 ± 0.18	6.89 ± 0.14	6.22 ± 0.08	7.59 ± 0.11	7.46 ± 0.22	8.1 ± 0.22	5.82 ± 0.18	6.42 ± 0.12	10.01 ± 0.15	9.48 ± 0.26	7.81 ± 0.2	9.46 ± 0.37	7.04 ± 0.27	10.75 ± 0.26	6.68 ± 0.22
dihydrokaempferol (µg/100g)	0.28 ± 0.01	0.37 ± 0.01	0.28 ± 0.01	0.24 ± 0.01	0.12 ± 0.01	0.19 ± 0.01	0.15 ± 0.01	0.2 ± 0.01	0.09 ± 0.01	0.16 ± 0.01	0.18 ± 0.01	0.22 ± 0.01	0.17 ± 0.01	0.28 ± 0.01	0.3 ± 0.01	0.28 ± 0.01	0.3 ± 0.01	0.3 ± 0.01	0.28 ± 0.01	0.36 ± 0.01
catechin (µg/100g)	14.72 ± 0.25	10.56 ± 0.25	12.5 ± 0.17	10.14 ± 0.11	17.92 ± 0.62	16.94 ± 0.33	13.23 ± 0.41	22.77 ± 0.57	30.53 ± 0.51	23.86 ± 0.59	20 ± 0.35	13.87 ± 0.35	7.68 ± 0.23	11.2 ± 0.27	13.35 ± 0.16	15 ± 0.23	12.91 ± 0.23	11.45 ± 0.28	17.25 ± 0.17	13.47 ± 0.38
epicatechin (µg/100g)	0.29 ± 0.01	0.53 ± 0.01	1.11 ± 0.04	0.81 ± 0.02	0.69 ± 0.02	0.32 ± 0	0.53 ± 0.01	0.61 ± 0.01	0.67 ± 0.01	0.5 ± 0	0.5 ± 0.01	0.63 ± 0.02	0.41 ± 0.01	0.87 ± 0.02	0.65 ± 0.01	0.79 ± 0.01	0.75 ± 0.02	0.94 ± 0.03	1.02 ± 0.01	0.81 ± 0.01
quercetin (µg/100g)	6.99 ± 0.25	3.17 ± 0.09	16.82 ± 0.63	16.63 ± 0.25	20.76 ± 0.22	24.13 ± 0.37	34.26 ± 0.42	29 ± 0.51	15.91 ± 0.19	14.21 ± 0.21	20.87 ± 0.57	25.62 ± 0.41	15.04 ± 0.3	17.31 ± 0.43	20.99 ± 0.65	11.58 ± 0.39	11.87 ± 0.45	12.08 ± 0.25	22.43 ± 0.65	20.25 ± 0.49
taxifolin (µg/100g)	2.94 ± 0.04	2.68 ± 0.09	3.98 ± 0.1	3.15 ± 0.1	12 ± 0.24	5.48 ± 0.17	4.49 ± 0.17	4.29 ± 0.15	8.77 ± 0.18	5.75 ± 0.14	9.59 ± 0.16	6.01 ± 0.21	7.18 ± 0.16	16.48 ± 0.54	6.14 ± 0.11	6.84 ± 0.14	5.97 ± 0.14	5.65 ± 0.09	7.99 ± 0.31	5.43 ± 0.14
isorhamnetin (µg/100g)	14.02 ± 0.28	13.11 ± 0.36	27.98 ± 0.49	22.09 ± 0.47	10.41 ± 0.33	16.22 ± 0.42	9.05 ± 0.33	16.19 ± 0.41	7.95 ± 0.13	25.35 ± 0.39	20.83 ± 0.51	6.94 ± 0.12	6.12 ± 0.2	9.97 ± 0.22	10.46 ± 0.33	14.4 ± 0.46	19.93 ± 0.35	7.13 ± 0.09	19.19 ± 0.75	14.98 ± 0.5
myricetin (µg/100g)	1.33 ± 0.02	1.04 ± 0.03	1.1 ± 0.01	0.93 ± 0.03	2.11 ± 0.04	1.52 ± 0.05	1.24 ± 0.02	1.97 ± 0.04	2.66 ± 0.07	2.9 ± 0.09	2.29 ± 0.07	1.25 ± 0.04	0.9 ± 0.01	1.29 ± 0.04	1.26 ± 0.02	1.36 ± 0.02	1.6 ± 0.04	1.08 ± 0.01	1.64 ± 0.03	1.52 ± 0.02
ampelopsin (µg/100g)	0.6 ± 0.01	0.99 ± 0.02	2.64 ± 0.03	1.91 ± 0.02	0.52 ± 0.01	1.39 ± 0.04	1.85 ± 0.03	2.96 ± 0.11	0.56 ± 0.02	0.48 ± 0.01	0.45 ± 0.01	0.27 ± 0.01	1.57 ± 0.02	0.36 ± 0.01	0.31 ± 0.01	0.31 ± 0.01	0.31 ± 0.01	0.34 ± 0.01	0.37 ± 0.01	0.35 ± 0.01
kaempferol_rhamnoside (µg/100g)	4.86 ± 0.05	1.71 ± 0.05	2.8 ± 0.04	3.08 ± 0.09	4.01 ± 0.05	2.57 ± 0.06	3.52 ± 0.06	3.67 ± 0.12	4.94 ± 0.05	5.14 ± 0.18	4.64 ± 0.07	4.14 ± 0.12	3.56 ± 0.13	4.49 ± 0.07	3.17 ± 0.03	4.71 ± 0.08	5.18 ± 0.19	4.29 ± 0.06	5.67 ± 0.12	4.41 ± 0.16
hyperoside (µg/100g)	167.56 ± 4.08	509.74 ± 11.23	157.33 ± 3.12	416.92 ± 10.91	438.11 ± 8.47	716.61 ± 12.42	1052.92 ± 20.15	876.31 ± 14.73	1035.22 ± 30.26	392.83 ± 7.76	303.81 ± 11.18	754.74 ± 27.87	582.27 ± 16.5	792.39 ± 22.54	675.65 ± 11.48	851.63 ± 22.67	1127.02 ± 22.41	866.43 ± 18.1	603.1 ± 18.73	1106.3 ± 21.44
naringenin_glucoside (µg/100g)	20.68 ± 0.41	77.2 ± 1.24	118.45 ± 1.77	25.56 ± 0.44	48.59 ± 1.02	63.75 ± 1.12	39.46 ± 0.73	69.35 ± 1.59	42.42 ± 0.58	77.9 ± 2.37	49 ± 0.55	105.81 ± 3.89	46.27 ± 0.79	40.91 ± 1.61	95.96 ± 3.62	127.99 ± 1.35	138.94 ± 4.23	53.35 ± 1.42	133.69 ± 3.95	133.89 ± 3.8
naringenin_galactoside (µg/100g)	4.59 ± 0.07	2.4 ± 0.07	5.52 ± 0.14	6.47 ± 0.1	3.95 ± 0.09	8.36 ± 0.11	5.12 ± 0.19	2.14 ± 0.02	4.71 ± 0.07	7.07 ± 0.25	4.07 ± 0.16	2.51 ± 0.08	4.55 ± 0.1	3.71 ± 0.08	3.59 ± 0.13	3.28 ± 0.05	4.49 ± 0.13	3.91 ± 0.11	3.37 ± 0.04	3.74 ± 0.14
catechin_sambubioside (µg/100g)	2.17 ± 0.03	2.39 ± 0.05	1.85 ± 0.02	2.19 ± 0.05	3.05 ± 0.1	2.2 ± 0.07	2.92 ± 0.06	1.87 ± 0.06	2.49 ± 0.09	2.48 ± 0.06	2.34 ± 0.07	2.89 ± 0.06	2.71 ± 0.07	2.65 ± 0.06	3.55 ± 0.1	3.24 ± 0.1	2.11 ± 0.05	3.14 ± 0.08	2.4 ± 0.06	2.43 ± 0.08

**quercitrin (μg/100g)**	140.91 ± 5.01	82.71 ± 1.49	100.96 ± 1.87	42.25 ± 1.6	567.33 ± 13.41	282.73 ± 5.38	186.09 ± 6.53	224.38 ± 6.05	311.72 ± 6.97	170.77 ± 5.41	244.57 ± 5.94	241.66 ± 7.95	136.54 ± 2.15	189.69 ± 5.61	277.37 ± 3.99	463.94 ± 8.02	368.85 ± 5.36	265.03 ± 9.18	281.61 ± 5.77	518.53 ± 14.14
**taxifolin_galactoside (μg/100g)**	27.73 ± 0.96	42.23 ± 0.53	59.93 ± 1.56	29.73 ± 0.96	15.81 ± 0.58	17.33 ± 0.68	39.59 ± 0.82	15.83 ± 0.29	19.73 ± 0.69	18.1 ± 0.7	23.64 ± 0.23	19.64 ± 0.46	19.2 ± 0.2	32.44 ± 0.72	13.1 ± 0.38	25.65 ± 0.26	28.23 ± 0.7	39.27 ± 0.67	15.18 ± 0.46	25.76 ± 0.95
**taxifolin_glucoside (μg/100g)**	26.83 ± 1.01	17.73 ± 0.22	13.89 ± 0.26	38.03 ± 0.43	10.48 ± 0.41	13.66 ± 0.18	5.86 ± 0.21	3.22 ± 0.09	11.44 ± 0.34	17.86 ± 0.47	28.5 ± 1.05	11.16 ± 0.28	10.43 ± 0.19	21.1 ± 0.64	12.99 ± 0.36	10.02 ± 0.26	32.32 ± 0.63	11.19 ± 0.43	15.96 ± 0.62	5.59 ± 0.18
**naringenin_sambubioside (μg/100g)**	31.55 ± 0.51	70.86 ± 1.39	35.42 ± 0.41	86.96 ± 2.87	79.37 ± 2.68	23.79 ± 0.8	66.14 ± 2.09	50.18 ± 0.54	92.64 ± 3.3	70.56 ± 2.23	65.19 ± 2.33	52.9 ± 0.58	47.28 ± 1.42	71.88 ± 2.51	52.88 ± 1.85	36.32 ± 1.01	44.18 ± 0.68	49.92 ± 1.01	45.58 ± 0.83	40.46 ± 0.7
**naringenin_rutinoside (μg/100g)**	18.48 ± 0.26	17.94 ± 0.37	15.79 ± 0.32	3.67 ± 0.1	11.49 ± 0.17	12.73 ± 0.19	9.72 ± 0.14	10.53 ± 0.11	8.46 ± 0.31	6.16 ± 0.24	9.12 ± 0.2	10.39 ± 0.37	10.62 ± 0.11	10.51 ± 0.38	8.08 ± 0.15	10.34 ± 0.27	12.92 ± 0.16	11.6 ± 0.4	13.01 ± 0.31	10.15 ± 0.39
**kaempferol_sambubioside (μg/100g)**	7.27 ± 0.08	1.65 ± 0.02	2.96 ± 0.1	2.75 ± 0.03	8.14 ± 0.2	5.88 ± 0.16	7.26 ± 0.16	5.34 ± 0.11	7.88 ± 0.12	6.36 ± 0.23	6.5 ± 0.16	6.05 ± 0.22	4.6 ± 0.14	6.82 ± 0.17	9.59 ± 0.28	7.77 ± 0.19	10.72 ± 0.41	6.75 ± 0.16	10.07 ± 0.24	7.5 ± 0.22
**dihydrokaempferol_sambioside (μg/100g)**	8.88 ± 0.2	5.27 ± 0.12	3.28 ± 0.11	11.22 ± 0.26	9.6 ± 0.19	7.47 ± 0.23	7.28 ± 0.09	8.56 ± 0.12	9.41 ± 0.33	6.99 ± 0.24	7.1 ± 0.18	7.4 ± 0.12	7.63 ± 0.23	8.36 ± 0.09	6.34 ± 0.15	7.71 ± 0.19	9.41 ± 0.13	7.22 ± 0.15	7.88 ± 0.28	7.81 ± 0.18
**kaempferol_rutinoside (μg/100g)**	51.72 ± 0.78	40.09 ± 0.95	74.01 ± 1.23	79.9 ± 0.86	182.48 ± 3.51	135.72 ± 4.78	145.57 ± 5.28	144.38 ± 2.37	159.55 ± 5.81	172.24 ± 6.19	176.27 ± 2.14	143.83 ± 5.56	120.33 ± 1.35	182.75 ± 6.8	235.85 ± 7.03	172.99 ± 6.43	243.45 ± 5.72	139.48 ± 4.65	199.9 ± 2.89	150.49 ± 2.08
**quercetin_glucuronide (μg/100g)**	18.82 ± 0.31	11.19 ± 0.43	44.58 ± 1.14	39.46 ± 1.43	36.46 ± 0.48	12.74 ± 0.26	9.41 ± 0.26	22.13 ± 0.7	13.23 ± 0.27	47.27 ± 1.14	51.97 ± 0.62	21.65 ± 0.79	28.47 ± 1.05	34.41 ± 0.91	48 ± 1.46	30.08 ± 0.98	52.06 ± 0.54	29.36 ± 0.33	32.63 ± 0.43	39.12 ± 0.78
**taxifolin_sambubioside (μg/100g)**	13.8 ± 0.3	10.27 ± 0.34	11.87 ± 0.27	14.01 ± 0.21	22.87 ± 0.29	12.16 ± 0.31	9.88 ± 0.35	12.74 ± 0.4	13.08 ± 0.19	9.82 ± 0.12	11.39 ± 0.25	16.12 ± 0.24	19.3 ± 0.59	20.92 ± 0.62	14.09 ± 0.17	20.97 ± 0.35	13.49 ± 0.48	23.73 ± 0.72	17.46 ± 0.45	25.96 ± 1.03
**rutin (μg/100g)**	171.11 ± 6.69	151.6 ± 3.37	116.36 ± 3.42	150.45 ± 4.07	285.85 ± 10.73	655.49 ± 25.4	225.39 ± 3.29	197.14 ± 6.1	222.59 ± 3.35	249.44 ± 7.47	247.35 ± 5.23	270.76 ± 7.94	345.38 ± 9.9	257.34 ± 4.42	239.75 ± 5.82	231.07 ± 2.59	328.26 ± 6.21	353.06 ± 6.08	344.41 ± 3.57	335.57 ± 6.33
**taxifolin_rutinoside (μg/100g)**	141.85 ± 2.12	171.59 ± 1.89	161.29 ± 4.59	135.03 ± 2.21	94.01 ± 1.93	132.69 ± 5.02	186.55 ± 3.05	52.34 ± 1.36	132.52 ± 4.84	119.41 ± 3.27	182.86 ± 7.18	111.28 ± 2.46	79.17 ± 2.89	124.57 ± 1.97	159.29 ± 4.76	180.12 ± 4.48	367.51 ± 4.46	227.4 ± 7.32	159.26 ± 5.37	165.42 ± 3.67
**ampelopsin_rutin (μg/100g)**	1.56 ± 0.05	0.89 ± 0.03	1.37 ± 0.05	1.55 ± 0.05	1.17 ± 0.03	0.73 ± 0.01	0.77 ± 0.01	0.94 ± 0.02	1.13 ± 0.02	1.1 ± 0.04	1.55 ± 0.01	1.74 ± 0.06	1.4 ± 0.03	1.7 ± 0.05	2.09 ± 0.07	1.24 ± 0.02	1.73 ± 0.06	1.42 ± 0.04	1.76 ± 0.03	0.95 ± 0.02
**theobromine (mg/g)**	1.15 ± 0.03	1.1 ± 0.03	1.01 ± 0.02	0.89 ± 0.02	1.21 ± 0.04	0.76 ± 0.01	1.18 ± 0.03	1.44 ± 0.03	0.82 ± 0.01	0.9 ± 0.02	1.03 ± 0.02	2.2 ± 0.07	1.5 ± 0.04	1.44 ± 0.05	1.11 ± 0.03	0.94 ± 0.01	1.22 ± 0.02	1.46 ± 0.02	1.13 ± 0.02	1.45 ± 0.04
**theophyllin (mg/g)**	0.88 ± 0.02	0.45 ± 0.01	0.55 ± 0.01	0.63 ± 0.02	0.48 ± 0.01	0.32 ± 0.01	0.29 ± 0.01	0.35 ± 0.01	0.31 ± 0.01	0.43 ± 0.01	0.47 ± 0.01	0.63 ± 0.01	0.42 ± 0.01	0.43 ± 0.01	0.43 ± 0.01	0.6 ± 0.01	0.61 ± 0.01	0.74 ± 0.01	0.43 ± 0.01	0.47 ± 0.01
**caffeine (mg/g)**	12.68 ± 0.5	23.69 ± 0.72	21.19 ± 1.57	21.84 ± 1.13	17.76 ± 0.55	8.65 ± 0.63	7.7 ± 0.44	7.37 ± 0.2	7.21 ± 0.33	9.02 ± 0.28	8.59 ± 0.45	8.37 ± 0.32	8.96 ± 0.28	9.54 ± 0.63	8.89 ± 0.17	8.75 ± 0.51	9.2 ± 0.23	7.59 ± 0.15	9.24 ± 0.4	8.8 ± 0.57
**melatonin (μg/g)**	6.87 ± 0.23	6.47 ± 0.09	8.07 ± 0.23	9.32 ± 0.1	8.12 ± 0.21	4.13 ± 0.08	5.73 ± 0.21	6.24 ± 0.14	8.11 ± 0.14	7.37 ± 0.17	4.42 ± 0.12	5.4 ± 0.17	4.53 ± 0.09	7.06 ± 0.13	4.4 ± 0.13	4.28 ± 0.08	5.07 ± 0.16	7.13 ± 0.2	4.91 ± 0.1	5.55 ± 0.16
