# Supplementary material for: Discrimination of Green Coffee (Coffea arabica and Coffea canephora) of Different Geographical Origin Based on Antioxidant Activity, High-Throughput Metabolomics, and DNA RFLP Fingerprinting
Source: Antioxidants (Basel). 2023 May 21;12(5):1135. doi: 10.3390/antiox12051135 (PMC10215230; doi:10.3390/antiox12051135)
Supplement: Supplementary file 1 [file antioxidants-12-01135-s001.zip › Supplementary-Table S2.pdf]

Suppelemntary Table S2: Qualitative and quantitative composition of fatty acids present in CA and CC accessions from different geographical origin and identified and quantified by GC-MS and GC-FID. See text for abbreviations.

	C14	C16	C18:2	C18:1 Z	C18:1 E	C18	C20	C22	Total
**CC1**	0.078 ± 0.002^{ab}	31.791 ± 0.601^{g}	32.947 ± 0.496^{gh}	11.342 ± 0.113^{a}	0.596 ± 0.021^{ghi}	7.262 ± 0.225^{de}	3.464 ± 0.061^{cde}	0.961 ± 0.023^{m}	88.446 ± 3.320^{fg}
**CC2**	0.082 ± 0.002^{a}	22.638 ± 0.641^{lm}	22.692 ± 0.373^{l}	7.169 ± 0.239^{e}	0.381 ± 0.005^{n}	4.616 ± 0.100^{l}	2.135 ± 0.034^{h}	0.536 ± 0.008^{op}	60.253 ± 1.817^{l}
**CC3**	0.062 ± 0.001^{fg}	27.342 ± 1.015^{h}	28.809 ± 0.952^{i}	8.042 ± 0.238^{cd}	0.511 ± 0.005^{lm}	6.099 ± 0.211^{h}	3.456 ± 0.122^{cde}	1.075 ± 0.033^{l}	75.399 ± 2.165^{hi}
**CC4**	0.033 ± 0.001^{no}	8.491 ± 0.269^{o}	7.683 ± 0.278^{m}	2.074 ± 0.055^{i}	0.185 ± 0.005^{o}	1.599 ± 0.057^{no}	0.86 ± 0.031^{lm}	0.246 ± 0.006r	21.173 ± 0.649^{n}
**CC5**	0.026 ± 0.001^{p}	16.54 ± 0.583^{n}	78.186 ± 3.102^{a}	1.988 ± 0.045^{i}	0.146 ± 0.005^{o}	1.898 ± 0.056^{n}	0.963 ± 0.011^{l}	0.495 ± 0.011^{p}	100.245 ± 2.289^{cd}
**CA1**	0.060 ± 0.001^{g}	34.908 ± 0.521^{ef}	33.381 ± 1.099^{fgh}	7.310 ± 0.202^{e}	0.696 ± 0.025^{de}	7.839 ± 0.260^{bc}	3.447 ± 0.053^{cde}	1.443 ± 0.048^{cd}	89.087 ± 3.136^{efg}
**CA2**	0.072 ± 0.001^{cd}	36.885 ± 0.61^{e}	38.763 ± 0.976^{de}	8.211 ± 0.163^{cd}	0.806 ± 0.01^{c}	7.066 ± 0.077^{ef}	3.153 ± 0.047^{ef}	1.351 ± 0.041^{def}	96.309 ± 1.932^{cde}
**CA3**	0.033 ± 0.001^{n}	60.763 ± 1.078^{a}	49.946 ± 1.891^{b}	1.115 ± 0.035^{l}	0.181 ± 0.006^{o}	1.291 ± 0.015^{o}	0.735 ± 0.028^{m}	0.358 ± 0.005q	114.427 ± 1.934^{b}
**CA4**	0.041 ± 0.001^{m}	46.122 ± 1.575^{b}	52.159 ± 1.749^{b}	11.469 ± 0.303^{a}	1.123 ± 0.034^{a}	10.57 ± 0.115^{a}	5.007 ± 0.087^{a}	2.072 ± 0.063^{a}	128.568 ± 2.879^{a}
**CA5**	0.063 ± 0.002^{ef}	32.519 ± 0.546^{fg}	31.6 ± 0.597^{hi}	5.761 ± 0.165^{f}	0.653 ± 0.017^{ef}	6.745 ± 0.156^{fg}	3.898 ± 0.121^{b}	1.533 ± 0.041^{c}	82.775 ± 2.463^{gh}
**CA6**	0.066 ± 0.001^{ef}	26.831 ± 0.731^{hi}	28.075 ± 0.904^{i}	5.321 ± 0.127^{fg}	0.568 ± 0.021^{hi}	5.538 ± 0.126^{i}	3.214 ± 0.06^{ef}	1.263 ± 0.028^{fgh}	70.879 ± 1.734^{i}
**CA7**	0.048 ± 0.001^{hi}	20.905 ± 0.315^{m}	20.028 ± 0.51^{l}	4.396 ± 0.163^{h}	0.471 ± 0.014^{m}	3.937 ± 0.080^{m}	1.647 ± 0.035^{i}	0.594 ± 0.016^{o}	52.030 ± 1.471^{m}
**CA8**	0.047 ± 0.001^{il}	43.467 ± 1.287^{c}	49.015 ± 0.53^{b}	9.541 ± 0.097^{b}	1.160 ± 0.026^{a}	8.225 ± 0.185^{b}	3.769 ± 0.051^{b}	1.689 ± 0.027^{b}	116.917 ± 4.475^{b}
**CA9**	0.049 ± 0.001^{hi}	27.361 ± 0.423^{h}	30.672 ± 0.628^{hi}	5.584 ± 0.13^{f}	0.612 ± 0.012^{fgh}	5.273 ± 0.063^{i}	2.499 ± 0.091^{g}	0.818 ± 0.027^{n}	72.871 ± 1.145^{i}
**CA10**	0.077 ± 0.002^{cb}	24.391 ± 0.787^{il}	22.340 ± 0.693^{l}	4.807 ± 0.088^{gh}	0.546 ± 0.015^{il}	5.477 ± 0.175^{i}	3.070 ± 0.049^{f}	1.334 ± 0.027^{efg}	62.046 ± 1.578^{l}
**CA11**	0.042 ± 0.001^{lm}	39.444 ± 0.509^{d}	42.825 ± 0.855^{c}	7.851 ± 0.238^{d}	0.798 ± 0.013^{c}	7.239 ± 0.215^{de}	3.834 ± 0.076^{b}	1.476 ± 0.028^{c}	103.511 ± 3.080^{c}
**CA12**	0.045 ± 0.001^{lm}	43.571 ± 0.682^{a}	48.782 ± 0.877^{b}	8.423 ± 0.234^{c}	0.871 ± 0.009^{b}	7.595 ± 0.086^{cd}	3.980 ± 0.110^{b}	1.366 ± 0.033^{de}	114.637 ± 2.211^{b}
**CA13**	0.028 ± 0.001^{op}	35.279 ± 0.487^{e}	41.296 ± 0.496^{cd}	7.029 ± 0.175^{e}	0.723 ± 0.02^{d}	6.384 ± 0.144^{gh}	3.498 ± 0.035^{c}	1.241 ± 0.034^{ghi}	95.481 ± 3.402^{def}
**CA14**	0.052 ± 0.001^{h}	32.191 ± 1.211^{g}	36.002 ± 0.756^{efg}	6.794 ± 0.159^{e}	0.579 ± 0.022^{hi}	6.432 ± 0.169^{gh}	3.250 ± 0.044^{def}	1.196 ± 0.028^{hi}	86.501 ± 2.531^{g}
**CA15**	0.068 ± 0.001^{de}	34.714 ± 0.529^{ef}	36.744 ± 1.135^{ef}	6.911 ± 0.073^{e}	0.635 ± 0.009^{fgh}	6.403 ± 0.084^{gh}	3.317 ± 0.099^{cde}	1.159 ± 0.029^{il}	89.954 ± 1.555^{efg}
